# Deletion of ARGLU1 causes global defects in alternative splicing in vivo and mouse cortical malformations primarily via apoptosis

**DOI:** 10.1038/s41419-023-06071-w

**Published:** 2023-08-23

**Authors:** Fenyong Yao, Shisheng Huang, Jiahui Liu, Chunhua Tan, Mengqi Xu, Dengkui Wang, Maoqing Huang, Yiyao Zhu, Xingxu Huang, Shuijin He

**Affiliations:** 1 School of Life Science and Technology, ShanghaiTech University, 393 Middle Huaxia Road, Pudong New District, 201210 Shanghai, China; 2 Institute of Neuroscience, Shanghai Institutes for Biological Sciences, Chinese Academy of Sciences, Shanghai, China; 3 University of Chinese Academy of Sciences, Beijing, China; 4 School of Information Science and Technology, ShanghaiTech University, 393 Middle Huaxia Road, Pudong New District, 201210 Shanghai, China; 5 Shanghai Clinical Research and Trial Center, 201210 Shanghai, China

**Keywords:** Developmental neurogenesis, Apoptosis

## Abstract

Haploinsufficient mutation in arginine and glutamine-rich protein 1 (*Arglu1*), a newly identified pre-mRNA splicing regulator, may be linked to neural developmental disorders associated with mental retardation and epilepsy in human patients, but the underlying causes remain elusive. Here we show that ablation of *Arglu1* promotes radial glial cell (RG) detachment from the ventricular zone (VZ), leading to ectopic localized RGs in the mouse embryonic cortex. Although they remain proliferative, ectopic progenitors, as well as progenitors in the VZ, exhibit prolonged mitosis, p53 upregulation and cell apoptosis, leading to reduced neuron production, neuronal loss and microcephaly. RNA seq analysis reveals widespread changes in alternative splicing in the mutant mouse embryonic cortex, preferentially affecting genes involved in neuronal functions. *Mdm2* and *Mdm4* are found to be alternatively spliced at the exon 3 and exon 5 respectively, leading to absence of the p53-binding domain and nonsense-mediated mRNA decay (NMD) and thus relieve inhibition of p53. Removal of p53 largely rescues the microcephaly caused by deletion of *Arglu1*. Our findings provide mechanistic insights into cortical malformations of human patients with *Arglu1* haploinsufficient mutation.

## Introduction

The cerebral cortex is a complex structure that comprises billions of neurons and commands all higher-order brain functions. During embryonic development, RGs give rise to nearly all cortical excitatory neurons via direct or indirect neurogenesis [1]. After the onset of neurogenesis, a RG undergoes asymmetric division to directly give rise to a postmitotic neuron and a daughter RG for self-renewal [2–5]. In addition, RGs indirectly produce excitatory neurons via intermediate progenitors (IPs) that generate neurons via symmetric divisions in the subventricular zone (SVZ), the other two transient amplifying progenitors including short neural precursors [6] and outer subventricular zone radial glial cells (oRGs, also called basal RGs (bRGs)) [7, 8], particularly in the primate cortex there are a substantial amount of oRGs assumed to be responsible for cortical expansion. Newly generated neurons migrate into the cortical plate in a birthdate-dependent inside-out manner [9–11], ultimately establishing the laminated structure of the neocortex consisting of six layers.

Human patients with mutations in *Arglu1* display developmental delay and intellectual disability, in some cases concurrent with neurological disorders such as epilepsy and autistic spectrum. ARGLU1 is an evolutionarily conserved protein that consists of two distinct domains, a positively charged arginine-rich N-terminus and a negatively charged glutamate-rich C-terminus that preferentially interact with spliceosomes and nuclear receptors [12, 13], respectively. ARGLU1 was originally identified as an activator for estrogen-dependent gene expression in breast cancer cells by interacting with MED1 (Mediator subunit 1) [14]. Two recent studies show that ARGLU1 is also an AS regulator in vitro, capable of mediating its own splicing via a non-coding ultraconserved element (UCE) [15] and other protein pre-mRNAs splicing that likely depends on its N-terminus [12]. In contrast, the C-terminus is more likely responsible for regulation of gene transcription via binding to nuclear receptors. *Arglu1* mutation in human patients is often accompanied with absence of a few neighboring genes [16, 17], raising a question as to whether *Arglu1* mutation causes malformations of cortical development. Therefore, establishing an *Arglu1* knockout mouse model is crucial to demonstrate an association between *Arglu1* and cortical development as well as related neurological diseases.

In this study, we employed a conditional null allele to delete *Arglu1* by Emx1-Cre recombination in developing mouse cortex. We found that a massive number of progenitors were delocalized into the cortical plate and the intermediate zone after removal of *Arglu1*. Although they remained proliferative, these ectopic progenitors, as well as those in the VZ, resulted in less cortical excitatory neurons generated owing to a delay in mitotic progression. Aside from that, loss of *Arglu1* significantly resulted in increased number of cell expressing cleaved Capase-3 (CASP3) and upregulation of p53 in the developing cortex. RNA seq revealed changes in alternative splicing and gene transcription in cKO mice, demonstrating dual roles of ARGLU1 in splicing and gene transcription regulation in vivo. Of those alternatively spliced genes, *Mdm2* and *Mdm4* underwent skipping of exon 3 and exon 5, leading to an increase in p53 activity due to absence of a p53-binding domain and a nonsense-mediated mRNA decay (NMD) of MDM4, respectively. Removal of p53 completely prevented cell apoptosis and largely rescued the microcephaly, suggesting that the aberrant MDM2/4-p53 axis is mainly responsible for microcephaly.

## Results

### *Arglu1* deletion leads to microcephaly

**Fig. 1:**
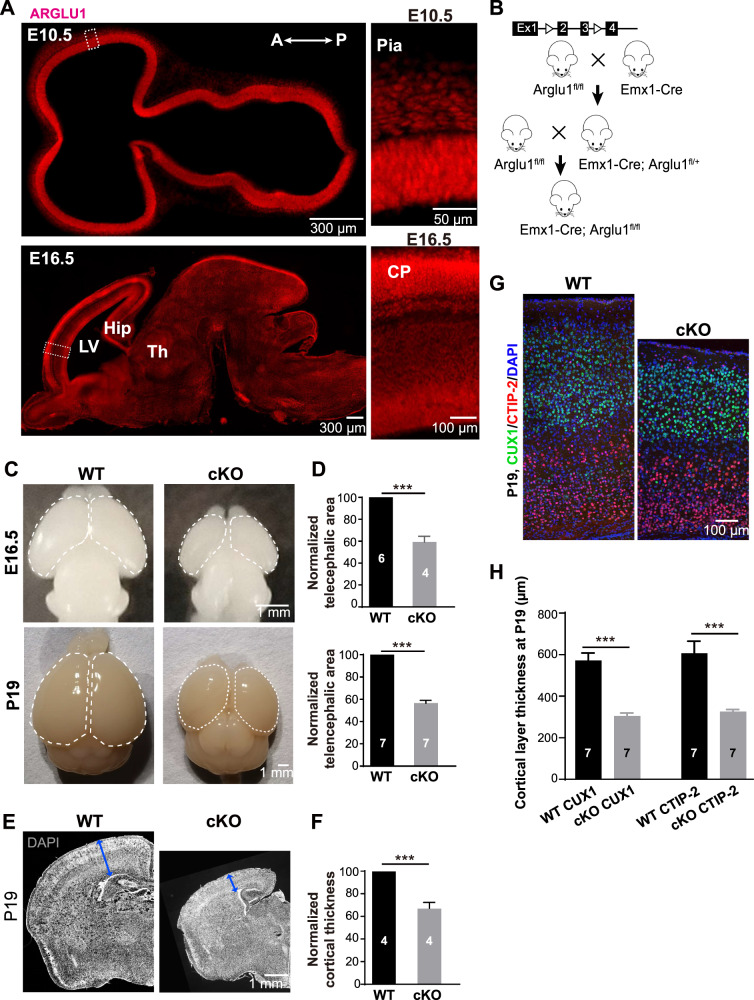
*Arglu1* deletion causes microcephaly in mice. **A** Profiling of ARGLU1 expression in developing mouse cortex. Representative images of E10.5 and E16.5 cortices stained for ARGLU1 from WT mice. Right panels are expanded from the corresponding boxed regions in the left. Note that ARGLU1 is robustly expressed in the cortex regardless of cell types throughout development. LV lateral ventricle, A anterior, P posterior, Hip hippocampus, Th thalamus. **B** The breeding strategy to generate conditional *Arglu1* null mice by crossing double floxed mice that contain two loxp sites flanking exons 2 and 3 with the Emx1-Cre line. **C** Representative whole-mount images of brains from wild-type (WT) and conditional knockout (cKO) mice at embryonic day 16.5 (E16.5) (top) and postnatal day 19 (P19) (bottom). **D** Quantification of the telencephalic area for WT and cKO brains (circled in **C**) at E16.5 (top) and P19 (bottom) (E16.5: WT, *n* = 6; cKO, *n* = 4; P19-P21: WT, *n* = 7; cKO, *n* = 7 mouse brains; ***P < 0.001, two-tailed Student *t* test). **E**, **F** Representative images of cortices stained for DAPI (**E**) and quantification of cortical thickness (**F**) for WT and cKO mice at P19-P21 (Cortex: WT, *n* = 4; cKO, *n* = 4 mouse brains; ***P < 0.001, two-tailed Student *t* test). **G**, **H** Representative images and quantification of P19-P21 cortices stained for CUX1 and CTIP-2 (WT, *n* = 7; cKO, *n* = 7 mouse brains; ***P < 0.001, Two-way ANOVA with *post hoc* bonferroni test). Note that *Arglu1* deletion significantly reduced the cortical area and cortical thickness regardless of the cortical layer. Bar graph data are represented as mean ± SEM.

While ARGLU1 is highly expressed in the central nervous system [12], little is known about its expression profile in the developing mouse brain. We performed immunohistochemistry (IHC) on sagittal mouse brain tissue sections at different stages of development. We found that ARGLU1 was robustly expressed in the ventricular zone of embryonic brains and the cortical plate throughout embryonic development (Fig. 1A). After birth, ARGLU1 was expressed highly in the neocortex, the hippocampus and the thalamus, but significantly low in the midbrain and the hindbrain (Supplementary Fig. 1).

To determine the role of *Arglu1* in cortical development, we sought to ablate expression of *Arglu1* in cortical excitatory neural progenitors and their progeny by crossing Emx1-Cre mice with *Arglu1* double floxed mice (*Arglu1*^*fl/fl*^) that contain two loxP sites flanking exons 2 and 3 (Fig. 1B). Immunostaining analyses confirmed significant knockdown of ARGLU1 in the *Emx1*^*Cre/+*^-*Arglu1*^*fl/fl*^ mouse cortex (referred to as *Arglu1* cKO) compared to wild-type controls (WT, referred to *Arglu1*^*fl/+*^ or *Arglu1*^*fl/fl*^ mice) (Supplementary Fig. 2A). Note that *Arglu1* cKO mice showed obvious microcephalic phenotypes at both developmental and mature stages. Compared with WT littermate controls, the telencephalic area that was measured by circumference was reduced by 40.9 ± 4.2%, 47.1 ± 2.7% and 43.5 ± 2.6% in *Arglu1* cKO mice at E16.5, at birth and at P19-P21 (Fig. 1C, D and Supplementary Fig. 3A, B), respectively. Moreover, the cortical thickness was reduced by 28.2 ± 1.9% and 32.8 ± 5.2% in cKO mice relative to WT controls at birth and P19, respectively (Supplementary Fig. 3C and Fig. 1E, F). To determine whether the cortical lamination was affected, we performed immunostaining of P19 brain sections for CUX1 (an upper cortical layer marker) and CTIP-2 (a lower cortical layer marker). While both CUX1+ cell- and CTIP-2+ cell-containing layer thicknesses were significantly reduced, the relative layer thickness was comparable between cKO and WT controls (CUX1: WT, 40.4 ± 2.49; cKO, 41.5 ± 0.44; CTIP-2: WT, 41.9 ± 1.05; cKO, 44.4 ± 0.95; Fig. 1G, H), suggesting a global reduction in cortical thickness. Notably, we observed a heterotopia that contained a substantial population of CUX1-positive neurons in the anterior dorsomedial region beneath the deep layer of cKO mice (Supplementary Fig. 2B), indicative of mild defects in cortical neuronal migration.

### *Arglu1* deletion causes delocalization of progenitors

**Fig. 2:**
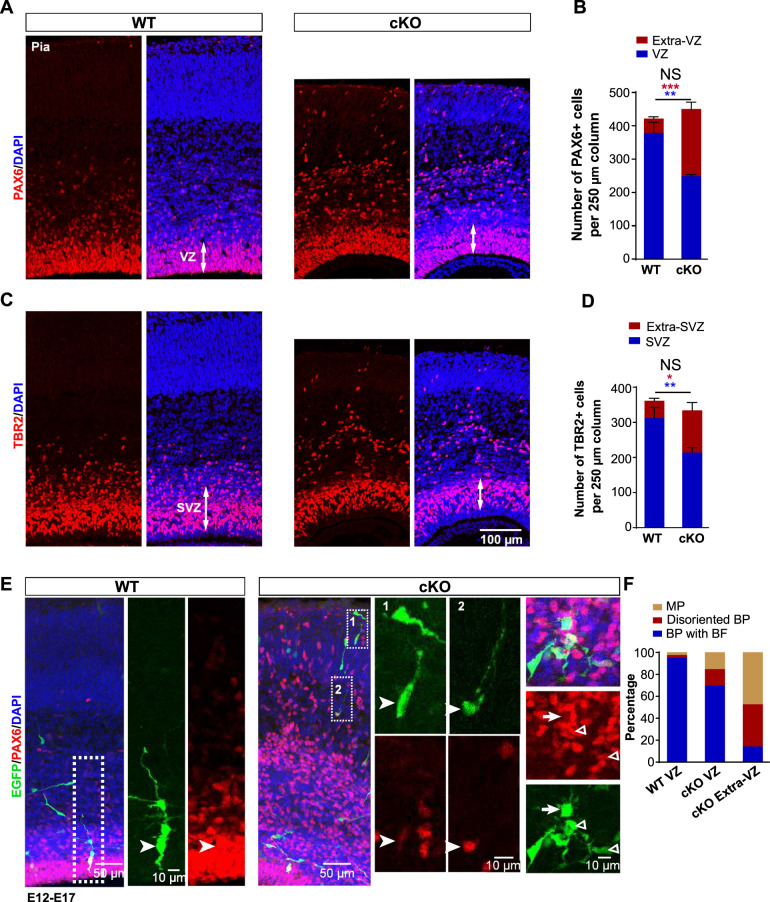
*Arglu1* deletion leads to RG delocalization and detachment from the VZ. **A**–**D** Representative images of cortices stained for PAX6 (**A**) and TBR2 (**C**) and quantification of the number of PAX6+ cells (**B**) and TBR2+ cells (**D**) in a 250 µm column from E16.5 WT and cKO mice (WT, *n* = 8; cKO, *n* = 9 mouse brains). Asterisks indicate significant differences of the number of total PAX6+ or TBR2+ cells (black), in the ventricular zone (VZ, blue), and in the extra-VZ (red) between WT and cKO mice. *P < 0.05; **P < 0.01; ***P < 0.001, two-tailed Student *t* test; NS not significant. Data are represented as mean ± SEM. **E** Representative images of EGFP-expressing cells stained for PAX6 in E17 cortices. Boxed regions are expanded into right panels corresponding to numeric digits. Arrow heads indicate EGFP+ RGs expressing PAX6. Arrow denotes a multipolar RG. Triangles indicate disoriented bipolar RGs. **F** Quantification of the percentage of EGFP + PAX6+ cells that were multipolar (MP), disoriented bipolar cells (BP) or bipolar with basal process (BP) (WT: VZ, *n* = 80 cells from four mouse brains; cKO: VZ, *n* = 66 cells from four mouse brains; Extra-VZ, *n* = 104 cells from four mouse brains). Note that none of delocalized RGs is an oRG-like cell in morphology.

To determine the possible causes of the microcephaly, we examined two major populations of progenitor cells that give rise to all cortical excitatory neurons in the embryonic mouse cortex: PAX6-expressing RGs residing in the VZ and TBR2-expressing IPs residing in the SVZ [4]. We observed a large number of progenitor cells that were ectopically located in the cortical plate and the intermediate zone of *Arglu1* cKO mice at E16.5. While the total number of PAX6-expressing RGs in a 250 µm column of *Arglu1* cKO mice was similar to those of WT controls, we observed a significant reduction in the VZ, but a discernible increase in outside VZ regions of cKO mice in contrast to WT controls (Fig. 2A, B). Consistent with results from PAX6 staining, we also observed a number of brain lipid-binding protein (BLBP, a bona fide RG marker)-positive cells localized in outside the VZ of cKO cortices, confirming that RGs were delocalized (Supplementary Fig. 4). Likewise, numerous TBR2-expression IPs were displaced to outside SVZ regions of cKO mouse cortices at E16.5 (Fig. 2C, D).

We next examined the morphology of these ectopic RGs in cKO mice because a small number of the oRG-like progenitor cells were previously reported to exist in outside the VZ of the developing mouse cortex [8, 18]. A typical oRG lacks the apical process anchoring to the lateral ventricular membrane, but retains a basal fiber to the pia. Retroviruses expressing enhanced GFP (EGFP) were injected into the lateral ventricle of embryonic mouse brains at E12, which is capable of infecting dividing RGs in the VZ and labeling them and their progeny with EGFP. RG morphology was recovered by immunostaining of E17 brain sections with antibodies against PAX6 and GFP. As we expected, most of RGs in the VZ of cKO cortices exhibited a typical bipolar morphology consisting of a basal process and an apical process, resembling RGs of the WT control (Fig. 2E). In contrast, ~50% of delocalized RGs in cKO cortices were multipolar with more than two short processes emanating from their soma; ~35% displayed an oblique or horizontal orientation with one or two short processes; and only ~15% showed a characteristic unipolar morphology with the basal process extending to the pia (Fig. 2E, F). BLBP staining revealed similar morphological RGs in outside the VZ of cKO cortices (Supplementary Fig. 4). These results together suggest that a large portion of delocalized RGs do not possess a prominent basal process, a typical morphology of an oRG, and therefore they are unlikely oRGs.

### Loss of *Arglu1* impairs neurogenesis owing to delayed mitotic progression

Previous studies show that detached RGs from the VZ are very likely to undergo premature differentiation by exiting the cell cycle [19, 20], which could contribute to the microcephaly through an impairment in neurogenesis. We co-immunostained brain sections for Ki67, a proliferation marker, and PAX6 at E16.5. Similar to those of WT controls, both RGs in the VZ and ectopic RGs in the extra-VZ of cKO mice expressed Ki67 (Supplementary Fig. 5A). Ectopic RG cells were also positive for a mitotic marker the phosphorylated histone H3 (PHH3), indicating that they were proliferative and did not prematurely exit the cell cycle to undergo differentiation (Supplementary Fig. 5B, C).

**Fig. 3:**
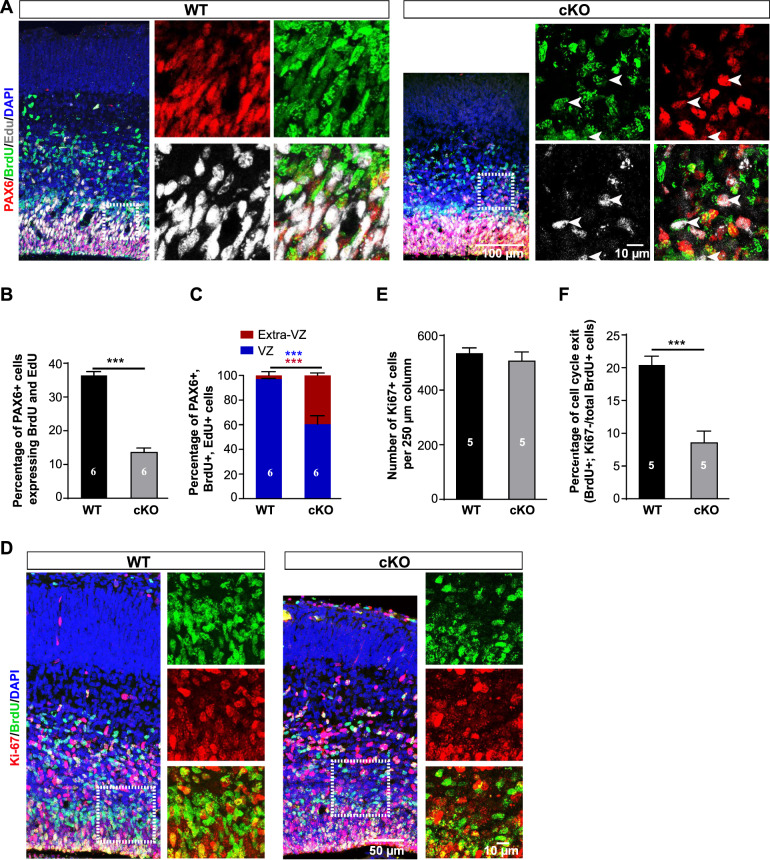
Loss of *Arglu1* impairs neurogenesis. **A**–**C** Representative images of cortices stained for PAX6, BrdU and EdU in E16 WT and cKO brain sections (**A**) and quantification of percentage of PAX+ cells expressing BrdU and EdU (**B**) and portions of triple PAX6+ BrdU+ EdU+ cells located in the VZ or the Extra-VZ (**C**) of E16 WT and cKO mouse cortices (WT, *n* = 6; cKO, *n* = 6 mouse brains). Arrows in (**A** *cKO*) indicate triple PAX6+ BrdU+ EdU+ cells. Asterisks in (**C**) indicate statistical significant differences for cell portions in the VZ (blue) and in the extra-VZ (red). BrdU was injected at E15 and a subsequent pulse of EdU was administered intraperitoneally at E16. Pregnant mice were sacrificed 1 hr after EdU injection for fixation of embryo brains. **D** Representative immunofluorescence images of E16 WT and cKO cortices stained for BrdU and Ki67. Right panels expanded from corresponding boxed regions of the left panels show the colocalization of BrdU and Ki67. **E**, **F** Quantification of the number of total Ki67+ cells (**E**) and percentage of BrdU+ cells that do not express Ki67 (**F**) (WT, *n* = 5; cKO, *n* = 5 mouse brains). Note that *Arglu1* deletion does not change the number of total Ki67+ cells, but reduces the number of newly generated postmitotic cells. ***P < 0.001, two-tailed Student *t* test. Bar graph data are represented as mean ± SEM.

We next examined whether RGs could undergo normal consecutive cell divisions by self-renewing after removal of *Arglu1*. One pulse of 5-bromo-2′-deoxyuridine (BrdU) was injected intraperitoneally into pregnant mice at E15 and another pulse of 5-ethynyl-2′-deoxyuridine (EdU) was sequentially administered at E16 with an interval of 24 h (hrs). Pregnant mice were sacrificed 1 h after EdU injection and embryo’s brains were recovered for analyses. A RG that consecutively goes through S-phase during the short period after BrdU/Edu injection should incorporate both BrdU and EdU. Quantitative analyses revealed that the percentage of PAX6+ cells expressing BrdU and EdU was significantly lower in the cKO cortex than that of the WT control (Fig. 3A, B), indicating reduced capacity of self-renewing for RGs. Ectopic PAX6+ BrdU+ and EdU+ cells accounted for more than 40% of total PAX6+ BrdU+ and EdU+ cells, contrasting to less than 5% of them in the extra-VZ of WT controls (Fig. 3C).

Given that the total number of PAX6+ cells in a 250 µm column were not altered after removal of *Arglu1* (Fig. 2B), we inferred that a reduction in the number of RGs expressing BrdU and EdU resulted from a delay in the mitotic progression. We next quantified Ki67+ cells, BrdU+ cells, and BrdU+ Ki67− cells at E16.5. In support of this hypothesis, we observed no change in the number of Ki67+ cells, but a significant decrease in the fraction of BrdU+ Ki67− cells out of total BrdU+ cells (Fig. 3D–F), suggesting a prolonged cell cycle for RGs in cKO mice.

### *Arglu1* deletion results in apoptosis

**Fig. 4:**
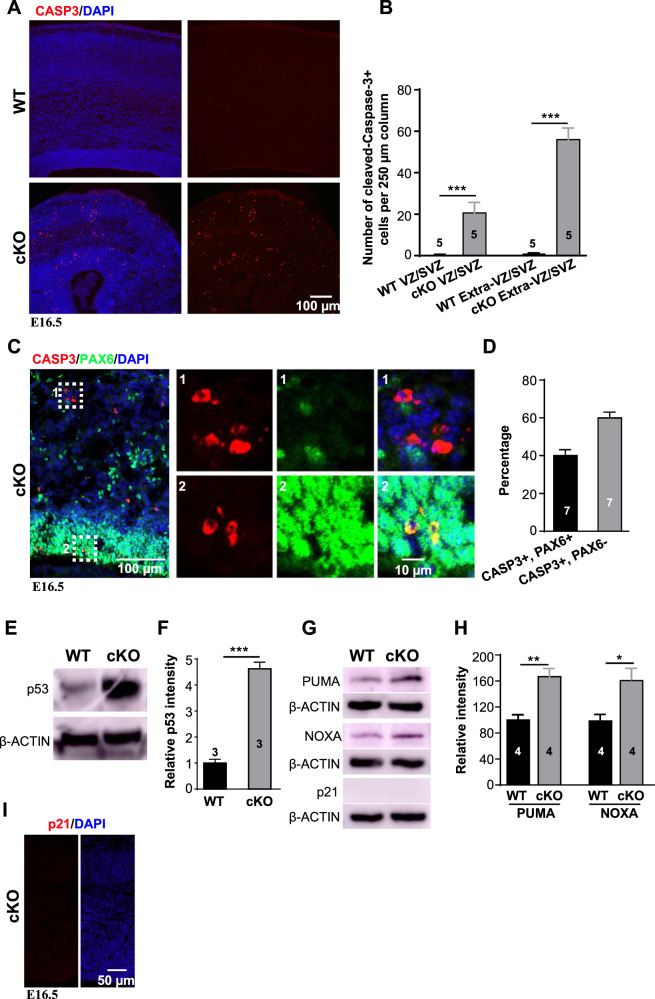
Loss of *Arglu1* results in cell apoptosis. **A** Representative images of E16.5 WT and cKO cortices stained with antibody against cleaved Caspase-3 (CASP3). **B** Quantification of the number of CASP3+ cells located in the VZ and SVZ zones and Extra-VZ/SVZ region of a 250 µm cortical column (WT, *n* = 5; cKO, *n* = 5 mouse brains). Results show a significant increase in the number of CASP3+ cells in cKO mice compared with that in WT controls. **C**, **D** Representative images and quantification show immunostaining of WT and cKO mouse cortices for CASP3 and PAX6 at E16.5 (WT, *n* = 7; cKO, *n* = 7 mouse brains). Right panels enlarged from the corresponding boxed regions in the left indicate CASP3 + PAX6- cells (*1*) and CASP3 + PAX6+ cells (*2*), respectively. **E**–**H** Sample images of immunoblots of P0.5 cortical homogenates with antibodies against p53 (**E**), PUMA, NOXA and p21 (**G**) and quantification of relative p53 (**F**) and PUMA and NOXA intensity (**H**) in WT and cKO mice (p53: WT, *n* = 3; cKO, *n* = 3 mouse brains; PUMA and NOXA: WT, *n* = 4; cKO, *n* = 4 mouse brains). **I** Representative images show immunostaining of E16.5 cKO mouse cortices for p21. Note that p21 staining is not observed in cKO cortices. *P < 0.05; **P < 0.001; ***P < 0.001. *P* values were determined by two-tailed Student *t* test. Bar graph data are represented as mean ± SEM.

Aside from defects in cellular proliferation, microcephaly could arise from cell apoptosis. We next examined cell apoptosis in *Arglu1* cKO mice. Immunohistochemistry revealed a significant increase in the number of cleaved CASP3+ cells regardless of their localization in cKO cortices compared with those of WT controls (Fig. 4A, B). PAX6-expressing cells accounted for ~40% of CASP3+ cells in E16.5 cKO cortices (Fig. 4C, D). Moreover, we detected significantly upregulated protein levels of p53 and two p53 downstream target genes (the BH3-only proteins PUMA and NOXA), which are required for induction of apoptosis [21–23], but not p21, another p53 target gene whose expression induces the G1/S cell cycle arrest [24], in the cortices of cKO mice in contrast to those of WT controls at E16.5 and P0.5 (Fig. 4E–I). To exclude the possibility of the off-target activation of p53 by Cre-induced loxp recombination [25–27], we crossed *Emx1*^*Cre/+*^-*Arglu1*^*fl/+*^ mice with *COUP-TFI*^*fl/fl*^ mice to generate *Emx1*^*Cre/+*^-*Arglu1*^*fl/+*^; *COUPTF* ^*fl/+*^mice that contain the same amount of loxP sites as *Arglu1* cKO mice. Neither CASP3+ cells nor expression of p53 were observed in *Emx1*^*Cre/+*^-*Arglu1*^*fl/+*^ or *Emx1*^*Cre/+*^-*Arglu1*^*fl/+*^; *COUPTF* ^*fl/+*^ mouse cortices (Supplementary Fig. 6A, B), suggesting that removal of *Arglu1* indeed caused cell apoptosis, a possible cause that leads to microcephaly.

### Loss of *Arglu1* drastically alters alternative mRNA splicing in vivo

ARGLU1 has been reported to regulate mRNA splicing and gene transcription in vitro [12, 15]. To pinpoint molecular mechanisms that underlay the defects in cell survival and proliferation in vivo, we crossed Emx1-Cre mice with *Arglu1*^*fl/fl*^*:Rosa26-tdTomato* mice to induce tdTOMATO expression in ARGLU1-deficient excitatory neurons. The tdTOMATO-expressing neurons were isolated by fluorescence-activated cell sorting (FACS) from P0.5 cerebral cortices of WT and cKO mice for RNA-seq. Both cluster analysis and principle component analysis (PCA) demonstrated a strong correlation coefficient within groups but not between WT and cKO mice (Supplementary Fig. 7A, B), indicating that these RNA-seq data were of high quality. Compared with WT controls, we found 957 differentially expressed genes (DEGs) in neurons of cKO mice, including 340 genes downregulated, such as *Arglu1*, and 617 genes upregulated (Supplementary Fig. 7C and Supplementary Tables 1 and 2). Gene ontology (GO) analysis of DEGs revealed that downregulated rather than upregulated DEGs were obviously enriched in regulating neuronal functions (Supplementary Fig. 7D).

**Fig. 5:**
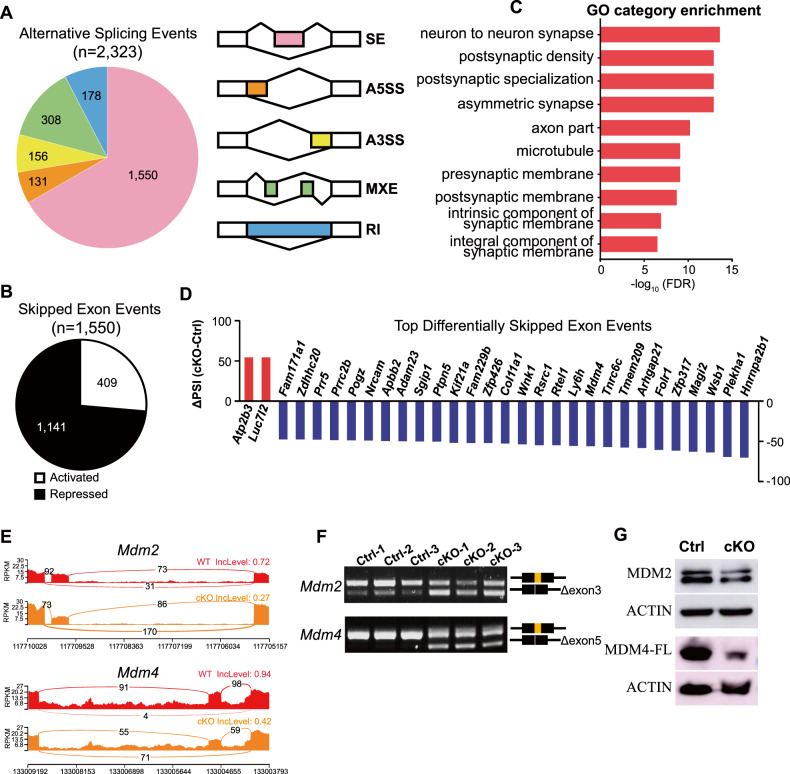
ARGLU1 regulates alternative splicing in developing mouse cortex in vivo. **A** Pie chart shows the classification of 2323 significant alternative splicing events (FDR < 0.05 and absolute ΔPSI (percentage of spliced-in) >15) identified by RNA-seq. The right panel illustrates the types of splicing events. Skipped exon (SE), alternative 5′ splice site (A5SS), alternative 3′ splice site (A3SS), mutually exclusive exons (MXE), and retained intron (RI) events were analyzed. **B** Ontology analyses of alternatively spliced genes show enriched neuronal functions. **C** A total of 1550 skipped exon events are altered in treated (see Supplementary Table 3). A total of 1141 events were repressed and 409 were activated. **D** The top list of the 30 AS exon events ranked highly according to the absolute ΔPSI. Activated events are in red. Repressed events are in blue. **E** Representative sashimi plots from bulk analyses showing alternatively spliced exon 3 of *Mdm2* and exon 5 of *Mdm4*. **F** RT-PCR validation of *Mdm2* and *Mdm4*. **G** Sample images of immunoblots of P0.5 cortical homogenates with antibodies against MDM2 or MDM4 (Replicates: WT, *n* = 3; cKO, *n* = 3 mouse brains). Note that the MDM2 and full-length MDM4 are evidently reduced in the mouse cortices after loss of *Arglu1*.

We next performed analysis of RNA-seq data for changes in alternative splicing (AS) events. Differential AS events were defined based on assessments of the percentage of spliced-in (∆PSI, >15%) and false discovery rate (FDR < 0.05). Consistent with previous findings from *Arglu1* shRNA experiments in vitro [12], we found that removal of *Arglu1* resulted in 2323 differential AS events in vivo (Fig. 5A and Supplementary Table 3). Skipped exon (SE) events (1550) comprised the largest portion (~66.7%) of AS events, including 1141 exon repressed and 409 exon-activated events (Fig. 5B). Similar to downregulated DEGs, we found alternatively spliced genes enriched in neuronal functions related with synapse formation and transmission (Fig. 5C). Several top differential SE events including *Hnrnpa2b1*, *Zfp317*, *Tmem209* and *Mdm4* from the top 30 list and *Mdm2* were validated by RT-PCR (Fig. 5D–F and Supplementary Fig. 8A, B), demonstrating a high correlation of the exon skipping-like incidence and the ∆PSI values from RNA-seq data. Of the top alternatively spliced genes, we focused on *Mdm2* and *Mdm4* because they have been reported to synergistically inhibit p53 activity in cortical progenitor cells [28, 29]. RNA-seq analysis of alternative splicing revealed skipping of *Mdm2* exon 3, which encodes a domain necessary for p53 binding, and skipping of *Mdm4* exon 5. For MDM2, exon 3 skipping resulted in a short isoform that lacks a p53-binding domain [30]. For MDM4, skipping of exon 5 that consists of 68 nucleotides led to a NMD due to a frameshift. In line with this, the levels of the MDM2 and full-length MDM4 protein were significantly reduced in the cortices of cKO mice compared to those of WT controls (Fig. 5G), thereby leading to accumulation of p53 through relieving MDM2/4-mediated p53 degradation.

To verify the roles of ARGLU1 in development and regulation of AS in neurons, we performed rescue experiments by transfection of *Arglu1* KO Neuro-2a (N2a) cells, a mouse neuroblastoma cell line, with plasmids containing the full-length *Arglu1* cDNA. Although proliferation was no altered in *Arglu1* KO N2a cells (Supplementary Fig. 9A, B), cell apoptosis was heavily observed in these cells and could be largely rescued by re-expression of ARGLU1 (Supplementary Fig. 9C–E). Moreover, we found that *Tmem209* SE in the rescue group (re-expression of ARGLU1 in KO N2a cells) was comparable to WT controls (Supplementary Fig. 8C). Both *Mdm2* and *Mdm4* SE were largely rescued and *Hnrnpa2b1* AS was at least partially rescued (Supplementary Fig. 8D–F), confirming the role of ARGLU1 in regulation of alternative splicing. The partial rescue was very likely due to technical limitations in the efficiency of cell transfection with plasmids.

### Microcephaly is largely rescued by removal of p53

**Fig. 6:**
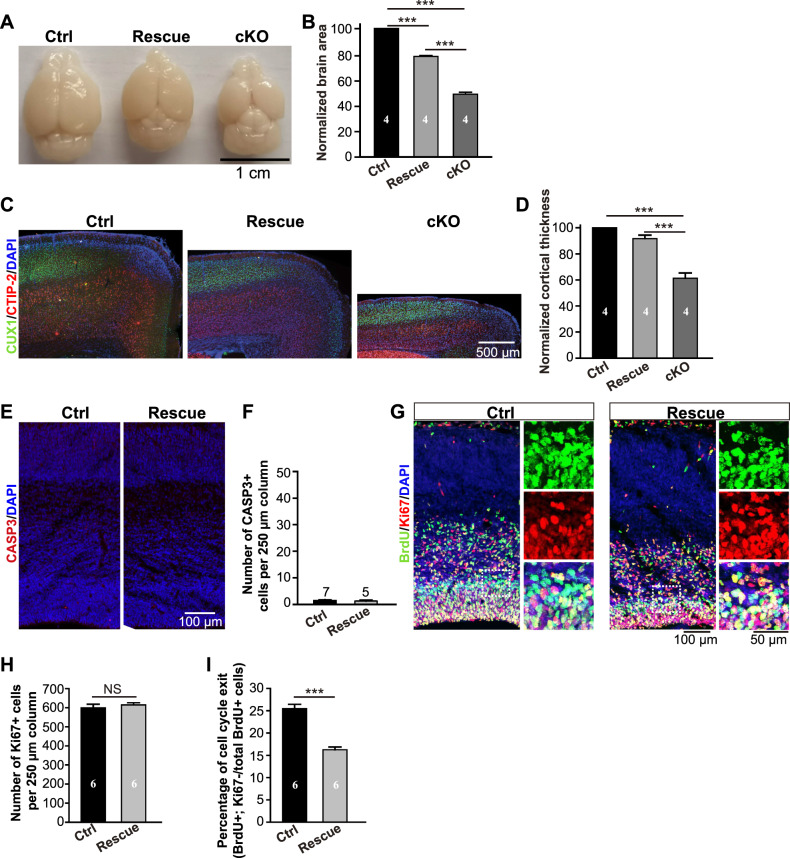
Removal of p53 prevents cell apoptosis and largely rescues the microcephaly caused by deletion of *Arglu1*. **A**, **B** Representative whole-mount images and quantification of the telencephalic area of brains from controls (Arglu1^fl/fl^; p53^−/−^ and/or Arglu1^fl/+^; p53^−/−^), rescues (Emx1-Cre-Arglu1^fl/fl^; p53^−/−^) and cKO mice (Emx1-Cre-Arglu1^fl/fl^) at P21 (Ctrl, *n* = 4; Rescue, *n* = 4; cKO, *n* = 4 mouse brains; ***P < 0.001, One-way ANOVA with posthoc Bonferroni’s test). **C**, **D** Representative images of cortices stained for DAPI, CUX1 and CTIP-2 (**C**) and quantification of cortical thickness (**D**) for Ctrl, Rescue and cKO mice at P21 (Ctrl, *n* = 4; Rescue, *n* = 4; cKO, *n* = 4 mouse brains; ***P < 0.001, One-way ANOVA with *post* *hoc* Bonferroni’s test). **E**, **F** Sample confocal images of P0.5 cortices stained with antibody against cleaved CASP3 for Ctrl and rescue mice and their corresponding quantification of the number of cleaved caspase-3 positive cells per 250 µm cortical column (WT, *n* = 7; cKO, *n* = 5 mouse brains). **G** Representative immunofluorescence images of E16 Ctrl and rescue cortices stained for BrdU and Ki67. Right panels expanded from boxed regions of the left panels show the colocalization of BrdU and Ki67. **H**, **I** Quantification of the number of total Ki67+ cells (**H**) and percentage of BrdU+ cells that do not express Ki67 (**I**) (WT, *n* = 6; cKO, *n* = 6 mouse brains). ***P < 0.001, two-tailed Student *t* test. Bar graph data are represented as mean ± SEM.

It is well known that MDM2 and MDM4 cooperate to inhibit activity of p53 [28, 29, 31]. To test whether upregulation of p53 led to the cell death and microcephaly, *Arglu1* cKO mice (*Emx1*^*Cre/+*^-*Arglu1*^*fl/fl*^) were crossed with the whole-body *p53* KO mice (*p53*^*−/−*^) to generate the *Emx1*^*Cre/+*^-*Arglu1*^*fl/fl*^*;p53*^*−/−*^ mice in which *p53* and *Arglu1* were simultaneously removed. We found that both the telencephalic area and the cortical thickness were largely rescued by removal of p53 (Fig. 6A–D) and no obvious apoptosis was detected in neither the cortices of rescue mice (*Emx1*^*Cre/+*^-*Arglu1*^*fl/fl*^*;p53*^*−/−*^ mice) nor the cortices of control mice (*Arglu1*^*fl/fl*^*;p53*^*−/−*^ *or Arglu1*^*fl/+*^*;p53*^*−/−*^ mice) (Fig. 6E, F). Moreover, the number of Ki67+ cells in a 250 µm-wide column of the cortex were similar between control (*Arglu1*^*fl/fl*^*;p53*^−*/−*^ *or Arglu1*^*fl/+*^*;p53*^*−/−*^ mice) and rescue mice (*Emx1*^*Cre/+*^-*Arglu1*^*fl/fl*^*;p53*^*−/−*^ mice). The fraction of BrdU+ Ki67− cells out of total BrdU+ cells was significantly reduced in rescue mice than that of control mice (Fig. 6G–I) and no p21 expression was observed in the cortices of *Arglu1* cKO mice (Fig. 4G, I), implicating that the prolonged cell cycle is not through p53 activation. Together, these results suggest that p53 accumulation leads to apoptosis and accounts for most of the microcephaly caused by deletion of *Arglu1*.

## Discussion

Whole-body knockout of *Arglu1* causes mouse embryos to cease development at E9.5 [12], demonstrating that ARGLU1 is critical for normal embryonic development. In this study, we employed conditional null mice of *Arglu1* induced by Emx1-Cre recombination, demonstrating important roles of ARGLU1 in progenitor proliferation and cortical neurogenesis. Microcephaly was evidently observed for *Arglu1* cKO mice at both developmental and mature stages. Further mechanistic studies showed: (1) that progenitors are massively delocalized; (2) that although delocalized RGs remain proliferative, cell proliferation regardless of progenitor localization is significantly reduced, likely resulting from a delay in cell cycle progression; (3) that deletion of *Arglu1* causes alternative splicing of *Mdm2* and *Mdm4*, two essential negative regulators of p53 activity, thereby leading to stabilization of p53 and cell apoptosis; and (4) removal of p53 completely prevents cell apoptosis and largely recues the microcephaly caused by deletion of *Arglu1*. Therefore, these results suggest that cell apoptosis, together with reduced proliferation, contribute to the microcephaly.

Microcephaly associated with loss of splicing factors has been reported in human patients [32, 33] and mouse models [33–37]. Cellular mechanistic studies show that this microcephaly results from either depletion of the progenitor pool by DNA damage-induced cell death [33, 34], diminished neuron production caused by chromosome separation inhibition-induced mitotic delay [35] or Bak1-induced apoptosis [36]. ARGLU1 has been characterized as an evolutionarily conserved AS regulator through directly interacting with conventional splicing factors [12]. By comparison, we observed no significant changes in the density of progenitor number in *Arglu1* cKO mice. Likewise, cortical organization and layer lamination are grossly unaffected after deletion of *Arglu1*, unlike that depletion of splicing regulators NOVA2 [38] or RBFOX2 [39] causes defects in neuronal migration. Instead, we found increased apoptosis and a mitotic delay, both of which contributed to a reduction in neuron production, eventually leading to the microcephaly. In contrast to conditional knockout mice (*Emx1-Ptbp2*^*−/−*^) showing massive neuronal death in the neonatal cortex [40], cell apoptosis mostly occurs embryonically in the cortex of *Arglu1* cKO mice because cortical area is not further reduced after birth. Recently, *Arglu1* was identified as a potential diagnostic gene in amyotrophic lateral sclerosis (ALS) [41], suggesting that ARGLU1 might serve as an anti-apoptotic factor in the adult central nervous system in the context of neurodegenerative diseases.

Our results suggest that cell apoptosis in cKO mice appears to be induced by p53 accumulation. Removal of *Arglu1* led to a short isoform MDM2 lacking the p53-binding domain and the NMD of MDM4 owing to skipping of the exon 3 and exon 5, respectively. Since MDM2 and MDM4 are essential for inhibition of p53 activity [28, 29, 42], changes in AS splicing of *Mdm2* and *Mdm4* resulted in p53 stabilization and activation. In support of this, the protein levels of the full-length MDM2 and MDM4 were significantly diminished in the cortices of *Arglu1* cKO mice. Furthermore, removal of p53 rescued the microcephaly associated with cell apoptosis but not with defects in mitotic progression. In support of this, the protein levels of two p53-related apoptotic factors PUMA and NOXA, but not the cell cycle arrest factor p21, are increased, in *Arglu1* cKO mouse cortices. These results together suggest the second checkpoint mechanism other than a p53-dependent one underlies delayed cell cycle progression [43–45]. Our finding is consistent with a previous report that removal of p53 partially rescued the microcephaly caused by depletion of *Prmt5*, a gene that encodes a protein arginine methyltransferase. In *Prmt5-null* mice, *Mdm4* undergoes skipping of exon 7 due to malfunction of constitutive splicing machinery induced by reduced methylation of the splicing Sm proteins, thereby leading to an unstable short isoform of MDM4 [37].

Several lines of evidence show that *Mdm2* exon 3 and *Mdm4* exon 7 in mice are alternatively spliced under pathological conditions in vivo and in vitro [37, 46, 47], but aberrant skipping of *Mdm4* exon 5 is previously unreported. Although ARGLU1 has been shown to directly interact with conventional splicing factors such as PUF60 and U2AF2, a further mechanistic study on the involvement of ARGLU1 in mRNA alternative splicing needs to be addressed in the future.

Neurogenesis in the embryonic mouse cortex begins at around E11 and Emx1-induced recombination occurs about one day ahead (E10) [48, 49]. Prior to E11, neural epithelial cells expand the progenitor pool by way of symmetric divisions. In *Emx1-Arglu1*^*−/−*^ mice, we might have only observed the effects of ARGLU1 on neurogenic behaviors of RGs and neuronal migration. Since robust ARGLU1 expression is observed in the neural epithelial stem cells before they transform into neural radial glial cells that directly produce neurons, whether ARGLU1 affects stem cell behaviors of neural epithelial cells such as proliferation, transformation and survival remains an open question. Further studies are needed to elucidate important roles of ARGLU1 in these early developmental processes. Given that ARGLU1 has been reported as a regulator of glucocorticoid signaling, which can be activated by binding of stress hormones to glucocorticoid receptor, in neural cells [12], it would be also interesting to test whether ARGLU1 plays an important role in fetal brain developmental abnormalities induced by maternal prenatal stress [50, 51].

## Materials and methods

### Mouse strains

*Arglu1*^*fl/+*^ C57BL/6 mice were generated by GemPharmatech using the CRISPR-Cas9 system. Cas9, two guide RNAs (sgRNA) and donor vector were together microinjected into fertilized eggs of C57BL/6J mice.

gRNA 1 sequence: 5′ CACTAGACACTGCTCCAACG 3′;

sgRNA2 sequence: 5′ GGATATAATTGACCTGATTG 3′

Two loxps were inserted into intron 1 and intron 3 by sgRNAs directed cas9 endonuclease cleavage and homologous recombination, respectively, resulting in flanking exons 2 and 3. The F0 generation containing two loxps was confirmed by PCR and sequencing. The F1 generation stable line was obtained by mating with C57BL/6 mice. To generate the conditional knockout line, *Arglu1*^*fl/+*^ C57BL/6 mice were crossed with the *Emx1-Cre* knock-in C57BL/6 mouse line (a kind gift from Dr. Ke Tang) to generate *Emx1-Cre;Arglu1*^*fl/fl*^ mice. *Emx1-Cre;Arglu1*^*fl/fl*^*;Rosa26-Tdtomato*^*fl/−*^ mice were generated by the same mating strategy by crossing *Emx1-Cre;Arglu1*^*fl/+*^ and *Rosa-Tdtomato*^*fl/+*^ mice line (a kind gift from Dr. Bin Zhou, Shanghai Institute of Biochemistry and Cell Biology, CAS, China). *Emx1-Cre;Arglu1*^*fl/fl*^ mice were crossed with the whole-body *p53* knockout mice (p53^−/−^ mice purchased from GemPharmatech) to generate *Emx1-Cre;Arglu1*^*fl/fl*^; p53^−/−^ mice. *Emx1-Cre;Arglu1*^*fl/+*^ mice were crossed with the COUP-TFI^*fl/fl*^ mice (a kind gift from Dr. Ke Tang) to generate *Emx1-Cre;Arglu1*^*fl/+*^; COUP-TFI^*fl/+*^ mice. Genotyping primers were shown as follows: *Arglu1*: *Arglu1*-F (ACGTGGACAGCATAGGATGAAATG)

*Arglu1*-R (TCATCTTCCTATCAACTGACCCTGG);

*Emx1-Cre*: *Cre*-F (GATCTCCGGTATTGAAACTCCAGC)

*Cre*-R (GCTAAACATGCTTCATCGTCGG);

*Tdtomato*: *td*-WT-F(AAGGGAGCTGCAGTGGAGTA),

*td*-WT-R(CCGAAAATCTGTGGGAAGTC),

*td*-Mut-F(CTGTTCCTGTACGGCATGG),

*td*-Mut-R(GGCATTAAAGCAGCGTATCC).

*P53*: *p53*-WT-F(CAGAAGTCACAGCACATGACG),

*P53*-WT-R(CTCCCAGAGACTGCTGTTAAAGTAG),

*P53*-Mut-F(GCTAGAAGTACCTCCCTGATTACCTG),

*P53*-Mut-R(TAGGGTAGGAACCAAAGAGCGTTG).

All mice were maintained under standard housing conditions of 22 ± 1 °C, 50 ± 10% relative humidity and a 12 h light-dark cycle with food and water. Male and female mice were randomly allocated into experimental groups. Animal protocols were approved by the Institutional Animal Care and Use Committees of ShanghaiTech University, China.

### Plasmid construction

For construction of sgRNAs, oligos were synthesized, annealed and cloned into BsaI site of the sgRNA expression vector pGL3-U6-sgRNA-EGFP. To generate *Arglu1* KO cell lines, two sgRNAs were used (sgArglu1-1:TAGTGGATCTGTTTGTGCTA; sgArglu1-2:TTGAGCGAGAAGTTCTCCGA). The full-length CDS of *Arglu1* was cloned from the cDNA library of the neonatal mouse neocortex by using the primers: *Arglu1-EGFP*-F (AATTCGCTAGCGGATCCGCCACCATGGGCCGGTCGCGGAGC) and *Arglu1-EGFP*-R(GGGGGGGAGGGAGAGGGGTCAATCCTGGGTTTTTAGTGAGAAGGA), *IRES* was amplified by using the primers: *IRES*-F (CCCCTCTCCCTCCCCCCC) and *IRES*-R (CATGGTGGCGACCGGTTTATCATCGTGTTTTTCAAAGGAAAACCACG), the *Arglu1-IRES* fragment was generated via the overlap PCR, then subcloned into pCAG-EGFP vector at the two restriction enzyme site BamHI and AgeI using In-Fusion cloning (Clontech).

### Cell culture and transfection

N2a cells was were maintained in the DMEM medium supplemented with 10% (v/v) FBS, 1% penicillin–streptomycin and 1% L-glutamine at 37 °C in humidified air containing 5% CO_2_. Cells were seeded on 12-well plates one day before transfection. For generating *Arglu1 knockout* cell line, the SpCas9 expressing plasmid (1 µg) and two sgRNA-expressing plasmids (0.5 µg + 0.5 µg) were cotransfected using Lipofectamine 2000 (Life Technologies) according to the manufacturer’s protocol. 72 h after transfection, the cells were harvested for flow sorting to generate single-cell clones. No mycoplasma contamination was detected for all N2a cell cultures. For rescuing experiments, cotransfection of the plasmid pCAG-Arglu1-IRES-EGFP with pGL3-PGK-puromycin into *Arglu1-KO* cells was also performed by using Lipofectamine 2000. After 48 h, medium was replaced with a medium containing 2 µg/ml puromycin to eliminate the negative cells. 48 h after puromycin selection, the cells were collected for RT-PCR experiments.

### Flow cytometry assay

Neonatal mice (P0.5) of *Emx1-cre;Rosa26-Tdtomato*^*fl/+*^ (WT controls) and *Emx1-cre;Arglu1*^*fl/fl*^*;Rosa26-Tdtomato*^*fl/+*^ (cKO pups) were anesthetized on ice; then brains were removed; neocortices were isolated, cut into tiny pieces in ice-cold EBSS as small as possible, digested by papain solution containing DNase at 37 °C for 30 min, then harvested and subjected to the flow cytometry (FACS AriaIII) for single tdTOMATO-positive cell sorting.

### In utero retrovirus injection

Timed pregnant ICR mice were used for *in utero* retrovirus injection and electroporation experiments as previously described [52]. Mice at embryonic day (E) 12 were anesthetized with isoflurane and positioned on the heating pad for surgery. In all, 1 μL EGFP-expressing retroviral solution (~10^9^ FU/ml) mixed with fast green (Sigma) was injected into the lateral ventricle of the embryos with a sharpened glass micropipette (Drummond Scientific). After completion of virus injection, the uterus was replaced back into the abdominal cavity and the wound was sutured. The surgery mice were placed into the warm ventilation chamber until they were recovered for showing physiological movements.

### RNA extraction and RT-PCR

Total RNA was extracted from cultured cells and the cerebral cortical tissues by using the TRIzol reagent (ThermoFisher, 15596026), and cDNA was reverse-transcribed by using the SuperScript III First-strand cDNA synthesis kit (ThermoFisher, 18080051). RT-PCR was performed using 2 × Taq Master Mix (Dye Plus) (Vazyme Biotech, P112-01). The following primers were used:

*Hnrnpa2b1:* (5′-GGAACTATGGTCCTGGAGGAAG-3′, 5′-AGTTCTGTGCAAAACTAGTC-3′);

*Mdm2*: (5′-ATGTGCAATACCAACATGTCTG-3′, 5′-CTTGCTGACTTACAGCCACT-3′);

*Mdm4:* 5′-GTAATGCACTATCTAGGCCAGT-3′, 5′-GATTGGAGTATTCTGTTGCACC-3′).

*Tmem209:* (5′-ATGCAAGGAGACGTAAGC-3′, 5′-CTTATTGTAGCCACTGACAGG-3′);

*Zfp317-F:* (5′-GTTATCAGGTTGGCAAACCCC-3′, 5′-AGTGTGAACGCGCATGTGTA-3′).

### RNA-seq and data analysis

The RNA samples were deeply (>20 million reads per sample) sequenced by an Illumina HiSeq X Ten (2 × 150 bp) at the Novogene Bioinformatics Institute (Beijing, China). For differential expression analysis, transcript abundance was quantified and analyzed using Salmon (v0.14.0) and DESeq2 (v1.22.2). Genes with p-value < 0.05 and fold change (log2 scale) ≥1 or ≤ −1 were considered as significant difference. Gene Ontology (GO) was analyzed using the cluster Profiler R package (v3.18.0). For splicing analysis, the clean data was first mapped to the mouse reference genome (version: mm10) by STAR software (Version 2.5.1) with annotation from GENCODE version vM21, then transcriptome-wide splicing events were identified by rMATS (v4.0.2). To discover differential splicing event, we set the threshold parameters at |ΔPSI | ≥ 0.15 and false discovery rate (FDR) < 0.05.

### Immunohistochemistry and confocal imaging

Adult pregnant mice were anesthetized with isoflurane and embryos were then removed from the pregnant mice, transcardially perfused through the left ventricle with cold PBS followed by 4% paraformaldehyde (PFA) fixation, followed by 4% PFA at 4 °C for overnight. After post-fixation, mouse brains were embedded in 2% low melting agarose (BBI life science) and sectioned at 60 μm by using a vibrating microtome (VT1200S, Leica). For frozen sections, specimens were dehydrated in PBS containing 20% sucrose at 4 °C for overnight and then embedded in OCT compound, frozen, and sectioned into 25 μm thickness using a freezing microtome (Leica CM1950).

For immunohistochemistry, brain slices were permeabilized with 0.4% Triton X-100 in PBS (PBST) for 15 min at room temperature, washed with PBS and then blocked with blocking buffer for 1 h at room temperature, followed by incubation with primary antibodies for overnight at 4 °C, washed in PBST by three times (10 min each), the primary antibodies used in this study were as follows: rabbit anti-ARGLU1 (1:500; HPA056792, Sigma-Aldrich), rat anti-BrdU (1:500; Accurate, OBT0030), rabbit anti- Cleaved CASP3 (1:200; Abcam, ab2302), rabbit anti-CUX1 (1:100; Santa Cruz, sc-13024), rat anti-CTIP-2 (1:500; Abcam, ab18465), rabbit anti-PAX6 (1:500; Biolegend, prb-278p), mouse anti-PAX6 (1:50; Santa Cruz sc-81649), rabbit anti-PHH3 (1:500; Millipore, 06-570), rabbit anti-TBR2 (1:500; Abcam, ab23345), mouse anti-p53 (1;500; Abcam ab26), rabbit anti-p21 (1:100; cell signaling, 2947T), mouse anti-Ki67 (1;200; BD Transduction Laboratories, 610968), rabbit anti-Ki67 (1:500; Abcam, ab15580), rabbit anti-BLBP (1:500; Abcam, ab32423), and chicken anti-GFP (1:500; Aves Labs, GFP-1020).

Images were obtained using a VS120T (Olympus) or Leica SP8 confocal microscopes (Leica microsystems), and further processed by ImageJ (National Institutes of Health). Brightness and contrast on entire images were linearly adjusted. All figures were composed using Illustrator software (Adobe Systems, Inc.).

### Western blot

For western blotting, mice were anesthetized, decapitated and brains were removed quickly. Crude protein was extracted by homogenizing 0.2 mg cerebral cortex in 200 µl RIPA lysis buffer containing 2 µl 1× complete protease inhibitor mixture. Lysates were centrifuged at 12,000 rpm at 4 °C for 10 min, then the supernatants were transferred to new tubes added with 1× loading buffer, and heated for 5 min at 95 °C. Protein was separated by SDS-PAGE electrophoresis and then transferred to nitrocellulose membrane at 200 mA for 2 h in 4 °C. The membrane was blocked for 2 h in 5% non-fat dry milk and then incubated with primary antibodies: rabbit anti-ARGLU1 (1:500; HPA034962, Sigma-Aldrich), rabbit anti-p21 (1:1000; cell signaling, 2947 T), rabbit anti-PUMA (1:1000; Abcam, ab9643), rabbit anti-NOXA (1:1000; Abcam, ab131088), rabbit anti-p53 (1:1000; LSBio, LS-C334397), mouse anti-p53 (1;1000; Abcam ab26), mouse anti-MDM2 (1:200; Santa Cruz sc-965), mouse anti-MDM4 (1:200; Santa Cruz sc-74468), and anti-β-actin (1:5000; Millipore), followed by incubation with HRP-conjugated secondary antibody. ECL detection kit (Bio-Rad) was used for signal detection. Data were analyzed using ImageJ (NIH)-Fuji software.

### Sequential BrdU and EdU labeling

Sequential BrdU and EdU labeling was performed as previously described [53, 54]. *Emx1-Cre::Arglu1*^*fl/fl*^ pregnant mice were intraperitoneally injected with 5-bromo-2′-deoxyuridine (BrdU) at embryonic day (E)15 (50 µg/g body weight), and 24 h later, 5-ethynyl-2′-deoxyuridine (EdU, 10 mg/kg body weight)) was administrated into the pregnant mice 1 h prior to sacrifice. For recovering BrdU and EdU, EdU staining was performed using the manufacturer’s protocol (Yefluor 647 Edu Imaging Kits, Yeasen), then followed by processing brain sections with 2 N HCl at 37 °C for 25 min to denature DNA before staining with BrdU antibody.

### Quantification of images and statistical analysis

Telencephalic area was measured by tracing the two cerebral hemispheres of a dorsally oriented whole-mount image using ImageJ. Cortical thickness was documented at the location of 1/4 pia surface away from the middle line of a coronal cortical section. Cell number in a region of a 250-µm cortical column from the pia surface to the ventricle was counted for embryonic data. All control data were collected from aged matched littermates.

Data analyses were blindly performed by experimenter. All statistical tests and data plots in this study were carried out using the GraphPad. Statistical significance was defined by *P* value less than 0.05 determined with two-sided tests. Details of statistical analyses performed for each figure are provided in figure legends. All figures were composed using Illustrator software (Adobe Systems, Inc.).

## Supplementary information

Supplementary Figures and Materials

Original WB Data File

Reproducibility checklist

## Data Availability

The high-throughput sequencing datasets and/or analysed during the current study are available in the NCBI Sequence Read Archive database repository under accession code (PRJNA866333). All other data generated or analysed during this study are included in this published article and its supplementary information files.
