# Supplementary material for: Deletion of ARGLU1 causes global defects in alternative splicing in vivo and mouse cortical malformations primarily via apoptosis
Source: Cell Death Dis. 2023 Aug 23;14(8):543. doi: 10.1038/s41419-023-06071-w (PMC10447433; doi:10.1038/s41419-023-06071-w)
Supplement: Supplementary file 1 — Supplementary Figures and Materials [file 41419_2023_6071_MOESM1_ESM.pdf]

Deletion of ARGLU1 causes global defects in alternative splicing *in vivo* and mouse cortical malformations primarily via apoptosis

Fenyong Yao[1,6], Shisheng Huang[1,2,3,6], Jiahui Liu[1,6], Chunhua Tan[1,6], Mengqi Xu[1], Dengkui Wang[1], Maoqing Huang[1], Yiyao Zhu[4], Xingxu Huang[1*], and Shuijin He[1,5*]

Supplementary Figures:

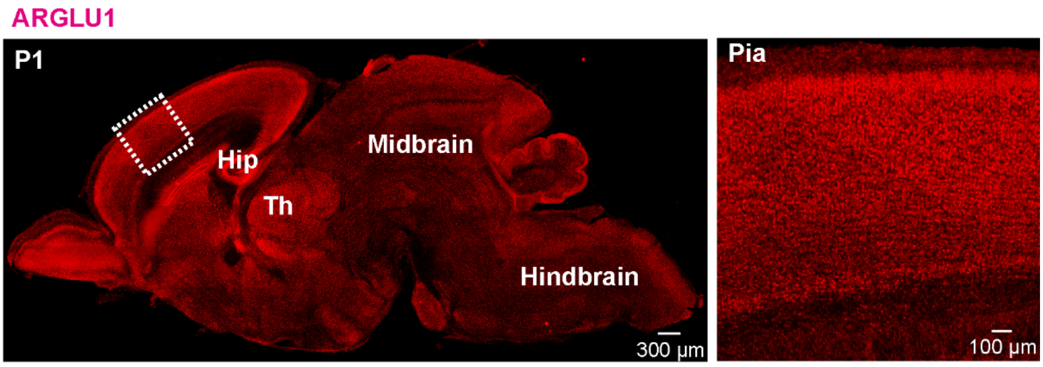

Supplementary Figure 1: ARGLU1 expression in P1 mouse cortex. Representative images of P1 cortices stained for ARGLU1 from WT mice. Right panel is expanded from the corresponding boxed region in the left. Hip, hippocampus; Th, thalamus; Pia, pia surface.

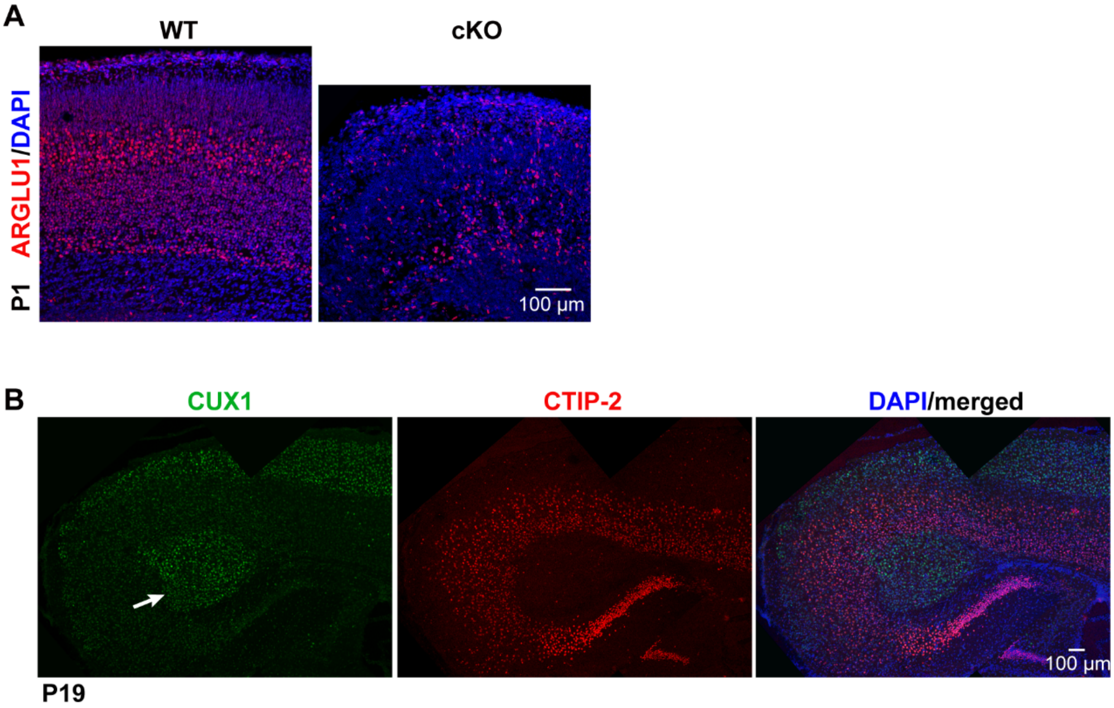

Supplementary Figure 2: Validation of *Arglu1* deletion in the cortex. (A) Representative images of P1 WT and cKO mouse cortices stained for ARGLU1. (B) Representative images showing a group of ectopic CUX1+ cells (arrow) located in the deep layer of cKO mouse cortex.

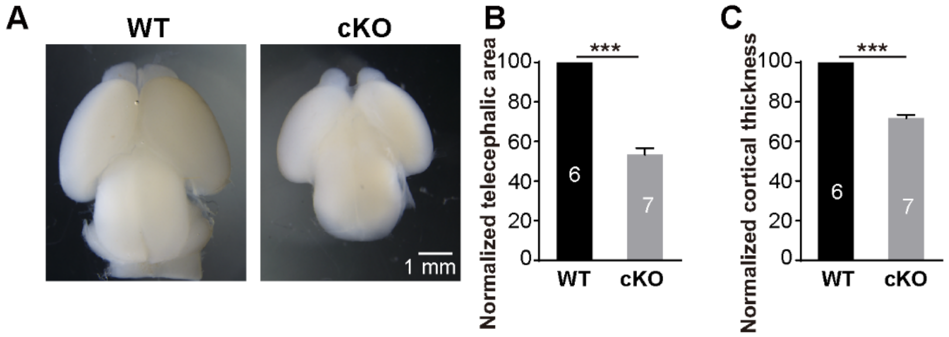

Supplementary Figure 3: *Arglu1* deletion leads to reduced brain size at birth. (A) Representative whole-mount images from WT and cKO mice at birth (P0.5). (B) Quantification of the telencephalic area of P0.5 WT and cKO brains (WT, n=6; cKO, n=7 mouse brains). (C) Quantification of cortical thickness of P0.5 WT and cKO mice (Cortex: WT, n=6; cKO, n=7 mouse brains). ***, P < 0.001, two-tailed student t-test.

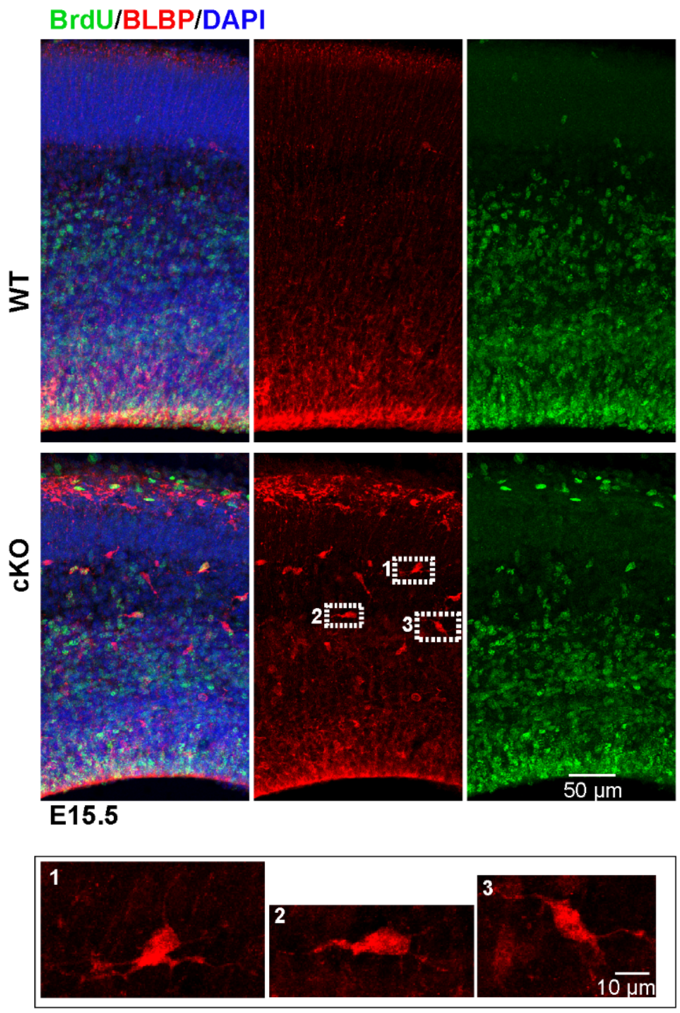

Supplementary Figure 4: Delocalized BLBP-expressing cells in the cKO cortex. Mice were intraperitoneally injected with BrdU at E14.5. E15.5 embryo cortices were recovered for immunostaining of BLBP, a bona fide RG marker, and BrdU, showing that delocalized BLBP-expressing cells are positive for BrdU. The bottom panels enlarged from corresponding regions in the top cKO cortex show multipolar RGs.

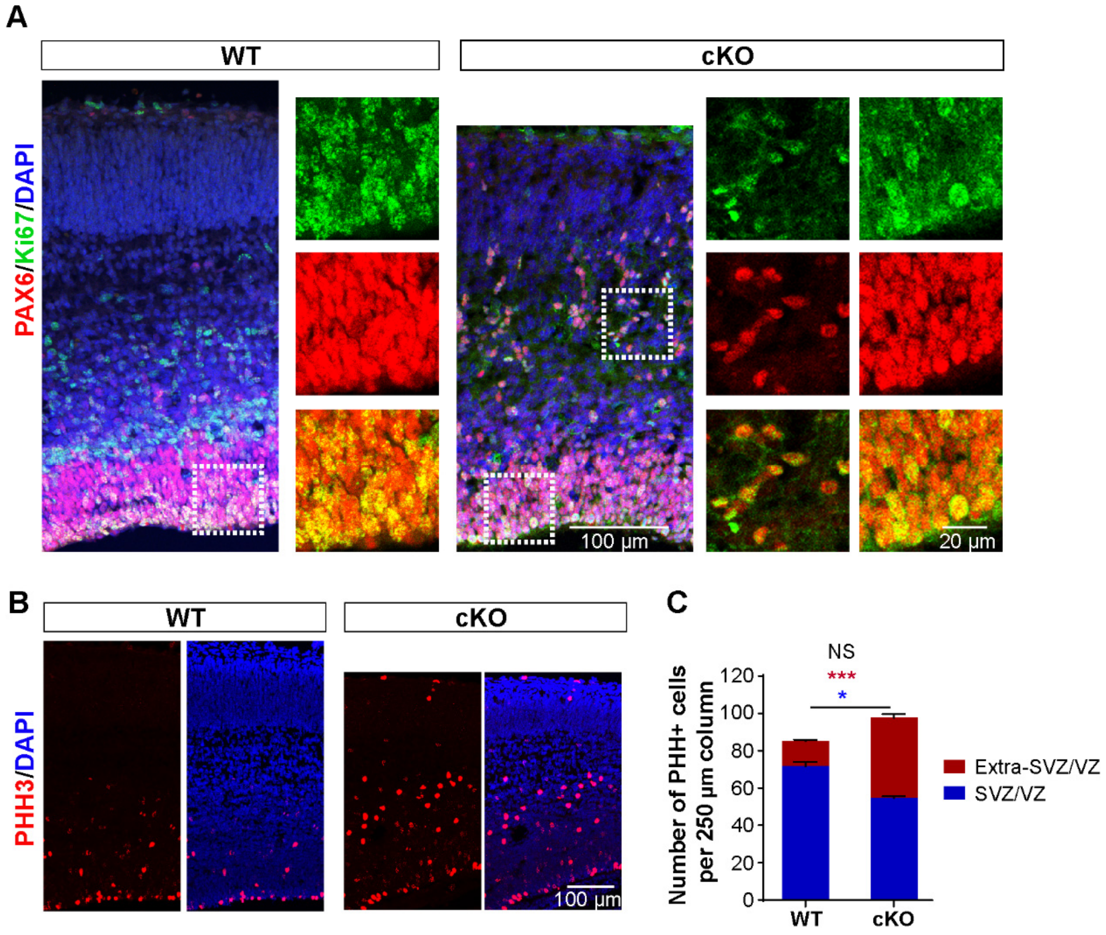

Supplementary Figure 5: Ectopic RGs remain proliferative. (A) Representative images of cortices stained for PAX6 and Ki67 in E16.5 WT and cKO brain sections. Boxed area are expanded into right panels. Arrow heads indicate delocalized RGs expressing PAX6 and Ki67 in cKO mice, suggesting that they are proliferative. (B, C) Representative images and quantification of a mitotic marker PHH3-positive cells in the cortices of E16.5 WT and cKO mice (WT, n=7; cKO, n=8 mouse brains). P values indicate significant differences of the number of total PHH3+ cells (*black*), in the VZ/SVZ (*blue*), and in the extra-VZ (*red*) between WT and cKO mice. Note that PHH3+ cells are delocalized in the cortical plate and the intermediate zone of cKO mice. *, $P<0.05$; ***, $P < 0.001$, two-tailed student t-test. NS, not significant. Bar graph data are represented as mean ± SEM.

Supplementary Figure 6

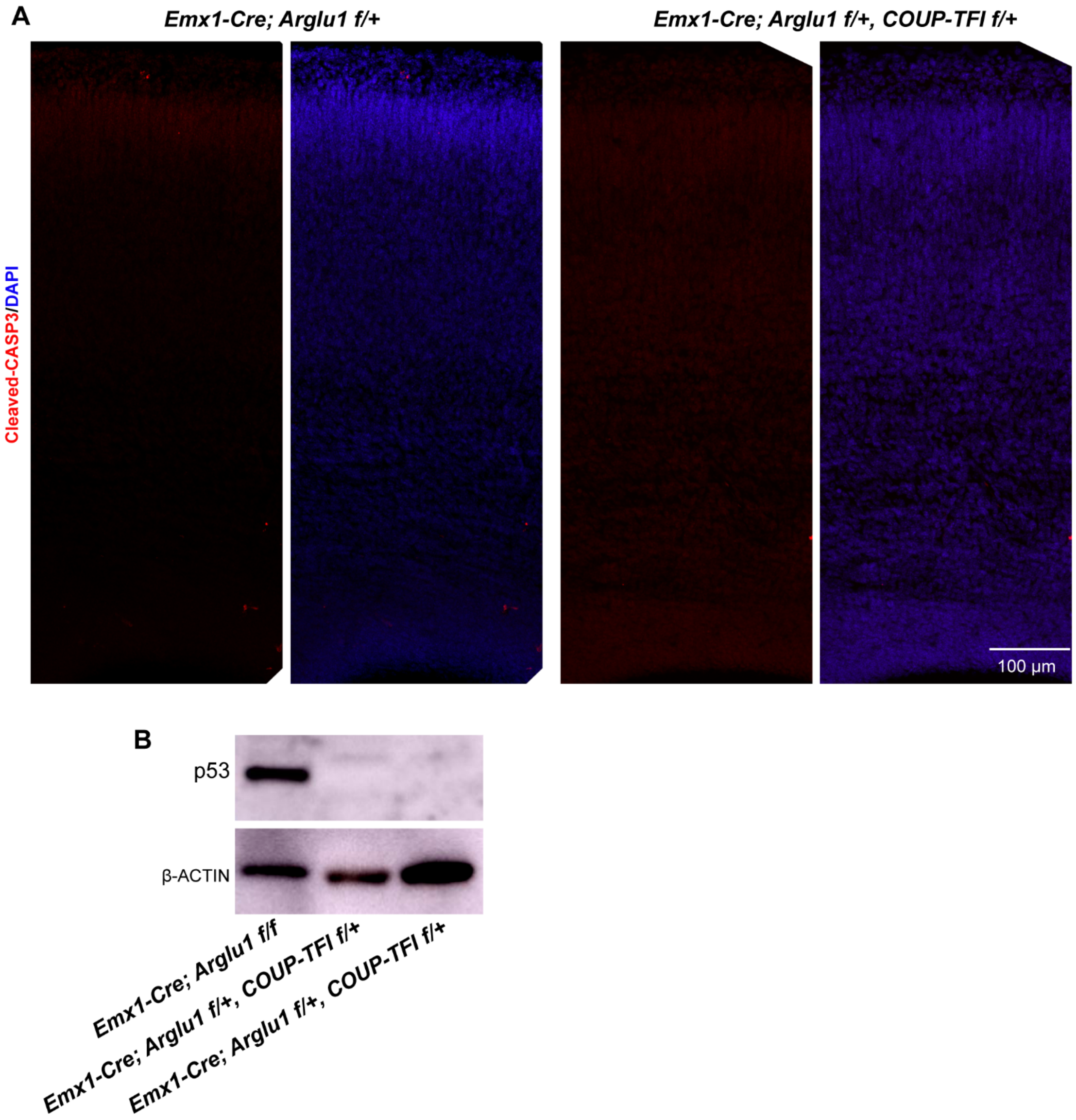

Supplementary Figure 6: Emx1-Cre-dependent recombination does not induce cell apoptosis. (A) Representative images of cortices stained for cleaved caspase-3 in E16.5 *Emx1-Cre;Arglu1*$^{fl/+}$ or *Emx1-Cre;Arglu1*$^{fl/+}$; COUP-TFI$^{fl/+}$ mouse brain sections. (B) Sample images of immunoblots of P0.5 cortical homogenates with antibody against p53 in *Emx1-Cre;Arglu1*$^{fl/f}$, *Emx1-Cre;Arglu1*$^{fl/+}$ or *Emx1-Cre;Arglu1*$^{fl/+}$; COUP-TFI$^{fl/+}$ mice. Experiments are replicated for 3 mice. Note that p53 is only expressed in *Arglu1* cKO mice other than the heterozygous mice containing the same number of loxp sites.

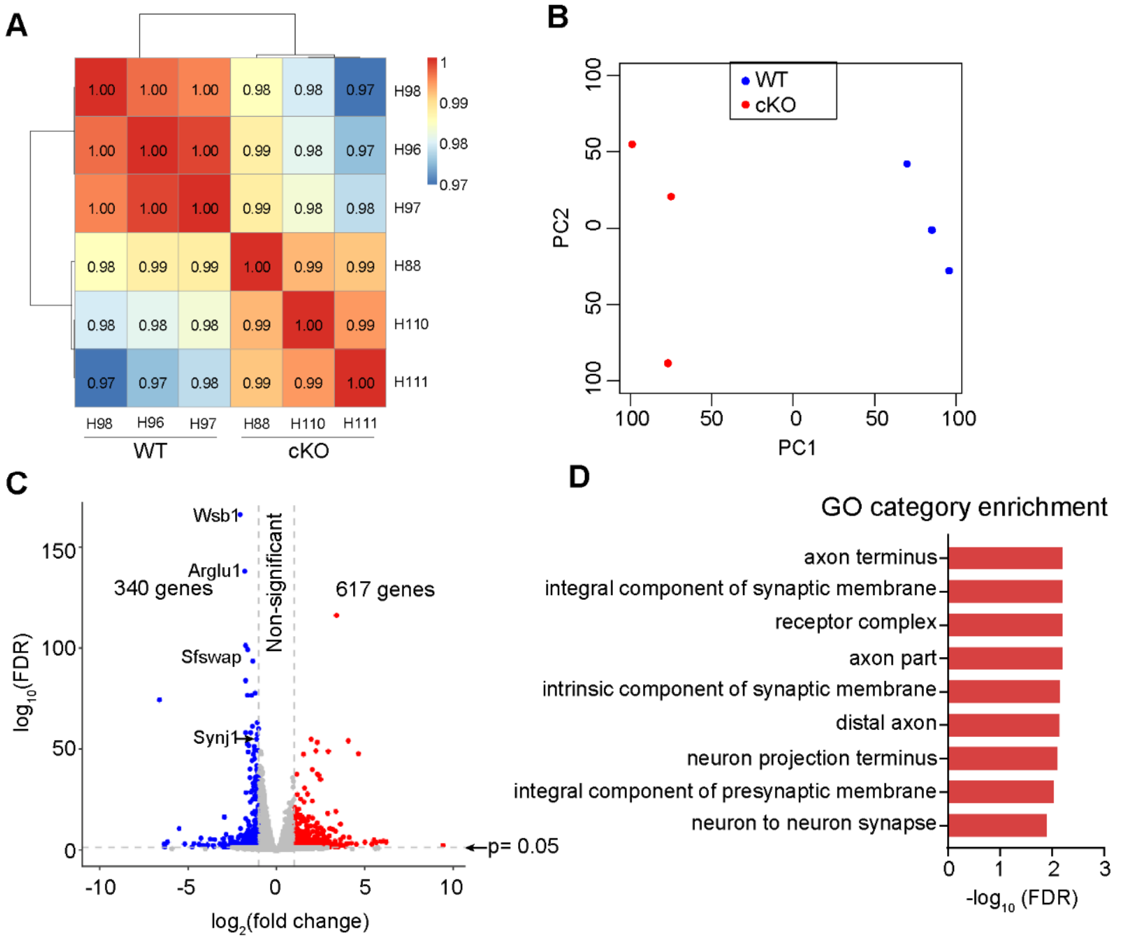

Supplementary Figure 7: ARGLU1 regulates gene transcription *in vivo*. Cluster (A) and principle analyses (B) show a significant correlation for data sets within WT or cKO groups, but not between WT and cKO groups, indicating RNA-seq data of good quality. (C) Volcano plot of $\log_{10}$(FDR) versus $\log_2$(fold change) for differentially expressed genes (also see Suppl. Table 1). Significance is defined as Padj<0.05. Significant regulated genes are highlighted by red (Upregulated) and blue (Downregulated). (D) Gene ontology analyses of differential expressed genes showing enrichments in neuronal functions only for downregulated genes in cKO mice.

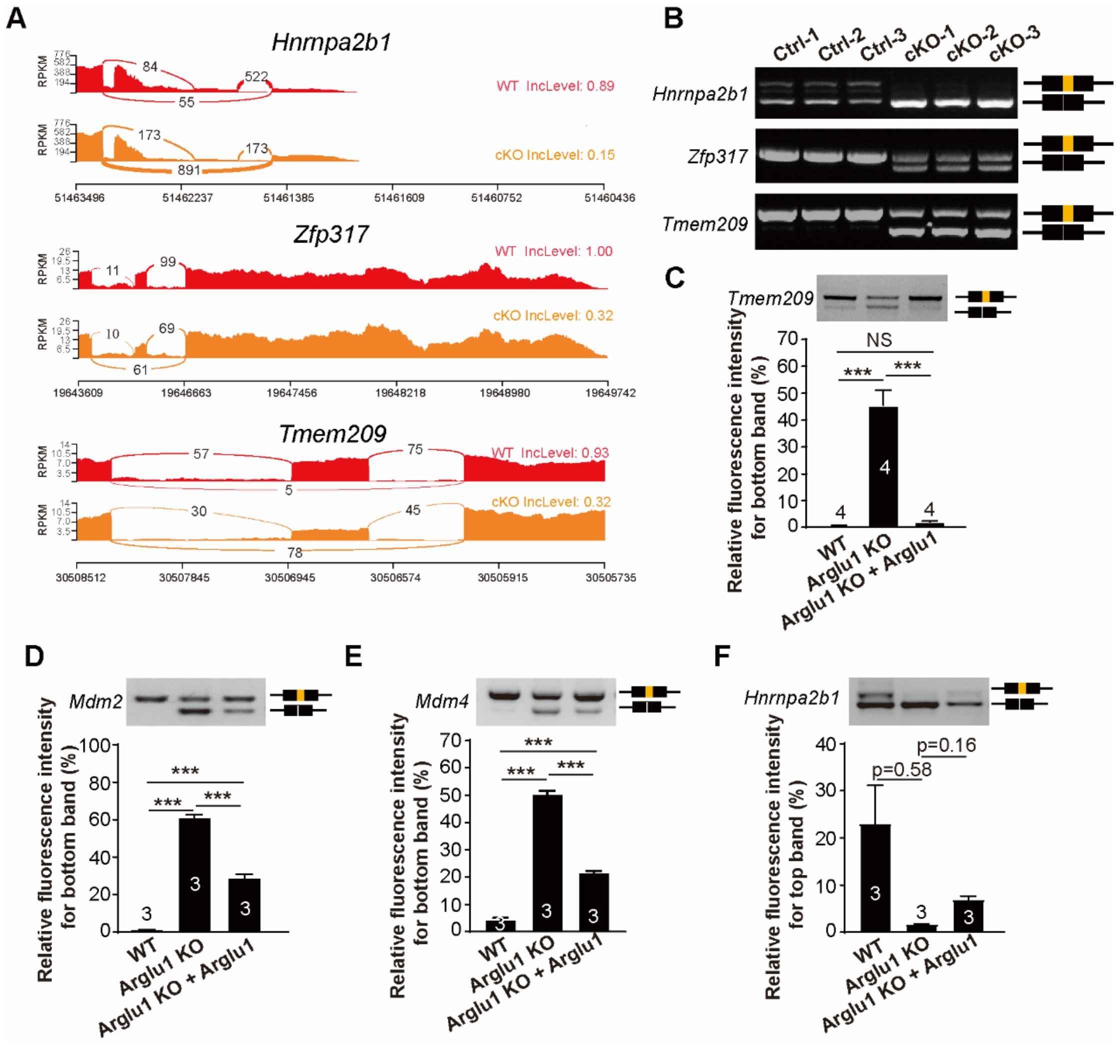

Supplementary Figure 8: Re-expression of ARGLU1 partially rescues differential alternative splicing. (A) Sashimi plots from bulk analyses showing alternatively spliced exons of *Hnrnpa2b1*, *Zfp317*, *and Tmem209*. (B) RT-PCR validation of *Hnrnpa2b1*, *Zfp317*, and *Tmem209*. (C-F) Representative images (*Top)* and quantification (*Bottom*) of RT-PCR showing alternatively spliced *Tmem209* (*C*), *Mdm2* (*D*), *Mdm4* (*E*), and *Hnrnpa2b1* (*F*) rescued by re-expression of ARGLU1 in *Arglu1* KO N2a cells. N number represents the number of experiments. ***, P < 0.001, one-way ANOVA with *post hoc* Bonferroni test. NS, not significant. Bar graphs are represented as mean ± SEM.

Supplementary Figure 9

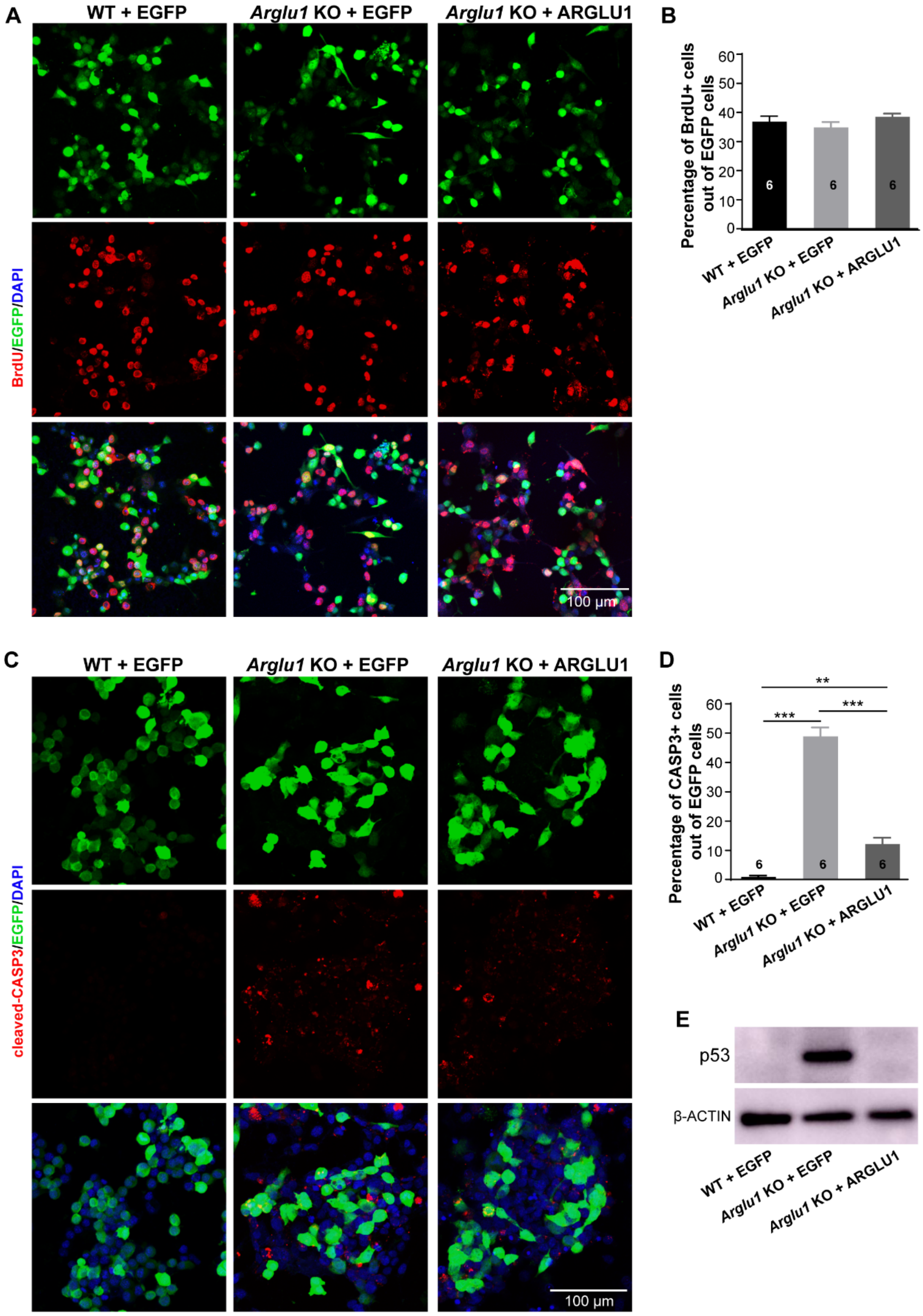

Supplementary Figure 9: Re-expression of ARGLU1 largely rescues cell apoptosis in *Arglu1* KO N2a cells. (A-D) Representative images of WT and *Arglu1* KO N2a cells with antibodies against BrdU (*A*), cleaved CASP3 (C) and quantification of percentage of BrdU+ cells (*B*) or CASP3+ cells (*D*) out of EGFP cells (*B*) . N2a cells were fixed

with 4% PFA 36 hrs after transfection with plasmids expressing EGFP only or Arglu1-IRES-EGFP (WT EGFP, n=6; KO EGFP, n=6; KO ARGLU1-EGFP, n=6 cell cultures). (E) Representative images of immunoblots of N2a cell homogenates with antibody against p53 showing a complete rescue of p53 expression after re-expression of ARGLU1 in KO cells (3 cell culture replicates). **, $P < 0.01$; ***, $P < 0.001$, one-way ANOVA with *post hoc* Bonferroni test. Bar graphs are represented as mean ± SEM.

Supplementary Table 1. The list of upregulated genes.

Gene Name	log2FoldChange	padj
B230216N24Rik	3.40105948	7.27E-117
Tmem86b	1.971829128	1.53E-55
Slc15a3	4.062542664	9.43E-55
Gm30223	2.318404022	6.02E-54
Tgfbr3l	2.244854303	9.97E-50
Gm5601	2.935021736	1.65E-49
Dqx1	4.642375033	2.54E-48
Gstm7	1.532875237	5.47E-48
Fam107a	2.03500832	1.46E-40
Gm5466	2.327137681	2.72E-38
Gm14204	1.148928191	3.12E-38
LOC105242595	2.385575029	9.93E-38
Eda2r	2.491593107	1.30E-35
LOC102640779	1.591950103	2.25E-31
Psrc1	1.758597968	1.88E-28
H19	1.177613119	3.11E-28
Fuz	1.362938123	6.33E-26
LOC105246114	2.037167756	7.23E-25
Hif3a	1.564170676	1.90E-24
Rbp1	1.047626508	1.51E-21
P2ry1	1.262310779	6.03E-21
Mbd3l2	3.363095719	9.49E-20
Mt1	1.033002775	1.17E-19
Htr3a	1.103036244	2.02E-19
LOC108168236	2.907509145	8.90E-19
Sult1a1	1.17887808	1.98E-18
Nxph2	1.347366535	5.74E-18
Net1	1.055191607	8.70E-18
Peg10	1.05847877	2.78E-17
Gm30807	1.836485794	4.37E-17
Gm14634	1.482758493	7.97E-17
Mttp	1.318404427	1.06E-16
Msh5	2.053592956	1.59E-16
Isl1	1.084196553	1.63E-16
Cd163	1.560870416	4.09E-16
4930581F22Rik	1.759471864	2.85E-15
2810468N07Rik	1.439817897	5.28E-15
Mc5r	1.619075527	6.30E-15
Fam71e1	1.006178146	7.02E-15
Zfp503	1.074826546	1.90E-14
Gm32178	2.313065869	4.28E-14
Rpa3	1.295895523	9.30E-14
Vax1	1.138318185	1.17E-13
Gm40668	3.6210356	1.69E-13
Gm40346	1.712196644	1.02E-12
4933421O10Rik	1.009675367	1.37E-12
Lrrc29	1.948794344	1.53E-12
Tas1r3	2.181284019	1.59E-12
Gm40688	1.830536153	1.91E-12
Gm41409	2.495349674	9.99E-12
Gm15517	2.331347351	2.13E-11

Hspb8	1.182500473	2.63E-11
LOC108167641	3.028097051	2.79E-11
Jpx	1.065479212	4.15E-11
Ms4a6c	1.732996508	4.91E-11
Piwil2	3.172638012	5.52E-11
Gm38524	1.047433536	1.24E-10
Ssc4d	1.757688824	1.33E-10
Gm39534	2.632103813	2.28E-10
Sp3os	1.062153173	3.69E-10
Gm32214	2.67896897	4.23E-10
Lacc1	1.351486762	5.54E-10
Gm12312	3.350734291	6.13E-10
Avil	2.064802015	6.87E-10
Trim72	2.646139561	7.88E-10
Gm13251	1.758549811	8.12E-10
Gm40721	1.497256332	8.76E-10
Myrf	1.762981977	9.36E-10
Gm32896	1.537825384	1.17E-09
Gm41901	2.290431175	1.43E-09
Alox15	1.064485289	1.50E-09
Pdk4	1.356264461	4.63E-09
Dlk1	1.217144205	5.43E-09
Gm38639	1.603186635	6.08E-09
Gm34696	1.085045277	6.18E-09
Tmem176a	1.225216443	6.80E-09
Gm15834	1.596919021	7.08E-09
Map3k6	1.434371414	1.00E-08
Ccdc122	2.442424398	1.08E-08
Spp1	1.131700681	1.90E-08
Gm4285	1.369281517	2.23E-08
Wbscr27	1.114356649	2.30E-08
Gm39099	1.872292949	2.42E-08
Galnt4	1.009937313	2.96E-08
Gm38504	1.193327203	4.20E-08
LOC108169121	2.641656641	5.40E-08
Junos	1.243405003	6.27E-08
C230037L18Rik	1.697413447	7.28E-08
Tspo2	2.335519021	8.28E-08
Acer2	1.3199726	8.58E-08
9530062K07Rik	3.000616209	1.21E-07
4933407L21Rik	1.200627425	1.23E-07
Ppp1r14a	1.604744946	1.63E-07
2610318N02Rik	1.211491885	1.70E-07
A930033H14Rik	1.006797529	1.86E-07
Rbm20	1.057175782	1.97E-07
Mal	1.749974805	2.34E-07
Dcxr	1.283946808	2.73E-07
LOC108168069	1.278453703	2.96E-07
Cyp4x1os	2.171711683	3.17E-07
Trim63	2.725896024	3.43E-07
LOC108167831	3.784090629	4.13E-07
Gpr35	1.805920614	4.84E-07
Ccdc188	1.413872887	4.91E-07

Gm40167	1.262301102	4.99E-07
Gm10516	1.122977701	5.95E-07
Gm40820	2.683694892	6.03E-07
Lhcgr	1.259076963	6.40E-07
Nqo1	1.14521786	6.52E-07
Gm35422	4.113131138	7.17E-07
LOC108167446	1.817789279	9.49E-07
Aspg	1.732113024	9.85E-07
Hist1h2ai	2.269089373	1.01E-06
2900052N01Rik	1.298239656	1.02E-06
Deptor	1.136777806	1.21E-06
Sytl5	1.081759752	1.51E-06
Gm19554	1.163569313	1.77E-06
Gm35002	2.883356632	1.88E-06
Gzmm	1.289062236	2.02E-06
Fgfbp1	1.196189871	2.27E-06
Tceanc	1.066273751	2.47E-06
Slc2a4	1.27695107	3.23E-06
Gm31953	1.507660256	3.67E-06
Gm33195	1.318648048	3.82E-06
Wfdc1	1.253114911	3.84E-06
Spag16	1.635667515	3.86E-06
Gm9925	1.395390957	3.87E-06
Ccdc78	1.457646701	3.95E-06
LOC108167693	1.471134463	4.60E-06
A430105J06Rik	1.328796804	4.79E-06
Gm31639	3.6303895	5.34E-06
Brs3	2.061507542	5.47E-06
Sgms2	1.168510656	5.61E-06
Adrb2	1.958326748	6.15E-06
Gm30259	1.15261741	6.30E-06
Ms4a6b	1.544336493	6.42E-06
5330434G04Rik	1.015348955	7.07E-06
Gm41313	2.436660964	7.22E-06
Gm34418	1.773442537	7.40E-06
Ang	2.139141849	7.61E-06
Gm12279	1.196617173	7.62E-06
Lrp8os2	1.041528259	9.63E-06
Gm33648	2.221485786	1.01E-05
Gm32666	5.517808628	1.07E-05
Tstd1	1.342957527	1.18E-05
Troap	1.001349908	1.22E-05
Ly6a	1.668264362	1.30E-05
Gm32934	1.823111435	1.31E-05
Galnt15	1.271482989	1.31E-05
Edn1	1.768186965	1.39E-05
Gm4988	1.619003286	1.47E-05
Gm16907	1.236179928	1.65E-05
Gm30604	2.552746251	1.73E-05
Gm39423	1.784366966	1.75E-05
Gm31282	1.102437534	1.75E-05
Gm41350	1.679232461	1.81E-05
Cox6b2	1.18691756	1.85E-05

Fhad1	1.431258701	1.87E-05
Gm29948	1.395680593	1.89E-05
Gm36146	1.985455587	1.90E-05
Gm42253	2.146316807	1.91E-05
Susd2	1.52840473	1.91E-05
Gm11755	2.529870196	2.09E-05
Aass	2.044350277	2.09E-05
Rmst	1.150397646	2.11E-05
LOC108168162	2.784308874	2.12E-05
LOC102641980	1.067450657	2.19E-05
Car14	1.000213417	2.32E-05
4833418N02Rik	1.057970513	2.33E-05
Eqtn	1.358360354	2.35E-05
Ms4a4a	1.662655749	2.38E-05
Dmrtc1a	1.590446801	2.64E-05
5031426D15Rik	1.483156199	2.64E-05
Gm40578	1.05116873	2.72E-05
2610020C07Rik	1.011096617	2.72E-05
LOC108167924	1.098905644	2.75E-05
Gm28905	1.775956468	2.93E-05
Gm39883	1.205849479	2.94E-05
Rsph4a	1.099268909	3.05E-05
Gm13241	1.417454784	3.22E-05
Gm40826	2.135185672	3.46E-05
1700019B03Rik	2.25504585	3.54E-05
Tmem45b	3.311360041	3.77E-05
Ooep	1.074082807	3.83E-05
Pm20d1	2.230255034	3.85E-05
Gm42270	6.073303872	3.98E-05
Ush1c	1.39447807	4.00E-05
Prss41	1.745331923	4.26E-05
Gm7972	1.218889743	4.56E-05
Gm40573	2.157652749	4.72E-05
Itga11	1.033270941	4.73E-05
Gm14210	6.007105881	4.83E-05
Speer4b	2.962733945	5.09E-05
Lmod1	1.757287225	5.35E-05
Txnip	1.101894133	5.37E-05
Grin3b	1.289162246	5.47E-05
Crispld2	1.402028013	5.60E-05
Gm33097	1.605220721	5.60E-05
Acta1	1.053069321	5.82E-05
Gm15510	1.266133257	5.82E-05
Dleu2	1.11816923	6.02E-05
BC030499	3.323971876	6.11E-05
Gm32650	2.748800045	6.16E-05
Pdzd9	1.288911966	6.33E-05
H2afb3	5.916489845	6.34E-05
Gm38480	1.442938976	6.55E-05
Neat1	1.212473189	7.05E-05
LOC108168876	2.010879618	7.29E-05
Arhgap40	1.669042366	7.57E-05
Gm32555	1.505525658	7.81E-05

Ifi44l	6.209739772	8.09E-05
E230029C05Rik	1.796721889	8.09E-05
Gm41795	2.353005169	8.58E-05
Fbxo36	1.292499196	8.77E-05
Gm41848	1.090069598	8.88E-05
Duxbl1	1.1656745	9.59E-05
Abcb4	1.272251378	9.90E-05
Dennd1c	1.847175471	0.000100255
Gm35782	1.41088387	0.000102661
Gm4890	1.353598672	0.000116477
Gm30712	2.906648911	0.000118504
Eno1b	6.20949994	0.00013189
LOC105247048	2.288425503	0.000137068
Gm3134	1.003514072	0.000143275
Gm16432	1.191134696	0.000143285
Gm42048	1.986877588	0.000151024
Mir708	5.013787938	0.000152294
Ldhd	1.61149149	0.000153758
A330069E16Rik	1.282792643	0.000155696
4933406C10Rik	1.282538677	0.000166173
Gstm2	1.037407391	0.000168913
Gm12522	1.936990401	0.000176452
Gcnt1	1.306439064	0.000177046
Gsx2	1.24512083	0.000178916
Vwa7	2.12010857	0.000189164
Myot	5.661641908	0.000205694
Gm39663	3.296388411	0.000232886
LOC108168201	2.213382641	0.00023743
Gm32186	2.03377543	0.000242836
Gm31532	1.711486068	0.000246362
LOC102640468	1.965595028	0.000250837
Gm13477	2.197835087	0.000255035
Gm32687	1.213311228	0.0002563
Tvp23bos	2.351640645	0.000284774
0610040B10Rik	1.112110227	0.000293115
Gm34343	1.948917767	0.000302032
LOC328448	1.283926	0.000306229
A830052D11Rik	1.296286611	0.000307947
Art5	2.551862791	0.000319807
Gm16124	2.237603468	0.000329193
LOC102632778	1.319355949	0.000339914
Samd3	1.491613345	0.000346982
Gm36195	1.262077106	0.000357096
Lmntd2	1.99898917	0.000362199
Gm40054	2.400064837	0.00038455
1700067K01Rik	1.317660091	0.000391817
Gm42047	2.082269374	0.000393765
LOC108168753	2.397836442	0.000402575
Gatsl3	1.190990768	0.000416346
Gm39059	2.531515253	0.000416873
Gm11423	1.646655559	0.000419107
Gm36500	1.219080057	0.000419612
Gm3519	2.64418878	0.000420358

Nkx2-3	1.207190901	0.000422603
LOC102636393	2.310053185	0.000429923
Gm10182	4.236930826	0.000432364
Baat	2.669754736	0.000437195
Prok2	1.59940778	0.000444827
Ttll13	1.485253728	0.000465849
Sapcd1	1.88067989	0.000472307
Gm33583	1.16640315	0.000474708
5830403F22Rik	1.879829593	0.000483215
Col9a1	1.015656542	0.000488069
Gm39275	3.044569502	0.000504066
4933432I09Rik	1.749113008	0.000548999
Gm41338	1.635650248	0.000549131
8430408G22Rik	1.935609029	0.000597229
Tcte2	1.161244193	0.000614044
Gm30122	1.506651578	0.000620557
Gm41297	2.187179134	0.000627678
Hmgn2-ps1	4.060599721	0.000652021
Gm17769	1.241475182	0.000653116
Gm32302	2.829023119	0.000660525
C1s1	1.610266232	0.000661104
Gm32151	1.611175945	0.000674541
Apoc1	1.98400297	0.0006788
G630064G18Rik	2.398665024	0.000686485
Myh6	1.201782705	0.00071119
Gm11944	1.124838079	0.000713791
5031434O11Rik	1.291854646	0.000738458
Gm41466	1.552424136	0.000763741
LOC108168353	1.750883608	0.000767564
LOC108168067	1.505581182	0.000797293
Sycp3	1.362850197	0.000802772
Gm12781	1.337364486	0.000857878
Olfr658	3.002643267	0.000870868
Tspan32	2.358174185	0.000879742
D730003I15Rik	1.081044913	0.000892327
Gm34421	2.182996605	0.000893499
Th	1.182955979	0.000895292
Gm7931	1.901740143	0.000906006
Gm42225	2.169682617	0.000908864
Gm27239	1.143725124	0.000923867
Rmi2	1.519808913	0.000962944
LOC102640250	1.237985811	0.000978369
Ribc1	1.386529975	0.00098119
Acad12	1.530551818	0.000986712
Gm29711	2.562550519	0.001009204
Hspb1	1.089473129	0.001056768
LOC108168402	1.630330623	0.001062651
Gm17224	1.151013138	0.001076932
Gm38648	1.637989489	0.001104741
Gm39021	3.60108407	0.001125549
Gm30335	3.02663856	0.001172657
Fscn2	1.235928539	0.001181085
Gm39252	2.844592927	0.001209003

Gm28578	3.94278114	0.001248865
Car3	1.784260659	0.001318308
Gm36210	1.3676323	0.001319628
Apol8	1.577874977	0.001349191
Gm38447	2.8713182	0.001372032
Gm6724	5.405142102	0.001394006
Gm10421	1.14126172	0.001448524
Gm17382	1.806585994	0.00145522
Angptl7	1.594486228	0.001504101
Cd86	1.436017885	0.001603316
Frmd7	1.467355249	0.001620817
Gm41145	4.523738786	0.001629641
Rbm47	1.068559347	0.001677433
Gm40269	1.705424647	0.001697343
Gm36193	3.27801197	0.001709765
Gm26559	1.490300011	0.001788635
1700019L22Rik	1.189063266	0.001815902
Sult5a1	2.171898936	0.001826513
Gm38440	1.13275772	0.001827258
Rab11fip1	1.24816583	0.001832498
Hist1h3a	2.503590434	0.001892499
Casc1	1.028844446	0.001912311
Ephx2	1.032241767	0.001945217
Aire	1.93115533	0.002077883
4930513L16Rik	1.972191556	0.002278062
Inca1	1.269961852	0.002324849
Gm41512	1.398615358	0.002351022
Acap1	1.154562929	0.002358582
Masp2	2.407246137	0.002371964
LOC108167456	1.850708049	0.002418545
LOC102640042	1.808683765	0.002451884
Gm39030	1.104611062	0.002457731
Gm30690	1.675059891	0.002470682
Tex29	1.487059528	0.002651624
A930007I19Rik	1.110002794	0.002654732
LOC102632415	1.43688139	0.002665487
Gm11827	1.837012585	0.00272069
Slfnl1	2.82932221	0.002779538
4930412L05Rik	1.27765341	0.002803236
Rbp4	1.256451144	0.002803236
Erich2	1.284714036	0.002890773
Gm39224	2.071840553	0.00312409
Slc52a3	1.288822174	0.003130827
Nr1h3	1.287883468	0.003134856
Plekhf1	1.582926637	0.003144694
Xrra1	1.248070879	0.003155273
Aicda	1.551265353	0.003208794
Gm30188	1.755841988	0.003224271
Col14a1	1.012221563	0.003237908
Gm36696	1.829461008	0.003341747
Kdm4d	1.263308581	0.003464998
Mpp4	1.130402989	0.00347961
Tdrd9	1.43962603	0.003504496

Mroh7	1.01408073	0.003720395
Gm17102	1.144100662	0.003724407
Steap4	1.826351567	0.00380887
Gm34571	2.519210826	0.003897351
n-TWcca1	3.756396163	0.003925504
Calml4	1.050934084	0.003936663
Mocos	1.811174277	0.004107443
Gm41215	2.46304538	0.004139811
Sspo	1.409300742	0.004171437
Gm38434	1.843198166	0.004229274
Gm30055	1.467387707	0.004302475
Scgn	2.019033945	0.004402056
Gm11223	3.680783958	0.004407631
Gm29443	2.561886436	0.004509812
Tmem252	1.029081456	0.004606786
Mtl5	1.30644616	0.004623241
Sytl1	1.920774958	0.004723816
Gm34354	1.015286267	0.004726399
Trim30d	1.363278128	0.004744573
Ldlrad1	3.717809745	0.004803745
LOC102639143	3.638799722	0.004949457
Cdkn2b	1.58418416	0.005119443
Gm39194	1.119660202	0.005158862
Gm42145	1.614827026	0.005202952
Rpph1	2.865842271	0.005209204
Gm14154	3.393009521	0.005215665
D330037F02Rik	3.050552634	0.005366915
Gm34851	1.238225884	0.005385951
Gm34917	1.186905166	0.005421757
Padi4	9.428526624	0.005698267
Dnaic1	1.183603948	0.005698267
Gm41024	1.894221896	0.005747946
Gm30352	1.282422694	0.005753126
BC106179	1.245699655	0.005947039
Slco1b2	2.173594982	0.005982848
Catsperg2	1.530261921	0.005995099
Gm42202	1.973234211	0.006030292
Rn7sk	1.640702056	0.006096866
Gm38766	1.078796783	0.006216637
Prr22	1.169749356	0.006319332
Gm38956	1.77044813	0.006322048
Gm33487	1.639944763	0.006322048
Acpt	1.344006336	0.006338115
Gm10698	1.15229875	0.006415328
LOC102639568	1.211378137	0.006455057
Akr1b8	1.329285949	0.006474155
Gm36523	2.104900827	0.006516101
AA465934	1.135931983	0.006574872
Gm2027	2.610144011	0.006800098
Gm32793	1.300773893	0.006817863
Gm30455	1.852203133	0.006862615
Prg4	1.07146819	0.0069401
Tbc1d21	3.043314777	0.007059166

Gsto2	1.790968476	0.007158876
Gm34979	1.259811778	0.007182526
Gm35566	1.699361205	0.007208809
Gm38664	1.243312688	0.007425138
Hist1h4d	1.822204402	0.007438878
Gm42063	1.029342032	0.007787016
Gm31966	2.229338827	0.007835516
Cilp	2.090246865	0.007996218
Gm39530	1.349876593	0.008037671
Gm39850	2.987002301	0.008038621
1700007L15Rik	1.805188266	0.008070995
P2ry14	1.22713446	0.008072366
Tgm4	1.449226054	0.008208575
Ppp3r2	1.706787613	0.008225604
C130060C02Rik	1.64931204	0.008228034
Edar	1.136433548	0.008270355
Gm29938	1.747228564	0.008281054
Gpr65	2.028741567	0.008307347
Rpl29-ps2	1.636727709	0.00850186
Mc1r	1.116733332	0.008516
Ptgir	2.95742335	0.008769201
Gm16894	1.241441921	0.008950766
Hist1h2bj	2.45898172	0.009069894
Gm31862	1.408530588	0.009190016
Gm26793	1.380935411	0.009230553
Gm33250	1.105772428	0.009652671
Msx2	1.864683969	0.009805066
Sptbn5	1.404314575	0.009987382
Efcab8	2.654043527	0.010309996
Spem1	1.780898446	0.010643769
Gm31054	1.470513186	0.010847979
Gm34278	1.62728606	0.011136933
LOC108167657	2.003694987	0.011252882
Ibsp	1.642880662	0.011261733
Gm26671	2.454378592	0.011575431
Gm42156	2.874098594	0.011750609
Gm33333	1.719111938	0.011854747
4933405L10Rik	1.725202425	0.011882047
Gm32304	1.843955473	0.01223488
Lppos	1.441228397	0.012412897
Gm34945	1.707023036	0.012453022
LOC108168969	2.617247997	0.012676502
Gm39271	1.992087426	0.012818321
2610037D02Rik	1.642024146	0.0132355
Gm10440	1.208719217	0.013331248
Gm9618	3.894215263	0.013456789
Loxhd1	2.662131026	0.013472713
Wfdc3	2.001650659	0.013548264
Gm36461	1.026347738	0.013665687
Synpo2l	1.40711887	0.01373359
Hist1h3h	2.110713022	0.013798596
Gm36101	1.94797879	0.014128997
Gm13154	2.733053028	0.014204691

Gm40540	1.208588766	0.014517493
Zbtb42	1.191174397	0.014602735
2010001A14Rik	1.186491821	0.01464555
Gm7488	1.355483531	0.014902471
5730422E09Rik	1.697659688	0.014943421
Gm42188	1.718421035	0.015116216
Ifi27l2a	1.234008677	0.015584397
Gm10282	1.11437439	0.015639538
Ucp1	2.275259065	0.015865568
Gm10263	1.29114723	0.016007662
Gm38862	2.715599129	0.016105692
Hist1h2ae	1.213315535	0.016262703
Gm26583	1.490417977	0.016381007
Sh2d6	1.45453764	0.016394897
Gm41287	1.030973614	0.016449119
Gm32398	2.186642103	0.016546898
Gm31852	1.344227156	0.016567763
Mafa	2.330711108	0.016686744
Zfp36l1-ps	1.542070174	0.016823114
Gm38566	1.001630648	0.017849193
Trp53cor1	1.741605726	0.018382397
Gm32123	1.764297816	0.018449758
Gm40010	2.043648436	0.018667412
Gm2109	3.408427771	0.018885135
Fam92b	2.486819133	0.019148016
Gm12506	1.834354944	0.019271865
Gjc3	1.610279808	0.019499667
Gm31344	1.452830126	0.019708453
Fbxo40	1.738140637	0.020125285
4930509J09Rik	1.075529623	0.020439084
A230020J21Rik	2.740663161	0.020635436
Aanat	1.178588551	0.02086494
Gm34505	1.245388995	0.020886617
LOC108169017	1.563100338	0.020956505
Gm26748	3.027373023	0.021296051
Gm34403	1.628540317	0.021521857
Gstm6	1.220357753	0.021522695
Marveld3	1.184618088	0.021721199
Slfn2	1.293570532	0.022004174
LOC102632465	1.442005821	0.022085828
Rps15a-ps6	3.492981351	0.022199809
Kcnn4	1.663753669	0.022241223
Wfikkn1	1.077915057	0.022268085
Gm39676	1.647686673	0.022511628
Gm36905	3.275899438	0.022582478
Ren1	2.542023411	0.022919393
Pctp	1.168209553	0.02317811
Bhlhe23	2.786123204	0.023330874
LOC108168144	1.391732476	0.023382569
6030443J06Rik	1.186257674	0.023486775
Gm35281	1.045751761	0.023618806
Gm41461	1.957115013	0.023690957
Gm21284	1.603988703	0.023690957

Gm35476	1.676545254	0.023980469
Gm36311	2.171112081	0.024533526
Scnn1b	1.582967505	0.02475453
Gm34128	2.693019204	0.024755206
Cd59a	1.359389577	0.025527374
Cd2	1.957088781	0.025593581
Arr3	1.231681572	0.025607824
LOC102634584	2.020215839	0.025793405
Mmel1	1.398867579	0.025934243
Gm40777	2.486539233	0.026157475
Gm35379	1.569033469	0.026431509
Slc10a6	2.168608182	0.027160362
Gm5067	1.905819992	0.027554388
Hsf4	1.10339866	0.027643841
Ccl9	1.110771435	0.027791385
Gm39483	1.719211486	0.027801756
9430060I03Rik	1.524772127	0.027839928
1810044D09Rik	1.005750676	0.027900077
LOC102634887	1.552533019	0.027908318
LOC105246847	2.102047256	0.028342053
Gm32970	1.040430626	0.028355918
LOC108168075	1.665360619	0.028601394
Gm40588	2.342603017	0.028926848
Ankmy1	1.495009581	0.028940229
Hist1h4c	2.333370155	0.029243149
Gm5898	1.180946954	0.029309255
Gm31227	2.959037178	0.029623908
Gm31893	2.15272716	0.030163416
Prdm12	1.11755507	0.030527305
Gm26908	1.214014401	0.030566684
Gm41590	2.444969419	0.030694374
Aox3	1.014873335	0.030912479
Krt73	1.214286182	0.031034137
Gm36904	1.056984337	0.031056635
Gm33159	1.546039628	0.031131806
Gm40180	1.290882587	0.031154291
Acss3	1.19859195	0.031446572
Gm32057	2.041579025	0.031519006
9530053A07Rik	1.194106014	0.032708338
Clec3a	1.393441451	0.033837901
LOC108168248	1.427561102	0.034349635
Nkx6-2	1.966053714	0.0347511
Gm33858	2.638574097	0.035263411
B3gnt8	1.186079555	0.035363862
Eppk1	1.008642984	0.03544827
Ifitm7	1.445335262	0.035687259
Tlr8	1.480536996	0.036265933
Otoa	1.255832598	0.037203935
Cd101	1.878895758	0.0377088
Ifi44	1.400303881	0.037764498
Gm13248	1.355688798	0.037896072
9430024E24Rik	1.965240445	0.038391674
a	1.356104895	0.038671597

Olfr1372-ps1	1.877290535	0.039036851
Galnt6	1.062139921	0.039321053
Muc2	1.096651915	0.039593498
Hist1h2bk	2.282031611	0.039739231
C230072F16Rik	1.036857641	0.039863958
Tex35	1.409443657	0.040140153
LOC102636024	1.276653718	0.04065293
Gm37548	1.541990384	0.040750753
1700123O21Rik	2.019607703	0.04086739
Mybpc1	1.339895663	0.041032954
Mb	1.749368217	0.041303705
Thbs4	1.01247249	0.041341793
Gm10914	1.219250728	0.041622127
Ftcd	1.761779188	0.042053669
Mrap	1.976048012	0.042132741
Wt1	2.376622637	0.04227954
Gm34746	2.303701574	0.042313769
Gm6657	1.352548618	0.042857932
Gm29917	1.883465375	0.043221517
Atp2a1	1.202286682	0.043260117
A630066F11Rik	1.042803768	0.043324558
LOC108168977	2.571979977	0.044201857
Gm29724	2.59235565	0.044872734
Cstad	1.723976125	0.044937162
BB123696	2.340062669	0.045749073
Gm41033	1.756526932	0.046534351
4932443I19Rik	1.011003683	0.046856159
Cfap58	1.035328562	0.047054589
Cpa1	1.60329932	0.047577281
Gm34385	1.728754331	0.047664153
Gm15607	1.204714806	0.048230626
Slc44a3	1.900830952	0.048714421
LOC102633627	1.439844901	0.048801314
Gm31792	1.208435836	0.048995783
Nrk	1.139698566	0.049247525
LOC108167592	1.028425839	0.049722377

Supplementary Table 2. The list of downregulated genes.

Gene Name	log2FoldChange	padj
Gm6969	-6.616245242	5.34E-75
Gm9855	-6.339671236	0.000958501
Gm6937	-6.327546063	0.034860447
Hsd3b1	-6.151914276	0.000115796
Hist1h2al	-5.506205694	2.93E-11
LOC108168958	-5.196270425	0.001182268
Avp	-4.775513245	0.029191048
Gm38492	-4.674798162	0.000702174
Gm8730	-4.382405747	0.001686536
Hao2	-4.35321121	0.003314787
Gm41265	-4.343813201	0.002717778
Gm11820	-4.268765826	1.57E-06
Gm14165	-4.163031537	0.004378444
Adamdec1	-3.937709651	0.018110875
Gm3375	-3.906233412	0.002706945
Gm6776	-3.881566003	0.004260728
Gm7827	-3.739461892	4.21E-06
Gm38880	-3.575451629	0.006406176
Pvalb	-3.528948581	0.017753126
Gm8707	-3.495669529	7.45E-06
Gm41363	-3.209588379	0.001123313
LOC108168932	-3.01182853	3.73E-06
Dnajc5b	-3.005569501	0.027047864
LOC108168824	-2.956260903	0.008520317
LOC108168281	-2.946008658	6.11E-17
LOC105244019	-2.945051423	0.020450985
LOC108168499	-2.935850914	0.000372132
1700080N15Rik	-2.92012315	0.026452863
9530036M11Rik	-2.908932662	0.036521906
Cd209f	-2.871275645	0.005153982
Gm41877	-2.858925838	2.23E-08
Gm30116	-2.857301848	0.012596341
Gm15319	-2.759683048	0.034665885
Il1rl1	-2.739642514	0.014775134
LOC108168993	-2.656611685	0.01223488
Gm7707	-2.640234351	0.001747529
Gm40235	-2.629303159	1.22E-06
Gm15104	-2.614109606	0.002357112
Gm12751	-2.607168336	1.03E-05
Gm38650	-2.588223878	0.009058358
Mir1188	-2.543111352	0.002526205
Gm9625	-2.531225931	1.02E-06
LOC108168305	-2.516953495	0.047730811
Gm30658	-2.489740382	0.043282272
Gm10819	-2.468856367	0.005829889
Gm33697	-2.467719742	0.030549034
Gm14637	-2.453803739	0.008014331
Prss52	-2.450457227	0.002377352
Gm27887	-2.441440311	0.00530871
Gm35599	-2.439215064	0.000110847
Hsd3b6	-2.413412862	0.018781979

Gm36267	-2.365227281	0.020513188
Adgrf4	-2.359035813	0.003605304
Cers3	-2.349530227	0.000480128
Gm41783	-2.330772853	0.004813181
Mir1970	-2.324991215	0.036811371
Gm39314	-2.299700987	0.046005696
Hsd3b3	-2.282909257	0.000262863
LOC102635661	-2.275641081	0.011136933
LOC108167491	-2.256931648	0.018854037
Gm36221	-2.203512043	0.00051761
Gm9726	-2.177129128	0.026180904
Ciita	-2.169510093	1.58E-06
Efcab3	-2.164010671	0.035046874
Gm15753	-2.147609569	3.33E-07
Gm31416	-2.10999793	0.049975175
Wsb1	-2.063290584	8.89E-167
LOC108168814	-2.055960308	0.000533545
Gm31214	-2.041514406	2.74E-11
Gm31326	-2.028729548	4.91E-08
LOC108168384	-2.003120346	0.012717765
Gm14327	-1.990111955	0.009877272
Gm20219	-1.977157306	9.26E-08
Mir1968	-1.958901844	0.026758251
Gm33467	-1.92653218	0.003189905
Lrrc26	-1.919048632	0.000497889
Msx3	-1.902643825	3.89E-05
Gm34676	-1.897375786	0.02052189
Lmx1b	-1.891176899	0.049840498
LOC102637219	-1.888301038	0.049761293
Gm33585	-1.883037728	0.021724716
LOC108167726	-1.882300414	5.04E-05
G430095P16Rik	-1.839480794	1.21E-06
LOC107988026	-1.82299568	0.038374004
LOC105243866	-1.821599235	0.00850415
Tgm3	-1.816449757	0.001101058
9130227L01Rik	-1.815823031	0.022785101
Gm1141	-1.810132674	0.016293736
4933408B17Rik	-1.800592751	4.28E-07
Arglu1	-1.79972163	1.14E-138
Tmem210	-1.796307344	0.007615712
LOC108168839	-1.793212406	0.027074296
Esr1	-1.783377455	1.59E-05
Gm3173	-1.775249066	3.22E-06
Mir1249	-1.766795777	8.54E-06
Gm36392	-1.76557566	4.47E-08
LOC102636733	-1.750768142	0.021147474
Trank1	-1.750626065	5.84E-102
Lmo7	-1.747120944	1.37E-84
Nwd2	-1.745657593	9.30E-59
Gm31336	-1.738669398	0.024161825
Srrm4os	-1.738356929	2.77E-16
Gm16035	-1.734055876	0.028166301
Usp43	-1.720802494	4.25E-29

LOC102640098	-1.718831621	5.35E-05
Gm40996	-1.716185585	5.86E-05
Flt3	-1.714011079	0.034387971
Ccl17	-1.713739077	0.01784315
LOC108168986	-1.678498993	0.022725177
Hbq1b	-1.673579679	4.09E-09
LOC108167915	-1.671270966	0.002662061
Gm32949	-1.665116617	0.000718025
LOC102639013	-1.663447587	0.000401965
1700093K21Rik	-1.660217738	0.000450292
Gm41595	-1.656280466	2.04E-05
Prkcg	-1.650991622	3.10E-53
Atp11b	-1.645428129	2.07E-77
Sfswap	-1.628754342	5.81E-100
Gm39786	-1.622504871	0.033824374
Mctp1	-1.596815861	3.28E-49
Satb2	-1.596437615	1.42E-10
LOC108168936	-1.591228699	7.56E-15
Tcf24	-1.575403965	0.009039334
Gm40393	-1.575388802	0.007239136
LOC101055992	-1.572990034	0.039263073
Ptprk	-1.565966731	1.69E-52
LOC108167431	-1.562644231	5.07E-13
Smyd1	-1.560614538	0.002061518
Gm42111	-1.560328467	0.007338662
5730409K12Rik	-1.559475926	0.034983029
Tbrg3	-1.544663491	0.03693625
Gm34180	-1.538912107	0.040348551
LOC108168740	-1.532488911	0.004716601
5930438M14Rik	-1.532446179	3.18E-07
Adgrf2	-1.529030505	0.03089954
LOC108169120	-1.528174023	0.018248857
Gm32005	-1.525496029	0.029243296
Slc26a4	-1.5185499	0.03161037
Slc47a1	-1.515821662	0.008420174
Gm15423	-1.515111477	0.003781694
Gm12426	-1.514255997	8.76E-13
Gm32849	-1.513644631	0.013973306
Cldn20	-1.509592915	0.003186649
Mgam	-1.505626163	0.032064334
Ccno	-1.503712617	0.017462544
Nr4a3	-1.499100415	1.82E-36
Enkur	-1.495508685	4.56E-05
Met	-1.495203204	8.48E-41
Gm39704	-1.488267168	0.049761293
Robo3	-1.48816731	2.79E-06
LOC108168877	-1.486378656	0.003949568
Dnah12	-1.482458014	0.001407301
Grm8	-1.480530741	3.14E-09
Gm33747	-1.479888535	0.008478526
Cfap69	-1.475870152	1.91E-24
Slc6a4	-1.471720906	0.000228909
Gm34576	-1.462238155	0.005433221

Cacna1g	-1.455721887	9.30E-59
LOC108167681	-1.452750412	0.004744573
LOC102635997	-1.450704745	0.022558655
Gm31090	-1.45067351	0.0214024
Gm15186	-1.449418035	0.045463477
Gm3164	-1.448836674	0.033114815
Mir344-2	-1.447505866	0.020220129
Nlrp5-ps	-1.445487107	1.59E-05
Tmem255b	-1.445248534	0.01457545
Tdrd6	-1.4391966	0.000163732
Crlf1	-1.438085037	2.25E-29
Micalcl	-1.43076701	1.21E-06
Gm4876	-1.426875217	0.027644978
1700020D05Rik	-1.421878343	0.000575167
LOC102634333	-1.412170822	0.000124936
Atp8a1	-1.410867207	2.55E-77
A830009L08Rik	-1.40368434	0.002768766
Ptgfr	-1.398054787	0.003855945
Rasgrf2	-1.378245205	6.37E-45
LOC108168306	-1.378068144	1.76E-08
Prrt1	-1.373240603	5.58E-30
Cpeb1	-1.36950809	2.27E-09
Col15a1	-1.358146154	8.09E-19
Gm32528	-1.3575256	7.76E-10
Gm3002	-1.357385087	4.80E-07
Slc9b2	-1.356288902	6.08E-06
Gm5069	-1.355879407	0.005098306
Sptlc2	-1.354509859	6.97E-62
Gm8773	-1.352069227	1.13E-06
LOC108168282	-1.344468909	0.010927114
Gm28154	-1.344308121	0.025046828
Cdc20b	-1.339148323	0.003723058
Ptk2b	-1.337242546	1.00E-25
2810442N19Rik	-1.333411107	0.030778255
Pfkp	-1.332974218	3.30E-94
Mcidas	-1.322216678	0.029637849
Rasl10a	-1.319239497	0.000919967
Gm28175	-1.316306419	1.04E-33
Nfix	-1.313964892	7.12E-11
Gm30810	-1.313742061	5.75E-05
Mkx	-1.311268106	9.95E-09
LOC108169128	-1.309218331	0.005135473
Arhgap6	-1.302004641	1.99E-06
Dok5	-1.300816321	5.81E-48
Kcnk4	-1.296637986	0.000451324
Prok1	-1.296429814	0.048774044
G530011O06Rik	-1.2956272	0.000971707
Tbc1d5	-1.285826312	4.47E-32
Gm33320	-1.27943539	0.045856357
A3galt2	-1.276636993	0.000187268
Tmem30c	-1.275222745	0.045072062
Ccl28	-1.268882415	0.000235132
Gm39014	-1.266610193	0.03195191

Tshr	-1.259313397	5.51E-06
Crhr1	-1.258112928	1.37E-19
Ptprd	-1.252726715	4.24E-52
Gm32643	-1.243906342	2.53E-05
4930426D05Rik	-1.24380599	0.041450337
Gm10419	-1.243356637	4.40E-15
Clk1	-1.238985261	7.59E-46
Colgalt1	-1.236630226	3.49E-50
Gm41688	-1.234908036	0.009240474
1700109K24Rik	-1.234232989	3.37E-09
Npas2	-1.230586566	1.05E-12
Dcdc2b	-1.227427354	0.000279396
Zscan26	-1.223323872	1.52E-27
Gpc5	-1.218675457	7.77E-07
Gm13199	-1.218432939	0.002715758
Alpk1	-1.21764299	0.002800191
Gm32133	-1.216622489	0.004484231
LOC108167356	-1.2156062	0.005957374
Gm2164	-1.212558394	2.00E-19
Grm7	-1.2081124	5.27E-26
LOC108168881	-1.206591921	0.003109857
Opcml	-1.204638927	7.63E-50
Grm2	-1.202767994	1.59E-15
Gls	-1.201917797	1.87E-78
Tnfsf10	-1.199083181	0.020709377
Fank1	-1.197719049	5.42E-05
Cpne9	-1.189217613	0.002976692
Nav1	-1.188628979	3.55E-35
Gm33877	-1.187939499	0.002061262
Npas4	-1.18682717	8.74E-15
Mvd	-1.18460619	1.14E-36
Spx	-1.184597971	5.47E-05
LOC108169162	-1.181617443	0.006741312
Mas1	-1.181091846	0.029286926
Rxfp1	-1.180745817	1.82E-12
Nmrk1	-1.172529075	0.005148124
Gm10277	-1.168688657	0.00230841
A930013F10Rik	-1.16784925	3.25E-18
Rgsl1	-1.165754699	0.005433221
Doc2a	-1.164915836	1.09E-18
Guf1	-1.164809082	1.18E-33
Nnmt	-1.162111996	0.0019424
Myo19	-1.160292639	6.69E-14
Adamts3	-1.156766066	9.47E-30
Gipr	-1.156753581	9.25E-20
Ephb6	-1.156305181	7.85E-26
Fancg	-1.146258509	1.36E-15
Gm27197	-1.144658101	7.22E-07
Pcdhgb1	-1.140104352	1.70E-05
A930017M01Rik	-1.139329629	5.29E-09
LOC108167761	-1.138795679	0.014201775
Gm42344	-1.135482422	0.030141471
Styk1	-1.134364896	9.78E-07

Fam65c	-1.133078295	0.031055319
2310067E19Rik	-1.127396687	0.0174956
Gm42158	-1.125244348	0.003245815
Ccdc116	-1.124434707	1.26E-09
Prss12	-1.123376418	1.51E-13
Gm2808	-1.122358651	0.007327088
Far1os	-1.121928441	5.21E-05
Sorcs1	-1.120086502	2.91E-27
Itsn1	-1.117848885	1.35E-55
Gm7890	-1.117546549	0.02885267
Adamts16	-1.115685268	5.43E-05
Bend6	-1.114205606	1.71E-40
Gm15764	-1.109487067	0.027155889
Necab1	-1.109091068	4.38E-37
Prkag2	-1.108958133	1.77E-42
Gm5093	-1.108861835	8.55E-09
Ccbe1	-1.105474132	0.011984992
Slc26a7	-1.101485893	2.14E-12
D130043K22Rik	-1.101471844	3.24E-15
Galr2	-1.09699873	0.007124114
LOC105244195	-1.095862336	0.032978676
Gm30248	-1.092751929	5.40E-08
Matk	-1.087236406	1.03E-19
Cdk18	-1.087006291	0.017349306
Kpna2-ps	-1.08459016	4.15E-06
Gm36117	-1.083879456	4.41E-05
Fbln7	-1.082740749	0.008774813
LOC101055754	-1.081509935	0.000151414
Grp	-1.079627227	1.93E-10
Rbm5	-1.079376971	9.04E-64
LOC108167879	-1.079158469	0.047545022
Npff	-1.076869201	0.014273921
Ntf3	-1.076540463	5.49E-08
Synj1	-1.0747006	6.60E-58
Zbtb40	-1.071013299	5.44E-10
Gm26868	-1.068428874	0.000101256
Cdkl2	-1.066836406	6.60E-26
Map1a	-1.066100512	1.07E-31
Sema7a	-1.063324783	1.54E-34
Tlr12	-1.061471231	0.04327787
4930467E23Rik	-1.051798743	9.76E-05
Cacna1d	-1.051797281	5.10E-31
Osbpl1a	-1.050252607	2.79E-39
Gm31443	-1.049752588	0.025384136
Pth1r	-1.048302436	2.83E-09
Tec	-1.048041168	6.87E-06
Lrrc45	-1.047024784	3.00E-19
Myo16	-1.04594077	2.62E-36
Ak6	-1.044290712	6.20E-11
Gabrb1	-1.042977454	2.15E-26
Nvl	-1.040860597	6.79E-50
Pou3f1	-1.040714499	1.46E-18
Apln	-1.037987936	8.41E-05

Chrna3	-1.035888046	0.000173187
Dmtn	-1.035786091	6.68E-26
4931403G20Rik	-1.035766916	0.034538715
Myo9b	-1.035222904	5.98E-30
3300002P13Rik	-1.035040918	0.000120451
Egr4	-1.032156055	0.042426272
Hook1	-1.029455379	1.59E-36
Zbed6	-1.024957119	7.51E-20
Ackr1	-1.024949838	2.07E-21
Ddn	-1.023809657	1.85E-22
Adamts20	-1.023726542	1.67E-18
Gm31305	-1.02347323	0.000172956
Ccdc67	-1.02141495	0.010987339
Sfpq	-1.019677984	1.87E-39
Lrp1b	-1.019473683	2.76E-19
Ntsr1	-1.016979856	7.34E-13
Camkv	-1.015417814	4.04E-36
Grin2b	-1.015301948	8.63E-17
Aim2	-1.010131578	3.75E-08
Rimklb	-1.008353775	1.12E-60
Chka	-1.006788229	7.40E-34
Rxfp3	-1.006333898	0.000218047
Gm32080	-1.006282715	0.00104235
Pum3	-1.004593124	1.16E-33

Supplementary Table 3. The list of alternatively spliced events.

Events	chr	geneSymbol	strand	pos	IncFormLen	SkipFormL	WT_IncLe	Treated_In	IncLevelDi	ABS(IncLe	PValue	FDR
SE	chr6	Hnrnpa2b1	-	51460434-51461433:51461920-51462055:51463411-51463493	284	149	0.8527	0.1523	0.7	0.7	0	0
SE	chr7	Plekha1	+	130908336-130908425:130909588-130909658:130910832-130913308	219	149	0.8193	0.1263	0.693	0.693	0	0
SE	chr7	Plekha1	+	130908336-130908425:130909588-130909735:130910832-130913308	296	149	0.816	0.1723	0.644	0.644	0	0
SE	chr11	Wsb1	-	79241984-79242156:79242499-79243575:79244463-79244563	298	149	0.7853	0.148	0.637	0.637	0	0
SE	chr5	Magi2	+	20651055-20651193:20661242-20661352:20702070-20702152	259	149	0.8227	0.196	0.627	0.627	0	0
SE	chr9	Zfp317	+	19643609-19643704:19645212-19645307:19646702-19649743	244	149	1	0.3867	0.613	0.613	0	0
SE	chr7	Folr1	-	101863925-101864094:101868398-101868554:101869157-101869304	298	149	0.9137	0.3093	0.604	0.604	0	0
RI	chr5	Sfswap	+	129501233-129501596:129501233-129504105:129503983-129504105	298	149	0.6617	0.0593	0.602	0.602	0	0
SE	chr7	Folr1	-	101863925-101864094:101868398-101868551:101869157-101869304	298	149	0.9267	0.3377	0.589	0.589	0	0
RI	chr15	Prpf40b	+	99304425-99304490:99304425-99305433:99305408-99305433	298	149	0.7267	0.1403	0.586	0.586	0	0
SE	chr2	Arhgap21	-	20879961-20881851:20883200-20883229:20887106-20887160	178	149	0.7623	0.181	0.581	0.581	0	0
SE	chr6	Tmem209	-	30505733-30505974:30506786-30506917:30508454-30508512	280	149	0.9043	0.3293	0.575	0.575	0	0
A5SS	chr5	Sfswap	+	129501233-129501596:129501233-129503593:129503983-129504105	298	149	0.616	0.042	0.574	0.574	0	0
SE	chr11	Tnrc6c	+	117739867-117740009:117741645-117741818:117742927-117743064	298	149	0.8857	0.3173	0.568	0.568	0	0
SE	chr1	Mdm4	-	133003791-133003890:133004654-133004692:133009137-133009192	187	149	0.7373	0.177	0.56	0.56	2.26485E-14	3.01E-12
SE	chr15	Ly6h	-	75566132-75566259:75566714-75566831:75567095-75567123	266	149	0.8547	0.2987	0.556	0.556	0	0
SE	chr2	Rtel1	+	181349669-181349709:181349895-181349980:181350126-181350203	234	149	0.964	0.4177	0.546	0.546	0	0
SE	chr3	Rsrc1	+	67349909-67350015:67355474-67355626:67356221-67358398	298	149	0.8957	0.3507	0.545	0.545	0	0
SE	chr7	Folr1	-	101863925-101864094:101868398-101868470:101869157-101869304	221	149	0.9173	0.3723	0.545	0.545	0	0
SE	chrX	Atp2b3	+	73555053-73555235:73559864-73559950:73570234-73573275	235	149	0.18	0.7243	-0.544	0.544	6.19504E-14	7.8E-12
SE	chr6	Luc7l2	+	38551334-38551908:38554980-38555063:38568847-38568917	232	149	0.3707	0.912	-0.541	0.541	0	0
SE	chr6	Wnk1	-	119952685-119952969:119953771-119953902:119956071-119956196	280	149	0.7317	0.1967	0.535	0.535	1.11022E-16	1.97E-14
SE	chrX	Atp2b3	+	73555053-73555235:73559864-73560017:73570234-73573227	298	149	0.2307	0.7573	-0.527	0.527	7.21645E-15	1.06E-12
SE	chr3	Col11a1	+	114133136-114133189:114138171-114138186:114138400-114138453	164	149	0.823	0.2957	0.527	0.527	1.6932E-12	1.7E-10
SE	chr7	Plekha1	+	130908336-130908425:130909588-130909628:130910832-130913308	189	149	0.902	0.376	0.526	0.526	0	0
SE	chr6	Wnk1	-	119952685-119952969:119953771-119954232:119956071-119956193	298	149	0.834	0.311	0.523	0.523	0	0
SE	chr9	Zfp426	-	20473028-20473108:20474907-20475052:20476396-20476478	294	149	0.932	0.4117	0.52	0.52	1.36557E-14	1.89E-12
SE	chr10	Fam229b	-	39118804-39119034:39120258-39120396:39122177-39122332	287	149	0.917	0.3977	0.519	0.519	0	0
SE	chr15	Kif21a	-	90972625-90972715:90976471-90976509:90980795-90980998	187	149	0.7123	0.1943	0.518	0.518	0	0
SE	chr11	Tnrc6c	+	117739867-117740009:117741669-117741818:117742927-117743064	298	149	0.9103	0.3983	0.512	0.512	0	0
SE	chr9	Zfp426	-	20473028-20473108:20474907-20475075:20476396-20476478	298	149	0.9327	0.423	0.51	0.51	6.83897E-14	8.56E-12
SE	chr7	Ptpn5	-	47090421-47090504:47090727-47090834:47091377-47091570	256	149	0.9923	0.4907	0.502	0.502	0	0
SE	chr4	Sgip1	+	102860770-102860794:102863665-102863733:102867640-102867696	217	149	0.7323	0.2327	0.5	0.5	0	0
SE	chr9	Zfp317	+	19643609-19643704:19645225-19645307:19646702-19649731	231	149	1	0.5	0.5	0.5	0	0
A5SS	chr5	Guf1	+	69567128-69567261:69567128-69567490:69568396-69568497	298	149	0.7203	0.2217	0.499	0.499	0	0
SE	chr1	Adam23	+	63572887-63572998:63581137-63581227:63592608-63596104	239	149	0.6397	0.1417	0.498	0.498	2.22142E-11	1.79E-09
SE	chr5	Apbb2	-	66391772-66391857:66392273-66392335:66400076-66400281	211	149	0.7937	0.2977	0.496	0.496	4.75175E-14	6.04E-12
RI	chr1	Hnrnpu	-	178323620-178323713:178323620-178325313:178325243-178325313	298	149	0.8887	0.395	0.494	0.494	0	0
RI	chr4	Ccnl2	+	155818539-155819000:155818539-155820428:155819706-155820428	298	149	0.8973	0.4047	0.493	0.493	0	0
MXE	chr8	Arglu1	-	8666297-8667416:8681384-8681467:8683748-8683973:8689977-8690106	298	232	0.489	0	0.489	0.489	0	0
SE	chr12	Nrcam	+	44576604-44576789:44578145-44578180:44590108-44590239	184	149	0.9103	0.422	0.488	0.488	0	0
SE	chr4	Sgip1	+	102860770-102860794:102863662-102863733:102867640-102867696	220	149	0.8457	0.3573	0.488	0.488	0	0
SE	chr3	Pogz	+	94862422-94862530:94864088-94864378:94864485-94864667	298	149	0.9473	0.461	0.486	0.486	0	0
SE	chr2	Prrc2b	+	32226945-32227036:32229263-32229344:32230611-32234537	230	149	0.8307	0.3457	0.485	0.485	0	0
SE	chr5	Magi2	+	20651055-20651193:20661264-20661352:20702070-20702218	237	149	0.9127	0.431	0.482	0.482	0	0
MXE	chr5	Rnf32	+	29198574-29198838:29206696-29206804:29224117-29224284:29225043-292	257	298	0.1353	0.615	-0.48	0.48	7.11198E-12	1.23E-09
MXE	chr10	Fam229b	-	39118804-39119034:39120258-39120396:39122177-39122332:39132247-391	298	287	0.2387	0.718	-0.479	0.479	5.21805E-15	1.5E-12
SE	chr15	Prr5	+	84663171-84663261:84681200-84681319:84687903-84687983	268	149	0.8753	0.3983	0.477	0.477	5.06151E-13	5.45E-11
SE	chr14	Zdhhc20	-	57846921-57847062:57850145-57850180:57856610-57856730	184	149	0.747	0.271	0.476	0.476	0	0
A3SS	chr13	Pfkp	-	6597859-6598011:6588489-6596062:6588489-6588653	298	149	0.9093	0.434	0.475	0.475	0	0
SE	chr2	Fam171a1	+	3220253-3220369:3223470-3223584:3224818-3227806	263	149	0.738	0.2637	0.474	0.474	0	0
RI	chr4	Srrm1	-	135344499-135344669:135344499-135346445:135346316-135346445	298	149	0.93	0.462	0.468	0.468	0	0
SE	chr3	Zfp697	+	98382454-98382578:98410491-98411487:98425223-98425500	298	149	0.1383	0.6047	-0.466	0.466	4.79645E-06	0.00012
SE	chrX	Kdm6a	+	18246197-18246416:18247567-18247722:18248270-18248359	298	149	0.87	0.4057	0.464	0.464	9.3604E-09	4.62E-07
SE	chr14	Lmo7	+	101911999-101912160:101913159-101913346:101914277-101914337	298	149	1	0.5383	0.462	0.462	0	0
SE	chr1	Lrrfip1	+	91107298-91107390:91108580-91108672:91112156-91112297	241	149	0.7987	0.338	0.461	0.461	9.28913E-12	8.04E-10
SE	chr6	Hnrnpa2b1	-	51460930-51461433:51461983-51462055:51463411-51463493	221	149	0.5853	0.125	0.46	0.46	0	0
SE	chr2	Pfkfb3	-	11471430-11474543:11475754-11475806:11478036-11478058	201	149	0.6547	0.196	0.459	0.459	1.33008E-09	7.78E-08
SE	chr12	Nrcam	+	44576604-44576780:44578145-44578180:44590108-44590239	184	149	0.849	0.3937	0.455	0.455	0	0
SE	chr1	Pam	-	97821091-97821988:97822936-97822992:97825837-97826037	205	149	0.9623	0.509	0.453	0.453	0	0
SE	chr4	Unc13b	+	43232530-43232608:43233904-43233909:43234666-43234740	154	149	0.7437	0.2903	0.453	0.453	1.12127E-08	5.43E-07
MXE	chr17	Nrxn1	-	90162114-90162433:90163792-90163881:90208323-90208494:90560741-905	298	238	0.749	0.297	0.452	0.452	0	0
SE	chr8	Rbm34	-	126959542-126959585:126961912-126961971:126965382-126965622	208	149	0.6437	0.1913	0.452	0.452	1.15049E-10	8.12E-09
MXE	chr3	Lrrc7	-	158163892-158164072:158170556-158170756:158175103-158175351:158177	298	298	0.7597	0.309	0.451	0.451	2.93554E-10	3.42E-08
MXE	chr9	Scamp5	-	57446072-57446173:57451356-57451484:57451980-57452034:57467760-574	203	277	0.7883	0.337	0.451	0.451	8.63623E-10	8.78E-08
SE	chr18	Atp9b	-	80734141-80736149:80736812-80736844:80738653-80738856	181	149	0.772	0.3217	0.45	0.45	0	0
RI	chr3	Fubp1	+	152231877-152232022:152231877-152234570:152234524-152234570	298	149	0.9933	0.5443	0.449	0.449	0	0
SE	chr3	Zfp697	+	98382454-98382578:98410491-98411302:98425223-98425500	298	149	0.1413	0.5873	-0.446	0.446	1.67547E-05	0.000358
A5SS	chr9	Fam214a	+	75018997-75019155:75018997-75023746:75025651-75025847	298	149	1	0.5553	0.445	0.445	4.44089E-16	1.44E-13
SE	chr4	Pik3cd	-	149654816-149654959:149655127-149655257:149655336-149655500	279	149	0.9507	0.5077	0.443	0.443	3.32512E-13	3.73E-11
SE	chrX	Gprasp1	+	135742692-135742978:135743442-135743522:135744499-135744575	229	149	0.652	0.21	0.442	0.442	0	0
SE	chr7	Peg3	-	6718426-6718556:6725372-6725523:6726490-6726574	298	149	0.047	0.489	-0.442	0.442	0	0
SE	chr4	Trit1	+	123044491-123044636:123046875-123047017:123049477-123049554	291	149	0.9663	0.524	0.442	0.442	1.63203E-14	2.23E-12
SE	chr15	Oxr1	+	41825890-41826052:41848670-41848750:41850459-41850584	229	149	0.7527	0.3107	0.442	0.442	6.88227E-13	7.26E-11
SE	chr3	Nbea	-	55992334-55992514:55994048-55994056:56000487-56000640	157	149	0.6183	0.177	0.441	0.441	0	0
SE	chr15	Adgrb1	+	74574102-74574168:74577395-74577493:74580565-74580736	247	149	0.7253	0.2877	0.438	0.438	1.75231E-05	0.000372
SE	chr5	Fryl	-	73023888-73023998:73024771-73024788:73027316-73027384	166	149	0.6813	0.245	0.436	0.436	1.13243E-14	1.59E-12
SE	chr14	Ccser2	-	36885751-36885790:36890294-36890426:36896255-36896344	281	149	0.987	0.552	0.435	0.435	0	0
A3SS	chr7	Fuz	+	44897520-44897624:44898503-44898871:44898776-44898871	298	149	0.9037	0.4693	0.434	0.434	2.34973E-11	3.94E-09
RI	chr8	Myo9b	+	71348331-71348901:71348331-71349194:71348935-71349194	181	149	0.709	0.2747	0.434	0.434	2.10775E-06	3.01E-05
SE	chr16	Tmem44	-	30547343-30547469:30549393-30549457:30550363-30550570	213	149	0.682	0.2483	0.434	0.434	1.52866E-10	1.05E-08
SE	chr6	Cttnbp2	-	18405184-18405263:18406456-18406551:18408482-18408757	244	149	1	0.5683	0.432	0.432	0	0
SE	chr16	Tmem44	-	30547343-30547469:30549393-30549461:30550363-30550568	217	149	0.653	0.2207	0.432	0.432	7.53853E-12	6.62E-10
SE	chr7	Folr1	-	101863925-101864094:101868398-101868467:101869157-101869304	218	149	0.941	0.5123	0.429	0.429	0	0
SE	chr15	Scaf11	-	96415032-96415151:96415777-96415947:96416849-96417189	298	149	0.9927	0.564	0.429	0.429	0	0
SE	chr1	Mdm4	-	133003791-133003890:133004654-133004721:133009137-133009192	216	149	0.9087	0.4807	0.428	0.428	0	0
SE	chr4	Thap3	-	151982638-151983352:151983635-151983736:151985669-151985861	250	149	0.8847	0.4577	0.427	0.427	0	0
SE	chr6	Krba1	+	48395586-48395887:48402871-48403010:48403378-48403497	288	149	0.8763	0.4507	0.426	0.426	0	0
SE	chr6	Luc7l2	+	38551449-38551908:38565655-38565722:38568847-38568917	216	149	0.4723	0.8973	-0.425	0.425	0	0
A5SS	chr19	Hps1	-	42757793-42757906:42757212-42757906:42756258-42756340	298	149	0.7103	0.2883	0.422	0.422	5.98445E-07	2.51E-05
RI	chr7	Tial1	-	128446731-128447107:128446731-128448208:128448121-128448208	298	149	0.6757	0.2537	0.422	0.422	0	0
SE	chr5	Rnf32	+	29206696-29206804:29224117-29224284:29225043-29225524	298	149	0.8527	0.431	0.422	0.422	1.85654E-11	1.52E-09
SE	chr6	Krba1	+	48395697-48395887:48402884-48403010:48403378-48403497	275	149	0.847	0.4253	0.422	0.422	1.44329E-15	2.24E-13
MXE	chr17	Ptprs	-	56426387-56426455:56427939-56428132:56428852-56428966:56434453-564	263	298	0.9633	0.5427	0.421	0.421	0	0
SE	chr2	Nudt5	+	5864347-5864448:5866233-5866241:5866354-5866407	157	149	0.94	0.5187	0.421	0.421	5.45293E-11	4.09E-09
SE	chr12	Pxdn	+	29999165-29999273:29999499-29999693:29999892-30000049	298	149	0.527	0.1063	0.421	0.421	6.54729E-10	4.05E-08
RI	chr1	Clk1	-	58419667-58419733:58419667-58421308:58421083-58421308	298	149	0.8297	0.412	0.418	0.418	4.44089E-16	3.09E-14
SE	chr15	Ly6h	-	75566132-75566259:75566763-75566831:75567095-75567185	217	149	0.9437	0.528	0.416	0.416	0	0
SE	chr14	Tdrd3	+	87457034-87457118:87458761-87458822:87472076-87472236	210	149	0.958	0.543	0.415	0.415	9.84768E-14	1.19E-11
SE	chr16	Pam16	-	4617126-4617262:4617919-4618150:4624851-4624942	298	149	0.8363	0.4213	0.415	0.415	0	0
SE	chr3	Postn	+	54377561-54377606:54380412-54380492:54383388-54383477	229	149	0.8827	0.4683	0.414	0.414	1.27454E-11	1.08E-09
SE	chr16	Grik1	-	87895897-87896351:87896809-87896895:87914154-87914404	235	149	0.4917	0.0803	0.411	0.411	0	0
SE	chr11	Clk4	+	51268769-51268846:51270537-51270595:51271800-51272022	207	149	0.9073	0.496	0.411	0.411	2.62013E-08	1.16E-06
SE	chr9	Stac	-	111606821-111606921:111634806-111635052:111689998-111690590	298	149	0.377	0.788	-0.411	0.411	1.93622E-06	5.46E-05
SE	chr8	Dus2	+	106030352-106030443:106031698-106031741:106033723-106033783	192	149	0.8417	0.4327	0.409	0.409	3.96952E-08	1.69E-06
A5SS	chr19	Hps1	-	42757793-42757906:42757665-42757906:42756258-42756340	276	149	0.6873	0.2797	0.408	0.408	4.6323E-06	0.000137
SE	chr17	Nrxn1	-	90162114-90162433:90163792-90163881:90208323-90208494	238	149	0.3617	0.7697	-0.408	0.408	0	0
SE	chr1	Ppip5k2	-	97713004-97713144:97717442-97717561:97719834-97719906	268	149	0.722	0.3143	0.408	0.408	6.66134E-16	1.08E-13
SE	chr14	Camk2g	-	20744604-20744648:20747819-20747851:20755659-20755702	181	149	0.7823	0.375	0.407	0.407	0	0
SE	chr6	Krba1	+	48395663-48395887:48401114-48403010:48403378-48403497	298	149	0.8357	0.431	0.405	0.405	2.08833E-13	2.42E-11
MXE	chr9	Scamp5	-	57446072-57446173:57451356-57451484:57451980-57452034:57467816-574	203	277	0.8413	0.437	0.404	0.404	1.2361E-11	1.94E-09
SE	chr3	Lrrc7	-	158170556-158170756:158175103-158175351:158177209-158177333	298	149	0.6673	0.263	0.404	0.404	4.34256E-08	1.83E-06
SE	chr16	Kalrn	-	34039879-34040009:34049830-34049922:34053560-34053661	241	149	0.7653	0.3617	0.404	0.404	5.55112E-16	9.03E-14
SE	chr5	Rnf32	+	29198574-29198838:29203055-29203197:29205669-29205701	291	149	0.834	0.43	0.404	0.404	2.88901E-08	1.27E-06
SE	chr5	43719	+	93167500-93167687:93171339-93173537:93176040-93176462	298	149	0.6967	0.2957	0.401	0.401	0	0
SE	chr10	Dip2a	-	76270396-76270457:76271376-76271405:76272533-76272703	178	149	0.6553	0.2547	0.401	0.401	1.75193E-13	2.06E-11
RI	chr5	Rad9b	-	122323223-122324224:122323223-122325464:122325351-122325464	298	149	0.799	0.3993	0.4	0.4	8.19955E-08	1.71E-06
A3SS	chr10	Dot1l	+	80790611-80791561:80791744-80795461:80792143-80795461	298	149	0.903	0.5037	0.399	0.399	5.85842E-12	1.08E-09
SE	chr18	Paip2	+	35598606-35598759:35600045-35600117:35613323-35613502	221	149	0.2283	0.6277	-0.399	0.399	1.18272E-06	3.54E-05
MXE	chr9	Abhd14a	-	106440621-106440856:106443806-106443942:106445526-106445829:106447	298	285	0.7257	0.328	0.398	0.398	0.002320003	0.029184
RI	chr9	Taf1d	+	15311527-15311695:15311527-15314032:15313990-15314032	298	149	0.9617	0.5647	0.397	0.397	0	0
SE	chr16	Dnm1l	-	16341422-16341468:16342697-16342735:16345998-16346145	187	149	0.3307	0.7277	-0.397	0.397	0	0

SE	chr1	Lrrfip1	+	91107298-91107390:91108580-91108672:91110355-91110447	241	149	0.6663	0.2707	0.396	0.396	2.4325E-13	2.79E-11
SE	chr9	Zfp558	-	18468614-18468702:18469279-18469396:18472886-18472994	266	149	0.8007	0.4057	0.395	0.395	0	0
SE	chr9	Dixdc1	-	50682033-50682200:50682537-50682626:50683725-50683796	238	149	0.8363	0.4417	0.395	0.395	1.82487E-08	8.31E-07
SE	chr4	Ccnl2	+	155817921-155817987:155818539-155818552:155820329-155820417	162	149	0.3243	0.7197	-0.395	0.395	1.1764E-11	9.98E-10
SE	chr4	Mpdz	-	81356330-81356497:81358626-81358742:81360004-81360213	265	149	0.771	0.3773	0.394	0.394	4.14772E-08	1.75E-06
SE	chr11	Bptf	-	107043633-107043825:107044492-107044665:107047000-107047343	298	149	0.8603	0.4673	0.393	0.393	0	0
SE	chr7	Fuz	+	44897520-44897624:44898503-44898700:44898776-44898871	298	149	0.94	0.5477	0.392	0.392	6.66134E-16	1.08E-13
SE	chr10	Ahi1	+	21058153-21058250:21070288-21070543:21072543-21072601	298	149	0.5353	0.1447	0.391	0.391	0	0
SE	chr13	Tbce	-	14005143-14005283:14005788-14005852:14005943-14006007	213	149	0.9873	0.5977	0.39	0.39	0	0
A5SS	chr9	Trank1	+	111373408-111373545:111373408-111374925:111377778-111377921	298	149	0.919	0.53	0.389	0.389	4.65183E-14	9.84E-12
MXE	chr12	Meg3	+	109544027-109544742:109544948-109545041:109549291-109549371:10954[illegible]	242	229	0.3153	0.7047	-0.389	0.389	0	0
SE	chr11	Cobl	-	12266856-12267144:12306958-12307128:12309614-12309752	298	149	0.809	0.42	0.389	0.389	2.31411E-06	6.35E-05
SE	chr15	Mapk15	+	75998059-75998263:75998371-75998474:75998550-75998678	252	149	1	0.6107	0.389	0.389	3.05311E-13	3.43E-11
SE	chr7	Eri2	-	119786739-119786827:119787404-119787485:119787734-119787834	230	149	0.9887	0.6007	0.388	0.388	1.02141E-14	1.45E-12
SE	chr4	Ptprd	-	76138556-76138573:76139294-76139302:76140514-76140702	157	149	0.6797	0.2913	0.388	0.388	2.87548E-14	3.78E-12
SE	chr16	Alg3	-	20607764-20607884:20608486-20608646:20608742-20608889	298	149	0.9033	0.515	0.388	0.388	2.78064E-10	1.84E-08
SE	chr13	Slf1	-	77091185-77091343:77092358-77092411:77100922-77101056	202	149	0.988	0.601	0.387	0.387	1.45439E-14	2E-12
MXE	chr5	Zfp644	-	106619540-106619642:106635610-106638635:106690730-106690877:10669[illegible]	296	298	0.0797	0.4653	-0.386	0.386	2.96167E-10	3.42E-08
SE	chr10	Jmjd1c	+	67215270-67215375:67216983-67217107:67218038-67218181	273	149	0.801	0.4163	0.385	0.385	1.6997E-09	9.73E-08
SE	chr19	Rbm4b	+	4760013-4762647:4764350-4764571:4765263-4765940	298	149	0.9157	0.532	0.384	0.384	6.81725E-07	2.18E-05
A5SS	chr8	Whsc1l1	+	25706524-25706607:25706524-25706640:25709063-25709204	181	149	0.749	0.3663	0.383	0.383	4.17174E-09	3.27E-07
SE	chr9	Rbm5	-	107749708-107749799:107750008-107750092:107750463-107750548	233	149	0.909	0.5273	0.382	0.382	0	0
SE	chr11	Usp34	+	23361587-23361716:23362577-23362612:23363058-23363188	184	149	0.5153	0.134	0.381	0.381	3.33067E-16	5.56E-14
SE	chr7	Ttc23	+	67647319-67647845:67662327-67662526:67667163-67667286	298	149	0.406	0.7863	-0.38	0.38	0.000451734	0.006085
SE	chr10	Ptprk	+	28551618-28551756:28554770-28554799:28560005-28560165	178	149	0.7227	0.3437	0.379	0.379	0	0
SE	chr16	Synj1	-	90936092-90939034:90940473-90940496:90944355-90944575	172	149	0.8073	0.4303	0.377	0.377	1.59571E-09	9.16E-08
SE	chr11	Aftph	-	20692465-20692588:20696751-20696921:20698313-20698373	298	149	0.6537	0.2763	0.377	0.377	8.13318E-11	5.85E-09
RI	chr17	Mdc1	+	35850295-35851005:35850295-35851156:35851113-35851156	255	149	0.267	0.6423	-0.375	0.375	2.20542E-08	4.84E-07
SE	chr2	Oprl1	+	181715690-181715946:181716502-181716535:181718377-181718732	182	149	0.2117	0.586	-0.374	0.374	0	0
SE	chr2	Kcnt1	+	25898699-25898835:25900158-25900286:25900372-25900480	277	149	0.8663	0.4927	0.374	0.374	7.54053E-06	0.000179
SE	chr10	Mdm2	-	117705155-117705229:117709700-117709784:117709946-117710028	233	149	0.6947	0.3203	0.374	0.374	0	0
A5SS	chr3	Gm3764	+	88227366-88227624:88227366-88228700:88229012-88229365	298	149	0.7157	0.3423	0.373	0.373	0	0
A3SS	chrX	Tceal3	+	136594202-136594250:136667060-136667190:136667124-136667190	212	149	0.273	0.645	-0.372	0.372	2.01148E-07	1.31E-05
RI	chr7	Zfp606	+	12480935-12481026:12480935-12481181:12481118-12481181	239	149	0.8167	0.4447	0.372	0.372	3.64938E-05	0.000362
A5SS	chr4	Usp48	+	137617982-137619974:137617982-137620377:137621062-137621259	298	149	0.87	0.4993	0.371	0.371	3.69371E-13	6.91E-11
SE	chr15	Scaf11	-	96415032-96415151:96415729-96415947:96416849-96417189	298	149	0.994	0.623	0.371	0.371	0	0
SE	chr14	Styx	+	45354946-45354978:45356713-45356766:45359259-45359356	202	149	0.9633	0.5923	0.371	0.371	0	0
RI	chr19	Hps1	-	42756258-42756340:42756258-42757906:42757793-42757906	298	149	0.79	0.42	0.37	0.37	1.38818E-07	2.65E-06
SE	chr4	Lrp8	+	107868978-107869150:107870012-107870188:107872273-107872373	298	149	0.672	0.3017	0.37	0.37	0	0
SE	chr5	Ddx55	+	124559128-124559317:124560718-124560800:124561856-124562037	231	149	0.989	0.6203	0.369	0.369	1.22125E-15	1.92E-13
SE	chr3	Pde7a	-	19229688-19229768:19230256-19230352:19230957-19231101	245	149	0.946	0.5783	0.368	0.368	0	0
SE	chr9	Clasp2	+	113858581-113858604:113860120-113860137:113862186-113862287	166	149	0.966	0.5983	0.368	0.368	0	0
SE	chr5	Zfp644	-	106666785-106666845:106690730-106690877:106696234-106696821	296	149	0.096	0.4643	-0.368	0.368	7.8577E-06	0.000186
SE	chr17	Birc6	+	74593089-74593249:74594322-74594453:74594870-74595001	280	149	0.9023	0.535	0.367	0.367	1.44452E-05	0.000315
SE	chr14	Kcnma1	-	23441993-23442079:23449200-23449211:23451599-23451667	160	149	0.968	0.601	0.367	0.367	1.18633E-08	5.7E-07
SE	chr15	Oxr1	+	41825890-41826052:41848670-41849506:41850459-41850584	298	149	0.5027	0.136	0.367	0.367	1.63531E-08	7.56E-07
SE	chr8	Adgrl1	+	83923019-83923108:83924206-83924355:83929464-83930264	298	149	0.074	0.4403	-0.366	0.366	0	0
SE	chr11	Tnip1	-	54939598-54939735:54940711-54940882:54956021-54956121	298	149	0.4423	0.0767	0.366	0.366	6.18176E-08	2.51E-06
A3SS	chr11	Baiap2	+	120000552-120000586:120003038-120006782:120006359-120006782	298	149	0.633	0.268	0.365	0.365	1.84728E-07	1.25E-05
A5SS	chr4	Pik3cd	-	149655336-149655500:149655127-149655500:149654816-149654959	298	149	0.965	0.6	0.365	0.365	4.55191E-15	1.23E-12
RI	chr12	Inf2	+	112612548-112612604:112612548-112615557:112614895-112615557	298	149	0.921	0.556	0.365	0.365	4.32865E-07	7.27E-06
RI	chr9	Yipf2	-	21588682-21589558:21588682-21589760:21589635-21589760	224	149	0.792	0.4277	0.364	0.364	4.66456E-10	1.41E-08
SE	chr10	Os9	-	127096630-127096742:127096915-127097079:127097932-127098121	298	149	0.2627	0.627	-0.364	0.364	0	0
SE	chr7	Wdr73	-	80897923-80897987:80898503-80898591:80900348-80900436	237	149	0.8047	0.4427	0.362	0.362	0	0
SE	chr11	Gjc1	-	102802841-102802916:102804281-102804512:102819504-102819686	298	149	0.2387	0.6003	-0.362	0.362	0.000167671	0.002597
A5SS	chr12	Rnaseh1	+	28657637-28657717:28657637-28658295:28658993-28659592	298	149	0.876	0.5147	0.361	0.361	2.00114E-09	1.74E-07
MXE	chr4	Ptprd	-	76594267-76594305:76765700-76765758:76956896-76956995:76981506-769	248	207	0.0603	0.4213	-0.361	0.361	0	0
SE	chr11	Socs7	+	97376437-97376541:97376997-97377686:97377936-97378066	298	149	0.933	0.572	0.361	0.361	6.57296E-12	5.83E-10
A5SS	chr3	Gm3764	+	88227366-88227624:88227366-88228694:88229012-88229365	298	149	0.7087	0.3483	0.36	0.36	0	0
SE	chr11	Bzrap1	+	87780882-87780953:87781302-87781370:87781490-87781593	217	149	0.8787	0.519	0.36	0.36	2.61569E-13	2.98E-11
RI	chr17	Syngap1	+	26966883-26966973:26966883-26968799:26967681-26968799	298	149	0.8397	0.481	0.359	0.359	5.10414E-11	1.8E-09
SE	chr4	Trnau1ap	-	132325208-132325260:132325352-132325489:132328469-132328566	286	149	0.6053	0.2467	0.359	0.359	1.82843E-07	6.66E-06
SE	chr7	Tial1	-	128446195-128446270:128446731-128447107:128448121-128448208	298	149	0.4307	0.7897	-0.359	0.359	1.74305E-14	2.36E-12
SE	chr5	Atxn2	+	121802948-121803080:121806222-121806275:121810851-121810996	202	149	0.6863	0.3277	0.359	0.359	0	0
RI	chr6	Tia1	+	86419101-86419133:86419101-86420411:86420324-86420411	298	149	0.8017	0.445	0.357	0.357	0	0
SE	chr9	Cish	+	107296659-107297034:107299924-107300144:107300368-107302784	298	149	0.5597	0.203	0.357	0.357	7.5123E-08	2.98E-06
SE	chr13	Ipo11	-	106857229-106857308:106857499-106857525:106860861-106860937	175	149	0.665	0.3077	0.357	0.357	2.18591E-06	6.05E-05
SE	chrX	Fhl1	+	56790464-56790650:56791239-56791438:56791844-56793346	298	149	0.7347	0.3773	0.357	0.357	0	0
SE	chr5	Zfp655	+	145235306-145235391:145235737-145235863:145237050-145238322	275	149	0.9787	0.623	0.356	0.356	6.84488E-11	5.01E-09
A3SS	chr4	Dock7	-	98955316-98955447:98946629-98946763:98946629-98946754	157	149	0.9567	0.602	0.355	0.355	1.6831E-13	4.05E-11
A5SS	chr6	Tia1	+	86419101-86419133:86419101-86419812:86420324-86420411	298	149	0.6527	0.298	0.355	0.355	0	0
SE	chr5	Clip1	-	123583521-123583638:123586334-123586354:123590793-123590845	169	149	0.5657	0.2107	0.355	0.355	0	0
SE	chr7	Xntrpc	+	102078733-102078818:102079005-102079177:102080529-102080604	298	149	0.9077	0.5523	0.355	0.355	3.97977E-07	1.34E-05
SE	chr11	Sez6	+	77976744-77976941:77977245-77977283:77977537-77977654	187	149	0.7627	0.409	0.354	0.354	0	0
SE	chr1	Adam23	+	63572887-63572998:63585365-63585455:63592608-63596101	239	149	0.6427	0.289	0.354	0.354	2.59548E-07	9.1E-06
SE	chr4	Lrp8	+	107843216-107843344:107846239-107846619:107847441-107847566	298	149	0.4717	0.826	-0.354	0.354	5.3088E-11	4E-09
SE	chr2	4930402H24Rik	-	130777205-130777281:130778417-130778501:130784424-130784460	233	149	0.9493	0.5957	0.354	0.354	1.43082E-08	6.74E-07
SE	chr11	Hexdc	+	121220864-121221161:121221236-121221337:121221501-121221590	250	149	0.8667	0.5123	0.354	0.354	3.66374E-15	5.54E-13
A3SS	chr15	Csad	-	102178961-102179160:102178527-102178832:102178527-102178616	298	149	0.911	0.5577	0.353	0.353	2.91978E-09	3.24E-07
SE	chr16	Robo2	-	73928024-73928177:73933046-73933171:73933587-73933868	274	149	0.7633	0.4107	0.353	0.353	0	0
SE	chr13	Kdm1b	+	47053619-47053735:47057677-47057697:47058776-47058814	169	149	0.601	0.2483	0.353	0.353	5.41029E-08	2.22E-06
RI	chr11	Hexdc	+	121221083-121221161:121221083-121221590:121221501-121221590	298	149	0.855	0.503	0.352	0.352	3.475E-14	2.02E-12
SE	chr13	Zfp369	+	65294396-65294484:65295600-65295726:65296063-65297795	275	149	0.862	0.5097	0.352	0.352	4.82857E-07	1.59E-05
A3SS	chr2	Fnbp4	+	90767408-90767541:90767853-90768700:90768516-90768700	298	149	0.684	0.3327	0.351	0.351	2.88658E-15	8.68E-13
SE	chr1	Dusp12	-	170880130-170880226:170880573-170880691:170885067-170885442	267	149	0.907	0.5557	0.351	0.351	1.30641E-08	6.22E-07
SE	chr12	Trmt5	-	73280003-73280632:73281005-73281656:73282612-73282736	298	149	0.9763	0.625	0.351	0.351	1.96065E-12	1.93E-10
SE	chr8	Cyld	+	88709891-88709996:88718290-88718298:88719298-88719396	157	149	0.5153	0.1647	0.351	0.351	1.95988E-07	7.09E-06
RI	chr2	Oprl1	+	181716502-181716535:181716502-181718093:181717955-181718093	298	149	0.849	0.4987	0.35	0.35	2.19851E-07	3.93E-06
SE	chr13	Zfp738	-	67667441-67671611:67672998-67673093:67673562-67673688	244	149	0.8333	0.4843	0.349	0.349	1.97766E-06	5.56E-05
SE	chr14	Mycbp2	-	103155124-103156767:103169857-103170036:103172567-103172689	298	149	0.7563	0.4073	0.349	0.349	2.63828E-10	1.75E-08
SE	chr9	Zfp266	-	20505977-20506065:20506728-20507723:20508019-20508185	298	149	0.8393	0.4907	0.349	0.349	6.77308E-11	4.97E-09
SE	chr5	Preb	-	30951667-30955737:30956262-30956334:30957888-30958061	221	149	0.8763	0.5277	0.349	0.349	1.79256E-08	8.17E-07
SE	chr14	Chd8	-	52201631-52201747:52202144-52202323:52202504-52202617	298	149	0.976	0.6267	0.349	0.349	0	0
SE	chr12	Rnaseh1	+	28657633-28657717:28658171-28658295:28658993-28659591	273	149	0.9073	0.5597	0.348	0.348	1.88277E-11	1.54E-09
SE	chr1	Sgk3	+	9881578-9881661:9881760-9881891:9884407-9884519	280	149	1	0.6523	0.348	0.348	1.38775E-10	9.62E-09
RI	chr3	Gm3764	+	88227366-88227624:88227366-88229365:88229012-88229365	298	149	0.8187	0.4713	0.347	0.347	0	0
RI	chr17	Luc7l	+	26253974-26254044:26253974-26255125:26255031-26255125	298	149	0.605	0.258	0.347	0.347	0	0
SE	chr2	Sh3glb2	-	30347081-30347104:30349226-30349237:30350288-30350380	160	149	0.4403	0.7877	-0.347	0.347	1.28003E-07	4.82E-06
A5SS	chr2	Fnbp4	+	90767408-90767541:90767408-90767955:90768516-90768700	298	149	0.651	0.3053	0.346	0.346	3.33067E-16	1.16E-13
SE	chr9	Fat3	-	15910189-15915621:15916204-15916239:15916936-15916995	184	149	1	0.6543	0.346	0.346	2.29805E-12	2.23E-10
SE	chr5	Ddx55	+	124559128-124559317:124560718-124560800:124561906-124562037	231	149	0.9323	0.5867	0.346	0.346	2.22045E-16	3.82E-14
A5SS	chrX	Thoc2	-	41813950-41814008:41813230-41814008:41812459-41812575	298	149	0.3493	0.6943	-0.345	0.345	1.11627E-05	0.000283
MXE	chr3	Syt6	+	103620786-103620906:103626982-103627132:103630902-103630969:10363[illegible]	298	216	0.1103	0.4557	-0.345	0.345	1.19205E-12	2.42E-10
SE	chr9	Zfp426	-	20473028-20473108:20474907-20475033:20476396-20476478	275	149	0.972	0.627	0.345	0.345	0	0
SE	chr4	Ccnl2	+	155817923-155817987:155818539-155819000:155820329-155820428	298	149	0.5683	0.913	-0.345	0.345	0	0
A3SS	chr17	Srrm2	+	23811639-23811731:23811817-23812620:23812428-23812620	298	149	0.482	0.1377	0.344	0.344	0	0
SE	chr11	Aftph	-	20692465-20692588:20696751-20696831:20698313-20698373	229	149	0.8157	0.4717	0.344	0.344	3.33067E-16	5.56E-14
SE	chr7	Prkcg	+	3314512-3314541:3318860-3319012:3319531-3319719	298	149	0.8513	0.5073	0.344	0.344	2.85922E-07	9.94E-06
SE	chr9	Tcf12	-	71868072-71868278:71868999-71869070:71870074-71870147	220	149	0.6937	0.35	0.344	0.344	8.54872E-15	1.23E-12
SE	chr11	1810032O08Rik	+	116671660-116671756:116672164-116672355:116672664-116672738	298	149	0.9183	0.574	0.344	0.344	0	0
A3SS	chr5	43719	+	93173473-93173537:93175404-93176462:93176040-93176462	298	149	0.868	0.5247	0.343	0.343	2.43887E-06	0.000109
A5SS	chr1	Mfsd6	-	52660807-52661087:52658699-52661087:52656299-52658545	298	149	0.928	0.5853	0.343	0.343	1.43774E-12	2.59E-10
MXE	chr12	Meg3	+	109544948-109545041:109546430-109546542:109549479-109549552:10954[illegible]	261	222	0.1867	0.53	-0.343	0.343	0	0
SE	chr1	Eif2d	+	131163520-131163565:131164403-131164506:131165120-131165209	252	149	0.9433	0.6003	0.343	0.343	2.23244E-11	1.8E-09
SE	chr19	Naa40	-	7229722-7229805:7229948-7230106:7230188-7230283	298	149	0.8683	0.525	0.343	0.343	2.88979E-10	1.9E-08
MXE	chr7	Usp29	+	6730560-6731302:6738807-6738945:6908286-6908385:6911852-6912023	287	248	0.8873	0.5457	0.342	0.342	3.78516E-08	2.67E-06
RI	chr11	Xpo1	+	23267662-23267763:23267662-23268912:23268849-23268912	298	149	0.6993	0.357	0.342	0.342	1.42144E-07	2.7E-06
SE	chr3	Ttc14	+	33803075-33803204:33803450-33803605:33804000-33804071	298	149	0.701	0.3593	0.342	0.342	1.11022E-16	1.97E-14
SE	chr15	Rims2	+	39462498-39462610:39469155-39469202:39472334-39472464	196	149	0.401	0.06	0.341	0.341	2.97762E-13	3.35E-11
SE	chr10	Grik2	-	49103567-49103648:49104979-49105065:49113370-49113620	235	149	0.354	0.6947	-0.341	0.341	6.12626E-05	0.001098
RI	chr6	Luc7l2	+	38568847-38568917:38568847-38570604:38570510-38570604	298	149	0.918	0.5783	0.34	0.34	0	0
SE	chr11	Fmnl1	+	103197658-103197778:103198135-103198233:103198483-103198590	247	149	0.5413	0.201	0.34	0.34	6.11913E-06	0.000149
SE	chr4	Asph	-	9635901-9635969:9637659-9637703:9639198-9639347	193	149	0.7647	0.426	0.339	0.339	0	0
SE	chr9	Snx14	-	88398239-88398371:88400723-88400749:88401920-88401978	175	149	0.8183	0.479	0.339	0.339	6.69733E-09	3.42E-07

SE	chr7	Prss36	-	127933371-127933492:127933567-127933832:127934390-127934623	298	149	0.4143	0.7533	-0.339	0.339	6.19764E-05	0.001107
SE	chrX	Enox2	-	49009707-49011634:49013133-49013180:49013497-49013582	196	149	0.6637	0.3247	0.339	0.339	6.2904E-05	0.00112
SE	chr16	Clec16a	+	10571494-10571599:10572918-10572923:10573300-10573423	154	149	0.9067	0.5683	0.338	0.338	4.28435E-13	4.67E-11
SE	chr8	Tcf25	+	123400625-123400697:123401076-123401189:123403150-123403815	262	149	0.0753	0.4133	-0.338	0.338	0	0
SE	chr10	Tpd52l1	-	31332373-31332969:31341140-31341178:31343012-31343026	187	149	0.9093	0.5713	0.338	0.338	4.67381E-07	1.54E-05
SE	chr9	Zfp317	+	19625500-19625637:19625735-19625995:19629097-19629206	298	149	0.6623	0.324	0.338	0.338	0.000178364	0.00274
SE	chr13	Agtpbp1	-	59528387-59528518:59531057-59531203:59532013-59532076	295	149	0.9063	0.5697	0.337	0.337	0	0
SE	chr15	Kansl2	-	98520318-98520425:98521636-98521762:98528863-98529029	275	149	0.7683	0.4317	0.337	0.337	0.000125239	0.00202
SE	chr4	Ptprd	-	76765700-76765758:76956896-76956995:76981506-76981539	248	149	0.1177	0.4543	-0.337	0.337	1.11022E-16	1.97E-14
SE	chr5	Tbc1d19	+	53837924-53837970:53838177-53838237:53846201-53846273	209	149	0.9797	0.6427	0.337	0.337	2.69862E-12	2.57E-10
SE	chr2	Rapgef4	+	72141153-72141172:72174423-72174476:72174802-72174908	202	149	0.6453	0.308	0.337	0.337	5.27185E-08	2.17E-06
A3SS	chr9	Dopey1	+	86500796-86500895:86501874-86501983:86501901-86501983	175	149	0.1717	0.508	-0.336	0.336	2.25676E-07	1.45E-05
SE	chr15	Csad	-	102178527-102178616:102178781-102178832:102178961-102179160	200	149	0.8917	0.5553	0.336	0.336	6.59907E-07	2.12E-05
SE	chr10	Mmp11	-	75927129-75927262:75927347-75927490:75928280-75928509	292	149	0.912	0.5757	0.336	0.336	4.06231E-13	4.46E-11
MXE	chr11	Clk4	+	51268165-51268231:51268769-51268846:51270537-51270595:51271800-512	226	207	0.218	0.553	-0.335	0.335	8.74801E-07	3.98E-05
SE	chr16	Trap1	-	4054754-4054909:4055797-4055870:4062330-4062401	222	149	0.9827	0.6473	0.335	0.335	0	0
SE	chr14	Erc2	+	28302876-28303010:28383824-28384043:28475603-28478537	298	149	0.1707	0.5053	-0.335	0.335	6.27276E-14	7.88E-12
MXE	chr15	Ncald	-	37508876-37508994:37563391-37563464:37661032-37661086:37780136-377	203	222	0.7703	0.4363	0.334	0.334	7.97034E-06	0.000266
MXE	chr6	Krba1	+	48395597-48395887:48403378-48403497:48403991-48404140:48405234-484	268	298	0.0697	0.404	-0.334	0.334	0	0
SE	chr1	Ncoa2	-	13162073-13162288:13164896-13165102:13166896-13167106	298	149	0.8587	0.5253	0.333	0.333	2.16001E-09	1.22E-07
SE	chr9	Cadm1	+	47829377-47829409:47836717-47836770:47848167-47848298	202	149	0.448	0.1147	0.333	0.333	0	0
A3SS	chr1	Slco5a1	-	12943980-12944112:12938989-12939200:12938989-12939127	221	149	0.971	0.6393	0.332	0.332	3.31948E-11	5.32E-09
MXE	chr17	AI413582	-	27564149-27564254:27564385-27564410:27564562-27564783:27565660-275	298	174	0.0527	0.385	-0.332	0.332	1.02508E-11	1.68E-09
SE	chr11	Tanc2	+	105913357-105913489:105914041-105914070:105914892-105914983	178	149	0.6163	0.284	0.332	0.332	3.68977E-09	2.01E-07
SE	chr17	Bag6	+	35141830-35142082:35142498-35142605:35142727-35142839	256	149	0.9543	0.622	0.332	0.332	2.93326E-10	1.93E-08
SE	chr5	Kmt2c	-	25293107-25293248:25295398-25295610:25298870-25300576	298	149	0.3353	0.666	-0.331	0.331	6.40101E-07	2.06E-05
SE	chr19	Hectd2	+	36599596-36599719:36601432-36601528:36604250-36604357	245	149	0.9307	0.5997	0.331	0.331	8.43073E-08	3.3E-06
SE	chr8	Ints10	+	68822157-68822250:68825052-68825129:68827277-68828643	226	149	0.725	0.3943	0.331	0.331	1.60257E-11	1.33E-09
SE	chr5	Crlf2	-	109555273-109555357:109555507-109555660:109556019-109556181	298	149	0.811	0.4797	0.331	0.331	3.52128E-08	1.51E-06
A3SS	chr3	Camk2d	+	126767514-126767579:126770785-126771846:126771774-126771846	298	149	0.9193	0.5893	0.33	0.33	1.14773E-10	1.66E-08
SE	chr19	Kcnip2	-	45795580-45795704:45797091-45797186:45815731-45815815	244	149	0.6857	0.3557	0.33	0.33	2.6357E-05	0.000534
SE	chr16	Ttc3	+	94403314-94403368:94410833-94410956:94415949-94416009	272	149	0.1223	0.4527	-0.33	0.33	0	0
A3SS	chr13	Pfkp	-	6598739-6598826:6588489-6596062:6588489-6588653	298	149	0.974	0.645	0.329	0.329	0	0
A3SS	chr15	Wdyhv1	+	58150606-58150656:58150982-58152745:58152597-58152745	298	149	0.5723	0.243	0.329	0.329	0.000149191	0.003343
SE	chr2	Cacnb4	-	52465598-52465678:52465894-52465913:52467244-52467320	168	149	0.991	0.6623	0.329	0.329	1.11022E-16	1.97E-14
SE	chr11	Trim41	-	48806403-48807846:48808465-48808487:48809050-48809280	171	149	0.945	0.616	0.329	0.329	1.75686E-09	1E-07
SE	chr14	Lrch1	-	74786281-74786390:74793127-74793231:74795297-74795369	253	149	0.668	0.3393	0.329	0.329	7.4979E-05	0.001303
MXE	chr15	Micall1	+	79108699-79109429:79115030-79115171:79119736-79119827:79120759-791	290	240	0.077	0.4053	-0.328	0.328	5.61987E-09	4.53E-07
RI	chr1	Cntn2	-	132509427-132510793:132509427-132515637:132511315-132515637	298	149	0.837	0.5093	0.328	0.328	4.78171E-07	7.98E-06
SE	chr12	Nrxn3	+	90283572-90283650:90321936-90322641:90331485-90334935	298	149	0.9903	0.6623	0.328	0.328	1.35447E-13	1.61E-11
SE	chr2	Ralgapa2	-	146412734-146412868:146418362-146418502:146421593-146421757	289	149	0.6973	0.3697	0.328	0.328	0.000152037	0.002385
SE	chr2	Vav2	-	27299374-27299472:27300430-27300444:27302008-27302110	163	149	0.9503	0.6223	0.328	0.328	0	0
A3SS	chr13	Pfkp	-	6598739-6598826:6597859-6598040:6597859-6598011	177	149	0.4997	0.1727	0.327	0.327	4.44089E-16	1.6E-13
SE	chr2	Vav2	-	27267230-27267384:27268151-27268237:27268899-27269022	235	149	0.854	0.5267	0.327	0.327	3.01981E-14	3.95E-12
A5SS	chr8	Trmt1	+	84689247-84689349:84689247-84689376:84689632-84689908	175	149	0.3383	0.664	-0.326	0.326	0.002764686	0.025533
SE	chr3	Gria2	-	80682904-80689351:80690404-80690518:80691320-80691434	263	149	0.7433	0.4177	0.326	0.326	0	0
SE	chr4	Trit1	+	123044491-123044636:123049152-123049264:123049477-123049554	261	149	0.9713	0.6457	0.326	0.326	2.53715E-11	2.02E-09
A3SS	chr3	Gm3764	+	88209635-88209716:88220079-88221050:88220956-88221050	298	149	0.7567	0.4317	0.325	0.325	2.51567E-05	0.000807
MXE	chr8	Adgrl1	+	83923019-83923108:83924206-83924355:83928298-83928382:83929464-839	298	233	0.32	0.644	-0.324	0.324	2.0685E-09	1.86E-07
RI	chr8	Pkn1	-	83671299-83671441:83671299-83671969:83671907-83671969	298	149	0.6433	0.3193	0.324	0.324	1.16338E-09	3.3E-08
SE	chr4	Pik3cd	-	149654816-149654959:149655127-149655248:149655336-149655500	270	149	0.9687	0.645	0.324	0.324	3.24185E-14	4.22E-12
SE	chr9	Taf1d	+	15311527-15311695:15312774-15312836:15313990-15314032	211	149	0.9513	0.6273	0.324	0.324	1.91847E-13	2.23E-11
SE	chr11	Hdac5	-	102218416-102218490:102221768-102221851:102224734-102224955	232	149	0.0173	0.3417	-0.324	0.324	0	0
SE	chr10	Ormdl2	-	128819954-128820105:128820261-128820514:128821542-128821631	298	149	0.95	0.626	0.324	0.324	4.74523E-09	2.53E-07
SE	chr1	Cacna1e	-	154472060-154472713:154473857-154473913:154477766-154477863	205	149	0.7747	0.4503	0.324	0.324	4.13042E-07	1.38E-05
SE	chr9	Dcun1d5	+	7203451-7203555:7205298-7205400:7206720-7207031	251	149	0.9763	0.652	0.324	0.324	0	0
SE	chr4	Casp9	+	141793686-141793986:141796438-141796834:141803821-141803855	298	149	1	0.6763	0.324	0.324	3.1858E-07	1.1E-05
MXE	chr3	Lrrc7	-	158164275-158164386:158170556-158170756:158175103-158175351:15817	298	298	0.6183	0.2957	0.323	0.323	5.50733E-07	2.77E-05
SE	chr18	Myo5b	+	74713548-74713632:74714826-74714974:74715959-74716078	297	149	0.9743	0.6517	0.323	0.323	1.10989E-07	4.26E-06
SE	chr2	Rgs19	-	181691233-181691354:181691490-181691595:181693879-181693977	254	149	0.7143	0.391	0.323	0.323	3.30278E-11	2.58E-09
SE	chr3	Adgrl2	-	148815855-148817928:148818648-148818686:148820973-148821054	187	149	0.5543	0.2317	0.323	0.323	8.88599E-05	0.001509
SE	chr16	Trap1	-	4054754-4054909:4060757-4060917:4062330-4062401	298	149	0.9843	0.662	0.322	0.322	0	0
SE	chr9	Stac	-	111606821-111606921:111634806-111635085:111689998-111690348	298	149	0.4627	0.7843	-0.322	0.322	8.81189E-05	0.0015
A3SS	chr7	Pdcd5	-	35644351-35644412:35642615-35643715:35642615-35642686	298	149	0.9843	0.6633	0.321	0.321	0	0
A3SS	chr4	Eif4g3	+	138177917-138178010:138180490-138180607:138180547-138180607	205	149	0.409	0.73	-0.321	0.321	0	0
A5SS	chr5	Srpk2	-	23527924-23528062:23527897-23528062:23525496-23525745	175	149	0.9817	0.6607	0.321	0.321	2.87115E-12	4.37E-10
RI	chr5	Pigg	+	108336104-108336222:108336104-108336362:108336362-108336561	287	149	0.8193	0.498	0.321	0.321	1.22306E-06	1.86E-05
SE	chr7	Grm5	+	88074191-88075130:88116193-88116288:88129981-88130110	244	149	0.28	0.6013	-0.321	0.321	3.33067E-16	5.56E-14
MXE	chr1	Rgs7	-	175059076-175059794:175066617-175066694:175076017-175076097:17507	229	226	0.6697	0.35	0.32	0.32	2.39175E-12	4.59E-10
SE	chr9	Dync2h1	-	6993938-6994146:6996864-6996939:7001341-7001496	224	149	0.987	0.6673	0.32	0.32	9.77427E-10	5.85E-08
SE	chr15	Mtss1	-	58945846-58946019:58947326-58947520:58951470-58951680	298	149	0.8173	0.497	0.32	0.32	2.9097E-08	1.28E-06
MXE	chr1	Nfasc	-	132576286-132576417:132579378-132579508:132582995-132583147:13259	298	279	0.7947	0.4757	0.319	0.319	1.27396E-06	5.37E-05
SE	chr11	Sfi1	-	3133072-3133207:3134318-3134501:3135654-3135830	298	149	0.8917	0.5727	0.319	0.319	0.000598565	0.007743
SE	chr14	Slain1	+	103656933-103657072:103684234-103684383:103685669-103685818	298	149	0.8313	0.5127	0.319	0.319	0	0
SE	chr1	Nfasc	-	132576286-132576417:132579378-132579508:132582995-132583147	279	149	0.212	0.531	-0.319	0.319	1.76783E-07	6.47E-06
RI	chr9	Azi2	+	118059057-118059175:118059057-118059666:118059620-118059666	298	149	0.63	0.3123	0.318	0.318	0.001938223	0.010951
RI	chr3	Ampd2	-	108086218-108086352:108086218-108086666:108086439-108086666	234	149	0.7977	0.48	0.318	0.318	0.001531393	0.009083
SE	chr2	Dzank1	-	144490113-144490277:144491772-144491897:144501427-144501519	274	149	0.7897	0.4713	0.318	0.318	0.000609003	0.007869
SE	chr7	Mir9-3hg	+	79500026-79500250:79527763-79527869:79531186-79534403	255	149	0.371	0.6893	-0.318	0.318	6.53461E-09	3.35E-07
SE	chr1	Cspp1	+	10058720-10058823:10059824-10059847:10064333-10064413	172	149	0.533	0.2147	0.318	0.318	0.000440569	0.00595
A5SS	chr17	Wdr4	-	31509821-31509912:31509769-31509912:31503510-31503727	200	149	0.887	0.57	0.317	0.317	1.57025E-09	1.42E-07
SE	chr6	Vopp1	-	57789977-57790035:57808536-57808692:57824927-57825166	298	149	0.1253	0.4423	-0.317	0.317	0	0
SE	chr2	Nmt2	+	3305397-3305439:3306983-3307000:3309467-3309611	166	149	0.271	0.5877	-0.317	0.317	4.88298E-05	0.000905
SE	chr14	Erc2	+	28302876-28303010:28383906-28384043:28475603-28478537	286	149	0.1693	0.4867	-0.317	0.317	3.90705E-11	3.02E-09
SE	chr7	Ppp2r2d	+	138846612-138846704:138848459-138848665:138865021-138865117	298	149	0.4037	0.7203	-0.317	0.317	9.00448E-07	2.78E-05
SE	chr9	Rbm5	-	107756909-107756992:107759776-107759849:107760322-107760391	222	149	0.7397	0.424	0.316	0.316	3.75424E-08	1.6E-06
SE	chr1	Nfasc	-	132576286-132576417:132588074-132588310:132595484-132595657	298	149	0.7967	0.4803	0.316	0.316	0.001947156	0.020867
A5SS	chr1	Ankrd45	+	161163267-161163405:161163267-161164214:161167712-161170510	298	149	0.6537	0.3387	0.315	0.315	7.42336E-09	5.39E-07
SE	chr6	Ppfibp1	+	146996390-146996482:146996809-146996901:146998167-146998281	241	149	0.4913	0.1767	0.315	0.315	1.00816E-05	0.000231
SE	chr12	Susd6	+	80828026-80828243:80851214-80851276:80868427-80868624	211	149	0.0337	0.3483	-0.315	0.315	2.22045E-16	3.82E-14
SE	chr9	Arpp21	-	112149335-112149372:112155418-112155516:112176095-112176235	247	149	0.435	0.749	-0.314	0.314	0.000155537	0.002432
SE	chr11	Cacna1g	-	94438105-94438189:94439709-94439777:94442730-94442885	217	149	0.9553	0.6417	0.314	0.314	1.10978E-12	1.14E-10
A3SS	chrX	Enox2	-	49013497-49013582:49010107-49013180:49010107-49011634	298	149	0.526	0.2133	0.313	0.313	0.0001668	0.003625
SE	chr5	Vps37d	-	135072899-135073983:135076458-135076645:135078062-135078194	298	149	0.165	0.4783	-0.313	0.313	4.11327E-08	1.74E-06
SE	chr18	Rock1	-	10073115-10073185:10073657-10073700:10079115-10079274	192	149	0.4483	0.135	0.313	0.313	3.93912E-05	0.00075
RI	chr13	Srek1	-	103763882-103764006:103763882-103769304:103764296-103769304	298	149	0.4903	0.1787	0.312	0.312	3.55271E-15	2.22E-13
RI	chr7	Tial1	-	128443807-128443946:128443807-128444100:128444042-128444100	243	149	0.9	0.588	0.312	0.312	0	0
SE	chr3	Gm3764	+	88209635-88209716:88220079-88220325:88220956-88221284	298	149	0.729	0.4167	0.312	0.312	0.000105903	0.001753
SE	chr9	Dnm2	+	21489743-21489794:21490658-21490669:21494504-21494617	160	149	0.5977	0.2853	0.312	0.312	2.10754E-12	2.06E-10
SE	chr13	Erbb2ip	-	103824720-103824926:103827907-103828023:103833498-103835028	265	149	0.9193	0.6077	0.312	0.312	3.59452E-11	2.79E-09
SE	chr3	Odf2l	+	145126093-145126225:145127023-145127151:145127831-145127890	277	149	0.8587	0.547	0.312	0.312	1.06714E-06	3.23E-05
MXE	chr17	Nrxn1	-	90033952-90037384:90059485-90059563:90162114-90162433:90208323-902	298	227	0.8427	0.5313	0.311	0.311	0	0
RI	chr2	Oprl1	+	181715690-181715946:181715690-181716535:181716502-181716535	298	149	0.584	0.2733	0.311	0.311	1.1383E-08	2.64E-07
SE	chr3	Mynn	+	30611441-30611619:30612469-30612552:30613552-30613638	232	149	0.9437	0.633	0.311	0.311	1.26565E-13	1.51E-11
SE	chr12	Nrcam	+	44576640-44576789:44584835-44584987:44590108-44590239	298	149	0.5223	0.2117	0.311	0.311	0.000315718	0.004461
SE	chr11	Clk4	+	51267171-51267331:51268165-51268231:51270537-51270595	215	149	0.3483	0.6593	-0.311	0.311	0.000429029	0.005807
SE	chr17	Lmf1	+	25654420-25654606:25655088-25655241:25655385-25655568	298	149	0.9237	0.6123	0.311	0.311	1.13923E-06	3.42E-05
SE	chr6	Krba1	+	48407673-48407822:48408529-48408726:48409170-48409343	298	149	0.893	0.5817	0.311	0.311	2.56473E-12	2.46E-10
A3SS	chr9	Golga4	+	118576223-118577228:118579542-118582519:118581849-118582519	298	149	0.805	0.4953	0.31	0.31	1.39089E-12	3.04E-10
MXE	chr16	Golgb1	+	36875173-36875263:36885011-36885188:36886131-36886228:36887239-368	298	246	0.1573	0.467	-0.31	0.31	9.00484E-07	4.06E-05
RI	chr3	Lrrcc1	+	14562959-14563094:14562959-14568303:14565733-14568303	298	149	0.4123	0.102	0.31	0.31	1.96094E-09	5.28E-08
SE	chr2	Oprl1	+	181715690-181715946:181716502-181716535:181717955-181718093	182	149	0.1033	0.413	-0.31	0.31	6.79792E-11	4.98E-09
SE	chr5	Pkd2	+	104489233-104489414:104490644-104490764:104494893-104494991	269	149	0.8627	0.5537	0.309	0.309	0.000189295	0.002884
A3SS	chr9	Hmgn3	-	83110993-83111106:83109942-83110874:83109942-83110429	298	149	0.6203	0.312	0.308	0.308	0	0
RI	chr14	Rps24	+	24493383-24493493:24493383-24495449:24493617-24495449	271	149	0.52	0.212	0.308	0.308	0	0
SE	chr8	Zfp612	+	110083607-110083783:110084344-110084451:110088422-110092752	256	149	0.9543	0.6467	0.308	0.308	6.18335E-09	3.19E-07
SE	chr9	Zfp810	-	22276737-22279280:22280438-22280559:22283168-22283260	270	149	0.8513	0.543	0.308	0.308	1.99519E-06	5.6E-05
RI	chr11	Jmjd6	-	116838990-116839128:116838990-116839931:116839838-116839931	298	149	0.7273	0.4207	0.307	0.307	8.39772E-06	0.0001
RI	chr14	Rps24	+	24493617-24493619:24493617-24495449:24495430-24495449	298	149	0.5257	0.219	0.307	0.307	0	0
SE	chr10	Epb41l2	+	25488951-25489093:25490710-25493327:25501578-25501631	298	149	0.842	0.535	0.307	0.307	0.002159264	0.0228
SE	chr5	Iqce	-	140674337-140674360:140675078-140675248:140677416-140677506	298	149	0.9337	0.6263	0.307	0.307	3.453E-11	2.69E-09
SE	chr4	Kcnq4	-	120704355-120704575:120711223-120711384:120713012-120713100	298	149	0.9897	0.683	0.307	0.307	2.74936E-12	2.61E-10
SE	chr4	Dffb	-	153971581-153971660:153971886-153972060:153972780-153972864	298	149	0.5857	0.2783	0.307	0.307	0.001151552	0.013456
SE	chr12	Gm9222	+	18024048-18024174:18024375-18024435:18061968-18062438	209	149	0.069	0.3757	-0.307	0.307	2.21896E-08	9.97E-07

MXE	chr9	Gria4	-	4424207-4424454:4427030-4427144:4432773-4432887:4456005-4456252	263	263	0.3367	0.6427	-0.306	0.306	7.70437E-09	6.05E-07
SE	chr15	Hdac10	-	89126869-89126995:89127150-89127218:89127386-89127490	217	149	0.874	0.5677	0.306	0.306	2.56943E-07	9.02E-06
SE	chr14	Dcaf11	+	55559953-55560121:55560441-55560627:55561210-55561419	298	149	0.408	0.7137	-0.306	0.306	0.000454553	0.006111
SE	chr17	Aars2	+	45509396-45509540:45514492-45514637:45514923-45515283	294	149	0.8947	0.5887	0.306	0.306	4.05662E-05	0.00077
SE	chr17	Ermard	+	15059344-15059469:15059766-15059984:15061069-15061182	298	149	0.859	0.5533	0.306	0.306	5.94107E-09	3.07E-07
SE	chr9	Lrrfip2	+	111205755-111205847:111213813-111213914:111214141-111214273	250	149	0.612	0.3057	0.306	0.306	3.14839E-10	2.06E-08
A3SS	chr5	43719	+	93167500-93167687:93175404-93176462:93176040-93176462	298	149	0.4893	0.1847	0.305	0.305	2.02132E-07	1.31E-05
MXE	chr3	Lrrc7	-	158163892-158164072:158164275-158164386:158170556-158170756:158177	298	260	0.1673	0.4723	-0.305	0.305	2.74251E-07	1.51E-05
MXE	chr9	Zfp426	-	20473028-20473108:20474907-20475033:20476396-20476478:20486330-204	231	275	0.04	0.345	-0.305	0.305	1.38554E-08	1.06E-06
MXE	chrX	Pdzd4	-	73796376-73796477:73798194-73798292:73798758-73798848:73799834-738	239	247	0.8757	0.571	0.305	0.305	2.63189E-12	4.83E-10
SE	chr3	Sort1	+	108354133-108354241:108355472-108355570:108356231-108356337	247	149	0.4723	0.168	0.304	0.304	0	0
SE	chr8	Adgrl1	+	83923019-83923108:83924206-83924355:83926262-83926276	298	149	0.1597	0.4637	-0.304	0.304	0	0
SE	chr12	Mta1	+	113133185-113133337:113133496-113133576:113134801-113134812	229	149	0.9583	0.6543	0.304	0.304	4.6686E-11	3.57E-09
SE	chr4	Eif4g3	+	138082893-138082988:138095909-138095929:138096752-138096931	169	149	0.668	0.3637	0.304	0.304	3.89688E-14	5.01E-12
SE	chr12	Map3k9	-	81725722-81725834:81726202-81726330:81729683-81729836	277	149	0.5433	0.2397	0.304	0.304	0.000513974	0.006781
SE	chr8	Ssbp4	-	70599688-70599757:70599840-70599899:70600396-70600485	208	149	0.5743	0.8787	-0.304	0.304	1.73856E-08	7.95E-07
SE	chr5	Ccdc149	-	52385105-52385221:52391089-52391121:52392671-52392747	181	149	0.8467	0.544	0.303	0.303	3.77636E-05	0.000724
SE	chr17	Birc6	+	74593089-74593249:74594364-74594453:74594870-74595001	238	149	0.955	0.6523	0.303	0.303	1.35551E-08	6.43E-07
SE	chr1	Nfasc	-	132576286-132576417:132579378-132579508:132593351-132593365	279	149	0.121	0.424	-0.303	0.303	3.38565E-09	1.86E-07
SE	chr10	Dot1l	+	80790611-80791561:80792143-80792773:80793507-80795461	298	149	0.0843	0.387	-0.303	0.303	3.78508E-12	3.48E-10
SE	chr8	Cacna1a	+	84612285-84612412:84615574-84615670:84617792-84617897	245	149	0.9367	0.6337	0.303	0.303	4.0048E-08	1.7E-06
A3SS	chr19	Chka	+	3864822-3871062:3874769-3875788:3875735-3875788	298	149	0.9147	0.6127	0.302	0.302	1.0915E-06	5.43E-05
SE	chr1	Eif2d	+	131163520-131163565:131164620-131164769:131165120-131165209	298	149	0.95	0.648	0.302	0.302	9.57409E-10	5.74E-08
SE	chr8	Crlf1	+	70503305-70503492:70503592-70503639:70503821-70504078	196	149	0.814	0.512	0.302	0.302	5.11099E-07	1.68E-05
SE	chr9	Arpp21	-	112149335-112149372:112155418-112155519:112176095-112176235	250	149	0.456	0.758	-0.302	0.302	0.000170247	0.002632
SE	chr17	Mtcl1	-	66336982-66338154:66340484-66340540:66342826-66344365	205	149	0.8783	0.5763	0.302	0.302	5.49601E-09	2.86E-07
SE	chr3	Dclk2	-	86791995-86792111:86793219-86793299:86793817-86793913	229	149	0.8767	0.5753	0.301	0.301	0	0
SE	chr13	Erbb2ip	-	103823314-103823406:103827907-103828023:103833498-103835028	265	149	0.488	0.1873	0.301	0.301	5.60751E-07	1.83E-05
SE	chr9	Rbm7	-	48494019-48494181:48494283-48494387:48495155-48495330	253	149	0.3243	0.6257	-0.301	0.301	0	0
SE	chr3	Atp11b	+	35839049-35839214:35843571-35843696:35849434-35849564	274	149	0.8533	0.5523	0.301	0.301	2.22045E-16	3.82E-14
SE	chr15	Pacsin2	-	83378999-83379195:83379684-83379806:83381762-83381883	271	149	0.7133	0.4127	0.301	0.301	4.73013E-05	0.000879
SE	chr10	Ormdl2	-	128819954-128820105:128820261-128820435:128821542-128821632	298	149	0.9407	0.6393	0.301	0.301	1.67175E-07	6.16E-06
SE	chr5	Guf1	+	69561654-69561857:69563083-69563222:69564524-69564647	288	149	0.3113	0.6123	-0.301	0.301	2.10147E-11	1.7E-09
SE	chr16	Senp5	-	31964270-31964320:31965745-31965828:31968800-31968937	232	149	0.9943	0.6937	0.301	0.301	0	0
A3SS	chr8	Atp11a	+	12859314-12859479:12861912-12868728:12864920-12868728	298	149	0.9723	0.672	0.3	0.3	3.42926E-07	2.05E-05
SE	chr9	Atr	+	95921653-95921832:95922748-95922907:95927169-95927348	298	149	0.6847	0.385	0.3	0.3	0.000178313	0.00274
SE	chr14	Lmo7	+	101911999-101912160:101913240-101913346:101914277-101914337	255	149	1	0.7003	0.3	0.3	0	0
SE	chr10	Pip5k1c	+	81314992-81315118:81315879-81315956:81317326-81317403	226	149	0.3047	0.6043	-0.3	0.3	0	0
SE	chr15	BC024139	-	76119517-76119829:76120003-76120136:76120254-76120338	282	149	0.9577	0.658	0.3	0.3	2.63986E-09	1.47E-07
A3SS	chr7	Map6	+	99323692-99323787:99326009-99330469:99330364-99330469	298	149	0.6787	0.3793	0.299	0.299	0.00334408	0.037826
SE	chr2	Mllt10	+	18071125-18071204:18092168-18092834:18101458-18101512	298	149	0.3447	0.6437	-0.299	0.299	1.52519E-06	4.42E-05
SE	chr2	Gpsm1	+	26339636-26339706:26340405-26340584:26344602-26344758	298	149	0.8677	0.569	0.299	0.299	0	0
SE	chr5	Ccdc64	-	115670154-115670300:115672144-115672155:115673080-115673196	160	149	0.5983	0.2993	0.299	0.299	4.84666E-10	3.08E-08
SE	chr10	Ppfia2	+	106869879-106869972:106882736-106882744:106893432-106893513	157	149	0.4987	0.1993	0.299	0.299	1.5993E-10	1.09E-08
RI	chr5	Hnrnpdl	-	100034721-100034984:100034721-100035739:100035635-100035739	298	149	0.864	0.566	0.298	0.298	0	0
SE	chr17	Ehmt2	+	34905342-34905456:34905610-34905711:34905910-34906058	250	149	0.8653	0.5677	0.298	0.298	0	0
SE	chr2	Oprl1	+	181715690-181715946:181716502-181718093:181718377-181718732	298	149	0.2303	0.5287	-0.298	0.298	2.22045E-15	3.42E-13
SE	chr1	Zcchc2	+	106023664-106023758:106027403-106027531:106029752-106031233	277	149	0.8793	0.581	0.298	0.298	7.6951E-06	0.000182
SE	chr15	Trmu	+	85893954-85894020:85894178-85895026:85896360-85896504	298	149	0.9167	0.619	0.298	0.298	3.45618E-08	1.49E-06
SE	chr16	Yeats2	+	20206084-20206245:20207624-20207793:20208419-20208577	298	149	0.9137	0.6157	0.298	0.298	3.35636E-09	1.84E-07
MXE	chr2	Scn3a	-	65524604-65524876:65525695-65525786:65525877-65525968:65526497-655	240	240	0.4047	0.7017	-0.297	0.297	7.42917E-09	5.89E-07
SE	chr10	Epb41l2	+	25488951-25489093:25499776-25499943:25501578-25501631	298	149	0.8653	0.5687	0.297	0.297	0.00149105	0.016708
SE	chr13	Agtpbp1	-	59514087-59514295:59517653-59517690:59525146-59525239	186	149	0.912	0.6147	0.297	0.297	9.22595E-14	1.12E-11
SE	chr8	Crlf1	+	70503305-70503492:70503597-70503639:70503821-70504081	191	149	0.863	0.5657	0.297	0.297	3.30603E-07	1.13E-05
SE	chr5	Prkag2	-	24871494-24871659:24873884-24874010:24875161-24875215	275	149	0.9673	0.67	0.297	0.297	0	0
SE	chr13	Larp4b	+	9158123-9158229:9158551-9158802:9164675-9164720	298	149	0.8967	0.5997	0.297	0.297	0	0
SE	chr4	Lrp8	+	107843216-107843344:107846239-107846619:107847444-107847566	298	149	0.3057	0.6027	-0.297	0.297	0	0
SE	chr6	C2cd5	-	143017883-143018073:143019328-143019429:143020324-143020374	250	149	0.7027	0.4053	0.297	0.297	0.000563986	0.007362
SE	chr17	Zfp942	-	21930366-21930492:21932993-21933113:21941726-21941850	269	149	0.7293	0.4327	0.297	0.297	6.4595E-05	0.001145
A3SS	chr3	Gm3764	+	88227366-88227624:88228559-88229365:88229012-88229365	298	149	0.753	0.4573	0.296	0.296	0	0
MXE	chr5	Prkag2	-	24871494-24871659:24875161-24875215:24876989-24877034:24878705-248	194	203	0.9733	0.6777	0.296	0.296	0	0
MXE	chr1	Nfasc	-	132576286-132576417:132579378-132579508:132582995-132583147:13258	298	279	0.8013	0.5057	0.296	0.296	6.02995E-06	0.00021
RI	chr10	Pip5k1c	+	81315879-81315956:81315879-81317403:81317326-81317403	298	149	0.4503	0.154	0.296	0.296	0	0
SE	chr2	Fnbp4	+	90767408-90767541:90767853-90767955:90768516-90768700	251	149	0.4693	0.1737	0.296	0.296	8.60524E-11	6.17E-09
SE	chr9	Rbm5	-	107754423-107754488:107755853-107755913:107756909-107756992	209	149	0.984	0.6883	0.296	0.296	0	0
SE	chr1	Enah	-	181930678-181930762:181931174-181931185:181935660-181935716	160	149	0.9537	0.6577	0.296	0.296	1.68452E-07	6.19E-06
SE	chr1	Map2	+	66401444-66401521:66416735-66416866:66420135-66420196	280	149	0.2843	0.5803	-0.296	0.296	0	0
SE	chr19	Hectd2	+	36599596-36599719:36601429-36601528:36604250-36604357	248	149	0.9387	0.643	0.296	0.296	8.15579E-09	4.09E-07
A5SS	chr11	Ybx2	+	69939238-69939327:69939238-69939692:69939874-69940158	298	149	0.815	0.5203	0.295	0.295	0.000464065	0.006154
RI	chr11	Zfp207	+	80403365-80403662:80403365-80408557:80404741-80408557	298	149	0.805	0.51	0.295	0.295	0	0
SE	chr1	Map2	+	66401444-66401521:66416732-66416866:66420135-66420196	283	149	0.286	0.5807	-0.295	0.295	0	0
SE	chr1	Nfasc	-	132576286-132576417:132579378-132579508:132588074-132588310	279	149	0.1187	0.4133	-0.295	0.295	4.11157E-05	0.000779
SE	chr11	Bcas3	+	85814000-85814065:85822022-85822112:85825202-85826058	239	149	0.7937	0.499	0.295	0.295	0.000226147	0.003356
A5SS	chr12	Acp1	-	30904870-30904983:30904718-30904983:30897745-30897806	298	149	0.393	0.687	-0.294	0.294	0	0
SE	chr1	Pikfyve	+	65192145-65192294:65192531-65192566:65195656-65195774	184	149	0.5483	0.254	0.294	0.294	0.000146911	0.002318
SE	chr18	Lman1	-	65980739-65982710:65983825-65983887:65984153-65984274	211	149	0.9727	0.6787	0.294	0.294	1.59583E-12	1.61E-10
MXE	chr2	Myef2	-	125095912-125096120:125097977-125098075:125098450-125098518:12510	217	247	0.452	0.7447	-0.293	0.293	1.44329E-15	4.68E-13
SE	chr5	Mtif3	-	146958825-146959276:146963221-146963380:146963723-146963797	298	149	0.1337	0.4267	-0.293	0.293	0.000116408	0.001895
A3SS	chr14	Ap5m1	+	49086245-49086359:49088590-49090879:49090200-49090879	298	149	0.8033	0.5117	0.292	0.292	6.36317E-06	0.000251
A5SS	chr14	Mphosph8	+	56673891-56674739:56673891-56674775:56676522-56676618	184	149	0.2743	0.566	-0.292	0.292	3.6371E-08	2.22E-06
MXE	chr8	Ankrd10	-	11619062-11619297:11620262-11620373:11623697-11623858:11628439-116	298	260	0.4797	0.1873	0.292	0.292	1.45018E-06	5.99E-05
MXE	chr11	Bcas3	+	85801785-85801952:85814000-85814065:85822022-85822112:85825202-858	214	239	0.3013	0.5933	-0.292	0.292	0.000527561	0.009089
SE	chrX	A830080D01Rik	+	159574945-159575097:159591332-159591357:159592071-159593074	174	149	0.8253	0.5337	0.292	0.292	2.71618E-06	7.29E-05
SE	chr18	Tmx3	+	90534382-90534438:90537075-90537204:90538499-90538567	278	149	1	0.708	0.292	0.292	0	0
SE	chrX	Lrch2	-	147496767-147496832:147498288-147498365:147503384-147503491	226	149	0.8703	0.5787	0.292	0.292	6.69146E-11	4.92E-09
SE	chr7	Mir9-3hg	+	79500026-79500250:79523917-79523976:79531186-79534403	208	149	0.0783	0.3703	-0.292	0.292	3.3562E-12	3.13E-10
SE	chrX	Cask	-	13549165-13549361:13550947-13550982:13552373-13552441	184	149	0.8663	0.5747	0.292	0.292	1.11022E-16	1.97E-14
SE	chr12	Mok	-	110815131-110815209:110834126-110834240:110840746-110840793	263	149	0.5713	0.8637	-0.292	0.292	0.000117884	0.001915
SE	chr9	Armc8	-	99502512-99502598:99505265-99505346:99520487-99520582	230	149	0.9827	0.6917	0.291	0.291	1.63753E-09	9.38E-08
SE	chr11	Clk4	+	51271800-51272022:51273078-51273168:51273560-51273626	239	149	0.4573	0.1663	0.291	0.291	1.33227E-15	2.08E-13
SE	chr1	Stau2	-	16463035-16463194:16486020-16486150:16491241-16491394	279	149	0.8107	0.5193	0.291	0.291	1.12924E-06	3.4E-05
SE	chr12	Vrk1	+	106070525-106070615:106073619-106073678:106075007-106077410	208	149	0.6447	0.354	0.291	0.291	1.41423E-10	9.78E-09
SE	chr8	Hpf1	+	60890421-60890536:60896738-60896836:60904000-60904087	247	149	0.314	0.6037	-0.29	0.29	0.000758772	0.009473
SE	chr14	Gm3636	-	6741425-6741465:6752115-6752265:6756947-6757604	298	149	0.3243	0.6143	-0.29	0.29	0.001412808	0.015975
SE	chr5	Lyar	+	38220504-38220635:38222931-38223054:38223134-38223227	272	149	0.2213	0.511	-0.29	0.29	0.000605504	0.007829
SE	chr9	Msantd2	+	37487766-37489833:37513614-37513703:37517217-37517305	238	149	0.3147	0.6043	-0.29	0.29	7.00234E-06	0.000168
SE	chr1	Mff	+	82741818-82741976:82745524-82745556:82750520-82750604	181	149	0.5853	0.296	0.289	0.289	2.13076E-05	0.000443
SE	chr6	Vps50	+	3594743-3594901:3598472-3598593:3600128-3600317	270	149	0.9743	0.6853	0.289	0.289	0	0
SE	chr4	Zcchc7	+	44908451-44908486:44916180-44916312:44925975-44926062	281	149	0.5183	0.229	0.289	0.289	5.05335E-05	0.000932
MXE	chr3	Gria2	-	80689103-80689351:80690404-80690518:80691320-80691434:80691703-806	263	263	0.2183	0.5063	-0.288	0.288	0	0
MXE	chr1	Ppip5k2	-	97713004-97713144:97716542-97716604:97717442-97717561:97719834-977	268	211	0.7887	0.501	0.288	0.288	1.99656E-06	7.87E-05
MXE	chr1	Mdm4	-	133003791-133003890:133004654-133004721:133009137-133009192:133012	204	216	0.171	0.4587	-0.288	0.288	7.71218E-05	0.001811
MXE	chr1	Rgs7	-	175059076-175059794:175066617-175066694:175076017-175076097:17507	229	226	0.7107	0.4223	0.288	0.288	0.000493822	0.008624
RI	chr5	Hnrnpdl	-	100034721-100035739:100034721-100036195:100036104-100036195	298	149	0.7663	0.478	0.288	0.288	0	0
RI	chr16	Son	+	91671948-91674774:91671948-91675548:91675401-91675548	298	149	0.461	0.1727	0.288	0.288	0	0
SE	chr5	Drc1	+	30347056-30347193:30348663-30348749:30350401-30350523	235	149	0.9683	0.6807	0.288	0.288	5.42475E-11	4.07E-09
MXE	chr19	Naa40	-	7229722-7229805:7230188-7230283:7234104-7234156:7235063-7235158	201	244	0.905	0.618	0.287	0.287	4.01184E-09	3.4E-07
SE	chr6	Pot1a	-	25745891-25745996:25748291-25748382:25750050-25750138	240	149	0.9893	0.7023	0.287	0.287	4.56357E-12	4.16E-10
SE	chr2	Rgs19	-	181688419-181689295:181689377-181689611:181689702-181689776	298	149	0.8607	0.5737	0.287	0.287	1.28753E-12	1.31E-10
SE	chr14	Lmo7	+	101914277-101914337:101915688-101915744:101917631-101917656	205	149	0.5043	0.2173	0.287	0.287	7.69771E-07	2.43E-05
SE	chr7	Nap1l4	-	143513579-143514637:143518850-143518885:143520384-143520440	184	149	0.595	0.3087	0.286	0.286	0	0
SE	chr2	Myef2	-	125098450-125098518:125098797-125098847:125100319-125100420	199	149	0.552	0.2657	0.286	0.286	0	0
SE	chr15	Micall1	+	79115030-79115171:79119736-79119827:79120759-79120901	240	149	0.9457	0.6593	0.286	0.286	6.48148E-13	6.88E-11
MXE	chr9	Gria4	-	4424320-4424454:4427030-4427144:4432773-4432887:4440866-4440904	263	263	0.413	0.6983	-0.285	0.285	5.38542E-07	2.72E-05
RI	chr13	LOC108168101	-	74610200-74610326:74610200-74611321:74611264-74611321	298	149	0.5467	0.262	0.285	0.285	0.002693557	0.014375
SE	chr14	Camk2g	-	20745792-20745860:20747819-20747851:20755659-20755702	181	149	0.681	0.396	0.285	0.285	4.29289E-06	0.000109
SE	chr5	Gtf2i	-	134274539-134274595:134276372-134276431:134279437-134279514	208	149	0.321	0.6063	-0.285	0.285	0	0
SE	chr7	Prkcg	+	3313698-3313832:3314133-3314220:3314512-3314541	236	149	0.5717	0.2863	0.285	0.285	9.23431E-05	0.001559
SE	chr6	Atp2b2	-	113746873-113748848:113755217-113755388:113756832-113757014	298	149	0.0987	0.3833	-0.285	0.285	1.11022E-16	1.97E-14
SE	chr6	Casp2	+	42276695-42276785:42276870-42276930:42279777-42279926	209	149	0.316	0.601	-0.285	0.285	5.63463E-06	0.000138
A3SS	chr13	Brd9	+	73941944-73942054:73942631-73942806:73942691-73942806	208	149	0.6447	0.361	0.284	0.284	7.77156E-16	2.55E-13
SE	chr17	Wdr43	+	71640234-71640320:71641287-71641418:71642705-71642833	280	149	0.988	0.7043	0.284	0.284	0	0
SE	chr5	Atp8a1	-	67751013-67751085:67756650-67756694:67761114-67761202	193	149	0.9307	0.6467	0.284	0.284	2.39253E-10	1.6E-08
SE	chr14	Mycbp2	-	103155124-103156767:103169857-103170012:103172567-103172689	298	149	0.8453	0.5617	0.284	0.284	4.94826E-13	5.36E-11

SE	chr4	St3gal3	-	118031840-118031882:118054650-118054697:118107549-118107693	196	149	0.2197	0.5033	-0.284	0.284	1.19545E-07	4.55E-06
SE	chr6	Ift122	+	115925416-115925495:115925753-115925917:115926236-115926699	298	149	0.9763	0.6923	0.284	0.284	0	0
A5SS	chr6	Itpr1	+	108388185-108388338:108388185-108388365:108389367-108389554	175	149	0.885	0.6017	0.283	0.283	3.91607E-05	0.000811
MXE	chr16	Tvp23a	-	10427383-10427472:10428659-10428803:10446930-10447009:10447225-104	228	293	0.0713	0.3543	-0.283	0.283	1.10756E-12	2.31E-10
MXE	chr3	Gria2	-	80689103-80689351:80690404-80690518:80691320-80691434:80692285-806	263	263	0.2977	0.581	-0.283	0.283	0	0
SE	chr1	Ankrd45	+	161163267-161163405:161164172-161164214:161167712-161170505	191	149	0.7687	0.4857	0.283	0.283	7.93213E-09	4E-07
SE	chr16	Ttc3	+	94403314-94403368:94408554-94408716:94410833-94410956	298	149	0.6183	0.3357	0.283	0.283	0	0
SE	chr7	Emsy	-	98600672-98600827:98602583-98602747:98610667-98610865	298	149	0.8793	0.5963	0.283	0.283	1.23571E-08	5.92E-07
SE	chr12	Brf1	-	112979685-112979757:112983027-112983058:112986459-112986552	180	149	0.777	0.494	0.283	0.283	5.41784E-07	1.77E-05
SE	chr14	Erc2	+	28025033-28025173:28029434-28029469:28040343-28040536	184	149	0.466	0.183	0.283	0.283	4.32987E-14	5.53E-12
SE	chr19	Nrxn2	+	6509778-6509949:6513716-6513805:6516923-6517248	238	149	0.3347	0.6173	-0.283	0.283	0	0
SE	chr1	Nav1	-	135455134-135455330:135457861-135457869:135458656-135458777	157	149	0.601	0.318	0.283	0.283	1.73095E-12	1.73E-10
MXE	chr17	Nrxn1	-	90033952-90037384:90059476-90059563:90162114-90162433:90208323-902	298	236	0.6817	0.4	0.282	0.282	4.67404E-14	1.15E-11
MXE	chr16	Ttc3	+	94403314-94403368:94409684-94409729:94410833-94410956:94415949-944	194	272	0.679	0.3973	0.282	0.282	1.11925E-11	1.78E-09
MXE	chr1	Rps6kc1	-	190783564-190783646:190799013-190800581:190834006-190834098:19084	241	298	0.9327	0.651	0.282	0.282	1.35319E-06	5.63E-05
MXE	chr8	Nfix	-	84704712-84708677:84713766-84713857:84721643-84721818:84726469-847	298	240	0.093	0.3747	-0.282	0.282	7.61604E-08	4.9E-06
MXE	chr16	Senp5	-	31959670-31963537:31964270-31964320:31965745-31965828:31968800-319	232	199	0.5967	0.315	0.282	0.282	2.84273E-06	0.000109
SE	chr3	Tars2	-	95750701-95750765:95750838-95750984:95751506-95751630	295	149	0.896	0.614	0.282	0.282	1.07413E-05	0.000244
SE	chr3	Rrnad1	-	87927021-87927117:87927563-87927660:87927924-87928016	246	149	0.949	0.6673	0.282	0.282	8.3923E-09	4.19E-07
SE	chr4	Pum1	+	130701042-130701110:130718157-130718262:130741463-130741556	254	149	0.182	0.464	-0.282	0.282	0.000568892	0.007419
SE	chr15	Ptk2	-	73330849-73331015:73365041-73365195:73394648-73394744	298	149	0.5487	0.8303	-0.282	0.282	0.000586176	0.007609
SE	chr1	Cspp1	+	10083501-10083558:10084013-10085952:10088051-10088184	298	149	0.1913	0.4737	-0.282	0.282	1.10934E-06	3.34E-05
A3SS	chrX	Armcx2	-	134807621-134807725:134807262-134807396:134807262-134807327	217	149	0.5137	0.2327	0.281	0.281	1.34944E-07	9.45E-06
SE	chr2	Madd	-	91138709-91138755:91140557-91140626:91142756-91142862	218	149	0.206	0.4873	-0.281	0.281	1.3831E-10	9.6E-09
SE	chr2	Cep250	+	155975671-155975864:155976084-155976211:155976389-155976558	276	149	0.967	0.6863	0.281	0.281	5.81535E-13	6.23E-11
SE	chr5	Pisd	-	32739137-32739373:32740624-32740789:32742210-32742475	298	149	0.93	0.6493	0.281	0.281	2.83721E-06	7.56E-05
A5SS	chr4	Rmdn1	+	19601356-19601443:19601356-19601606:19602643-19602673	298	149	0.4687	0.189	0.28	0.28	1.16393E-08	8.09E-07
SE	chr1	Cnnm3	+	36521701-36521839:36524034-36524132:36525166-36528237	247	149	0.3817	0.1017	0.28	0.28	3.04984E-11	2.4E-09
MXE	chr1	Clasp1	+	118525297-118525455:118531978-118532085:118536135-118536197:11853	256	211	0.1173	0.3967	-0.279	0.279	1.16362E-12	2.4E-10
SE	chr1	Rcor3	-	192098769-192101155:192101626-192101867:192108644-192108701	298	149	0.8477	0.5687	0.279	0.279	0.000920232	0.011141
SE	chr2	Scn3a	-	65525695-65525786:65525877-65525968:65526497-65526625	240	149	0.215	0.4943	-0.279	0.279	4.99833E-05	0.000923
SE	chr16	Golgb1	+	36885011-36885188:36886134-36886228:36887239-36887403	243	149	0.9413	0.6623	0.279	0.279	3.74885E-06	9.65E-05
SE	chr1	Tceb1	-	16647955-16648007:16656296-16656459:16656992-16657119	298	149	0.5263	0.2477	0.279	0.279	5.30467E-11	4E-09
SE	chr7	Igflr1	+	30566164-30566381:30566749-30566936:30567143-30567500	298	149	0.8873	0.6087	0.279	0.279	2.39517E-05	0.00049
MXE	chr16	Ttc3	+	94403314-94403368:94408554-94408716:94410833-94410956:94415949-944	298	272	0.7713	0.4933	0.278	0.278	0	0
MXE	chr15	Ptk2	-	73325749-73325836:73343133-73343359:73365041-73365195:73394648-733	298	298	0.1943	0.4723	-0.278	0.278	7.39293E-10	7.62E-08
RI	chr10	Myl6b	-	128497201-128497417:128497201-128497714:128497646-128497714	298	149	0.7637	0.486	0.278	0.278	2.55723E-05	0.000266
RI	chr10	Pip4k2c	-	127197067-127198748:127197067-127199144:127199045-127199144	298	149	0.9667	0.6887	0.278	0.278	0	0
SE	chr8	Adgrl1	+	83923019-83923108:83924206-83924355:83926259-83926276	298	149	0.5667	0.8443	-0.278	0.278	2.3415E-06	6.42E-05
SE	chr10	Ank3	+	69980272-69980394:69980824-69980850:69982116-69982161	175	149	0.5177	0.24	0.278	0.278	3.78031E-13	4.19E-11
SE	chr9	Golga4	+	118577142-118577228:118579542-118579606:118581849-118582408	213	149	0.8497	0.5717	0.278	0.278	1.91114E-12	1.89E-10
SE	chr10	Ptprk	+	28560005-28560165:28562107-28562142:28566495-28566679	184	149	0.814	0.5357	0.278	0.278	6.16099E-07	1.99E-05
SE	chr9	Msantd2	+	37487766-37489833:37513614-37513703:37517373-37517459	238	149	0.3363	0.614	-0.278	0.278	0.000105553	0.001748
SE	chr3	Mfn1	+	32563012-32563138:32563375-32563493:32563793-32563895	267	149	0.965	0.687	0.278	0.278	2.08944E-13	2.42E-11
SE	chr2	Fam188a	-	12386609-12386679:12410001-12410080:12419159-12419460	228	149	0.581	0.8577	-0.277	0.277	1.58183E-07	5.84E-06
A5SS	chr2	Gfra4	-	131041016-131041150:131040951-131041150:131039632-131040325	213	149	0.7963	0.52	0.276	0.276	0.000231553	0.003478
SE	chr16	Itsn1	+	91841489-91841610:91842628-91842642:91848722-91848969	163	149	0.3503	0.074	0.276	0.276	0	0
A5SS	chr1	Taf1a	+	183408610-183408724:183408610-183408763:183409235-183410201	187	149	0.6813	0.4067	0.275	0.275	0.000178627	0.002804
RI	chr8	C230057M02Rik	-	122729771-122735056:122729771-122738893:122735738-122738893	298	149	0.8307	0.556	0.275	0.275	0.000730828	0.004827
SE	chr7	Slc8a2	+	16150341-16150444:16150574-16150591:16152819-16152943	166	149	0.862	0.5873	0.275	0.275	2.49578E-13	2.86E-11
SE	chr2	Dusp15	-	152949038-152949120:152950621-152950654:152951335-152951405	182	149	0.873	0.598	0.275	0.275	6.55195E-11	4.83E-09
SE	chr3	Arnt	+	95490255-95490378:95490553-95490655:95491023-95491170	251	149	0.7863	0.511	0.275	0.275	6.35639E-07	2.05E-05
SE	chr15	Mapk15	+	75994845-75994943:75995072-75995101:75995234-75995324	178	149	0.6803	0.4053	0.275	0.275	0.001793373	0.019491
SE	chr4	Casp9	+	141793612-141793986:141796435-141796834:141803821-141803855	298	149	1	0.7253	0.275	0.275	9.82135E-07	3.01E-05
SE	chr5	Fryl	-	73050302-73050505:73050867-73050875:73054053-73054229	157	149	0.3937	0.1187	0.275	0.275	8.47917E-07	2.64E-05
SE	chr7	Pnkp	+	44859888-44859945:44860077-44860184:44860360-44860431	256	149	0.9037	0.6287	0.275	0.275	1.37174E-09	7.99E-08
MXE	chr3	Cp	+	19987933-19988072:19989053-19989218:20005467-20005571:20008540-200	298	253	0.5897	0.8633	-0.274	0.274	0.000168057	0.003496
RI	chr13	Slc38a9	+	112731499-112735864:112731499-112738751:112737988-112738751	298	149	0.9167	0.6427	0.274	0.274	0.000128497	0.001067
SE	chr19	Syt7	+	10417963-10418042:10421757-10421888:10435550-10435685	280	149	0.4887	0.7623	-0.274	0.274	2.10814E-09	1.2E-07
MXE	chr2	Dph7	+	24962174-24962810:24963023-24963156:24963456-24963543:24965557-249	282	236	0.5643	0.291	0.273	0.273	0.000100452	0.002266
MXE	chr16	Robo1	+	72145614-72145748:72563700-72563783:72742126-72742452:72904630-729	232	298	0.3923	0.1197	0.273	0.273	9.20196E-11	1.2E-08
RI	chr8	C230057M02Rik	-	122729771-122734273:122729771-122735056:122735009-122735056	298	149	0.789	0.5157	0.273	0.273	0.008618219	0.037978
SE	chr11	Kif1c	+	70700323-70700709:70702446-70702551:70702772-70702905	254	149	0.6587	0.386	0.273	0.273	0.000362648	0.005018
SE	chr2	Metap1d	+	71512126-71512168:71522533-71522630:71523802-71523849	246	149	0.989	0.716	0.273	0.273	1.3441E-11	1.13E-09
SE	chr9	Arpp21	-	112143309-112143538:112147423-112147482:112149335-112149372	208	149	0.4387	0.7113	-0.273	0.273	6.61851E-09	3.39E-07
SE	chr6	Vopp1	-	57774711-57774788:57808536-57808692:57824927-57825166	298	149	0.245	0.518	-0.273	0.273	1.24678E-13	1.49E-11
SE	chr19	Tmem180	+	46369237-46369333:46371936-46372107:46372182-46372375	298	149	0.9637	0.6903	0.273	0.273	3.61659E-07	1.22E-05
SE	chr11	Epn2	-	61533571-61533681:61534578-61534613:61535271-61535383	184	149	0.4213	0.1487	0.273	0.273	1.11022E-16	1.97E-14
SE	chr15	Rbfox2	-	77086067-77086155:77089862-77089893:77097940-77098072	180	149	0.323	0.0497	0.273	0.273	1.90958E-14	2.58E-12
SE	chr9	Pkm	+	59671921-59672073:59675029-59675195:59675568-59675734	298	149	0.6287	0.3557	0.273	0.273	0	0
SE	chr5	Camkk2	-	122737433-122737533:122742100-122742228:122743870-122743957	277	149	0.975	0.702	0.273	0.273	0	0
MXE	chr1	Enah	-	181930678-181930762:181931174-181931185:181935660-181935716:18195	205	160	0.1357	0.4073	-0.272	0.272	9.84074E-05	0.002229
SE	chr14	Mettl3	-	52297869-52298085:52298640-52298815:52299701-52300101	298	149	0.5697	0.2973	0.272	0.272	2.55765E-06	6.93E-05
SE	chr1	Pikfyve	+	65192145-65192294:65192534-65192566:65195656-65195774	181	149	0.4313	0.1597	0.272	0.272	3.77038E-05	0.000723
SE	chr17	Slc25a27	-	43661614-43661698:43664074-43665125:43666900-43666986	298	149	0.971	0.6987	0.272	0.272	2.04281E-14	2.74E-12
SE	chr15	Chkb	-	89428134-89428229:89428411-89428578:89428714-89428827	298	149	0.972	0.6997	0.272	0.272	0	0
SE	chr1	Ppip5k2	-	97716542-97716604:97717442-97717561:97719834-97719906	268	149	0.9877	0.7153	0.272	0.272	3.67595E-13	4.09E-11
SE	chr5	Afap1	+	35984189-35984306:35987055-35987321:35989554-35989668	298	149	0.8583	0.5867	0.272	0.272	4.93333E-10	3.13E-08
SE	chr14	Slmap	-	26459410-26459460:26459904-26459954:26460042-26460210	199	149	0.8727	0.6003	0.272	0.272	7.87465E-06	0.000186
SE	chr4	Sfpq	+	127027397-127027518:127029827-127032805:127035808-127035854	298	149	0.8177	0.546	0.272	0.272	0	0
SE	chr2	H13	+	152700183-152700268:152703995-152704194:152704622-152705052	298	149	0.7033	0.4317	0.272	0.272	0	0
SE	chr8	Dcun1d2	-	13271590-13271720:13278517-13278685:13287804-13287879	298	149	0.8773	0.6057	0.272	0.272	3.4777E-07	1.18E-05
SE	chr2	Fam73b	+	30382966-30383019:30383590-30383706:30383929-30385519	265	149	0.8353	0.5637	0.272	0.272	2.54702E-11	2.03E-09
SE	chr14	Erc2	+	28317249-28317314:28383824-28384043:28475603-28478537	298	149	0.21	0.482	-0.272	0.272	2.49123E-12	2.4E-10
SE	chr18	AW554918	+	25339714-25340384:25344864-25344962:25399926-25400107	247	149	0.4243	0.6963	-0.272	0.272	0.001320071	0.015118
SE	chrX	Cnksr2	-	157910117-157910250:157919746-157919892:157934640-157934708	295	149	0.3767	0.649	-0.272	0.272	1.17315E-07	4.48E-06
SE	chr7	Usp29	+	6738807-6738945:6908286-6908389:6927452-6927516	252	149	0.1107	0.383	-0.272	0.272	1.20848E-07	4.59E-06
MXE	chr17	St6gal2	+	55444368-55445809:55463019-55463171:55463886-55463958:55481905-554	298	221	0.2463	0.5177	-0.271	0.271	0.000166726	0.003477
SE	chr4	Nfib	-	82310303-82310391:82320474-82320556:82327729-82327913	231	149	0.1447	0.4157	-0.271	0.271	3.36003E-06	8.76E-05
SE	chr14	Dzip1	-	118922911-118923167:118923897-118924017:118924425-118924518	269	149	0.7473	0.4763	0.271	0.271	9.55375E-09	4.7E-07
SE	chr14	Pcdh9	-	93326679-93326880:93560476-93560577:93885697-93888888	250	149	0.3817	0.6523	-0.271	0.271	0.000507816	0.006714
SE	chr1	Clasp1	+	118531978-118532085:118536135-118536197:118539266-118539369	211	149	0.932	0.661	0.271	0.271	7.03433E-07	2.24E-05
SE	chr13	Tppp	+	74021139-74021450:74030750-74030903:74031221-74035753	298	149	0.9257	0.6543	0.271	0.271	3.26585E-08	1.41E-06
A3SS	chr1	Usp40	-	87954151-87954245:87952340-87954051:87952340-87952458	298	149	0.391	0.1213	0.27	0.27	1.63055E-06	7.49E-05
MXE	chr17	Traf7	-	24513193-24513376:24513899-24513932:24514125-24514217:24516505-245	241	182	0.0523	0.322	-0.27	0.27	1.04203E-11	1.69E-09
MXE	chr14	Uggt2	-	119002826-119002986:119007145-119007327:119008063-119008147:11900	233	298	0.244	0.5143	-0.27	0.27	4.33374E-06	0.000157
SE	chr2	Metap1d	+	71515637-71515800:71522511-71522630:71523802-71523849	268	149	0.9247	0.655	0.27	0.27	3.31203E-07	1.13E-05
SE	chr3	Dram2	+	106566045-106566184:106571611-106571693:106572973-106573065	231	149	0.8393	0.569	0.27	0.27	2.11267E-06	5.88E-05
SE	chr7	Fam168a	+	100812920-100813000:100818736-100818762:100824132-100824257	175	149	0.6153	0.3457	0.27	0.27	3.9968E-15	6E-13
SE	chr14	Mycbp2	-	103155124-103156767:103160841-103160942:103172567-103172689	250	149	0.4567	0.187	0.27	0.27	2.17909E-07	7.8E-06
SE	chr4	Icmt	+	152297214-152297568:152298685-152298773:152299671-152299840	237	149	0.9553	0.6853	0.27	0.27	4.74063E-09	2.53E-07
SE	chr4	Inpp5b	+	124742933-124743030:124743636-124743733:124743874-124743903	246	149	0.869	0.5987	0.27	0.27	1.96391E-06	5.53E-05
SE	chrX	Zfp280c	-	48543405-48543441:48543585-48543703:48547265-48547327	267	149	0.8323	0.562	0.27	0.27	1.27549E-08	6.08E-07
MXE	chr1	Stau2	-	16463035-16463194:16486020-16486165:16491241-16491394:16509342-165	298	294	0.263	0.532	-0.269	0.269	1.35489E-06	5.63E-05
SE	chr18	Snx2	+	53216381-53216524:53216970-53216981:53218102-53218182	160	149	0.6367	0.368	0.269	0.269	1.22125E-15	1.92E-13
SE	chr4	Usp48	+	137616358-137616507:137619923-137619974:137621062-137621259	200	149	0.0443	0.3133	-0.269	0.269	8.61011E-12	7.48E-10
SE	chr8	2610044O15Rik8	-	129221544-129221604:129221824-129221950:129233905-129234046	275	149	0.944	0.6747	0.269	0.269	3.0112E-07	1.04E-05
SE	chr5	Fam109a	+	121849028-121849124:121850470-121850546:121851168-121851279	225	149	0.8773	0.608	0.269	0.269	3.46525E-11	2.69E-09
SE	chr3	Sh3d19	+	86107212-86107352:86113106-86113178:86114787-86114917	221	149	0.9877	0.7187	0.269	0.269	4.68758E-11	3.57E-09
SE	chr11	Fam134c	-	101102006-101102090:101102883-101103009:101103890-101103920	275	149	0.9133	0.6443	0.269	0.269	3.88578E-15	5.85E-13
A3SS	chr4	Snapc3	+	83460163-83460359:83461031-83466137:83465965-83466137	298	149	0.6863	0.4187	0.268	0.268	0.000253531	0.005125
MXE	chr9	Zfp426	-	20473028-20473108:20476396-20476478:20478133-20478254:20486330-204	270	231	0.9387	0.6703	0.268	0.268	1.07959E-06	4.66E-05
MXE	chr1	Nfasc	-	132576286-132576417:132579378-132579508:132584364-132584480:13258	265	279	0.7587	0.4907	0.268	0.268	0.000667668	0.010864
MXE	chr1	Cspp1	+	10058720-10058823:10064333-10064413:10065323-10065421:10066426-100	229	247	0.1307	0.3987	-0.268	0.268	5.0034E-06	0.000178
SE	chr9	Cep295	-	15324149-15324291:15325507-15325746:15326075-15326177	298	149	0.914	0.6463	0.268	0.268	5.57712E-11	4.16E-09
SE	chr2	Cables2	-	180264214-180264306:180264403-180264474:180273100-180273465	220	149	0.6137	0.8817	-0.268	0.268	5.8211E-09	3.02E-07
SE	chr1	Mfsd6	-	52656299-52658545:52658699-52658723:52660807-52661087	173	149	0.9497	0.682	0.268	0.268	1.05449E-12	1.09E-10
SE	chr5	Mphosph9	-	124315373-124315896:124316082-124316171:124320845-124320954	238	149	0.629	0.3607	0.268	0.268	0.000401831	0.005483
MXE	chr18	Nme5	-	34562636-34562803:34567036-34567154:34571456-34571661:34578558-345	298	267	0.4503	0.1833	0.267	0.267	1.33166E-05	0.000409
SE	chr13	Ercc6l2	+	63869026-63869178:63870689-63870769:63871796-63872927	229	149	0.9363	0.669	0.267	0.267	6.38897E-05	0.001135
SE	chr5	Limk1	-	134661389-134661858:134662103-134662157:134664185-134664316	203	149	0.9577	0.6907	0.267	0.267	4.98402E-06	0.000124
A3SS	chr17	3110052M02Rik	+	21652267-21652345:21656096-21657611:21657519-21657611	298	149	0.8353	0.569	0.266	0.266	8.28609E-08	6.5E-06
SE	chr10	Epb41l2	+	25488951-25489093:25490710-25493327:25499776-25499943	298	149	0.971	0.7053	0.266	0.266	3.5913E-06	9.29E-05
SE	chr17	Adamts10	+	33524121-33524258:33528284-33528470:33528680-33528828	298	149	0.8997	0.6337	0.266	0.266	4.62367E-09	2.47E-07

SE	chr19	Carnmt1	+	18693649-18693762:18703361-18703464:18704020-18704792	252	149	0.8853	0.619	0.266	0.266	3.59642E-05	0.000695
A5SS	chr6	Ptprz1	+	22999200-23000199:22999200-23002746:23007274-23007418	298	149	0.893	0.6283	0.265	0.265	0	0
A5SS	chr6	Dctn1	+	83179599-83179811:83179599-83179905:83182524-83182769	242	149	0.9027	0.6373	0.265	0.265	1.62421E-06	5.77E-05
SE	chr2	Itch	+	155138196-155138272:155157774-155157975:155163393-155163483	298	149	0.366	0.1007	0.265	0.265	4.05231E-13	4.45E-11
SE	chr1	Clasp1	+	118525297-118525455:118531978-118532085:118536135-118536197	256	149	0.1853	0.45	-0.265	0.265	3.9666E-11	3.06E-09
SE	chr4	Ptprd	-	76133063-76133332:76135545-76135556:76136811-76136921	160	149	0.945	0.68	0.265	0.265	0	0
SE	chr5	Nup54	-	92424274-92424470:92425623-92425810:92428100-92428326	298	149	0.9223	0.6573	0.265	0.265	0	0
MXE	chr1	Slco5a1	-	12881293-12881452:12894427-12894625:12938989-12939127:12943980-129	287	298	0.0383	0.3023	-0.264	0.264	4.12465E-07	2.12E-05
RI	chr2	Snhg11	+	158380939-158381040:158380939-158386145:158382504-158386145	298	149	0.9803	0.7167	0.264	0.264	0	0
SE	chr17	Nrxn1	-	90560741-90560860:90561802-90561828:90565305-90565478	175	149	0.4127	0.149	0.264	0.264	1.662E-13	1.96E-11
SE	chr5	Adgrl3	+	81771584-81771680:81792361-81792412:81794264-81802140	200	149	0.724	0.4603	0.264	0.264	1.42422E-06	4.16E-05
SE	chr4	Usp1	+	98923810-98924243:98926455-98926674:98928234-98928354	298	149	0.6807	0.4163	0.264	0.264	2.7078E-07	9.45E-06
SE	chr7	Tial1	-	128446195-128446270:128446731-128446798:128448121-128448208	216	149	0.417	0.6807	-0.264	0.264	4.84595E-08	2.01E-06
SE	chr15	Tfcp2	-	100520563-100520715:100522389-100522495:100525567-100525672	255	149	0.77	0.5063	0.264	0.264	4.09108E-05	0.000775
A3SS	chr13	Grk6	+	55459013-55459147:55459782-55460927:55459784-55460927	150	149	0.6413	0.3787	0.263	0.263	1.4969E-08	1.4E-06
RI	chr11	Cdkl3	+	52084371-52084490:52084371-52089778:52089589-52089778	298	149	0.5157	0.253	0.263	0.263	1.84671E-06	2.69E-05
SE	chr2	Nsun6	-	15038074-15038227:15039733-15039842:15042227-15042306	258	149	0.9373	0.6743	0.263	0.263	8.88202E-05	0.001509
SE	chr18	Fhod3	+	25001791-25002032:25022622-25022978:25028065-25028169	298	149	0.0473	0.3103	-0.263	0.263	6.66134E-16	1.08E-13
SE	chr2	Rbck1	-	152322160-152322271:152323172-152323332:152324267-152324440	298	149	0.8113	0.5483	0.263	0.263	3.35887E-12	3.13E-10
SE	chr2	Phf21a	+	92320229-92320459:92326913-92327164:92328315-92328401	298	149	0.8373	0.5747	0.263	0.263	6.87325E-05	0.001209
SE	chr9	Arpp21	-	112143309-112143538:112147423-112147479:112149335-112149372	205	149	0.2863	0.5497	-0.263	0.263	2.12851E-06	5.91E-05
SE	chr11	Cbx1	+	96802728-96802822:96806616-96806794:96807803-96808149	298	149	0.925	0.662	0.263	0.263	0	0
SE	chr11	Tvp23b	+	62885076-62885165:62886189-62886320:62891942-62892070	280	149	0.907	0.6437	0.263	0.263	3.35731E-13	3.75E-11
SE	chr2	H13	+	152700183-152700268:152703995-152704141:152704622-152705052	295	149	0.5657	0.3023	0.263	0.263	0	0
SE	chr10	Agap2	+	127089230-127089358:127090142-127090201:127090427-127090575	208	149	0.9167	0.6537	0.263	0.263	0	0
A5SS	chr2	Gfra4	-	131041016-131041150:131040889-131041150:131039632-131040325	275	149	0.8463	0.584	0.262	0.262	3.50321E-05	0.000735
SE	chr1	Map4k4	+	40007433-40007530:40008331-40008339:40009688-40009863	157	149	0.9307	0.6687	0.262	0.262	0	0
SE	chr9	Neil1	-	57143451-57143613:57143758-57143819:57143979-57144006	210	149	0.51	0.2477	0.262	0.262	0.000394365	0.00539
SE	chr3	Gpsm2	-	108702865-108703086:108711277-108711580:108722041-108722299	298	149	0.8697	0.608	0.262	0.262	1.55613E-05	0.000335
SE	chr2	Mllt10	+	18071125-18071204:18092784-18092834:18101458-18101512	199	149	0.1613	0.4233	-0.262	0.262	0.000164594	0.002556
A5SS	chr19	Fam178a	+	44941459-44942426:44941459-44942939:44943449-44943519	298	149	0.6887	0.4277	0.261	0.261	4.63251E-09	3.58E-07
SE	chr12	Ralgapa1	-	55602890-55604268:55612073-55612103:55612634-55612758	179	149	0.976	0.715	0.261	0.261	0	0
SE	chr6	Hnrnpa2b1	-	51461121-51461433:51462339-51463254:51463411-51463493	298	149	0.9783	0.7177	0.261	0.261	0	0
SE	chr6	Ing3	+	21949615-21949793:21950038-21950320:21952129-21952229	298	149	0.9083	0.647	0.261	0.261	3.37245E-05	0.000659
SE	chr9	Lrrc49	-	60610258-60610421:60614999-60615177:60621569-60621716	298	149	0.9833	0.7227	0.261	0.261	0	0
SE	chr6	Tac1	+	7556684-7556780:7557216-7557260:7557720-7557743	193	149	0.372	0.6327	-0.261	0.261	5.16877E-10	3.27E-08
SE	chr15	Rbfox2	-	77086067-77086155:77094451-77094490:77097940-77098072	188	149	0.904	0.643	0.261	0.261	0	0
MXE	chr9	Map2k5	-	63194202-63194247:63197311-63197372:63216993-63217025:63235289-632	181	210	0	0.2597	-0.26	0.26	7.14598E-11	9.64E-09
MXE	chr13	Atxn1	-	45735115-45735178:45795509-45795637:45956455-45956575:45963574-459	269	277	0.0633	0.323	-0.26	0.26	2.37921E-05	0.000675
RI	chr6	Magi1	-	93675453-93679002:93675453-93683090:93682952-93683090	298	149	0.787	0.527	0.26	0.26	2.09288E-12	9.49E-11
SE	chr11	Lrrc45	+	120717480-120717538:120717641-120717902:120718037-120718172	298	149	0.8127	0.5527	0.26	0.26	0.000286741	0.004114
SE	chr9	Lrrfip2	+	111205755-111205847:111207797-111207889:111213813-111213914	241	149	0.1457	0.4057	-0.26	0.26	0.000732385	0.009201
SE	chr2	Chchd5	+	129129700-129129857:129130401-129130566:129133275-129134112	298	149	0.488	0.7483	-0.26	0.26	2.60251E-05	0.000527
SE	chr15	Ptk2	-	73343133-73343359:73365041-73365195:73394648-73394744	298	149	0.2273	0.487	-0.26	0.26	2.04976E-08	9.25E-07
SE	chr14	Erc2	+	28317249-28317314:28383906-28384043:28475603-28478537	286	149	0.147	0.407	-0.26	0.26	6.31939E-13	6.72E-11
A5SS	chr18	Poli	-	70530188-70530321:70529751-70530321:70528702-70528827	298	149	0.4777	0.737	-0.259	0.259	0.001511688	0.015755
MXE	chr5	Prkag2	-	24871494-24871659:24875161-24875215:24878705-24878763:24880684-248	207	203	0.9803	0.721	0.259	0.259	0	0
MXE	chr10	Fam229b	-	39118804-39119034:39120258-39120396:39122177-39122335:39132247-391	298	287	0.5627	0.8213	-0.259	0.259	5.33639E-10	5.81E-08
MXE	chr16	Senp5	-	31964270-31964320:31968800-31968937:31977680-31977757:31983264-319	226	286	0.938	0.6793	0.259	0.259	7.23472E-07	3.43E-05
SE	chr1	Pogk	-	166408399-166408532:166408998-166409331:166409707-166409828	298	149	0.8223	0.5637	0.259	0.259	8.73937E-09	4.35E-07
SE	chr2	Osbpl6	+	76406601-76406689:76407175-76407605:76499257-76499422	298	149	0.1063	0.3653	-0.259	0.259	1.16724E-05	0.000262
SE	chr5	Smurf1	-	144897983-144898058:144899147-144899212:144899365-144899498	214	149	0.9717	0.713	0.259	0.259	5.55112E-16	9.03E-14
SE	chrX	BC065397	+	136759856-136759918:136760418-136760575:136761991-136762088	298	149	0.9853	0.726	0.259	0.259	3.88674E-07	1.31E-05
SE	chr8	Rfx1	+	84094727-84094876:84094950-84095158:84095396-84095549	298	149	0.9853	0.727	0.258	0.258	1.79823E-12	1.79E-10
SE	chr14	Ccser2	-	36885751-36885790:36890342-36890426:36896255-36896344	233	149	0.9927	0.735	0.258	0.258	0	0
SE	chr13	Spock1	-	57426091-57429485:57430289-57430426:57436110-57436172	286	149	0.9573	0.6997	0.258	0.258	0	0
SE	chr6	Hnrnpa2b1	-	51461121-51461433:51461983-51463254:51463411-51463493	298	149	0.9793	0.7217	0.258	0.258	0	0
SE	chr6	Erc1	-	119694571-119694731:119713680-119713811:119722173-119722308	280	149	0.1687	0.4267	-0.258	0.258	3.10862E-14	4.06E-12
SE	chr12	Entpd5	-	84396679-84396958:84399340-84399412:84405406-84405475	221	149	0.436	0.1783	0.258	0.258	0.00073458	0.009216
SE	chr4	Nol9	+	152045695-152045830:152045945-152046035:152046547-152046644	239	149	0.7707	0.5127	0.258	0.258	0.004916424	0.045512
MXE	chr7	Ptpn5	-	47089777-47090018:47090421-47090504:47090727-47090834:47091377-470	256	232	0.4887	0.232	0.257	0.257	0	0
MXE	chr9	Map2k5	-	63216993-63217025:63217339-63217365:63235289-63235318:63256977-632	178	175	0.2203	0.4777	-0.257	0.257	0.000991597	0.014924
MXE	chrX	Cnksr2	-	157872105-157872153:157910117-157910250:157919746-157919892:15793	295	282	0.349	0.6057	-0.257	0.257	0.000386947	0.00699
RI	chr2	Rbm39	-	156177336-156177385:156177336-156178952:156178880-156178952	298	149	0.4307	0.1733	0.257	0.257	0	0
SE	chr3	Syt6	+	103626982-103627132:103630902-103630969:103632447-103635178	216	149	0.9237	0.6663	0.257	0.257	9.55125E-13	9.92E-11
SE	chr16	Tvp23a	-	10427383-10427472:10428659-10428803:10446930-10447009	293	149	0.9257	0.669	0.257	0.257	2.27596E-14	3.02E-12
SE	chr16	Chrd	+	20735746-20735893:20736264-20736383:20736909-20737065	268	149	0.7973	0.54	0.257	0.257	0.000578401	0.007527
SE	chr17	Adamts10	+	33544955-33545057:33545304-33545437:33545542-33545665	282	149	0.8763	0.6197	0.257	0.257	1.27324E-10	8.92E-09
SE	chr8	Dctd	+	48137258-48137374:48138179-48138878:48140343-48141667	298	149	0.99	0.7333	0.257	0.257	3.23429E-09	1.78E-07
SE	chr4	Anks6	-	47023744-47023860:47024506-47024679:47025686-47025753	298	149	0.7073	0.4507	0.257	0.257	0.005183669	0.047606
A3SS	chr7	Hps5	-	46779072-46779160:46778021-46778199:46778021-46778100	247	149	0.8417	0.586	0.256	0.256	0.000802881	0.012705
RI	chr2	Qsox2	-	26209124-26209793:26209124-26211459:26211274-26211459	298	149	0.871	0.6147	0.256	0.256	3.87884E-06	5.08E-05
RI	chr16	Vwa5b2	+	20589468-20590281:20589468-20590898:20590673-20590898	298	149	0.878	0.622	0.256	0.256	5.69612E-05	0.000531
SE	chr7	Tmem80	+	141328130-141328266:141331418-141331561:141332910-141332929	292	149	0.601	0.3453	0.256	0.256	2.22276E-07	7.94E-06
SE	chr8	Ttc13	-	124690277-124690359:124694830-124694940:124709903-124709973	259	149	0.3357	0.592	-0.256	0.256	2.18619E-07	7.82E-06
SE	chr6	Hnrnpa2b1	-	51461121-51461433:51461920-51463254:51463411-51463493	298	149	0.9807	0.7247	0.256	0.256	0	0
SE	chr11	4933427D14Rik	-	72159400-72159520:72162743-72165598:72166638-72166823	298	149	0.7443	0.4887	0.256	0.256	0.000513428	0.006775
SE	chr5	Agfg2	-	137655608-137655728:137660162-137660194:137661364-137661489	181	149	0.6947	0.4383	0.256	0.256	0.001949999	0.020894
A3SS	chr8	Atp11a	+	12859314-12859479:12861912-12868728:12863759-12868728	298	149	0.932	0.677	0.255	0.255	2.94721E-05	0.000902
MXE	chr4	Eif4g3	+	138058627-138058739:138082893-138082988:138095909-138095929:13809€	244	169	0.597	0.8523	-0.255	0.255	6.51304E-06	0.000225
RI	chr5	Aasdh	-	76873659-76876422:76873659-76877658:76877527-76877658	298	149	0.543	0.2883	0.255	0.255	0.001686139	0.009747
SE	chr8	Eps15l1	-	72345957-72346171:72358313-72358445:72367904-72367986	281	149	0.5883	0.8433	-0.255	0.255	3.34853E-10	2.18E-08
SE	chr2	Dclre1c	+	3444201-3444255:3446153-3446261:3447074-3447162	257	149	0.309	0.0543	0.255	0.255	4.24079E-10	2.73E-08
SE	chr11	Gjc1	-	102802841-102802916:102804281-102804512:102818970-102819282	298	149	0.1007	0.3557	-0.255	0.255	4.5135E-12	4.12E-10
MXE	chr11	Clk4	+	51267171-51267331:51268769-51268846:51270537-51270595:51271800-512	226	207	0.008	0.2623	-0.254	0.254	1.55431E-15	4.94E-13
MXE	chr17	3110052M02Rik	+	21651803-21652345:21657519-21657611:21658913-21659039:21661315-216	241	275	0.3903	0.644	-0.254	0.254	0.00060797	0.010094
SE	chr17	Serac1	-	6067529-6067660:6069299-6069388:6070753-6070889	238	149	0.5783	0.3247	0.254	0.254	0.004214795	0.040084
SE	chr19	Lipo3	-	33580465-33580574:33582053-33582251:33583047-33583164	298	149	0.929	0.6753	0.254	0.254	0.000557599	0.007295
SE	chr7	Sez6l2	+	126967014-126967205:126967707-126967745:126967927-126968044	187	149	0.6373	0.3837	0.254	0.254	1.17684E-14	1.65E-12
A5SS	chr7	Fam160a2	-	105384988-105385151:105384946-105385151:105383781-105384572	190	149	0.4297	0.1763	0.253	0.253	8.14289E-07	3.17E-05
A5SS	chrX	Wnk3	+	151293810-151293900:151293810-151294041:151294422-151294552	289	149	0.4607	0.208	0.253	0.253	8.19861E-05	0.001514
MXE	chr2	Pfkfb3	-	11471430-11474543:11480198-11480371:11481871-11481935:11482054-114	213	298	0.8177	0.565	0.253	0.253	7.94507E-07	3.69E-05
SE	chr14	Zc3h13	+	75284305-75284665:75293891-75294002:75305789-75305897	260	149	0.944	0.691	0.253	0.253	4.85845E-06	0.000121
SE	chr2	Metap1d	+	71453319-71453398:71500779-71500869:71506785-71506942	239	149	0.16	0.4127	-0.253	0.253	4.60135E-08	1.92E-06
SE	chr1	Clasp1	+	118525297-118525455:118536135-118536197:118539266-118539369	211	149	0.6233	0.3707	0.253	0.253	3.50702E-06	9.09E-05
SE	chr2	Mllt10	+	18071125-18071204:18092168-18092834:18109782-18109891	298	149	0.5477	0.801	-0.253	0.253	0.00181282	0.019647
SE	chr17	Rnps1	+	24414675-24414734:24415041-24415162:24418457-24418612	270	149	0.5957	0.3423	0.253	0.253	1.08593E-05	0.000246
SE	chr14	Uggt2	-	119008063-119008147:119008721-119008811:119008999-119009094	239	149	0.28	0.0267	0.253	0.253	2.27229E-12	2.2E-10
MXE	chr1	Rps6kc1	-	190783564-190783646:190799013-190800581:190844487-190844602:19087'	264	298	0.9747	0.7227	0.252	0.252	2.55962E-12	4.76E-10
RI	chr6	Magi1	-	93675453-93679002:93675453-93680877:93680789-93680877	298	149	0.745	0.4933	0.252	0.252	0.000166182	0.001329
SE	chr6	Ctnna2	-	76881637-76882837:76902287-76902430:76915734-76915868	292	149	0.8117	0.5597	0.252	0.252	0	0
SE	chr8	Adgrl1	+	83923019-83923108:83924206-83924355:83928668-83928891	298	149	0.703	0.9553	-0.252	0.252	7.63449E-07	2.41E-05
SE	chr19	Fam178a	+	44941459-44942426:44942882-44942939:44943449-44943519	206	149	0.663	0.4107	0.252	0.252	4.30562E-07	1.43E-05
SE	chr8	Polb	-	22642305-22642356:22645045-22645094:22650489-22650555	198	149	0.6	0.8523	-0.252	0.252	8.98896E-05	0.001524
SE	chr5	Adgrl3	+	81771584-81771680:81792361-81792378:81794264-81795730	166	149	0.532	0.2803	0.252	0.252	1.3463E-05	0.000297
SE	chr1	2310035C23Rik	+	105750322-105750410:105750973-105751043:105753503-105755121	219	149	0.9877	0.7357	0.252	0.252	0	0
A5SS	chr2	Rbm43	-	51934939-51935009:51934832-51935009:51932400-51932523	255	149	0.8983	0.647	0.251	0.251	0.000118909	0.002067
A5SS	chr9	Mto1	+	78461520-78461594:78461520-78461691:78464884-78465002	245	149	0.7667	0.516	0.251	0.251	0.000110336	0.001953
MXE	chr11	Clk4	+	51267171-51267331:51268165-51268231:51270537-51270595:51271800-512	215	207	0.3737	0.6247	-0.251	0.251	3.14782E-05	0.00086
RI	chr13	Ankra2	+	98272772-98272852:98272772-98273918:98273378-98273918	298	149	0.501	0.2503	0.251	0.251	3.44169E-15	2.21E-13
RI	chr8	Cdh8	-	99028769-99029911:99028769-99031058:99030876-99031058	298	149	0.7433	0.4927	0.251	0.251	1.02368E-08	2.39E-07
SE	chr18	Wdr7	+	63779869-63779957:63791796-63791891:63794225-63794343	244	149	0.6317	0.381	0.251	0.251	8.4238E-06	0.000197
SE	chr2	Gm14322	+	177762846-177762900:177767739-177767775:177768044-177768170	185	149	0.9413	0.6907	0.251	0.251	3.01556E-08	1.32E-06
SE	chr12	E2f6	+	16810903-16811315:16813847-16813912:16816400-16816454	214	149	0.434	0.1833	0.251	0.251	1.64793E-05	0.000353
SE	chr16	Ccdc50	+	27435562-27435677:27436368-27436412:27436498-27436602	193	149	0.9257	0.6743	0.251	0.251	5.55112E-16	9.03E-14
SE	chr2	Cacna1b	-	24622599-24622707:24625238-24625334:24631899-24632026	245	149	0.725	0.4737	0.251	0.251	1.65288E-06	4.74E-05
SE	chr2	Ptprt	-	161656142-161656290:161687069-161687098:161697974-161698109	178	149	0.5537	0.8043	-0.251	0.251	0.000693663	0.008774
SE	chr15	BC024139	-	76124573-76124781:76125062-76125146:76125215-76125365	233	149	0.64	0.891	-0.251	0.251	8.29812E-06	0.000194
SE	chr2	Fam188a	-	12386609-12386679:12396179-12396258:12419159-12419460	228	149	0.585	0.8357	-0.251	0.251	1.2885E-06	3.81E-05
SE	chr19	Rps6kb2	-	4162603-4162723:4162822-4162862:4163168-4163213	189	149	0.928	0.6773	0.251	0.251	1.73195E-14	2.35E-12
SE	chr8	Wdr17	-	54685396-54685518:54687526-54687726:54690204-54690234	298	149	1	0.749	0.251	0.251	9.23585E-08	3.58E-06
SE	chr7	Brsk2	+	141998920-141999014:142000842-142000889:142002063-142004244	196	149	0.363	0.614	-0.251	0.251	0.000100407	0.001673
MXE	chr10	Mdm1	+	118146595-118146971:118147981-118148115:118150822-118150956:11815	283	283	0.611	0.3607	0.25	0.25	0.000481483	0.008438
RI	chr11	Lrrc45	+	120718442-120718553:120718442-120719132:120719040-120719132	298	149	0.83	0.5797	0.25	0.25	4.4942E-09	1.14E-07

SE	chr11	Adam19	+	46099853-46099929:46112757-46112949:46113623-46113688	298	149	0.967	0.7167	0.25	0.25	1.0192E-10	7.22E-09
SE	chr11	Hdac5	-	102218416-102218490:102219145-102219281:102224734-102224955	285	149	0.1353	0.3857	-0.25	0.25	1.31441E-06	3.88E-05
SE	chrX	Gprasp1	+	135751945-135752017:135796949-135797037:135797553-135797629	237	149	0.4753	0.7257	-0.25	0.25	1.86674E-09	1.06E-07
SE	chr7	Ube3a	+	59229004-59229271:59243350-59243469:59247173-59247217	268	149	0.238	0.4877	-0.25	0.25	8.32579E-09	4.16E-07
SE	chr15	Cdh9	+	16823159-16823453:16828344-16828463:16828942-16829109	268	149	0.9483	0.6983	0.25	0.25	8.32087E-07	2.6E-05
SE	chr5	Preb	-	30951667-30955737:30955930-30956089:30957888-30958061	298	149	0.93	0.68	0.25	0.25	6.49902E-10	4.03E-08
MXE	chr16	Gnb1l	+	18499046-18499140:18548093-18548191:18552367-18552582:18564035-185	247	298	0.0657	0.3143	-0.249	0.249	0.000183975	0.003785
RI	chr15	Taf2	-	55015129-55016153:55015129-55016601:55016373-55016601	298	149	0.82	0.5707	0.249	0.249	3.78453E-12	1.61E-10
SE	chr2	Oprl1	+	181715690-181715946:181717955-181718093:181718377-181718732	287	149	0.338	0.5867	-0.249	0.249	6.03449E-10	3.77E-08
SE	chr6	Zfp384	+	125024746-125025082:125025848-125025940:125030798-125031022	241	149	0.5253	0.276	0.249	0.249	7.48281E-09	3.78E-07
SE	chr10	Pex7	-	19894250-19894358:19894725-19894802:19904563-19904713	226	149	0.9243	0.6757	0.249	0.249	5.44966E-08	2.23E-06
SE	chr11	Ncor1	-	62345235-62345318:62349395-62349493:62351378-62351546	247	149	0.346	0.5947	-0.249	0.249	2.38913E-05	0.000489
SE	chr1	Fn1	-	71612468-71612650:71613763-71614035:71615360-71615551	298	149	0.7383	0.489	0.249	0.249	1.7787E-07	6.51E-06
SE	chr9	Map4	+	110063017-110063123:110064228-110064449:110067485-110067668	298	149	0.7363	0.487	0.249	0.249	0	0
SE	chr8	Vps9d1	-	123245114-123245210:123245482-123245712:123245944-123246073	298	149	0.9617	0.7127	0.249	0.249	1.24345E-14	1.73E-12
SE	chr4	Usp1	+	98923810-98924243:98926436-98926674:98928234-98928354	298	149	0.738	0.489	0.249	0.249	2.29683E-07	8.17E-06
SE	chr16	Alg3	-	20608742-20608889:20608987-20609086:20610543-20610749	248	149	0.8967	0.6473	0.249	0.249	1.71962E-05	0.000366
RI	chr3	Gria2	-	80691320-80691434:80691320-80692532:80692285-80692532	298	149	0.6353	0.387	0.248	0.248	0	0
RI	chr2	Kcnb1	-	167095969-167100815:167095969-167106359:167101093-167106359	298	149	0.6097	0.3617	0.248	0.248	0.000105108	0.000907
SE	chr14	Vwa8	+	78994400-78994568:78995086-78995163:79009164-79009279	226	149	0.9627	0.715	0.248	0.248	1.26687E-06	3.76E-05
SE	chr16	Anks3	-	4941432-4941797:4941899-4941982:4942211-4942294	232	149	0.617	0.369	0.248	0.248	0.001981382	0.021192
SE	chr2	Ninl	-	150963355-150963495:150964134-150964270:150966078-150966242	285	149	0.894	0.646	0.248	0.248	0.002008853	0.021439
SE	chrX	Armcx5	+	135742692-135742978:135743563-135743614:135744499-135744575	200	149	0.377	0.1287	0.248	0.248	1.36138E-09	7.95E-08
SE	chr18	8030462N17Rik	-	77643068-77643194:77653129-77653296:77673900-77674684	298	149	0.3303	0.5787	-0.248	0.248	6.2661E-05	0.001117
SE	chr5	Evi5	-	107799126-107799260:107809590-107809736:107810290-107810322	295	149	0.9047	0.6563	0.248	0.248	6.87959E-07	2.2E-05
SE	chr11	Tom1l2	-	60231101-60231137:60232898-60232957:60241818-60241893	208	149	0.8513	0.603	0.248	0.248	3.06464E-09	1.69E-07
A3SS	chr12	Nrxn3	+	90283572-90283650:90331485-90334935:90331788-90334935	298	149	0.569	0.8157	-0.247	0.247	3.58718E-07	2.1E-05
A5SS	chr3	Gria2	-	80692285-80692532:80691703-80692532:80691320-80691434	298	149	0.5193	0.272	0.247	0.247	0	0
A5SS	chr1	Speg	+	75384828-75384987:75384828-75385164:75387798-75389104	298	149	0.5447	0.298	0.247	0.247	0.000140275	0.00233
A5SS	chr16	Vwa5b2	+	20589468-20590281:20589468-20590531:20590673-20590898	298	149	0.8543	0.6077	0.247	0.247	0.000280847	0.00413
MXE	chr5	Gabra2	-	71007923-71008066:71012701-71012783:71034730-71034797:71091972-710	216	231	0.4327	0.68	-0.247	0.247	0.000666694	0.010859
RI	chr10	Ankrd24	+	81635688-81635752:81635688-81636426:81636369-81636426	298	149	0.462	0.2147	0.247	0.247	5.59479E-05	0.000526
SE	chr7	Abcc8	-	46107238-46108435:46108753-46108866:46109541-46109643	262	149	0.9023	0.6557	0.247	0.247	3.32395E-05	0.000651
SE	chr16	Grik1	-	87896259-87896351:87896689-87896895:87914154-87914404	298	149	0.2897	0.043	0.247	0.247	6.98326E-11	5.1E-09
SE	chr7	Mef2a	-	67240314-67240440:67244521-67244544:67251657-67251844	172	149	0.86	0.613	0.247	0.247	3.33067E-16	5.56E-14
SE	chr2	Snph	-	151597109-151597258:151600257-151600364:151600978-151601074	256	149	0.1677	0.4143	-0.247	0.247	0.000129709	0.002079
SE	chr18	Csnk1a1	+	61580339-61580487:61584205-61584404:61585165-61590315	298	149	0.9783	0.7317	0.247	0.247	1.70526E-07	6.26E-06
SE	chr7	Mir9-3hg	+	79500026-79500250:79523917-79523976:79527763-79527869	208	149	0.4227	0.6693	-0.247	0.247	0.000377408	0.005191
SE	chr8	Wdr17	-	54685396-54685518:54687526-54687732:54690004-54690234	298	149	1	0.753	0.247	0.247	9.74231E-08	3.76E-06
SE	chr9	Zfp26	-	20442180-20442265:20444259-20444385:20444894-20444981	275	149	1	0.7533	0.247	0.247	8.69803E-11	6.22E-09
A3SS	chr8	Atp11a	+	12859314-12859479:12863759-12868728:12864920-12868728	298	149	0.9697	0.7237	0.246	0.246	1.48919E-06	6.98E-05
SE	chr8	Ank1	+	23123832-23123978:23131438-23131554:23132097-23132184	265	149	0.5573	0.3117	0.246	0.246	0.000210759	0.00316
SE	chr2	Ralgapb	+	158448320-158448449:158449489-158449497:158450177-158450310	157	149	0.3957	0.1497	0.246	0.246	0.000543717	0.007137
SE	chr17	Ptprs	-	56427939-56428132:56428852-56428966:56434453-56434597	263	149	0.985	0.7393	0.246	0.246	0	0
MXE	chr12	Myt1l	+	29783855-29783888:29811373-29811734:29826866-29827837:29832288-298	298	298	0.2557	0.5003	-0.245	0.245	6.57938E-08	4.29E-06
MXE	chr15	Pou6f1	-	100580810-100580951:100584031-100584159:100585875-100586125:10058	298	277	0.8003	0.5557	0.245	0.245	0.000348714	0.006437
SE	chr4	Nfib	-	82310303-82310391:82323545-82323710:82327729-82327913	298	149	0.3117	0.5567	-0.245	0.245	2.87377E-05	0.000576
SE	chr5	Crlf2	-	109555273-109555357:109555507-109555627:109556019-109556181	269	149	0.8847	0.6393	0.245	0.245	8.50519E-08	3.33E-06
SE	chr9	Mthfsl	-	88688605-88688985:88715488-88715749:88719659-88719798	298	149	0.549	0.7937	-0.245	0.245	0.000258701	0.003768
MXE	chr5	Mphosph9	-	124315373-124315896:124316082-124316171:124320845-124320998:12432	298	238	0.6313	0.8757	-0.244	0.244	3.32593E-05	0.000896
SE	chr11	Sfi1	-	3133072-3133207:3135283-3135542:3135654-3135830	298	149	0.9003	0.6563	0.244	0.244	0.002639983	0.027101
SE	chr2	Fmnl2	+	53108252-53108458:53112277-53112297:53114112-53114236	169	149	0.8007	0.557	0.244	0.244	1.40405E-05	0.000308
SE	chr1	Vangl2	-	172013083-172013345:172022477-172022586:172026591-172026760	258	149	0.3827	0.627	-0.244	0.244	8.70559E-05	0.001484
SE	chr2	Ergic3	+	156011349-156011406:156015377-156015391:156015989-156016020	163	149	0.8247	0.581	0.244	0.244	0	0
SE	chr2	Rif1	+	52072820-52073253:52074247-52074325:52076081-52076177	227	149	0.6913	0.9353	-0.244	0.244	1.2262E-05	0.000274
SE	chr4	Oscp1	+	126082882-126082985:126083412-126083724:126084393-126084462	298	149	0.9647	0.721	0.244	0.244	1.83427E-09	1.04E-07
SE	chr15	Fam49b	-	63950006-63950127:63957585-63957667:63970930-63970968	231	149	0.7217	0.478	0.244	0.244	2.05607E-07	7.41E-06
A5SS	chr11	Zkscan17	-	59503677-59503810:59503384-59503810:59502920-59503100	298	149	0.9193	0.6763	0.243	0.243	2.07074E-07	1.05E-05
MXE	chr2	Commd9	+	101886262-101886319:101897040-101897179:101898868-101898902:10189	288	183	0.0797	0.323	-0.243	0.243	6.12354E-09	4.91E-07
RI	chr11	Sfi1	-	3131884-3132131:3131884-3132327:3132216-3132327	232	149	0.579	0.822	-0.243	0.243	6.56379E-06	8.17E-05
RI	chr5	Gigyf1	+	137518482-137518612:137518482-137518753:137518688-137518753	223	149	0.932	0.689	0.243	0.243	1.3195E-06	1.99E-05
RI	chr5	Pan3	+	147545606-147545735:147545606-147548500:147546365-147548500	298	149	0.692	0.4493	0.243	0.243	0.000414785	0.002908
SE	chr14	Rarb	-	16548414-16548562:16618705-16618785:16818690-16819151	229	149	0.5193	0.7623	-0.243	0.243	0.001116887	0.013088
SE	chr18	Usp14	-	10016541-10016644:10017991-10018095:10022818-10022850	253	149	0.6073	0.8503	-0.243	0.243	0	0
SE	chr6	Zfp239	+	117863077-117863142:117869218-117869273:117870256-117872766	204	149	0.6633	0.9063	-0.243	0.243	0.000154505	0.002417
SE	chr15	Prpf40b	+	99304425-99304490:99305166-99305193:99305408-99305433	176	149	0.905	0.662	0.243	0.243	2.27485E-13	2.62E-11
A3SS	chr1	Nvl	-	181097136-181097224:181094984-181095689:181094984-181095054	298	149	0.3783	0.1367	0.242	0.242	1.78502E-12	3.79E-10
A5SS	chr11	Mllt6	+	97672457-97672592:97672457-97672682:97673460-97674071	238	149	0.288	0.0463	0.242	0.242	1.52685E-11	1.96E-09
A5SS	chr11	Nt5c3b	-	100437974-100438161:100436714-100438161:100436155-100436240	298	149	0.8493	0.6077	0.242	0.242	0.000695896	0.008574
MXE	chr16	Tmem44	-	30543677-30543840:30545126-30545219:30547343-30547469:30550363-305	275	242	0.202	0.444	-0.242	0.242	6.55818E-09	5.23E-07
SE	chr3	Dclk1	+	55516873-55516986:55521850-55521923:55533570-55539061	222	149	0.172	0.414	-0.242	0.242	0	0
SE	chr15	Poldip3	-	83135294-83135389:83137468-83137554:83138132-83138522	235	149	0.803	0.5613	0.242	0.242	3.01059E-12	2.83E-10
SE	chr12	Meg3	+	109544027-109544742:109544948-109545041:109546430-109546542	242	149	0.211	0.453	-0.242	0.242	0	0
SE	chr16	Pam16	-	4617126-4617262:4617919-4618003:4624851-4624946	233	149	0.9353	0.6937	0.242	0.242	0	0
SE	chr2	Pkp4	+	59347924-59348041:59350494-59350622:59352262-59352335	277	149	0.899	0.6567	0.242	0.242	0	0
A5SS	chr8	Tmem208	+	105328322-105328458:105328322-105328484:105328772-105329057	174	149	0.757	0.516	0.241	0.241	4.90154E-05	0.000998
RI	chr11	Zfp207	+	80395390-80395573:80395390-80395875:80395792-80395875	298	149	0.8187	0.578	0.241	0.241	0	0
RI	chr19	Chka	+	3874769-3874798:3874769-3875788:3875735-3875788	298	149	0.794	0.5527	0.241	0.241	0.000402169	0.002828
SE	chr9	Hemk1	-	107329324-107329432:107336179-107336272:107336572-107336663	242	149	0.818	0.577	0.241	0.241	0.002155257	0.022772
SE	chr1	R3hdm1	+	128231323-128231467:128234994-128235148:128235542-128235626	298	149	1	0.759	0.241	0.241	3.21965E-15	4.89E-13
SE	chr4	Ptprf	-	118217092-118217189:118218012-118218044:118220493-118220608	181	149	0.8313	0.59	0.241	0.241	5.58137E-08	2.28E-06
SE	chr16	Top3b	+	16870719-16871055:16875072-16875155:16877832-16878016	232	149	0.1127	0.3537	-0.241	0.241	4.11336E-05	0.000779
SE	chr2	Snph	-	151597109-151597258:151600257-151600463:151600978-151601074	298	149	0.244	0.485	-0.241	0.241	0.000890757	0.010844
RI	chr1	Qsox1	-	155778155-155778851:155778155-155779938:155779718-155779938	298	149	0.6023	0.362	0.24	0.24	1.40172E-05	0.000156
RI	chr4	Fam219a	-	41521867-41521969:41521867-41523988:41523915-41523988	298	149	0.9323	0.6927	0.24	0.24	8.65092E-08	1.77E-06
RI	chr8	Rtbdn	+	84949882-84949956:84949882-84950676:84950502-84950676	298	149	0.5177	0.2777	0.24	0.24	0.002140052	0.011877
RI	chr5	Gigyf1	+	137518482-137518753:137518482-137518986:137518878-137518986	272	149	0.8633	0.6233	0.24	0.24	2.29163E-05	0.000244
SE	chr15	Chkb	-	89428134-89428229:89428411-89428544:89428714-89428827	282	149	0.9787	0.739	0.24	0.24	0	0
SE	chr5	Rundc3b	-	8490336-8492589:8512261-8512407:8520859-8521016	295	149	0.5877	0.828	-0.24	0.24	0.000440776	0.005952
SE	chr2	Ppp1r26	+	28447885-28447982:28448692-28448908:28450302-28455508	298	149	0.5937	0.3537	0.24	0.24	0.003169952	0.03156
SE	chr9	Mlip	-	77102084-77102393:77138350-77138556:77164765-77164893	298	149	0.262	0.5017	-0.24	0.24	0.001790639	0.019474
SE	chr11	Cbx1	+	96802728-96802822:96806616-96806764:96807803-96808149	297	149	0.9323	0.6927	0.24	0.24	0	0
SE	chr17	U2af1	-	31648845-31648894:31652502-31652568:31654979-31655066	215	149	0.958	0.718	0.24	0.24	7.01661E-14	8.73E-12
SE	chr16	Tnk2	+	32677894-32677985:32678669-32678713:32679459-32680807	193	149	0.7887	0.5487	0.24	0.24	1.726E-09	9.86E-08
SE	chr16	Thap7	-	17527959-17528740:17528935-17529075:17530230-17530385	289	149	0.7323	0.492	0.24	0.24	1.28859E-07	4.85E-06
SE	chrX	Ogt	+	101640051-101640322:101642976-101643156:101643947-101644190	298	149	0.986	0.7457	0.24	0.24	0	0
MXE	chr9	Scamp5	-	57446072-57446173:57451356-57451484:57451980-57452034:57467933-574	203	277	0.9217	0.683	0.239	0.239	5.5018E-10	5.95E-08
MXE	chr9	Pkm	+	59671921-59672073:59675029-59675195:59675568-59675734:59678044-596	298	298	0.7747	0.5357	0.239	0.239	0	0
SE	chr11	Cpeb4	+	31908825-31908875:31918801-31918824:31920409-31920582	172	149	0.9023	0.6633	0.239	0.239	5.30116E-09	2.77E-07
SE	chr13	Mocs2	+	114818237-114818323:114819336-114819457:114820802-114820946	270	149	0.7803	0.541	0.239	0.239	2.22045E-16	3.82E-14
SE	chr5	Gtf2i	-	134274539-134274595:134276372-134276428:134279437-134279514	205	149	0.2147	0.4537	-0.239	0.239	3.42504E-13	3.82E-11
SE	chr12	Mta1	+	113116988-113117081:113120211-113120261:113120827-113120953	199	149	0.5953	0.3563	0.239	0.239	2.19769E-12	2.14E-10
SE	chr3	Ctbs	+	146450467-146450630:146454347-146454485:146454948-146455156	287	149	0.9153	0.676	0.239	0.239	0.000231623	0.003427
SE	chr5	Wdfy3	-	101930828-101931005:101935990-101936073:101937282-101937739	232	149	0.8897	0.6507	0.239	0.239	1.35849E-06	4E-05
A3SS	chr11	Hdac5	-	102219145-102219281:102218416-102218953:102218416-102218487	298	149	0.864	0.626	0.238	0.238	2.34585E-05	0.000769
A3SS	chr2	Rbm39	-	156177336-156177385:156172828-156174974:156172828-156174168	298	149	0.779	0.5413	0.238	0.238	1.40074E-05	0.0005
A3SS	chr16	Vwa5b2	+	20589468-20590281:20590470-20590898:20590673-20590898	298	149	0.8297	0.5913	0.238	0.238	0.001291116	0.018563
A5SS	chr17	Slc25a27	-	43666900-43667190:43664078-43667190:43661614-43661698	298	149	0.966	0.728	0.238	0.238	1.22422E-11	1.61E-09
A5SS	chr5	Actl6b	+	137553540-137553659:137553540-137553783:137554996-137555096	272	149	0.9453	0.707	0.238	0.238	2.6469E-07	1.29E-05
A5SS	chr14	Khnyn	+	55893992-55894099:55893992-55894159:55894287-55894388	208	149	0.4503	0.2127	0.238	0.238	0.002436304	0.023114
A5SS	chrX	Atrx	-	105929100-105929406:105928959-105929406:105911470-105911654	289	149	0.877	0.639	0.238	0.238	2.22455E-12	3.73E-10
MXE	chr17	Tulp4	+	6106828-6106996:6121828-6122344:6137211-6139156:6198672-6198852	298	298	0.3117	0.5493	-0.238	0.238	1.48352E-09	1.4E-07
RI	chr2	Frmd5	-	121545529-121548023:121545529-121549232:121548889-121549232	298	149	0.8873	0.6497	0.238	0.238	2.24106E-09	5.84E-08
SE	chr4	Nasp	-	116605684-116605763:116610403-116611377:116611972-116612081	298	149	0.389	0.1507	0.238	0.238	0	0
SE	chr7	Eef2k	+	120873328-120873409:120879814-120879851:120880305-120880476	186	149	1	0.7623	0.238	0.238	1.50569E-08	7.04E-07
SE	chr5	Iqce	-	140691600-140691670:140692794-140692928:140693415-140693543	283	149	0.4323	0.1947	0.238	0.238	3.7986E-06	9.75E-05
SE	chr4	Ube2j2	+	155955234-155955336:155955422-155955560:155956368-155956448	287	149	0.9557	0.7177	0.238	0.238	0	0
SE	chr11	Hdac5	-	102218416-102218487:102219145-102219281:102224734-102224955	285	149	0.0653	0.3037	-0.238	0.238	0	0
SE	chr7	Tacc2	+	130753618-130753661:130755897-130755905:130759165-130759325	157	149	0.898	0.6603	0.238	0.238	0	0
SE	chr5	Ssh1	-	113958714-113958908:113960600-113960665:113961350-113961418	214	149	0.9917	0.7537	0.238	0.238	1.00527E-09	6E-08
SE	chr10	Jmjd1c	+	67215270-67215375:67218117-67218181:67218277-67218469	213	149	0.9563	0.7187	0.238	0.238	9.71583E-07	2.98E-05
SE	chr7	Usp29	+	6738793-6738945:6908286-6908385:6927452-6927516	248	149	0.1733	0.411	-0.238	0.238	8.08193E-05	0.001391
MXE	chr12	Acp1	-	30897745-30897806:30904718-30904831:30904870-30904983:30905069-309	262	262	0.447	0.2097	0.237	0.237	0	0
MXE	chr5	Rpap2	+	107625888-107625968:107633063-107633131:107655157-107655310:10766	217	298	0.3907	0.6273	-0.237	0.237	0.000104884	0.002345

SE	chr16	Dnm1l	-	16316602-16316778:16318445-16318477:16319452-16319508	181	149	0.945	0.708	0.237	0.237	2.6823E-13	3.06E-11
A3SS	chr9	Mrpl4	+	21006827-21006856:21007264-21007540:21007364-21007540	248	149	0.9347	0.6983	0.236	0.236	8.08378E-05	0.002051
MXE	chr3	Pogz	+	94856043-94856201:94860796-94860971:94862422-94862530:94864485-948	298	257	0.9687	0.7327	0.236	0.236	3.10862E-14	7.77E-12
RI	chr8	Hook2	+	84998204-84998291:84998204-84998391:84998386-84998391	242	149	0.5797	0.8157	-0.236	0.236	1.10289E-05	0.000127
SE	chr16	Synj1	-	90969183-90969374:90971564-90971587:90973910-90974066	172	149	0.9493	0.7133	0.236	0.236	2.59903E-13	2.97E-11
SE	chr13	Kif2a	-	106968638-106968788:106969704-106969817:106974495-106974562	262	149	0.8907	0.6543	0.236	0.236	2.23155E-14	2.98E-12
SE	chr6	Rad52	+	119902198-119902835:119915967-119916042:119918587-119918786	224	149	0.647	0.8833	-0.236	0.236	0.001032827	0.012271
SE	chr16	Vps8	+	21448349-21448403:21451356-21451361:21456229-21456353	154	149	0.6587	0.4227	0.236	0.236	0.00337691	0.033324
SE	chr3	4932438A13Rik	+	36989795-36989888:36992677-36992727:36994775-36994981	199	149	0.9867	0.7517	0.235	0.235	1.00142E-13	1.21E-11
SE	chr1	Map2	+	66401444-66401521:66416735-66416866:66420138-66420196	280	149	0.646	0.881	-0.235	0.235	6.64139E-09	3.4E-07
SE	chr19	Stx3	-	11789557-11789631:11790048-11790093:11791746-11791845	194	149	0.5973	0.362	0.235	0.235	0.001298287	0.014902
SE	chr14	Slmap	-	26413168-26414988:26416203-26416292:26418113-26418247	238	149	0.6357	0.4007	0.235	0.235	0.000222415	0.003307
SE	chr14	Fermt2	-	45462116-45462240:45462715-45462735:45464724-45464945	169	149	0.252	0.0173	0.235	0.235	0	0
SE	chr4	Ubap2	-	41235517-41235594:41245456-41245492:41251535-41251671	185	149	0.1167	0.352	-0.235	0.235	2.35034E-13	2.71E-11
SE	chr8	Rnf170	+	26129047-26129155:26135629-26135702:26138980-26139090	222	149	0.9613	0.7267	0.235	0.235	9.22482E-09	4.57E-07
SE	chr3	Slc44a5	+	154265459-154265543:154267677-154268620:154269983-154270047	298	149	0.735	0.5	0.235	0.235	0.000592011	0.007675
SE	chr9	Mthfsl	-	88688605-88688985:88715488-88715749:88719489-88719605	298	149	0.615	0.8497	-0.235	0.235	6.19404E-05	0.001107
A5SS	chr2	Baz2b	-	59907544-59907749:59907439-59907749:59906916-59907051	253	149	0.5783	0.3443	0.234	0.234	1.01175E-05	0.000263
A5SS	chr8	Pard6a	+	105701148-105701286:105701148-105701317:105702174-105702396	179	149	0.433	0.199	0.234	0.234	0.002211154	0.021521
A5SS	chr5	Mtf2	+	108069162-108069363:108069162-108069523:108080825-108081023	298	149	0.5113	0.2777	0.234	0.234	0.000173578	0.002761
MXE	chr11	Aftph	-	20692465-20692588:20696751-20696831:20698313-20698373:20703711-207	209	229	0.527	0.7613	-0.234	0.234	1.14618E-05	0.000357
SE	chr18	Spata24	-	35660444-35660574:35660792-35660891:35662012-35662031	248	149	0.9743	0.7403	0.234	0.234	5.21126E-08	2.15E-06
SE	chr10	Uhrf1bp1l	+	89745002-89745288:89755421-89755554:89772587-89772749	282	149	0.08	0.314	-0.234	0.234	8.88178E-16	1.42E-13
SE	chr11	Mbtd1	+	93933881-93933958:93939521-93939712:93943662-93943864	298	149	0.2627	0.497	-0.234	0.234	0.001431825	0.016146
SE	chr7	2410002F23Rik	+	44246720-44246808:44247251-44247416:44248252-44248295	298	149	0.518	0.2837	0.234	0.234	7.54808E-06	0.000179
SE	chr15	Zfp740	+	102208236-102208361:102208753-102208876:102209155-102209273	272	149	0.903	0.6693	0.234	0.234	1.88738E-14	2.56E-12
SE	chr9	Edc3	+	57713381-57713561:57715948-57716267:57727186-57727521	298	149	0.735	0.5007	0.234	0.234	2.81742E-09	1.57E-07
SE	chr9	Pfkfb4	+	108992022-108992112:108998842-108998975:108999113-108999209	282	149	0.9877	0.754	0.234	0.234	2.35489E-09	1.33E-07
SE	chr8	Sorbs2	+	45775589-45775703:45785269-45785325:45789976-45790025	205	149	0.2683	0.502	-0.234	0.234	9.64859E-05	0.001618
SE	chr8	Tmco3	+	13313806-13313956:13314558-13314650:13318843-13318922	241	149	0.133	0.367	-0.234	0.234	0.000313423	0.004433
A3SS	chr5	Aasdh	-	76877527-76877658:76873659-76876678:76873659-76876422	298	149	0.4383	0.205	0.233	0.233	0.000549383	0.009439
MXE	chr5	Zfp644	-	106619540-106619642:106635610-106638635:106666785-106666845:10669(	209	298	0.1667	0.4	-0.233	0.233	0.000600615	0.009997
SE	chr12	Rian	+	109640267-109640328:109642565-109642652:109645262-109645331	236	149	0.99	0.7567	0.233	0.233	0	0
SE	chr4	Arhgef10l	-	140583824-140583932:140586784-140586900:140591818-140591987	265	149	0.7427	0.5097	0.233	0.233	2.55252E-05	0.000518
SE	chrX	Gk	-	85701937-85704396:85706238-85706324:85711051-85711131	235	149	0.89	0.6573	0.233	0.233	6.65751E-05	0.001176
SE	chr5	Rundc3b	-	8490336-8492589:8512267-8512407:8520859-8521016	289	149	0.589	0.8223	-0.233	0.233	0.00065755	0.008388
SE	chr19	Trub1	+	57485073-57485212:57487174-57487321:57488009-57488051	296	149	1	0.767	0.233	0.233	3.08018E-08	1.34E-06
SE	chr8	Ints10	+	68822157-68822250:68824190-68824267:68827277-68828643	226	149	0.3473	0.1143	0.233	0.233	4.72568E-06	0.000118
A3SS	chr11	Hdac5	-	102219145-102219281:102218416-102218781:102218416-102218487	298	149	0.8683	0.6363	0.232	0.232	2.87113E-05	0.000893
A3SS	chr2	Ralgapb	+	158448320-158448449:158450177-158450310:158450189-158450310	160	149	0.3857	0.618	-0.232	0.232	0.002324465	0.028651
A5SS	chr12	Adck1	+	88360514-88360595:88360514-88360783:88368335-88368480	298	149	0.5543	0.786	-0.232	0.232	0.004270246	0.035406
RI	chr2	Bcl2l11	+	128128620-128128756:128128620-128129014:128128925-128129014	298	149	0.8683	0.6367	0.232	0.232	0.00152162	0.009083
SE	chr4	Nfib	-	82310303-82310391:82323572-82323710:82327729-82327913	287	149	0.3207	0.553	-0.232	0.232	7.25801E-05	0.001268
SE	chr14	Pnma2	+	66911110-66911293:66914307-66914764:66915322-66915450	298	149	0.1507	0.383	-0.232	0.232	3.7501E-05	0.000719
SE	chr16	Ttc3	+	94383912-94384066:94384346-94384388:94384784-94384934	191	149	0.5787	0.347	0.232	0.232	2.86882E-13	3.25E-11
SE	chr11	B9d1	+	61506344-61506412:61509072-61509168:61509470-61509532	245	149	0.865	0.633	0.232	0.232	0.000334853	0.004687
SE	chr18	Sil1	-	35438267-35438380:35467846-35467943:35498791-35499290	246	149	0.1157	0.3473	-0.232	0.232	1.37218E-05	0.000302
SE	chr7	Whamm	+	81586134-81586299:81588418-81588552:81589338-81589525	283	149	0.2927	0.525	-0.232	0.232	0.000619306	0.007981
SE	chr7	Pde3b	+	114519512-114519649:114521664-114521775:114523333-114523446	260	149	0.988	0.7563	0.232	0.232	1.10166E-09	6.53E-08
A3SS	chr4	Sfpq	+	127027397-127027518:127029827-127035854:127035808-127035854	298	149	0.855	0.6243	0.231	0.231	0	0
MXE	chr4	Nfib	-	82290173-82296811:82310303-82310391:82320474-82320556:82323572-823	231	237	0.2423	0.473	-0.231	0.231	0.004544429	0.048316
RI	chr6	Tra2a	-	49252382-49252515:49252382-49253108:49252797-49253108	298	149	0.8553	0.6247	0.231	0.231	8.0258E-13	3.86E-11
SE	chr14	Dzip1	-	118922911-118923167:118923897-118924095:118924425-118924518	298	149	0.8503	0.6197	0.231	0.231	6.78294E-11	4.97E-09
SE	chr13	4833439L19Rik	-	54559198-54559324:54564185-54564272:54565182-54565375	236	149	0.4927	0.7233	-0.231	0.231	0.000171958	0.002655
A3SS	chr11	Cant1	-	118418930-118419118:118417574-118417806:118417574-118417654	298	149	0.538	0.7683	-0.23	0.23	1.59158E-05	0.000547
A5SS	chr9	Prkcsh	+	22011438-22011575:22011438-22011596:22011674-22011770	169	149	0.5973	0.3673	0.23	0.23	1.81049E-05	0.000431
A5SS	chr7	Ccdc9	-	16275280-16275375:16275256-16275375:16274042-16274416	172	149	0.761	0.5313	0.23	0.23	0.000150565	0.002476
MXE	chr3	Gria2	-	80682904-80689351:80690404-80690518:80691320-80691743:80692285-806	298	263	0.1453	0.3757	-0.23	0.23	0	0
MXE	chr14	Camk2g	-	20744604-20744648:20747819-20747851:20755659-20755702:20760144-207	192	181	0.6477	0.8773	-0.23	0.23	4.21885E-15	1.27E-12
MXE	chr11	Adamts2	+	50667991-50668150:50737166-50737368:50756696-50756779:50773217-507	298	232	0.6137	0.384	0.23	0.23	0.003494271	0.039764
RI	chr12	LOC108167368	+	20664495-20664888:20664495-20665076:20665021-20665076	280	149	0.908	0.6783	0.23	0.23	0.000128681	0.001067
RI	chr1	Sf3b1	-	55014869-55016182:55014869-55016490:55016376-55016490	298	149	0.836	0.6063	0.23	0.23	0	0
SE	chr8	Cdk10	+	123229112-123229181:123230268-123230375:123230594-123230684	256	149	0.943	0.713	0.23	0.23	1.4058E-08	6.64E-07
SE	chr7	2410002F23Rik	+	44246733-44246808:44247238-44247416:44248252-44248295	298	149	0.4967	0.2663	0.23	0.23	2.27961E-05	0.000469
SE	chr1	Map2	+	66401444-66401521:66416732-66416866:66420138-66420196	283	149	0.6527	0.8823	-0.23	0.23	1.46411E-08	6.87E-07
SE	chr9	Snx19	+	30440096-30440225:30462231-30462415:30463220-30463307	298	149	0.7053	0.4753	0.23	0.23	0.002626503	0.026983
SE	chr13	Gpbp1	-	111440632-111440816:111447896-111447955:111448933-111448999	208	149	0.6973	0.467	0.23	0.23	9.99201E-16	1.59E-13
SE	chr1	Glb1l	-	75208319-75208379:75208687-75208837:75209056-75209222	298	149	0.8623	0.6327	0.23	0.23	0.001186267	0.013791
SE	chr7	Caly	-	140073941-140074100:140081393-140081538:140082093-140082387	294	149	0.2383	0.468	-0.23	0.23	5.3016E-05	0.000972
A3SS	chr19	Ccnj	+	40844660-40844959:40845045-40845204:40845078-40845204	181	149	0.923	0.6943	0.229	0.229	1.14736E-05	0.000418
SE	chr11	Col23a1	+	51577074-51577160:51577662-51578555:51579575-51579613	298	149	0.2897	0.061	0.229	0.229	1.38417E-08	6.55E-07
SE	chr14	Mbnl2	+	120396451-120396604:120403336-120403371:120404638-120404732	184	149	0.4033	0.174	0.229	0.229	0.003389609	0.03342
SE	chr4	Tnc	-	63976446-63976568:63984460-63984732:63993027-63993299	298	149	0.9663	0.7377	0.229	0.229	2.93086E-11	2.32E-09
SE	chr7	Zfand6	-	84634239-84634409:84642286-84642437:84689746-84689879	298	149	0.0743	0.3037	-0.229	0.229	3.21632E-12	3.01E-10
SE	chr9	Lztfl1	-	123712430-123712624:123715300-123715420:123717526-123717620	269	149	0.9003	0.6713	0.229	0.229	2.75437E-06	7.37E-05
SE	chr15	Hdac7	-	97796098-97796231:97796833-97796951:97797941-97798038	267	149	0.9227	0.6937	0.229	0.229	1.21868E-11	1.03E-09
SE	chr15	Hdac7	-	97800695-97800802:97801074-97801329:97802076-97802125	298	149	0.324	0.0953	0.229	0.229	2.44593E-12	2.36E-10
SE	chr2	Pced1a	-	130422225-130422375:130422466-130422704:130422813-130422892	298	149	0.872	0.6433	0.229	0.229	0.000199267	0.003012
A3SS	chr3	Gm3764	+	88220079-88221284:88224265-88224435:88224268-88224435	151	149	0.5747	0.3463	0.228	0.228	0.000273854	0.005489
RI	chr18	Ptpn2	-	67665501-67665874:67665501-67672768:67672667-67672768	298	149	0.4263	0.198	0.228	0.228	1.49208E-10	4.85E-09
SE	chr2	Spg11	-	122083400-122083561:122084895-122085040:122086863-122086969	294	149	0.8963	0.6683	0.228	0.228	0.001759499	0.019183
SE	chr11	Rapgef6	+	54691238-54691740:54694270-54694572:54696080-54699286	298	149	0.51	0.2817	0.228	0.228	0.000429607	0.005814
SE	chr16	Ttc3	+	94403314-94403368:94409684-94409729:94410833-94410956	194	149	0.8557	0.628	0.228	0.228	0	0
SE	chr6	Ezh2	-	47540677-47540855:47541845-47541970:47542366-47542406	274	149	0.7543	0.5267	0.228	0.228	2.21767E-10	1.48E-08
SE	chr8	Sorbs2	+	45769829-45769895:45770546-45770590:45772534-45772708	193	149	0.796	0.5683	0.228	0.228	9.04294E-07	2.79E-05
SE	chr9	Kirrel3	+	35023454-35023652:35025111-35025146:35028332-35028439	184	149	0.8273	0.5997	0.228	0.228	4.59017E-09	2.46E-07
SE	chr5	Add1	+	34628415-34628513:34629139-34629172:34630470-34631885	182	149	0.6073	0.379	0.228	0.228	2.69706E-10	1.79E-08
SE	chr1	Pantr1	-	42648200-42648456:42663556-42663602:42666841-42667003	195	149	0.3067	0.535	-0.228	0.228	0	0
SE	chr1	Bpnt1	+	185348744-185348835:185349837-185349881:185352182-185352379	193	149	0.7213	0.4933	0.228	0.228	3.26542E-05	0.000641
SE	chrX	Gprasp2	+	135839034-135839262:135839802-135839872:135840393-135840469	219	149	0.946	0.7177	0.228	0.228	4.46975E-08	1.87E-06
A3SS	chr6	Dcp1b	+	119206470-119206598:119217734-119217979:119217803-119217979	217	149	0.9487	0.7213	0.227	0.227	3.8087E-05	0.001108
A3SS	chr19	Cpsf7	+	10531781-10531999:10532131-10532334:10532258-10532334	275	149	0.8253	0.5983	0.227	0.227	0.003964938	0.042639
A3SS	chr16	Nxpe3	-	55895288-55896773:55890582-55890849:55890582-55890811	186	149	0.3233	0.5507	-0.227	0.227	0.001581267	0.021533
MXE	chr14	Phf7	-	31248043-31248134:31248995-31249047:31249608-31249717:31250705-312	258	201	0.7507	0.524	0.227	0.227	0.003390007	0.038773
MXE	chr18	Usp14	-	10016541-10016644:10017991-10018095:10022818-10022850:10024534-100	181	253	0.7547	0.5273	0.227	0.227	1.22125E-15	4.05E-13
MXE	chr4	Dhrs3	+	144918651-144918794:144919230-144919504:144919841-144920079:144923	298	298	0.1423	0.3693	-0.227	0.227	0.000419473	0.007471
MXE	chr12	Dtnb	+	3772587-3772746:3773551-3773640:3774570-3774610:3779619-3779654	238	189	0.195	0.4217	-0.227	0.227	0.000109832	0.002434
SE	chr10	Pcbp3	-	76825860-76825992:76847736-76847812:76892557-76892656	225	149	0.0393	0.266	-0.227	0.227	6.5028E-12	5.8E-10
SE	chr7	2410002F23Rik	+	44247238-44248295:44249542-44249599:44250021-44250059	206	149	0.9857	0.7583	0.227	0.227	0	0
SE	chr2	Kcnt1	+	25910993-25911356:25912655-25912717:25915779-25915866	211	149	0.89	0.6633	0.227	0.227	7.45431E-09	3.77E-07
SE	chr7	Tmem143	+	45906918-45907112:45908107-45908237:45909322-45909601	279	149	0.8203	0.593	0.227	0.227	0.000683808	0.008668
SE	chr17	Mllt1	-	56899769-56900296:56902563-56902688:56905712-56905855	274	149	0.218	0.4453	-0.227	0.227	2.56462E-14	3.38E-12
SE	chr17	Slc29a1	-	45590257-45590338:45593332-45593432:45595482-45595543	249	149	0.936	0.7093	0.227	0.227	2.37259E-05	0.000486
SE	chr1	Rgs8	+	153665836-153665916:153667825-153667953:153670783-153670884	277	149	0.732	0.5047	0.227	0.227	5.94932E-05	0.001071
SE	chr3	Adgrl2	-	148820451-148820468:148820973-148821054:148821280-148821408	230	149	0.118	0.3453	-0.227	0.227	0.001201412	0.01394
A3SS	chr2	Mga	+	119935149-119935666:119938492-119938786:119938639-119938786	295	149	0.7033	0.477	0.226	0.226	0.000129227	0.00297
A3SS	chr2	Rbm39	-	156176403-156176457:156172828-156174974:156172828-156173022	298	149	0.907	0.6807	0.226	0.226	0.000105051	0.002544
A5SS	chr4	Usp48	+	137616358-137616507:137616358-137620377:137621062-137621259	298	149	0.384	0.61	-0.226	0.226	2.70851E-05	0.000596
MXE	chr3	Gria2	-	80682904-80688714:80689217-80689351:80690404-80690518:80691320-806	263	283	0.4173	0.1913	0.226	0.226	0	0
MXE	chr1	Nfasc	-	132576286-132576417:132582995-132583147:132584364-132584480:132593	265	298	0.034	0.2603	-0.226	0.226	1.45557E-06	6E-05
SE	chr16	Pdxdc1	-	13833161-13834846:13836386-13836633:13837043-13837147	298	149	0.8067	0.5803	0.226	0.226	2.44019E-07	8.62E-06
SE	chr17	Gpank1	+	35121514-35121798:35122229-35122291:35123053-35123659	211	149	0.1003	0.326	-0.226	0.226	8.85348E-07	2.74E-05
SE	chr7	B230209E15Rik	-	61535076-61535488:61604516-61604700:61615160-61615327	298	149	0.688	0.9143	-0.226	0.226	5.38794E-07	1.77E-05
SE	chr15	Scrib	-	76049203-76049301:76049457-76049540:76050050-76050112	232	149	0.449	0.2227	0.226	0.226	3.55487E-09	1.94E-07
SE	chr11	Map2k4	-	65735192-65735366:65743323-65743465:65745552-65745697	291	149	0.1197	0.3453	-0.226	0.226	7E-06	0.000168
SE	chr4	Zcchc7	+	44908451-44908486:44916184-44916312:44925975-44926062	277	149	0.5123	0.286	0.226	0.226	0.004585507	0.042942
A3SS	chr7	Usp29	+	6730560-6731302:6738807-6738945:6738856-6738945	197	149	0.6567	0.4317	0.225	0.225	0.000329923	0.006365
MXE	chr17	Tjap1	-	46263689-46263813:46264883-46264978:46271800-46271929:46282346-462	278	244	0.4077	0.633	-0.225	0.225	0.002642327	0.031995
MXE	chr16	Rbfox1	+	7352932-7353064:7375905-7375969:7391988-7392040:7407964-7408039	213	201	0.6823	0.457	0.225	0.225	0.000122239	0.00267
SE	chr17	Nfya	-	48395650-48395796:48398985-48399068:48400438-48400580	232	149	0.836	0.6113	0.225	0.225	3.24856E-05	0.000639
SE	chr17	Gfer	-	24693189-24694442:24695254-24695417:24695831-24696227	298	149	0.9673	0.7427	0.225	0.225	5.92637E-13	6.34E-11
SE	chr14	Mphosph8	+	56689029-56689115:56690907-56691061:56693424-56693578	298	149	0.9783	0.7537	0.225	0.225	0	0
SE	chr17	Caskin1	+	24495966-24496017:24496099-24496217:24496426-24496571	267	149	0.9487	0.724	0.225	0.225	1.59418E-09	9.16E-08
SE	chr6	Parp11	+	127453315-127453822:127470702-127470830:127474236-127474311	277	149	0.3683	0.593	-0.225	0.225	0.001893451	0.020371

MXE	chr16	Anks3	-	4953819-4953954:4955858-4955939:4960397-4960568:4964120-4964203	298	230	0.5367	0.3123	0.224	0.224	1.01022E-06	4.45E-05
MXE	chr5	Mphosph9	-	124315373-124315896:124316082-124316171:124320845-124320954:124324	258	238	0.6267	0.851	-0.224	0.224	0.000997149	0.014948
MXE	chr8	Rbm34	-	126953366-126953449:126959542-126959585:126961912-126961971:12696	208	192	0.254	0.03	0.224	0.224	2.50555E-12	4.72E-10
RI	chr2	Kcnb1	-	167095969-167099860:167095969-167100815:167100728-167100815	298	149	0.8407	0.617	0.224	0.224	2.6699E-05	0.000274
SE	chr14	Tdrd3	+	87457034-87457118:87458757-87458822:87472076-87472236	214	149	0.981	0.757	0.224	0.224	5.55112E-16	9.03E-14
SE	chrX	Drp2	+	134446628-134446693:134446962-134447027:134447848-134447991	214	149	0.9877	0.7637	0.224	0.224	5.55112E-16	9.03E-14
SE	chr3	Plk4	+	40810662-40810764:40811234-40811383:40811880-40812013	298	149	0.9137	0.6897	0.224	0.224	0.000355334	0.004937
SE	chr11	Rpain	+	70973015-70973075:70973802-70973913:70974069-70974132	260	149	0.914	0.6897	0.224	0.224	2.07969E-07	7.47E-06
SE	chr17	Abcc10	-	46306957-46307300:46310038-46310233:46310377-46310530	298	149	0.7297	0.954	-0.224	0.224	3.67301E-05	0.000708
SE	chr2	Slc4a10	+	62243362-62243453:62244352-62244441:62250379-62250536	238	149	0.5257	0.3013	0.224	0.224	0.000188122	0.00287
SE	chr6	Luc7l2	+	38551334-38551908:38554980-38555063:38570510-38570604	232	149	0.1657	0.39	-0.224	0.224	1.74347E-11	1.44E-09
SE	chr7	Edrf1	+	133643993-133644149:133646990-133647091:133647345-133647466	250	149	0.4123	0.1887	0.224	0.224	0.000194526	0.002954
SE	chr6	Pex26	+	121185710-121185850:121187259-121187566:121190101-121190247	298	149	0.9803	0.756	0.224	0.224	2.95176E-10	1.94E-08
A5SS	chr13	Lgals8	-	12461427-12461738:12461241-12461738:12459174-12459316	298	149	0.1557	0.3783	-0.223	0.223	0.000147854	0.002439
A5SS	chr7	Tial1	-	128448121-128448208:128446731-128448208:128446195-128446270	298	149	0.5357	0.7587	-0.223	0.223	7.33628E-07	2.99E-05
MXE	chr8	Polb	-	22647413-22647471:22648182-22648256:22648305-22648324:22650489-226	168	223	0.0737	0.2963	-0.223	0.223	0	0
SE	chrX	Rbmx	-	57385133-57385221:57386141-57387393:57388061-57388143	298	149	0.6307	0.4073	0.223	0.223	0	0
SE	chr16	Prdm15	-	97816849-97817045:97818212-97818341:97821768-97822010	278	149	1	0.7767	0.223	0.223	1.49421E-08	7E-07
SE	chr4	Klhl17	-	156230505-156230686:156230901-156230974:156231085-156231173	222	149	0.9163	0.6933	0.223	0.223	8.91329E-08	3.47E-06
SE	chr10	Sppl2b	+	80863455-80863551:80864072-80864154:80865121-80865202	231	149	0.7603	0.5377	0.223	0.223	0.000125938	0.00203
SE	chr17	3110052M02Rik	+	21652267-21652345:21656179-21656332:21657519-21657611	298	149	0.6827	0.4593	0.223	0.223	0.001284789	0.014783
MXE	chr5	Lyar	+	38220504-38220635:38222931-38223054:38223134-38223227:38224627-382	272	242	0.192	0.4137	-0.222	0.222	0.003369175	0.038632
RI	chr7	Zfp939	+	39472858-39474439:39472858-39477427:39477311-39477427	298	149	0.9093	0.6877	0.222	0.222	0.000510681	0.003512
RI	chr8	Ikbkb	-	22659205-22660459:22659205-22661492:22661402-22661492	298	149	0.5583	0.3367	0.222	0.222	3.99543E-07	6.77E-06
RI	chr1	Hjurp	-	88263109-88266280:88263109-88266658:88266332-88266658	199	149	0.731	0.509	0.222	0.222	0.002392683	0.012991
RI	chr11	Baiap2	+	120003038-120003085:120003038-120006782:120006359-120006782	298	149	0.9547	0.7323	0.222	0.222	3.87487E-05	0.00038
RI	chr5	Tmem33	+	67286096-67286216:67286096-67291461:67287010-67291461	298	149	0.8383	0.6163	0.222	0.222	2.81131E-12	1.23E-10
SE	chr3	Rrnad1	-	87927055-87927117:87927563-87927715:87927924-87928048	298	149	0.9543	0.732	0.222	0.222	4.06988E-08	1.72E-06
SE	chr4	Fancg	-	43004559-43004605:43004744-43005008:43005211-43005277	298	149	1	0.7777	0.222	0.222	5.16966E-08	2.14E-06
SE	chr6	Cecr5	-	120509494-120510462:120510631-120510819:120514398-120514572	298	149	0.9887	0.767	0.222	0.222	3.68372E-13	4.09E-11
SE	chr1	Dusp12	-	170880130-170880226:170880573-170880691:170880915-170881028	267	149	0.885	0.663	0.222	0.222	2.03418E-07	7.34E-06
SE	chr18	Bin1	+	32429700-32429723:32430750-32430857:32431671-32431742	256	149	0.276	0.0543	0.222	0.222	0	0
SE	chr2	Oprl1	+	181715690-181715946:181717996-181718093:181718377-181718732	246	149	0.1697	0.392	-0.222	0.222	1.7873E-09	1.02E-07
SE	chr8	C230057M02Rik	-	122729771-122729894:122734088-122738893:122752114-122752649	298	149	0.0543	0.276	-0.222	0.222	6.20559E-12	5.55E-10
SE	chr3	Arnt	+	95460377-95460421:95466793-95466837:95470177-95470390	193	149	0.552	0.3303	0.222	0.222	0.000388973	0.005328
SE	chr15	Zfp385a	-	103313895-103315225:103315361-103315456:103315627-103315796	244	149	0.9747	0.7523	0.222	0.222	0	0
SE	chr12	Tmed8	-	87174541-87174667:87176634-87176830:87181389-87181473	298	149	0.9157	0.6933	0.222	0.222	0.00024229	0.003564
SE	chr3	Ppp3ca	+	136928522-136928619:136932011-136932040:136935029-136937725	178	149	0.5227	0.3003	0.222	0.222	0	0
SE	chrX	Atp11c	-	60223283-60225793:60229923-60230013:60236557-60236710	239	149	0.9667	0.7447	0.222	0.222	1.66146E-06	4.75E-05
A3SS	chr2	Celf2	-	6560659-6560790:6553785-6553982:6553785-6553964	166	149	0.353	0.132	0.221	0.221	1.14218E-06	5.61E-05
MXE	chr10	Ank3	+	69980272-69980394:69982116-69982161:69986083-69993762:69994372-699	194	298	0.1383	0.3593	-0.221	0.221	3.69562E-09	3.17E-07
MXE	chr12	Map4k5	-	69816290-69816393:69818022-69818080:69818384-69818498:69822803-698	263	207	0.0963	0.317	-0.221	0.221	1.40421E-07	8.3E-06
MXE	chr11	Hnrnph1	+	50379821-50379964:50382537-50382715:50382800-50382871:50382949-503	298	220	0.5377	0.759	-0.221	0.221	1.30055E-07	7.78E-06
MXE	chr3	Camk2d	+	126767514-126767579:126770785-126770857:126771774-126771846:12678	221	221	0.9617	0.7407	0.221	0.221	4.32987E-15	1.27E-12
RI	chr17	Anks1	+	28059294-28059391:28059294-28062775:28060562-28062775	298	149	0.677	0.4563	0.221	0.221	2.35098E-06	3.34E-05
SE	chr12	Inf2	+	112611410-112612036:112612548-112612604:112614895-112615557	205	149	0.029	0.25	-0.221	0.221	2.14361E-10	1.44E-08
SE	chr2	Grb14	-	64912476-64912853:64914716-64914809:64916829-64916916	242	149	0.99	0.7693	0.221	0.221	7.70495E-14	9.53E-12
SE	chr2	Kcnt1	+	25898696-25898835:25900223-25900286:25900372-25900480	212	149	0.9407	0.7193	0.221	0.221	8.1397E-07	2.55E-05
SE	chr2	Cstf3	+	104660519-104660714:104663294-104663447:104664069-104664163	298	149	0.9883	0.7677	0.221	0.221	2.22045E-16	3.82E-14
SE	chr11	Rnf157	-	116336353-116338830:116340338-116340378:116345907-116345955	189	149	0.9353	0.7147	0.221	0.221	0.000658686	0.008401
SE	chr12	Siva1	+	112644828-112645084:112646838-112647032:112647825-112647981	298	149	0.889	0.668	0.221	0.221	5.71453E-11	4.26E-09
A3SS	chr6	Serbp1	+	67271887-67272027:67272819-67272938:67272864-67272938	193	149	0.9947	0.7743	0.22	0.22	0	0
A3SS	chr4	Pank4	+	154978393-154978442:154978921-154979025:154978955-154979025	182	149	0.5817	0.362	0.22	0.22	1.9619E-06	8.85E-05
A3SS	chr7	Ttyh1	+	4133663-4133738:4133866-4135407:4134351-4135407	298	149	0.7503	0.53	0.22	0.22	7.36311E-12	1.33E-09
A5SS	chr7	Pcf11	-	92659798-92659888:92659405-92659888:92657308-92658866	298	149	0.2863	0.5067	-0.22	0.22	2.07093E-06	7.15E-05
A5SS	chr16	Nxpe3	-	55895288-55896773:55894928-55896773:55890582-55890811	298	149	0.018	0.2377	-0.22	0.22	2.19208E-09	1.87E-07
RI	chr8	Hpf1	+	60905548-60906438:60905548-60907580:60907337-60907580	298	149	0.817	0.597	0.22	0.22	0.000509632	0.003512
RI	chr8	Purg	+	33386330-33387208:33386330-33389495:33389169-33389495	298	149	0.8023	0.5823	0.22	0.22	0.00020017	0.001576
SE	chr1	Ilkap	-	91390607-91390663:91391109-91391197:91398651-91398693	237	149	0.915	0.6953	0.22	0.22	5.35625E-11	4.03E-09
SE	chr4	Rcc1	-	132337731-132337910:132338051-132338238:132339925-132340006	298	149	0.943	0.7233	0.22	0.22	1.43765E-09	8.35E-08
SE	chr7	Zfand6	-	84634239-84634409:84642286-84642437:84652882-84653042	298	149	0.11	0.3297	-0.22	0.22	8.67607E-08	3.38E-06
SE	chr12	Eml5	-	98819861-98819869:98821600-98821614:98824634-98824760	163	149	1	0.7797	0.22	0.22	1.84667E-10	1.25E-08
SE	chr9	Fam214a	+	75018997-75019155:75023585-75023653:75025651-75025847	217	149	1	0.7797	0.22	0.22	1.16751E-12	1.2E-10
SE	chr3	Phtf1	+	103985753-103985909:103988615-103988774:103991038-103991211	298	149	0.924	0.7037	0.22	0.22	0.000157936	0.002463
SE	chr7	Zfp788	+	41632733-41633694:41643333-41643432:41647483-41647617	248	149	0.4773	0.257	0.22	0.22	0.002391956	0.024875
SE	chr9	Zfp266	-	20505977-20506065:20506728-20506882:20508019-20508185	298	149	0.9217	0.7017	0.22	0.22	1.51346E-12	1.54E-10
SE	chr17	3110052M02Rik	+	21651803-21652345:21656206-21656332:21657519-21657611	275	149	0.6757	0.456	0.22	0.22	0.001994005	0.021314
SE	chr11	Sh3pxd2b	+	32407562-32407666:32408968-32409051:32411457-32411574	232	149	0.597	0.3773	0.22	0.22	7.76266E-05	0.001344
A5SS	chr11	Med1	-	98158329-98158469:98155177-98158469:98152154-98154906	298	149	0.9823	0.7633	0.219	0.219	3.47565E-08	2.17E-06
A5SS	chr10	Chpt1	-	88475426-88475536:88475333-88475536:88472813-88472984	241	149	0.7367	0.5177	0.219	0.219	0.006297134	0.048113
MXE	chr12	Npas3	+	53354345-53354434:53501034-53501278:53640454-53640536:53831696-538	298	231	0.4803	0.6993	-0.219	0.219	0.003844013	0.042809
MXE	chr12	Rian	+	109640267-109640328:109642565-109642652:109643414-109643461:109645	236	196	0.6047	0.3853	0.219	0.219	1.56735E-08	1.17E-06
MXE	chr3	Gria2	-	80682904-80688714:80690404-80690518:80691320-80691434:80692285-806	263	263	0.4837	0.7023	-0.219	0.219	5.44009E-15	1.54E-12
MXE	chr11	Trim37	+	87129716-87129817:87137590-87137630:87140485-87140601:87145464-871	189	265	0.4577	0.239	0.219	0.219	2.38906E-05	0.000677
SE	chr8	Rltpr	+	105697217-105697438:105697561-105697641:105697868-105697945	229	149	0.2867	0.5057	-0.219	0.219	5.90036E-08	2.4E-06
SE	chr4	Morn1	+	155092269-155092359:155093062-155093149:155101266-155101362	236	149	0.63	0.849	-0.219	0.219	0.001627554	0.017942
SE	chr2	Scn3a	-	65524604-65524876:65525695-65525786:65525877-65525968	240	149	0.3787	0.16	0.219	0.219	7.25029E-06	0.000173
SE	chr1	Raph1	-	60503518-60503595:60510417-60510491:60525592-60526100	223	149	0.5867	0.8057	-0.219	0.219	0.000741685	0.009289
SE	chr8	Ttc13	-	124690277-124690359:124699656-124699721:124709903-124709973	214	149	0.0493	0.2683	-0.219	0.219	2.01059E-08	9.09E-07
SE	chr8	Polb	-	22648182-22648256:22648803-22648832:22650489-22650555	178	149	0.1193	0.338	-0.219	0.219	4.99034E-12	4.52E-10
SE	chr12	Atp5s	+	69724927-69725014:69737951-69738061:69740899-69741107	259	149	0.659	0.8777	-0.219	0.219	0.000878105	0.010708
SE	chr11	Ccdc85a	-	28385688-28388948:28392854-28392952:28396046-28396165	247	149	0.431	0.212	0.219	0.219	0.000321421	0.004529
SE	chr9	Zfp266	-	20505977-20506065:20506728-20506882:20521020-20521119	298	149	0.8493	0.6307	0.219	0.219	0.000158649	0.002471
SE	chr8	Klhl26	-	70455466-70455648:70475807-70475936:70476850-70476955	278	149	0.155	0.3737	-0.219	0.219	0.000566844	0.007394
SE	chr8	Sipa1l2	-	125422579-125422702:125423193-125423246:125433178-125433309	202	149	0.497	0.2777	0.219	0.219	0.000701817	0.008856
SE	chr19	Coro1b	+	4152522-4152579:4153227-4153490:4153597-4154035	298	149	0.96	0.7413	0.219	0.219	0	0
A3SS	chr2	Frmd5	-	121548889-121549232:121545529-121548709:121545529-121548023	298	149	0.839	0.621	0.218	0.218	3.06386E-05	0.000929
MXE	chr16	Grik1	-	87895902-87896351:87914154-87914404:87923077-87923302:87935898-879	298	298	0.5737	0.3553	0.218	0.218	0.001035036	0.015395
MXE	chr7	Ppp2r2d	+	138846612-138846704:138848459-138848665:138865021-138865117:13886	298	245	0.456	0.6737	-0.218	0.218	5.89663E-06	0.000206
SE	chr2	Lypd6b	+	49863811-49863906:49903376-49903452:49930925-49930984	225	149	0.3123	0.5307	-0.218	0.218	0.000138063	0.002194
SE	chr7	Lins1	+	66708639-66708728:66709294-66709495:66710230-66710746	298	149	1	0.7817	0.218	0.218	2.8721E-06	7.64E-05
SE	chr9	Trank1	+	111383074-111383162:111385119-111385204:111386371-111386474	234	149	0.4283	0.2103	0.218	0.218	4.00848E-07	1.34E-05
SE	chr1	Dusp12	-	170880130-170880226:170880915-170881028:170885067-170885540	262	149	0.9073	0.6897	0.218	0.218	8.21524E-06	0.000193
SE	chr8	Ttc13	-	124690277-124690359:124695636-124695752:124709903-124709973	265	149	0.332	0.5503	-0.218	0.218	1.33692E-05	0.000295
SE	chr6	Adcyap1r1	+	55484942-55485033:55491227-55491310:55495574-55495615	232	149	1	0.7823	0.218	0.218	1.02532E-08	5.01E-07
SE	chr10	Plpp2	-	79530498-79530772:79530877-79531028:79533665-79533787	298	149	0.6917	0.91	-0.218	0.218	0.003106539	0.031013
A5SS	chr5	Trafd1	-	121373339-121373474:121373150-121373474:121372603-121372675	298	149	0.884	0.667	0.217	0.217	0	0
MXE	chr3	Gria2	-	80682904-80688714:80689217-80689351:80690404-80690518:80692285-806	263	283	0.695	0.4783	0.217	0.217	7.69557E-11	1.03E-08
MXE	chr9	Map4	+	110072613-110072705:110079781-110079893:110081421-110081610:11008	261	298	0.6403	0.4237	0.217	0.217	9.53892E-10	9.57E-08
MXE	chr16	Itsn1	+	91841489-91841610:91848722-91848969:91853695-91853900:91854573-918	298	298	0.3363	0.5533	-0.217	0.217	2.37377E-12	4.59E-10
RI	chr10	Ankrd24	+	81639307-81639344:81639307-81639936:81639856-81639936	298	149	0.411	0.1937	0.217	0.217	3.00124E-06	4.1E-05
RI	chr15	Phf20l1	+	66623761-66623883:66623761-66624099:66623992-66624099	256	149	0.673	0.4563	0.217	0.217	7.72052E-05	0.000688
SE	chr11	Ccdc88a	+	29493879-29494093:29497343-29497426:29499335-29499406	232	149	0.9257	0.709	0.217	0.217	0.000378666	0.005207
SE	chr9	Zfp558	-	18459657-18459752:18467920-18468046:18468614-18468702	275	149	0.9537	0.737	0.217	0.217	8.28887E-09	4.15E-07
SE	chr9	Pcsk7	+	45926734-45926828:45927037-45927118:45927575-45927668	230	149	0.365	0.1477	0.217	0.217	0.000548207	0.007191
SE	chrX	Dlg3	+	100809862-100809907:100811502-100811543:100812726-100812776	190	149	0.577	0.7937	-0.217	0.217	3.18948E-09	1.76E-07
SE	chr4	Ptpru	-	131780830-131780866:131785743-131785760:131787308-131787384	166	149	0.423	0.6397	-0.217	0.217	0.000168631	0.00261
SE	chr11	Ncor1	-	62345235-62345318:62349353-62349493:62351378-62351546	289	149	0.57	0.787	-0.217	0.217	4.22874E-09	2.29E-07
SE	chr1	Clk1	-	58419667-58419733:58420121-58420211:58421083-58421308	239	149	0.5333	0.3167	0.217	0.217	0.00022572	0.00335
SE	chr4	Rars2	+	34630473-34630570:34633658-34633713:34636952-34637035	204	149	0.9097	0.6927	0.217	0.217	1.6E-06	4.6E-05
SE	chr8	Dcun1d2	-	13258947-13259043:13261385-13261467:13271590-13271720	231	149	0.9257	0.709	0.217	0.217	1.93306E-06	5.46E-05
SE	chr1	Rnf152	-	105276917-105284954:105355041-105355163:105356343-105356710	271	149	0.2693	0.4867	-0.217	0.217	0.000607795	0.007855
SE	chr6	Bms1	-	118413731-118413919:118416463-118416542:118418409-118418617	228	149	0.987	0.7697	0.217	0.217	4.59373E-09	2.46E-07
SE	chr7	Stim1	+	102430885-102430951:102433197-102433233:102435384-102437319	185	149	0.6043	0.3873	0.217	0.217	0.004295056	0.040648
SE	chrX	Atp11c	-	60229925-60230013:60236372-60236437:60236557-60236710	214	149	0.3353	0.118	0.217	0.217	4.67018E-05	0.000869
RI	chr15	Csad	-	102178527-102178616:102178527-102178832:102178781-102178832	298	149	0.571	0.3553	0.216	0.216	0.0015286	0.009083
SE	chr19	Vldlr	+	27234824-27234912:27236171-27236362:27238053-27238424	298	149	0.503	0.719	-0.216	0.216	9.10385E-05	0.001542
SE	chr9	Fbxl12	-	20637749-20639198:20642024-20642096:20644195-20644298	221	149	0.9687	0.7523	0.216	0.216	3.13976E-08	1.37E-06
SE	chr6	Magohb	-	131285643-131285725:131288036-131288146:131289389-131289447	259	149	0.993	0.7773	0.216	0.216	0	0
SE	chr13	Col4a3bp	+	96616457-96616549:96616994-96617071:96624695-96624790	226	149	0.7463	0.53	0.216	0.216	7.8539E-07	2.47E-05
SE	chr11	Mlx	+	101087754-101087790:101088236-101088325:101088598-101088704	238	149	0.9003	0.684	0.216	0.216	2.22045E-16	3.82E-14
SE	chrX	Taz	+	74288448-74288513:74288662-74289019:74289105-74289182	298	149	0.8663	0.6503	0.216	0.216	0.000892955	0.010869
SE	chr19	Wbp1l	+	46599106-46599293:46606712-46606759:46644353-46644455	196	149	0.562	0.3457	0.216	0.216	1.45009E-06	4.22E-05
SE	chr4	Hook1	+	96014808-96014901:96016409-96016443:96016943-96017025	183	149	0.9803	0.7643	0.216	0.216	8.97236E-09	4.46E-07

SE	chr2	Elmo2	-	165301941-165301991:165303666-165303701:165304900-165304978	184	149	0.4623	0.2463	0.216	0.216	4.58051E-09	2.46E-07
SE	chr17	Fez2	-	78384744-78384819:78386991-78387071:78400630-78400898	229	149	0.6767	0.461	0.216	0.216	3.29041E-07	1.13E-05
SE	chr9	Mthfsl	-	88688605-88688985:88694260-88694332:88715488-88715749	221	149	0.0823	0.298	-0.216	0.216	2.13128E-06	5.92E-05
A5SS	chr14	Dcaf11	+	55560441-55560627:55560441-55560818:55561210-55561442	298	149	0.6593	0.444	0.215	0.215	0.000474155	0.006271
MXE	chr17	Nrxn1	-	90162114-90162433:90163792-90163881:90208323-90208494:90362760-903	298	238	0.8773	0.662	0.215	0.215	0	0
MXE	chr1	Stau2	-	16463035-16463194:16486020-16486150:16491241-16491394:16509342-165	298	279	0.1537	0.369	-0.215	0.215	1.54992E-08	1.16E-06
RI	chr18	Brd8	-	34599826-34600241:34599826-34602247:34602203-34602247	298	149	0.4497	0.2347	0.215	0.215	6.9178E-13	3.46E-11
SE	chr18	Apc	+	34261068-34261157:34266058-34266222:34268298-34268379	298	149	0.7757	0.5603	0.215	0.215	6.91955E-08	2.77E-06
SE	chr2	Mtg2	+	180083345-180083460:180084451-180084589:180085380-180085902	287	149	0.9887	0.7737	0.215	0.215	5.22466E-08	2.15E-06
SE	chr2	Lrrc4c	+	97464684-97468420:97522122-97522199:97603489-97603540	226	149	0.0547	0.2697	-0.215	0.215	1.81645E-11	1.49E-09
SE	chr7	Dmwd	+	19078055-19078237:19081919-19081993:19082276-19082775	223	149	0.9683	0.753	0.215	0.215	7.28117E-09	3.69E-07
SE	chr4	Tmem57	-	134833198-134833321:134836767-134837034:134837849-134837990	298	149	0.3393	0.1247	0.215	0.215	0.002311147	0.024168
A3SS	chr17	Phf10	-	14946655-14946763:14945001-14946387:14945001-14945184	298	149	0.9967	0.7827	0.214	0.214	0	0
MXE	chrX	Dlg3	+	100807180-100807255:100809862-100809907:100811502-100811543:100812	194	190	0.7797	0.5657	0.214	0.214	2.83873E-07	1.56E-05
SE	chr2	Tpd52l2	+	181508166-181508267:181510504-181510530:181511574-181511615	175	149	0.5563	0.3423	0.214	0.214	1.00212E-08	4.9E-07
SE	chr14	Ogdhl	+	32322012-32322116:32323879-32323947:32325794-32325998	217	149	0.5893	0.375	0.214	0.214	0.002992958	0.030065
SE	chr12	Fam49a	+	12262133-12262238:12276343-12276415:12295616-12295688	221	149	0.3083	0.5227	-0.214	0.214	0.000404929	0.005519
SE	chr12	Dtnb	+	3772587-3772746:3773551-3773640:3774570-3774610	238	149	0.238	0.452	-0.214	0.214	0.001278107	0.014716
SE	chr7	Tenm4	+	96852333-96852582:96854717-96854737:96863450-96863682	169	149	0.9543	0.74	0.214	0.214	4.83052E-07	1.59E-05
A5SS	chr17	Wdr4	-	31509821-31509912:31509769-31509912:31503510-31503666	200	149	0.8517	0.6383	0.213	0.213	6.29245E-07	2.62E-05
RI	chr8	Cers4	+	4520216-4520308:4520216-4520682:4520554-4520682	298	149	0.5277	0.315	0.213	0.213	3.21951E-06	4.38E-05
SE	chr7	Chrna7	-	63103780-63103889:63104978-63105064:63106004-63106198	235	149	1	0.7867	0.213	0.213	5.47086E-10	3.45E-08
SE	chr4	Ptprd	-	76099412-76099605:76100447-76100752:76102955-76103099	298	149	0.7247	0.5113	0.213	0.213	0.003267041	0.032366
SE	chr2	Cacna1b	-	24661754-24661880:24670073-24670156:24677822-24678039	232	149	0.0903	0.3037	-0.213	0.213	1.5343E-09	8.85E-08
SE	chr5	Git2	-	114752279-114752327:114753148-114753153:114753279-114753324	154	149	0.792	0.579	0.213	0.213	0.003098979	0.030956
SE	chr5	Ept1	+	30247463-30247531:30248412-30248520:30256078-30256340	257	149	1	0.787	0.213	0.213	1.84704E-11	1.52E-09
SE	chr10	Anks1b	+	90895099-90895206:90897247-90897426:90914696-90914767	298	149	0.974	0.7613	0.213	0.213	7.90568E-12	6.91E-10
SE	chr11	Slc43a2	+	75563562-75563708:75566404-75566415:75566979-75567117	160	149	0.7247	0.5113	0.213	0.213	0.004266595	0.040443
SE	chr1	Dcaf8	+	172186296-172186368:172186855-172186912:172187353-172187460	206	149	0.9847	0.7717	0.213	0.213	0	0
SE	chr11	Rps6kb1	-	86535411-86535460:86543931-86544045:86544566-86544765	263	149	0.3843	0.5977	-0.213	0.213	1.20035E-07	4.56E-06
A3SS	chr4	Mier1	+	103115560-103115660:103118239-103118315:103118242-103118315	151	149	0.6997	0.4873	0.212	0.212	0.002149861	0.02698
MXE	chr8	Rbm34	-	126959542-126959585:126965382-126965622:126969983-126970128:12697(	294	298	0.8297	0.6177	0.212	0.212	7.45969E-09	5.89E-07
MXE	chr7	Dlg2	+	92386915-92386990:92417224-92417323:92418088-92418133:92427691-924	248	194	0.4553	0.2433	0.212	0.212	1.15476E-06	4.96E-05
SE	chr15	Fam91a1	+	58441578-58441726:58442832-58442966:58443161-58443274	283	149	0.9803	0.768	0.212	0.212	0	0
SE	chr2	Ppig	+	69722819-69722957:69731746-69731813:69732197-69732274	216	149	0.9357	0.7233	0.212	0.212	1.38209E-11	1.16E-09
SE	chr7	Emsy	-	98587130-98590884:98593331-98593810:98594752-98595312	298	149	0.8133	0.6013	0.212	0.212	2.72166E-06	7.3E-05
SE	chr1	Cacna1e	-	154486498-154486707:154488044-154488133:154488785-154488838	238	149	0.5303	0.3187	0.212	0.212	0.001163734	0.01357
SE	chr2	4930402H24Rik	-	130777205-130777281:130778417-130778504:130784424-130784460	236	149	0.977	0.7653	0.212	0.212	1.58544E-11	1.32E-09
SE	chr11	Fdxr	-	115269697-115269896:115270015-115270099:115270323-115270430	233	149	0.5557	0.3437	0.212	0.212	0.00013035	0.002087
A3SS	chr19	Smc5	-	23218405-23218530:23215155-23215225:23215155-23215199	174	149	0.3397	0.1283	0.211	0.211	0.003634213	0.03995
A3SS	chr11	Pus10	+	23706885-23706946:23707361-23707568:23707523-23707568	298	149	0.2697	0.0587	0.211	0.211	9.80001E-08	7.44E-06
A5SS	chr10	Ankrd24	+	81635688-81635752:81635688-81635857:81636369-81636426	253	149	0.3343	0.1233	0.211	0.211	1.08327E-05	0.000277
MXE	chrX	Pdzd4	-	73796376-73796477:73796830-73796892:73798194-73798292:73799834-738	247	211	0.0407	0.252	-0.211	0.211	0	0
RI	chr13	2010111I01Rik	+	63298712-63298856:63298712-63302877:63301779-63302877	298	149	0.796	0.5853	0.211	0.211	0.0002978	0.002205
SE	chr4	Cpne3	-	19536527-19536625:19539152-19539180:19540741-19540830	177	149	0.2843	0.073	0.211	0.211	2.28062E-05	0.00047
SE	chr18	Myo5b	+	74713548-74713632:74714826-74714959:74715959-74716078	282	149	0.982	0.771	0.211	0.211	1.14848E-07	4.39E-06
SE	chr11	Kat7	-	95271853-95275887:95276443-95276549:95277469-95277615	255	149	0.8283	0.617	0.211	0.211	1.57294E-07	5.81E-06
SE	chr4	Camta1	-	151586415-151586482:151792419-151792502:151830418-151830536	232	149	0.6523	0.863	-0.211	0.211	3.02371E-06	7.97E-05
SE	chr9	Cacna2d2	+	107518193-107518269:107519179-107519199:107522123-107522183	169	149	0.3883	0.1777	0.211	0.211	9.64746E-05	0.001618
SE	chr7	Ankrd27	+	35591752-35591882:35600315-35600425:35602428-35602582	259	149	0.8747	0.6633	0.211	0.211	0.003543116	0.034655
SE	chr11	Slc38a10	-	120140593-120140686:120141600-120141645:120150980-120151063	194	149	0.8617	0.6503	0.211	0.211	5.84892E-07	1.9E-05
SE	chr7	Zfp787	-	6131489-6133171:6134642-6134708:6143062-6143151	215	149	0.0753	0.2867	-0.211	0.211	6.3558E-11	4.7E-09
A3SS	chr3	Gria2	-	80692285-80692532:80691320-80691743:80691320-80691434	298	149	0.4563	0.2463	0.21	0.21	0	0
MXE	chr14	Appl1	-	26958453-26958511:26959425-26959466:26960951-26961038:26962806-269	236	190	0.7063	0.496	0.21	0.21	2.56155E-05	0.00072
MXE	chr4	St3gal3	-	118031840-118031882:118054650-118054697:118107549-118107693:11813	293	196	0.916	0.7057	0.21	0.21	6.18901E-07	3.05E-05
RI	chr15	Kifc2	+	76662595-76662896:76662595-76663083:76662974-76663083	225	149	0.073	0.2827	-0.21	0.21	5.9952E-15	3.66E-13
SE	chr6	Tulp3	-	128324985-128325078:128325424-128325520:128325877-128325991	245	149	0.3237	0.534	-0.21	0.21	0.000455061	0.006117
SE	chr5	Srrm3	+	135857038-135857215:135859282-135859299:135867127-135867146	166	149	0	0.21	-0.21	0.21	1.26656E-07	4.79E-06
SE	chr1	Cspp1	+	10083501-10083558:10085747-10085952:10088051-10088184	298	149	0.284	0.4937	-0.21	0.21	0.000467399	0.00625
SE	chr14	Pinx1	+	63878112-63878188:63892723-63892833:63919097-63919855	259	149	0.066	0.2757	-0.21	0.21	2.56171E-07	9E-06
A3SS	chr14	Bmp1	-	70490020-70490200:70486147-70486930:70486147-70486272	298	149	0.195	0.404	-0.209	0.209	5.93466E-08	4.87E-06
A5SS	chr19	Cbwd1	-	24961384-24961616:24961317-24961616:24957904-24957990	215	149	0.634	0.425	0.209	0.209	0.000368011	0.005119
SE	chr7	Shank1	+	44343658-44343738:44344298-44344321:44344506-44344574	172	149	0.815	0.6057	0.209	0.209	6.29399E-11	4.66E-09
SE	chr2	Fmnl2	+	53126853-53126976:53128717-53128842:53130412-53130497	274	149	0.9223	0.713	0.209	0.209	2.28264E-07	8.13E-06
SE	chr1	Pam	-	97821091-97821988:97822936-97822989:97825837-97826037	202	149	0.9897	0.781	0.209	0.209	0	0
SE	chr2	Metap1d	+	71512126-71512168:71515637-71515800:71523802-71523849	298	149	0.991	0.7823	0.209	0.209	1.62205E-11	1.34E-09
SE	chr12	Rnaseh1	+	28655585-28655684:28657272-28657326:28657633-28657717	203	149	0.987	0.7783	0.209	0.209	3.42193E-08	1.48E-06
SE	chr2	Ubr1	-	120893068-120893216:120895072-120895162:120896496-120896593	239	149	0.9327	0.7237	0.209	0.209	0.000749326	0.009369
SE	chr8	Unc13a	-	71636538-71636567:71637772-71637927:71639217-71639222	298	149	1	0.7913	0.209	0.209	2.34638E-07	8.32E-06
SE	chr5	Ept1	+	30247463-30247531:30252710-30252784:30256078-30256340	223	149	1	0.7907	0.209	0.209	2.96994E-11	2.35E-09
SE	chrX	Atrx	-	105899881-105899993:105911470-105911654:105928959-105929406	298	149	0.2897	0.499	-0.209	0.209	3.69964E-07	1.25E-05
SE	chr10	Herc4	+	63314487-63314569:63315660-63315843:63316700-63316802	298	149	0.808	0.599	0.209	0.209	1.52243E-05	0.000329
A3SS	chr5	Chfr	+	110151527-110151686:110152336-110153280:110153118-110153280	298	149	0.9843	0.7763	0.208	0.208	0	0
MXE	chr6	Adcyap1r1	+	55484942-55485033:55487659-55487742:55491230-55491310:55494077-554	232	229	0.0807	0.289	-0.208	0.208	9.1394E-08	5.71E-06
MXE	chr8	Lzts1	-	69132669-69136159:69138352-69139149:69140609-69141086:69183974-691	298	298	0.3887	0.181	0.208	0.208	0.00087192	0.013482
SE	chr13	Trip13	-	73919995-73920101:73920348-73920434:73921173-73921236	235	149	0.9623	0.7547	0.208	0.208	1.27839E-06	3.79E-05
SE	chr3	Clk2	+	89168695-89168923:89169618-89169702:89170049-89170115	233	149	0.799	0.5913	0.208	0.208	9.8104E-05	0.00164
SE	chr7	Zfp180	+	24097821-24097895:24101488-24101568:24104405-24107709	229	149	0.8407	0.633	0.208	0.208	0.000595512	0.007712
SE	chr19	Zfyve27	+	42184118-42184189:42185620-42185640:42185805-42185949	169	149	0.87	0.662	0.208	0.208	1.23279E-09	7.25E-08
SE	chr8	Letm2	-	25581673-25581786:25585349-25585468:25586607-25586807	268	149	1	0.7923	0.208	0.208	1.19934E-07	4.56E-06
SE	chr3	Ovgp1	+	105975971-105976027:105977664-105977829:105978274-105978398	298	149	0.989	0.7807	0.208	0.208	2.63904E-06	7.11E-05
SE	chr7	Emsy	-	98590594-98590884:98593331-98593985:98594752-98595312	298	149	0.7903	0.5823	0.208	0.208	2.24931E-05	0.000464
SE	chr7	Pdcd5	-	35642615-35642686:35643624-35643715:35644351-35644412	240	149	0.9913	0.7837	0.208	0.208	0	0
SE	chr8	Tmem208	+	105328322-105328458:105328590-105328692:105328772-105329057	251	149	0.868	0.6603	0.208	0.208	1.97087E-08	8.91E-07
SE	chr16	Tra2b	-	22254984-22255117:22258986-22259261:22265741-22265959	298	149	0.291	0.4987	-0.208	0.208	2.22045E-16	3.82E-14
SE	chr17	Unkl	+	25228583-25228666:25229239-25229466:25230207-25230409	298	149	0.7853	0.577	0.208	0.208	0.000637296	0.00818
A3SS	chr11	Lrrc45	+	120718442-120718553:120718650-120719132:120719040-120719132	298	149	0.796	0.5887	0.207	0.207	8.62359E-06	0.000326
A3SS	chr16	Brwd1	-	96002434-96003354:95992089-95994279:95992089-95993977	298	149	0.859	0.6523	0.207	0.207	0.003433305	0.038351
MXE	chr3	Mynn	+	30611441-30611619:30612469-30612552:30613552-30613638:30616551-306	232	235	0.4233	0.2167	0.207	0.207	5.5392E-06	0.000194
MXE	chrX	Slc25a14	+	48623578-48623828:48623962-48624206:48625596-48625640:48628136-486	298	193	0.751	0.5443	0.207	0.207	0.000121835	0.002665
SE	chr10	Ankrd24	+	81640819-81640990:81641976-81642048:81642301-81642419	221	149	0.6607	0.454	0.207	0.207	0.003927549	0.037848
SE	chr3	Adam15	-	89340401-89340472:89340713-89340776:89340863-89340937	212	149	0.373	0.1657	0.207	0.207	1.70369E-05	0.000363
SE	chr15	Naga	-	82335843-82336020:82336770-82336941:82337387-82337522	298	149	0.712	0.5053	0.207	0.207	0.000199345	0.003012
SE	chr15	Csad	-	102176997-102177786:102178527-102178832:102178961-102179160	298	149	0.783	0.576	0.207	0.207	0.001570403	0.017432
SE	chr17	Cacna1h	-	25380225-25380295:25380520-25380653:25380804-25380913	282	149	0.9677	0.7603	0.207	0.207	0	0
SE	chr16	Tnk2	+	32679459-32680807:32680900-32680995:32681152-32681279	244	149	0.4607	0.2537	0.207	0.207	4.76749E-09	2.54E-07
SE	chrX	Atp11c	-	60229165-60229252:60236372-60236437:60236557-60236710	214	149	0.3053	0.0987	0.207	0.207	0.002590772	0.026691
A3SS	chr4	Ubxn11	+	134126207-134126280:134126366-134126506:134126421-134126506	203	149	0.7343	0.9403	-0.206	0.206	9.14034E-05	0.002243
MXE	chrX	Dlg3	+	100807180-100807255:100809862-100809907:100812726-100812776:10081	194	199	0.5677	0.362	0.206	0.206	1.02902E-05	0.000325
MXE	chr10	Ank3	+	69980272-69980394:69982116-69982197:69986083-69993762:69994372-699	230	298	0.2627	0.4683	-0.206	0.206	1.80318E-07	1.05E-05
MXE	chr1	Nfasc	-	132579378-132579508:132582995-132583147:132588074-132588310:13259	298	298	0.9207	0.715	0.206	0.206	3.84965E-11	5.43E-09
MXE	chr7	Fam71e1	+	44496600-44496730:44496915-44497064:44497503-44497633:44500150-445	298	279	0.7397	0.5337	0.206	0.206	0.000348742	0.006437
MXE	chr12	Kcnk10	-	98436192-98436334:98440592-98440778:98489905-98490065:98496193-984	298	298	0.3993	0.6057	-0.206	0.206	0.001381335	0.019286
MXE	chr10	Mdm1	+	118146595-118146971:118147981-118148115:118150789-118150956:11815	283	298	0.545	0.3393	0.206	0.206	0.002810261	0.03363
RI	chr13	Ggps1	-	14049793-14052130:14049793-14052393:14052278-14052393	295	149	0.9613	0.7553	0.206	0.206	1.29208E-11	5.05E-10
SE	chr14	Bin3	+	70118604-70118652:70123841-70124042:70124183-70124244	298	149	0.8927	0.687	0.206	0.206	0.001499789	0.01678
SE	chr16	Chrd	+	20739407-20739510:20739954-20740056:20741190-20741344	251	149	0.9037	0.6977	0.206	0.206	0.000264452	0.003838
SE	chr14	Phf7	-	31239631-31239737:31240274-31240433:31240859-31240983	298	149	0.7067	0.5007	0.206	0.206	0.003094621	0.030917
SE	chr9	Sltm	+	70581301-70581464:70583932-70584112:70584753-70584877	298	149	0.852	0.6457	0.206	0.206	4.27769E-13	4.67E-11
SE	chr4	Tnc	-	63982651-63982923:63984460-63984732:63993027-63993299	298	149	0.9397	0.734	0.206	0.206	2.53442E-12	2.43E-10
SE	chr6	Tia1	+	86418878-86418932:86419101-86419812:86420324-86420411	298	149	0.7423	0.5363	0.206	0.206	3.82319E-06	9.81E-05
SE	chr11	Ctns	-	73188430-73188561:73188689-73188792:73191678-73191762	252	149	0.8273	0.6217	0.206	0.206	0.000383906	0.005269
SE	chr18	Nme5	-	34567036-34567154:34571456-34571661:34578558-34578767	298	149	0.4543	0.2487	0.206	0.206	0.001188994	0.013815
SE	chr1	Gls	-	52199855-52199887:52204346-52204516:52207301-52207359	298	149	0.243	0.037	0.206	0.206	0	0
SE	chr19	Nrxn2	+	6454979-6455280:6463824-6463847:6472258-6472419	172	149	0.53	0.3237	0.206	0.206	6.25942E-10	3.9E-08
SE	chr6	C2cd5	-	143067056-143067141:143072543-143072694:143073663-143073787	298	149	0.6947	0.9007	-0.206	0.206	0.004023065	0.038616
SE	chr17	AI413582	-	27564149-27564254:27564385-27564410:27564562-27564750	174	149	0.989	0.783	0.206	0.206	0	0
SE	chr12	Smek1	-	101044490-101044659:101045526-101045681:101049660-101049818	298	149	0.2263	0.4327	-0.206	0.206	3.42751E-07	1.17E-05
SE	chr5	Pan3	+	147526519-147526680:147526925-147527041:147527129-147527230	265	149	0.9393	0.7333	0.206	0.206	8.32673E-05	0.001428
A3SS	chr7	Tmem91	-	25670486-25670635:25669139-25669498:25669139-25669350	296	149	0.6597	0.4547	0.205	0.205	0.004038316	0.043299
A5SS	chr14	Tmem55b	-	50930634-50930849:50930613-50930849:50930134-50930324	169	149	0.7337	0.529	0.205	0.205	1.43283E-09	1.34E-07
MXE	chr11	Map2k4	-	65735192-65735366:65743323-65743465:65775733-65775765:65788136-657	181	291	0.8483	0.6433	0.205	0.205	0.000977259	0.014766
MXE	chr5	Preb	-	30951671-30955737:30955930-30956089:30957888-30958061:30958471-309	298	298	0.0443	0.2497	-0.205	0.205	5.65215E-13	1.23E-10
MXE	chr13	Gfm2	+	97150325-97150413:97151635-97151724:97153228-97153316:97155190-971	238	237	0.116	0.321	-0.205	0.205	0.000980451	0.0148

SE	chr6	Casd1	+	4631519-4631598:4634002-4634103:4635746-4635886	250	149	0.9587	0.7537	0.205	0.205	4.88665E-12	4.43E-10
SE	chr1	Kif1a	-	93025621-93025735:93026832-93026855:93034546-93034572	172	149	0.5413	0.3367	0.205	0.205	0.00187376	0.020192
SE	chr11	Hdac5	-	102218416-102218487:102221768-102221851:102224734-102224955	232	149	0.0053	0.21	-0.205	0.205	0	0
SE	chr6	Parp11	+	127453315-127453822:127471548-127471668:127474236-127474311	269	149	0.6307	0.836	-0.205	0.205	2.09729E-05	0.000436
SE	chr9	Gria4	-	4424207-4424454:4427030-4427144:4432773-4432887	263	149	0.5363	0.3313	0.205	0.205	0.00023104	0.003422
SE	chr9	Ireb2	+	54882319-54882456:54883897-54884115:54885875-54885944	298	149	0.9823	0.7777	0.205	0.205	0	0
A3SS	chr9	Msantd2	+	37510131-37513703:37517217-37517472:37517373-37517472	298	149	0.9527	0.7483	0.204	0.204	6.4567E-08	5.24E-06
A5SS	chr10	Ank3	+	69986083-69992475:69986083-69993762:69994372-69994446	298	149	0.909	0.7053	0.204	0.204	2.72116E-13	5.3E-11
RI	chrX	Wdr45	+	7724187-7724291:7724187-7726292:7726198-7726292	298	149	1	0.7963	0.204	0.204	5.61986E-06	7.1E-05
SE	chr7	Phrf1	+	141258032-141260707:141260915-141261114:141261213-141261542	298	149	0.9663	0.762	0.204	0.204	4.85473E-09	2.57E-07
SE	chr6	Mical3	-	120962755-120962865:120969355-120969492:120973403-120973647	286	149	0.398	0.6017	-0.204	0.204	8.57866E-05	0.001466
SE	chr7	Zfand6	-	84634239-84634409:84642286-84642437:84679310-84679408	298	149	0.0467	0.251	-0.204	0.204	0	0
SE	chr6	Adcyap1r1	+	55484942-55485033:55491227-55491310:55494077-55494206	232	149	0.4507	0.2467	0.204	0.204	4.53049E-12	4.13E-10
SE	chr19	Ppp6r3	-	3459361-3459480:3459747-3459764:3464545-3464695	166	149	0.548	0.344	0.204	0.204	2.98638E-07	1.03E-05
SE	chr3	Papss1	+	131564768-131564983:131567502-131567678:131579311-131579425	298	149	0.1207	0.3243	-0.204	0.204	2.66343E-12	2.54E-10
SE	chr11	Slc38a10	-	120141600-120141645:120147721-120147838:120150980-120151063	266	149	0.3953	0.599	-0.204	0.204	1.53624E-06	4.44E-05
SE	chr15	Hsf1	+	76495970-76496078:76497619-76497694:76497768-76497829	224	149	0.9707	0.767	0.204	0.204	1.34309E-08	6.38E-07
SE	chr5	Cdk8	+	146286108-146286239:146292639-146292767:146295163-146295232	277	149	0.913	0.709	0.204	0.204	1.35442E-06	3.99E-05
A3SS	chr4	Pusl1	-	155890499-155890669:155889434-155889723:155889434-155889596	275	149	0.693	0.896	-0.203	0.203	2.42226E-05	0.00078
MXE	chr2	Metap1d	+	71512126-71512168:71515637-71515800:71523802-71523849:71524717-715	298	196	0.6867	0.4833	0.203	0.203	4.72825E-05	0.001205
MXE	chr15	Mapk15	+	75993731-75993928:75994845-75994943:75995072-75995101:75995234-759	247	178	0.7843	0.9877	-0.203	0.203	4.68032E-09	3.9E-07
RI	chr19	Sfxn2	+	46594672-46594868:46594672-46596901:46596668-46596901	298	149	0.7697	0.973	-0.203	0.203	1.98915E-07	3.63E-06
RI	chr11	Gas2l1	-	5063826-5064478:5063826-5065094:5064837-5065094	298	149	0.7903	0.5877	0.203	0.203	0.000700503	0.004651
SE	chr2	Stx16	+	174091482-174091622:174092413-174092567:174093434-174093525	298	149	0.749	0.5463	0.203	0.203	4.68208E-06	0.000118
SE	chr4	Npr2	+	43643054-43643174:43643335-43643409:43643606-43643683	223	149	0.5423	0.7453	-0.203	0.203	1.3709E-06	4.02E-05
SE	chr5	Slc15a4	-	127604111-127604188:127604538-127604706:127608772-127609061	298	149	0.9527	0.75	0.203	0.203	5.79526E-06	0.000142
SE	chr6	Plekha5	+	140579427-140579476:140580463-140580535:140581968-140582042	221	149	0.9333	0.73	0.203	0.203	1.51196E-08	7.05E-07
SE	chr8	Camsap3	+	3599217-3599249:3600358-3600405:3600676-3600814	196	149	0.6253	0.422	0.203	0.203	4.95433E-05	0.000917
SE	chr17	Slc8a1	-	81432644-81432774:81437745-81437813:81441773-81441790	217	149	0.13	0.3327	-0.203	0.203	4.85484E-09	2.57E-07
SE	chr16	Fgd4	-	16462001-16462059:16469306-16469372:16469704-16469842	215	149	0.1087	0.3117	-0.203	0.203	4.27261E-05	0.000805
SE	chr16	Fyttd1	+	32898878-32898939:32900633-32900707:32902419-32902545	223	149	0.965	0.7617	0.203	0.203	0	0
SE	chr17	3110052M02Rik	+	21652267-21652345:21656096-21656332:21657519-21657611	298	149	0.865	0.662	0.203	0.203	1.60392E-06	4.61E-05
SE	chr19	Sorbs1	-	40336986-40337035:40340045-40340146:40344356-40344439	250	149	0.8757	0.6723	0.203	0.203	0.005328368	0.048704
A3SS	chr4	Hook1	+	96014808-96014901:96015665-96017025:96016943-96017025	298	149	0.986	0.784	0.202	0.202	7.85707E-10	9.95E-08
A3SS	chr2	Pdss1	+	22929744-22929857:22934319-22935639:22935559-22935639	298	149	0.5983	0.396	0.202	0.202	0.001275149	0.01844
A5SS	chr11	Cdkl3	+	52004187-52004417:52004187-52004510:52004913-52005092	241	149	0.5	0.298	0.202	0.202	0.000585317	0.007401
MXE	chr5	Vps37d	-	135072899-135073983:135074394-135074476:135076458-135076645:13507ᵋ	298	231	0.09	0.292	-0.202	0.202	1.71953E-10	2.08E-08
MXE	chr17	Ptprs	-	56426387-56426455:56427939-56428132:56428852-56429157:56434453-564	298	298	0.981	0.779	0.202	0.202	0	0
RI	chr9	Ip6k2	+	108796064-108796396:108796064-108796572:108796541-108796572	292	149	0.7323	0.53	0.202	0.202	1.02771E-11	4.22E-10
SE	chr17	Phf10	-	14944994-14945184:14946199-14946387:14946655-14946763	298	149	0.997	0.7953	0.202	0.202	0	0
SE	chr16	Anks3	-	4957641-4957762:4960397-4960568:4964120-4964203	298	149	0.444	0.2423	0.202	0.202	5.10195E-07	1.68E-05
SE	chr2	Mtg2	+	180084064-180084282:180084451-180084582:180085380-180085902	280	149	0.9553	0.7533	0.202	0.202	7.71117E-05	0.001337
SE	chr2	Csrnp3	+	65845767-65845884:65860531-65860692:65877965-65878052	298	149	0.4417	0.6433	-0.202	0.202	1.47702E-05	0.00032
SE	chrX	Map7d2	+	159459378-159459483:159460267-159460365:159462114-159462224	247	149	0.9103	0.7087	0.202	0.202	7.56188E-08	3E-06
SE	chr2	Upf2	+	5951457-5951553:5952428-5952621:5957471-5957850	298	149	0.2207	0.423	-0.202	0.202	0.001711813	0.018737
SE	chr4	Adgrb2	+	130014551-130014636:130014935-130015033:130017062-130017233	247	149	0.2367	0.4383	-0.202	0.202	1.18794E-14	1.66E-12
SE	chr3	Srsf11	-	158023243-158024231:158024698-158024761:158026725-158026858	212	149	0.876	0.674	0.202	0.202	4.18317E-05	0.00079
SE	chr13	Cenph	-	100759686-100760194:100761761-100761924:100763550-100763601	298	149	0.983	0.7807	0.202	0.202	8.15289E-07	2.55E-05
A3SS	chr2	Slc39a13	-	91066013-91066126:91065582-91065739:91065582-91065700	187	149	0.2847	0.4853	-0.201	0.201	0.001347832	0.019206
A5SS	chr10	Izumo4	+	80704418-80704435:80704418-80704472:80704699-80704713	185	149	0.4477	0.2467	0.201	0.201	5.81614E-05	0.001151
A5SS	chr12	Smek1	-	101053468-101053584:101053429-101053584:101051531-101051662	187	149	0.291	0.492	-0.201	0.201	0.005140038	0.040877
MXE	chr4	Gm11837	+	14936441-14936539:14942200-14942275:14947439-14947545:14952621-149	224	255	0.0533	0.2543	-0.201	0.201	9.2826E-06	0.000298
MXE	chr6	Ccdc136	+	29414786-29414980:29415431-29415655:29421991-29422097:29426625-294	298	255	0.4063	0.6077	-0.201	0.201	0.000881678	0.013578
MXE	chr3	Nbea	-	55992334-55992514:56000487-56000640:56002724-56002941:56004534-560	298	298	0.7457	0.5447	0.201	0.201	3.25696E-11	4.73E-09
MXE	chrX	Atp11c	-	60229165-60229252:60229923-60230013:60236372-60236437:60236557-602	214	239	0.314	0.113	0.201	0.201	0.002610534	0.031764
RI	chr4	Srsf4	+	131884276-131884482:131884276-131885492:131885309-131885492	298	149	0.4383	0.237	0.201	0.201	2.73981E-05	0.000279
RI	chr2	Rbm39	-	156172828-156174168:156172828-156177385:156177336-156177385	298	149	0.852	0.6507	0.201	0.201	3.59802E-06	4.79E-05
RI	chr6	Magi1	-	93675453-93680877:93675453-93683090:93682952-93683090	298	149	0.8863	0.6853	0.201	0.201	1.19326E-08	2.74E-07
SE	chr8	Borcs8	+	70145024-70145134:70145811-70145890:70147090-70148635	228	149	0.961	0.7603	0.201	0.201	9.21546E-07	2.84E-05
SE	chr13	Tmem14c	+	41016250-41016398:41017115-41017173:41017706-41017782	207	149	0.81	0.609	0.201	0.201	6.53377E-12	5.81E-10
SE	chr13	Pfkp	-	6588489-6588653:6597859-6598040:6598739-6598826	298	149	0.7473	0.5467	0.201	0.201	0.003822878	0.036969
SE	chr3	Mfsd8	-	40821863-40822110:40822195-40822312:40823827-40823961	266	149	0.9897	0.7883	0.201	0.201	2.88963E-08	1.27E-06
SE	chr19	Chuk	-	44084417-44084478:44086908-44087059:44087916-44088039	298	149	0.9713	0.7707	0.201	0.201	1.37209E-11	1.15E-09
SE	chr5	Ogfod2	+	124113429-124113528:124114065-124114192:124114388-124114645	276	149	0.6187	0.8193	-0.201	0.201	3.26038E-05	0.000641
SE	chr9	Map4	+	110079781-110079893:110081421-110081610:110081841-110083173	298	149	0.358	0.5587	-0.201	0.201	2.56462E-14	3.38E-12
SE	chrX	Timm17b	+	7899336-7899469:7900961-7901060:7903673-7903736	248	149	0.945	0.7437	0.201	0.201	0	0
SE	chr6	Tmem209	-	30508454-30508512:30508666-30508842:30509685-30509783	298	149	0.782	0.581	0.201	0.201	0.001594258	0.017643
SE	chr15	Hsf1	+	76499948-76500053:76500131-76500196:76500267-76500336	214	149	0.4757	0.677	-0.201	0.201	0.001819892	0.019708
SE	chr11	Snx11	-	96773136-96773222:96774441-96774495:96776968-96777061	203	149	0.932	0.731	0.201	0.201	2.60073E-11	2.07E-09
MXE	chr12	Myt1l	+	29783855-29783888:29784251-29784313:29811373-29811734:29832288-298	211	298	0.794	0.5943	0.2	0.2	8.77876E-08	5.51E-06
MXE	chr18	Mapk4	-	73935006-73935167:73937130-73937274:73969891-73971276:74063924-740	298	293	0.1423	0.3423	-0.2	0.2	0.000732586	0.011708
MXE	chr7	Fam71e1	+	44496588-44496730:44496915-44497033:44497503-44497633:44500150-445	267	279	0.7157	0.516	0.2	0.2	0.001254873	0.017998
MXE	chr1	Glb1l	-	75202408-75202466:75202575-75202749:75202847-75202941:75208319-752	243	298	0.3773	0.577	-0.2	0.2	0.004012194	0.044039
MXE	chr10	Cnot2	-	116501192-116501317:116517317-116517383:116527827-116527949:116537	271	215	0.6877	0.8873	-0.2	0.2	3.27323E-05	0.000885
RI	chr3	Hltf	+	20116572-20116743:20116572-20118490:20117778-20118490	298	149	0.771	0.5707	0.2	0.2	0.00089166	0.005679
RI	chr6	Bms1	-	118392544-118392724:118392544-118392991:118392803-118392991	226	149	0.3517	0.552	-0.2	0.2	0.000788203	0.005151
SE	chr5	Depdc5	+	32955853-32955958:32959281-32959346:32964632-32964783	214	149	0.892	0.6923	0.2	0.2	3.80684E-05	0.000728
SE	chr6	Ptcd3	-	71894287-71894372:71894821-71894881:71895905-71895992	209	149	0.963	0.7633	0.2	0.2	3.79696E-14	4.89E-12
SE	chr9	Tle3	+	61394608-61394682:61401853-61402057:61407354-61407473	298	149	0.914	0.7137	0.2	0.2	0.000428258	0.0058
SE	chr2	Mtg2	+	180071794-180071886:180078619-180078823:180082086-180082233	298	149	0.9243	0.7247	0.2	0.2	1.44331E-07	5.38E-06
SE	chr4	Fancg	-	43004559-43004605:43004744-43005033:43005211-43005277	298	149	1	0.7997	0.2	0.2	4.40002E-08	1.84E-06
SE	chr4	Kif1b	-	149214907-149215025:149220542-149220790:149222227-149222364	298	149	0.9577	0.758	0.2	0.2	0	0
SE	chr9	Sec22c	-	121695534-121695742:121700553-121700677:121704924-121705029	273	149	0.203	0.4027	-0.2	0.2	1.26697E-06	3.76E-05
SE	chr11	Crlf3	-	80046493-80047737:80048592-80048704:80056452-80056584	261	149	0.862	0.6623	0.2	0.2	0.000323602	0.004551
SE	chr5	Magi2	+	19907091-19908013:19999442-19999618:20065552-20065671	298	149	0.0533	0.253	-0.2	0.2	7.54952E-15	1.11E-12
SE	chr2	Bbs5	+	69654063-69654112:69654246-69654373:69655499-69655634	276	149	0.9477	0.748	0.2	0.2	1.4949E-05	0.000324
SE	chr9	Rnf214	-	45898432-45898491:45900211-45900256:45904806-45904919	194	149	0.794	0.5943	0.2	0.2	0.001349415	0.015398
SE	chr12	Synj2bp-cox16	-	81480931-81481002:81504544-81504639:81532716-81532911	244	149	0.9223	0.7227	0.2	0.2	1.66307E-05	0.000355
A3SS	chr2	Strbp	-	37580673-37580806:37577770-37579802:37577770-37579468	298	149	1	0.8007	0.199	0.199	1.43795E-06	6.87E-05
A3SS	chr14	Erc2	+	28317249-28317314:28383824-28384043:28383906-28384043	230	149	0.9847	0.7853	0.199	0.199	5.29839E-07	2.92E-05
A3SS	chr7	Brsk2	+	142000842-142000889:142002063-142004244:142002475-142004244	298	149	0.7037	0.5047	0.199	0.199	0.000387927	0.007178
RI	chr16	Abcc5	-	20400761-20401071:20400761-20402334:20402260-20402334	298	149	0.9503	0.7517	0.199	0.199	2.1233E-12	9.49E-11
SE	chr6	Zfp638	+	83954711-83954761:83956694-83956762:83957509-83957592	217	149	0.8157	0.6167	0.199	0.199	2.88853E-06	7.68E-05
SE	chr11	Ikzf1	+	11740950-11741009:11748311-11748571:11754007-11754174	298	149	0.956	0.7567	0.199	0.199	5.84141E-05	0.001056
SE	chr4	Epha7	+	28943323-28943378:28947540-28947653:28948598-28948783	262	149	1	0.8007	0.199	0.199	4.16452E-07	1.39E-05
SE	chr7	Dmpk	+	19087240-19087333:19087494-19087700:19088001-19088109	298	149	0.9553	0.7563	0.199	0.199	3.79818E-05	0.000727
SE	chr10	Rufy2	+	62994705-62994770:62995313-62995379:62997881-62997982	215	149	0.9693	0.7703	0.199	0.199	1.46439E-10	1.01E-08
SE	chr7	Cog7	-	121936954-121937140:121941170-121941235:121943778-121943894	214	149	0.843	0.644	0.199	0.199	0.001523859	0.017006
SE	chr18	Ammecr1l	+	31775987-31776077:31776790-31776860:31778857-31778953	219	149	0.0567	0.2553	-0.199	0.199	5.19633E-08	2.14E-06
SE	chr14	Appl1	-	26959425-26959466:26960951-26961038:26962806-26962877	236	149	0.9847	0.7853	0.199	0.199	0	0
SE	chr2	Arfgap1	+	180977320-180977352:180978412-180978441:180979171-180979227	178	149	0.5907	0.3917	0.199	0.199	2.76131E-09	1.54E-07
SE	chr2	Rpn2	+	157321743-157321872:157323223-157323270:157325711-157326057	196	149	0.6353	0.4363	0.199	0.199	1.53514E-10	1.06E-08
SE	chr2	Optn	-	5045987-5046199:5054540-5054716:5063907-5063938	298	149	0.6477	0.8463	-0.199	0.199	0.002803334	0.028468
SE	chr9	Gramd2	+	59707764-59708095:59709452-59709509:59709862-59709937	206	149	0.983	0.7843	0.199	0.199	5.79174E-07	1.88E-05
A3SS	chr16	Abcc5	-	20402260-20402334:20400761-20401071:20400761-20401034	185	149	0.8823	0.6847	0.198	0.198	9.97789E-07	5.07E-05
A3SS	chr7	Tial1	-	128448121-128448208:128446731-128447107:128446731-128446798	298	149	0.742	0.9403	-0.198	0.198	2.05391E-14	5.29E-12
MXE	chr6	Ccdc136	+	29414786-29414980:29415431-29415655:29422037-29422097:29426625-294	298	209	0.445	0.643	-0.198	0.198	0.003291147	0.037984
MXE	chr12	Lrfn5	+	61669480-61669654:61768008-61768077:61839408-61840812:61850992-618	218	298	0.172	0.37	-0.198	0.198	1.16577E-06	4.99E-05
SE	chr15	Scube1	-	83618602-83618689:83620056-83620210:83621029-83621133	298	149	1	0.8023	0.198	0.198	8.03545E-09	4.04E-07
SE	chr16	Anks3	-	4941415-4941797:4941899-4941987:4942693-4942778	237	149	1	0.8023	0.198	0.198	2.38932E-07	8.45E-06
SE	chr13	Fam193b	-	55549118-55549357:55549962-55550007:55550224-55550407	194	149	0.307	0.109	0.198	0.198	5.80377E-09	3.01E-07
SE	chr7	Dmpk	+	19086489-19086584:19086893-19087041:19087240-19087333	297	149	0.872	0.6737	0.198	0.198	0.001792595	0.019491
SE	chrX	Aifm1	-	48499772-48499871:48503583-48503713:48513236-48513563	279	149	0.8277	0.6293	0.198	0.198	0.00083288	0.010233
SE	chr1	Rps6kc1	-	190783564-190783646:190798652-190798741:190799013-190800581	238	149	0.992	0.7937	0.198	0.198	3.33067E-16	5.56E-14
SE	chr16	Rbfox1	+	7375905-7375969:7391988-7392040:7407964-7408039	201	149	0.477	0.6747	-0.198	0.198	6.55711E-08	2.64E-06
SE	chr1	Ipo9	-	135420292-135420353:135426931-135427041:135430268-135430419	259	149	0.298	0.496	-0.198	0.198	2.5492E-07	8.97E-06
SE	chr10	Herc4	+	63299120-63299239:63303845-63303868:63306348-63306446	172	149	0.7773	0.5797	0.198	0.198	6.32015E-05	0.001125
SE	chr15	Slc25a32	-	39098129-39098242:39099873-39100033:39112348-39112716	298	149	1	0.8017	0.198	0.198	2.01991E-06	5.66E-05
SE	chr7	Shkbp1	-	27345173-27345328:27346971-27347172:27348332-27348448	298	149	0.9723	0.7743	0.198	0.198	7.72923E-07	2.44E-05
A3SS	chr7	Myo7a	-	98066341-98066467:98065612-98065895:98065612-98065781	262	149	0.1387	0.3353	-0.197	0.197	0.002334048	0.028651
A5SS	chr2	Gapvd1	-	34717316-34717382:34717253-34717382:34715205-34715330	211	149	0.8503	0.6537	0.197	0.197	1.67776E-08	1.13E-06
A5SS	chr12	Dtnb	+	3773551-3773640:3773551-3774610:3779619-3779654	298	149	0.9003	0.703	0.197	0.197	9.48667E-06	0.00025
MXE	chr3	Gm3764	+	88209635-88209716:88220079-88221050:88221103-88221284:88225944-882	298	298	0.4283	0.2313	0.197	0.197	8.91442E-05	0.00205
MXE	chrX	Slc25a14	+	48623578-48623828:48623962-48624206:48628136-48628183:48629252-486	298	196	0.864	0.6667	0.197	0.197	0.000187384	0.003845

MXE	chr10	Pex7	-	19887060-19887173:19888656-19888762:19894250-19894358:19904563-199	257	255	0.0657	0.2623	-0.197	0.197	1.55058E-06	6.35E-05
RI	chr3	Thbs3	+	89216681-89216887:89216681-89217941:89217685-89217941	298	149	0.443	0.2463	0.197	0.197	7.4996E-11	2.61E-09
SE	chr3	Ank2	-	127104761-127104862:127248134-127248232:127498668-127500347	247	149	0.5437	0.7403	-0.197	0.197	0.000454551	0.006111
SE	chr19	Trpm3	+	22733000-22733171:22749771-22749845:22766634-22766808	223	149	0.179	0.3763	-0.197	0.197	1.38606E-06	4.06E-05
SE	chr18	Mapk4	-	73935006-73935167:73937130-73937274:73969891-73971276	293	149	0.949	0.7523	0.197	0.197	7.12804E-06	0.000171
SE	chr1	Cdc42bpa	+	180084372-180084505:180093944-180094186:180094381-180094491	298	149	0.9027	0.7057	0.197	0.197	6.07572E-08	2.47E-06
SE	chr2	Ncoa5	-	165012836-165013162:165023146-165023212:165034683-165034779	215	149	0.7917	0.5943	0.197	0.197	1.11022E-15	1.75E-13
SE	chr5	Cdc7	+	106976630-106976712:106979316-106979444:106982964-106984439	277	149	0.94	0.7433	0.197	0.197	1.30807E-05	0.000289
SE	chr10	Coq10a	-	128364405-128364506:128365023-128365245:128368007-128368153	298	149	0.9767	0.7797	0.197	0.197	3.44158E-12	3.19E-10
SE	chr7	Ube3a	+	59247173-59247217:59262022-59262058:59271947-59272236	185	149	0.0473	0.244	-0.197	0.197	1.87295E-13	2.18E-11
SE	chr12	Trim9	-	70250957-70251260:70252443-70252484:70268062-70268200	190	149	0.2473	0.444	-0.197	0.197	0.003105217	0.031004
SE	chr10	Jmjd1c	+	67215270-67215375:67218038-67218181:67218277-67218469	292	149	0.96	0.7627	0.197	0.197	2.50595E-06	6.8E-05
SE	chr18	Cables1	+	11888737-11888829:11905925-11906002:11923360-11923456	226	149	0.4037	0.2063	0.197	0.197	0.002736759	0.027903
A3SS	chr2	Ehmt1	-	24877455-24877626:24863772-24863929:24863772-24863908	169	149	0.3503	0.154	0.196	0.196	1.08168E-07	7.96E-06
A3SS	chr2	Arfgap1	+	180979661-180979720:180980398-180980474:180980404-180980474	154	149	0.939	0.743	0.196	0.196	0.000102215	0.002483
A5SS	chr17	Slc25a27	-	43664078-43664265:43664074-43664265:43661614-43661698	152	149	0.829	0.6333	0.196	0.196	3.99812E-06	0.000123
A5SS	chr15	Chkb	-	89428714-89428827:89428411-89428827:89428134-89428229	298	149	0.9827	0.7863	0.196	0.196	0	0
A5SS	chrX	Armcx2	-	134807621-134807725:134807454-134807725:134807262-134807327	298	149	0.8573	0.661	0.196	0.196	1.92145E-11	2.23E-09
A5SS	chr9	Cep70	+	99277937-99278018:99277937-99278161:99281028-99281102	291	149	1	0.804	0.196	0.196	1.12365E-06	4.21E-05
MXE	chr2	Mtg2	+	180070593-180070615:180071794-180071886:180078619-180078823:18008	241	298	0.06	0.2563	-0.196	0.196	7.33478E-08	4.74E-06
RI	chrX	C77370	-	104076761-104081102:104076761-104083283:104083154-104083283	298	149	0.96	0.7637	0.196	0.196	8.23785E-14	4.69E-12
RI	chr11	Zfp207	+	80403365-80403408:80403365-80403662:80403601-80403662	298	149	0.7657	0.5697	0.196	0.196	4.35083E-10	1.33E-08
RI	chr3	Tpm3	+	90090010-90090079:90090010-90091091:90091013-90091091	298	149	0.3017	0.1057	0.196	0.196	2.19758E-07	3.93E-06
SE	chr5	Asphd2	-	112391158-112392255:112393785-112393999:112394718-112394801	298	149	0.3427	0.539	-0.196	0.196	5.73515E-10	3.59E-08
SE	chr6	Dgki	-	36975147-36975247:36994859-36994921:37000042-37000241	211	149	0.7473	0.551	0.196	0.196	0.001880604	0.020252
SE	chr5	Aasdh	-	76901918-76902185:76904209-76904380:76905423-76905514	298	149	0.792	0.988	-0.196	0.196	1.0592E-06	3.21E-05
SE	chr14	Fam149b	+	20352704-20352839:20363263-20363450:20367846-20367952	298	149	0.8043	1	-0.196	0.196	6.26916E-09	3.23E-07
SE	chr9	Bmper	+	23373833-23373942:23375566-23375706:23377638-23377742	289	149	1	0.8043	0.196	0.196	3.03342E-06	7.99E-05
SE	chr8	Gse1	+	120572636-120572906:120574171-120574278:120574934-120575305	256	149	0.2317	0.428	-0.196	0.196	1.2725E-05	0.000283
SE	chr15	Trmu	+	85893954-85894020:85894926-85895026:85896360-85896504	249	149	0.9517	0.7557	0.196	0.196	8.13787E-09	4.09E-07
SE	chr12	Trim9	-	70248794-70251260:70255038-70255226:70268062-70268200	298	149	0.1217	0.3177	-0.196	0.196	3.91735E-05	0.000746
A3SS	chr17	Atl2	-	79852525-79852956:79848390-79850793:79848390-79850278	298	149	0.6403	0.4457	0.195	0.195	0.000488573	0.008627
MXE	chr8	Rltpr	+	105697217-105697438:105697561-105697641:105697868-105697945:105698	229	226	0.123	0.318	-0.195	0.195	9.76101E-10	9.7E-08
MXE	chr7	B230209E15Rik	-	61534745-61534785:61535076-61535488:61604516-61604700:61615160-616	298	298	0.4297	0.6243	-0.195	0.195	0.002223067	0.028197
MXE	chr4	Ptprd	-	76594267-76594305:76765750-76765758:76981506-76981539:77194296-771	182	207	0.5073	0.7027	-0.195	0.195	0.00436551	0.046937
MXE	chr2	Cstf3	+	104655689-104655791:104660519-104660714:104663294-104663447:104664	298	298	0.0183	0.2133	-0.195	0.195	1.75415E-14	4.53E-12
MXE	chr9	Hmgcll1	+	76056452-76056559:76072627-76072722:76130312-76130437:76133755-761	244	274	0.3537	0.5483	-0.195	0.195	8.79047E-05	0.002028
RI	chr15	Hdac10	-	89124175-89124253:89124175-89124588:89124532-89124588	298	149	0.5893	0.394	0.195	0.195	0.000113952	0.000967
RI	chr7	Atxn2l	-	126491708-126492215:126491708-126492608:126492449-126492608	298	149	0.592	0.397	0.195	0.195	9.49352E-13	4.4E-11
RI	chr5	Gigyf1	+	137520547-137520800:137520547-137521002:137520885-137521002	232	149	0.324	0.519	-0.195	0.195	0.000163165	0.001309
SE	chr4	Srsf4	+	131873612-131873870:131886131-131886304:131891207-131891349	298	149	0.1633	0.3587	-0.195	0.195	5.89761E-08	2.4E-06
SE	chr14	Pbrm1	+	31019138-31019293:31022720-31022859:31023067-31023170	288	149	0.645	0.45	0.195	0.195	9.17193E-05	0.001551
SE	chr4	Tnc	-	63976446-63976568:64000698-64000970:64006246-64006509	298	149	0.707	0.9017	-0.195	0.195	2.30926E-07	8.21E-06
SE	chr6	Plxna1	-	89335397-89335493:89337151-89337351:89340523-89340637	298	149	0.989	0.7943	0.195	0.195	0	0
SE	chr13	Rnf180	-	105181450-105181675:105246663-105246698:105252252-105252347	184	149	0.9847	0.79	0.195	0.195	9.97174E-08	3.84E-06
SE	chr11	Lyrm7	-	54839289-54841202:54848579-54848660:54850345-54850412	230	149	0.903	0.7077	0.195	0.195	0.00011284	0.001849
SE	chr7	Actn4	-	28909903-28909988:28911490-28911575:28912240-28912321	234	149	0.1437	0.339	-0.195	0.195	1.02312E-06	3.12E-05
SE	chr9	Vstm5	+	15238918-15239358:15257230-15257556:15257651-15257791	298	149	0.9907	0.7953	0.195	0.195	4.91793E-11	3.73E-09
SE	chr8	Mri1	-	84250576-84251711:84253863-84254087:84254176-84254352	298	149	0.9777	0.7823	0.195	0.195	1.16757E-11	9.93E-10
SE	chr8	Evi5l	+	4200868-4200921:4201934-4201966:4202936-4203076	181	149	0.8383	0.6433	0.195	0.195	1.85335E-10	1.25E-08
A3SS	chr7	Chmp2a	-	13033829-13034042:13032289-13032565:13032289-13032350	298	149	0.7243	0.9183	-0.194	0.194	0.001803508	0.023576
A5SS	chr7	Eed	-	89956344-89956413:89956255-89956413:89955531-89955604	237	149	0.8387	0.645	0.194	0.194	1.53734E-06	5.5E-05
MXE	chr2	Myef2	-	125097563-125097634:125097977-125098075:125098450-125098518:12510	217	247	0.1607	0.355	-0.194	0.194	0	0
RI	chr15	Trps1	-	50830829-50831006:50830829-50831958:50831607-50831958	298	149	0.884	0.6897	0.194	0.194	0.006500261	0.030298
RI	chr7	Dyrk1b	+	28185714-28185749:28185714-28186029:28185834-28186029	232	149	0.9603	0.766	0.194	0.194	5.05104E-10	1.51E-08
SE	chr3	Gba	+	89206134-89206368:89206760-89206812:89207227-89207315	201	149	0.0113	0.2057	-0.194	0.194	6.02851E-14	7.6E-12
SE	chr9	Hmgn3	-	83109942-83110429:83110691-83110874:83110993-83111106	298	149	0.2967	0.103	0.194	0.194	1.75315E-11	1.44E-09
SE	chr19	Trpm3	+	22897670-22897810:22900134-22900163:22901046-22901335	178	149	0.9	0.7057	0.194	0.194	0.000204303	0.003079
SE	chr4	Hnrnpr	+	136310942-136311089:136314087-136314255:136316361-136316479	298	149	0.7373	0.5433	0.194	0.194	6.85341E-13	7.24E-11
SE	chr7	Rdh13	-	4442594-4442749:4443737-4443771:4444203-4444321	183	149	0.3617	0.1673	0.194	0.194	1.13185E-05	0.000256
SE	chr16	Itsn1	+	91854573-91854655:91861194-91861239:91863257-91863378	194	149	1	0.8063	0.194	0.194	9.74776E-14	1.18E-11
SE	chr9	Lingo1	-	56685120-56685281:56724301-56724345:56775539-56775648	193	149	0.7113	0.9057	-0.194	0.194	0.000235238	0.003472
A3SS	chr15	Hdac10	-	89124532-89124588:89124175-89124428:89124175-89124253	298	149	0.4553	0.2623	0.193	0.193	0.000182065	0.003898
MXE	chr9	Clasp2	+	113854173-113854346:113858581-113858604:113860120-113860137:113862	172	166	0.6643	0.857	-0.193	0.193	4.29551E-08	2.99E-06
MXE	chr3	Adgrl2	-	148815855-148817928:148818648-148818686:148820451-148820468:148821	166	187	0.133	0.3263	-0.193	0.193	0.001022908	0.015245
RI	chr2	Klhl23	+	69833675-69835250:69833675-69836651:69836094-69836651	298	149	0.9907	0.7977	0.193	0.193	0	0
SE	chr8	Eps15l1	-	72340990-72341524:72358313-72358445:72367904-72367986	281	149	0.5267	0.72	-0.193	0.193	1.18408E-05	0.000265
SE	chr10	Psap	+	60295946-60296002:60297537-60297545:60299098-60299229	157	149	0.8063	0.6137	0.193	0.193	0	0
SE	chr17	Zfp871	-	32777157-32777217:32777401-32777527:32788138-32788287	275	149	0.986	0.7933	0.193	0.193	2.13163E-14	2.85E-12
SE	chr17	Nrxn1	-	90704066-90704367:90991367-90991378:90992115-90992144	160	149	0.6007	0.794	-0.193	0.193	0.000558356	0.007301
SE	chr8	Orc6	+	85299632-85299992:85301109-85301238:85302796-85302959	278	149	0.6443	0.837	-0.193	0.193	5.8521E-05	0.001057
SE	chr6	Plekha5	+	140579427-140579476:140580347-140580535:140581968-140582088	298	149	0.9557	0.763	0.193	0.193	2.52262E-11	2.02E-09
SE	chr5	Atp8a1	-	67764864-67764991:67767020-67767185:67769052-67769163	298	149	0.988	0.795	0.193	0.193	6.73417E-12	5.95E-10
SE	chr12	Sypl	+	32967536-32967743:32968634-32968643:32974128-32974319	158	149	0.5127	0.32	0.193	0.193	0.001515	0.016919
SE	chrX	Huwe1	+	151857808-151857913:151859455-151859688:151860167-151860268	298	149	0.4003	0.2077	0.193	0.193	4.77305E-07	1.58E-05
A3SS	chr4	Ccnl2	+	155817923-155817987:155818539-155820428:155820329-155820428	298	149	0.7747	0.9667	-0.192	0.192	0	0
MXE	chr7	Fgfr2	-	130218998-130219121:130228623-130228792:130261749-130262066:130265	298	298	0.273	0.465	-0.192	0.192	0.003923891	0.043413
MXE	chr12	Meg3	+	109544027-109544742:109544948-109545041:109546430-109546542:109549	242	261	0.1287	0.3203	-0.192	0.192	0	0
RI	chr11	Cdkl3	+	52004187-52004417:52004187-52005092:52004913-52005092	298	149	0.461	0.2687	0.192	0.192	0.000244467	0.001832
RI	chr19	Kat5	-	5609852-5609918:5609852-5610094:5610018-5610094	247	149	0.635	0.4433	0.192	0.192	0.01006607	0.04344
SE	chr1	Tfb2m	-	179531304-179531464:179532065-179532127:179533633-179533722	211	149	0.951	0.7587	0.192	0.192	5.7444E-09	2.99E-07
SE	chrX	Rragb	+	153143976-153144075:153148452-153148535:153150362-153150428	232	149	0.8797	0.6873	0.192	0.192	1.99629E-12	1.96E-10
SE	chr6	Nup210	-	91054390-91054496:91055225-91055417:91060562-91060735	298	149	0.993	0.801	0.192	0.192	1.93412E-12	1.91E-10
SE	chrX	Irak1	-	74020943-74021061:74021849-74021963:74022185-74022249	263	149	0.9143	0.7223	0.192	0.192	3.78499E-07	1.27E-05
SE	chr4	Plaa	-	94587487-94587587:94589881-94590074:94602877-94603247	298	149	0.7263	0.534	0.192	0.192	7.22992E-06	0.000173
SE	chr7	Tial1	-	128443807-128443946:128444042-128444100:128444281-128444404	207	149	0.0793	0.2717	-0.192	0.192	0	0
A3SS	chr11	Aldh3a2	-	61248862-61249097:61247929-61248482:61247929-61248041	298	149	0.4013	0.2107	0.191	0.191	0.003010465	0.035797
A3SS	chr5	Gabra4	-	71633546-71633698:71623855-71624114:71623855-71623980	282	149	0.7787	0.9693	-0.191	0.191	4.96565E-07	2.84E-05
A3SS	chr6	Magi1	-	93682952-93683090:93675453-93679002:93675453-93679002	298	149	0.5347	0.3433	0.191	0.191	4.83162E-05	0.001341
A3SS	chr1	Ivns1abp	+	151354634-151354759:151355167-151356236:151356129-151356236	298	149	0.3673	0.5587	-0.191	0.191	1.9984E-15	6.27E-13
MXE	chr17	Nrxn1	-	90163792-90163881:90208323-90208494:90362760-90362941:90560741-905	298	298	0.5037	0.3123	0.191	0.191	2.33969E-10	2.79E-08
MXE	chr16	Dnm1l	-	16338593-16338679:16340996-16341067:16341422-16341468:16345998-163	195	220	0.429	0.2383	0.191	0.191	1.03364E-07	6.41E-06
MXE	chr18	Nedd4l	+	65165572-65165646:65174045-65174492:65178919-65178996:65181328-651	298	226	0.012	0.203	-0.191	0.191	0	0
MXE	chr16	Eif4g1	+	20672720-20672876:20674228-20674314:20674786-20674806:20675344-206	235	169	0.2833	0.474	-0.191	0.191	4.25138E-05	0.00111
RI	chr3	Gm3764	+	88227366-88227624:88227366-88228694:88228559-88228694	298	149	0.8133	0.6223	0.191	0.191	3.21965E-13	1.68E-11
RI	chr2	St6galnac4	+	32587078-32587227:32587078-32587681:32587581-32587681	298	149	0.482	0.291	0.191	0.191	0.000206715	0.001607
SE	chr4	Nfib	-	82290173-82296811:82323572-82323710:82327729-82327913	287	149	0.101	0.2917	-0.191	0.191	6.77048E-11	4.97E-09
SE	chr10	Grip1	+	119453835-119454422:119897715-119897795:119929901-119930036	229	149	0.592	0.7833	-0.191	0.191	0.000357443	0.00496
SE	chr5	Epha5	-	84107989-84108056:84117561-84117626:84142278-84142383	214	149	0.6857	0.4943	0.191	0.191	1.77118E-05	0.000376
SE	chr14	Ppif	+	25696004-25696092:25697573-25697669:25698266-25698341	245	149	0.8277	0.6363	0.191	0.191	0.000306505	0.004352
SE	chr9	Map4	+	110068693-110068985:110071026-110071139:110078003-110078084	262	149	0.1007	0.2913	-0.191	0.191	6.54554E-12	5.81E-10
SE	chr10	Mdm1	+	118164201-118164447:118166625-118166684:118168051-118168999	208	149	0.8917	0.7007	0.191	0.191	0.000282634	0.004067
SE	chr10	Fyn	+	39526781-39526930:39529919-39530074:39532016-39532195	298	149	0.9733	0.7823	0.191	0.191	1.99569E-07	7.21E-06
SE	chr5	Zcchc4	+	52796582-52796659:52804379-52804451:52806957-52807107	221	149	0.944	0.753	0.191	0.191	0.000320079	0.004513
MXE	chr9	Pcsk7	+	45919792-45919894:45925911-45926067:45926734-45926828:45927037-459	298	243	0.682	0.8723	-0.19	0.19	2.49571E-05	0.000703
MXE	chr5	Prkag2	-	24871494-24871659:24873884-24874010:24875161-24875215:24876989-248	203	275	0.4467	0.637	-0.19	0.19	1.77856E-06	7.1E-05
MXE	chr17	Gm7072	-	22289769-22289895:22292408-22292455:22309505-22309597:22313237-223	241	196	0.7973	0.607	0.19	0.19	0.004339227	0.046687
MXE	chr18	Fbn2	-	58093294-58093419:58095182-58095235:58095910-58096062:58102330-581	298	202	0.5887	0.7787	-0.19	0.19	0.003801727	0.042431
RI	chr11	Rai1	+	60193939-60194032:60193939-60197838:60197789-60197838	298	149	0.4727	0.2827	0.19	0.19	8.934E-09	2.15E-07
SE	chr2	Ptpra	+	130503994-130504335:130509830-130509856:130517731-130517815	175	149	0.3413	0.5317	-0.19	0.19	5.78773E-08	2.36E-06
SE	chr8	Mthfsd	-	121101444-121101536:121101803-121101922:121102914-121103004	268	149	0.092	0.2823	-0.19	0.19	7.51438E-07	2.38E-05
SE	chr11	B9d1	+	61506344-61506412:61507628-61507739:61509470-61509532	260	149	0.8783	0.6887	0.19	0.19	0.000191504	0.002914
SE	chr5	Ablim2	+	35857826-35857964:35873170-35873224:35874633-35874640	203	149	0.0237	0.2137	-0.19	0.19	2.38515E-08	1.06E-06
SE	chr4	Pum1	+	130701042-130701110:130718154-130718262:130741463-130741556	257	149	0.6183	0.808	-0.19	0.19	0.000102687	0.001706
SE	chr13	Cntnap3	-	64748433-64748657:64751677-64751916:64757205-64757423	298	149	0.9887	0.799	0.19	0.19	1.56537E-08	7.27E-07
SE	chr8	Dcun1d2	-	13255963-13257976:13258947-13259043:13261385-13261467	245	149	0.914	0.724	0.19	0.19	1.66288E-05	0.000355
SE	chr9	Rasa2	-	96552303-96552409:96553113-96553186:96557400-96557477	222	149	0.9697	0.7797	0.19	0.19	2.88909E-05	0.000579
SE	chr4	Dock7	-	98946629-98946754:98953866-98953880:98955316-98955447	163	149	0.9907	0.8007	0.19	0.19	0	0
A3SS	chr6	Hipk2	-	38737358-38737520:38730869-38731076:38730869-38730995	229	149	0.4013	0.2123	0.189	0.189	0.000191323	0.004061
A3SS	chr5	Ddx55	+	124560718-124560800:124561856-124562037:124561906-124562037	198	149	0.118	0.3067	-0.189	0.189	2.22955E-05	0.000738
A5SS	chr5	Asphd2	-	112393785-112394174:112393139-112394174:112391158-112392255	298	149	0.347	0.158	0.189	0.189	7.43441E-07	2.99E-05
A5SS	chr10	Adarb1	-	77312378-77312526:77312348-77312526:77311106-77311274	178	149	0.6473	0.8367	-0.189	0.189	2.42787E-06	8.15E-05
MXE	chr1	Nfasc	-	132576286-132576417:132579378-132579508:132588074-132588310:13259	298	279	0.9407	0.7517	0.189	0.189	5.60246E-10	6E-08
SE	chr15	2310069G16Rik	+	44787763-44787890:44788485-44788623:44805235-44805829	287	149	0.9283	0.739	0.189	0.189	0.003471955	0.03406

SE	chr9	Dalrd3	+	108571782-108571864:108571941-108572117:108572187-108572297	298	149	0.985	0.7963	0.189	0.189	1.11022E-16	1.97E-14
SE	chr6	Phc1	-	122336858-122337015:122338472-122338480:122339598-122339622	157	149	0.517	0.328	0.189	0.189	0.003405361	0.03354
SE	chr13	Simc1	+	54523970-54526672:54528304-54528551:54529154-54529223	298	149	0.8593	0.6703	0.189	0.189	0.001989537	0.021273
SE	chr2	Ntng2	-	29196982-29197149:29198814-29198915:29204898-29204924	250	149	0.0273	0.216	-0.189	0.189	0	0
SE	chr6	Zfp248	-	118453846-118453887:118454677-118454911:118455416-118455506	298	149	0.9723	0.783	0.189	0.189	2.90304E-06	7.71E-05
SE	chrX	Cstf2	+	134072415-134072474:134072678-134072764:134073214-134073277	235	149	0.6573	0.8463	-0.189	0.189	0.000330647	0.004637
SE	chr7	Caly	-	140073941-140074100:140081381-140081538:140082093-140082301	298	149	0.327	0.5163	-0.189	0.189	0.001606454	0.017748
SE	chrX	Ogt	+	101640218-101640322:101643006-101643156:101643947-101644190	298	149	0.989	0.7997	0.189	0.189	0	0
A3SS	chr6	Nup205	+	35203963-35204013:35204695-35205534:35205325-35205534	298	149	0.2387	0.0503	0.188	0.188	1.89848E-14	5.07E-12
MXE	chr3	Dclk1	+	55502809-55502905:55506701-55506781:55516873-55516986:55521850-555	229	262	0.868	0.6797	0.188	0.188	0	0
MXE	chr14	Ccser2	-	36874936-36879762:36885751-36885790:36890294-36890426:36896255-368	281	188	0.5657	0.3777	0.188	0.188	0.000168639	0.003503
SE	chr3	Celsr2	-	108393340-108393510:108393650-108393832:108393936-108394024	298	149	0.814	0.6257	0.188	0.188	9.62368E-06	0.000222
SE	chr7	Mical2	+	112331896-112332039:112333829-112333954:112346722-112346908	274	149	0.893	0.7053	0.188	0.188	5.22042E-05	0.000959
SE	chr4	Eps15	+	109379867-109380029:109382785-109382966:109385319-109387666	298	149	0.834	0.646	0.188	0.188	3.47477E-08	1.5E-06
SE	chr19	Fads1	+	10194318-10194397:10194489-10194614:10194871-10196874	274	149	0.9577	0.7697	0.188	0.188	0	0
SE	chr12	Meg3	+	109545398-109546542:109549479-109549552:109549653-109549810	222	149	0.9517	0.764	0.188	0.188	0	0
SE	chr7	Saxo2	-	82645650-82645829:82645923-82646460:82648392-82648481	298	149	0.936	0.748	0.188	0.188	0.000278915	0.00402
SE	chr9	Lztfl1	-	123712430-123712624:123715300-123715424:123717526-123717557	273	149	0.942	0.7543	0.188	0.188	2.23223E-08	1E-06
SE	chr3	Csde1	+	103039933-103040042:103040441-103040533:103041145-103041242	241	149	0.3667	0.1783	0.188	0.188	0	0
SE	chr7	Brsk2	+	141998633-141998722:142000842-142000889:142002063-142004244	196	149	0.5787	0.7667	-0.188	0.188	0.000759465	0.009478
A3SS	chr7	Tsg101	-	46910745-46910810:46908905-46909068:46908905-46908991	225	149	0.9267	0.74	0.187	0.187	0	0
A3SS	chr17	Ptprs	-	56427791-56427817:56426556-56426653:56426556-56426563	238	149	0.1713	0.358	-0.187	0.187	0.001691431	0.022603
A5SS	chr14	Ndst2	-	20724748-20724820:20724711-20724820:20724461-20724563	185	149	0.8623	0.675	0.187	0.187	0.000773411	0.009434
A5SS	chr6	C87436	+	86438610-86438847:86438610-86439258:86443727-86443975	298	149	0.662	0.8493	-0.187	0.187	0.001003061	0.011624
MXE	chr1	Nckap5	-	125913636-125915021:125976926-125977062:125981383-125981697:126014	298	285	0.6363	0.8237	-0.187	0.187	0.000399128	0.007134
MXE	chr2	Oprl1	+	181715357-181715489:181715690-181715946:181716502-181716535:181717	298	182	0.9173	0.73	0.187	0.187	9.09449E-05	0.002076
SE	chr1	Enah	-	181930678-181930762:181931174-181931185:181956394-181956571	160	149	0.34	0.153	0.187	0.187	1.42614E-07	5.33E-06
SE	chr16	Trmt2a	+	18250820-18251001:18251359-18251474:18251549-18251660	264	149	0.763	0.95	-0.187	0.187	2.74974E-07	9.59E-06
SE	chr16	Slx4	-	3994708-3994920:3995694-3995877:3999118-3999345	298	149	0.8123	0.625	0.187	0.187	0.001248606	0.014429
SE	chr17	Wiz	-	32358943-32359494:32360038-32360427:32361461-32361964	298	149	0.5137	0.701	-0.187	0.187	4.56158E-06	0.000115
SE	chr17	Tulp4	+	6106828-6106996:6121828-6122344:6137211-6139156	298	149	0.226	0.4127	-0.187	0.187	2.73176E-08	1.2E-06
SE	chr6	P3h3	-	124845202-124845319:124845508-124845658:124845942-124846043	298	149	0.919	0.732	0.187	0.187	1.68291E-08	7.74E-07
SE	chr4	Dock7	-	98946629-98946763:98952264-98953880:98955316-98955447	298	149	0.6433	0.4563	0.187	0.187	0.002690207	0.027513
A5SS	chr10	Smarcc2	+	128487934-128488017:128487934-128488359:128488896-128490174	298	149	0.2983	0.112	0.186	0.186	0	0
A5SS	chr5	Nsun5	+	135374927-135375065:135374927-135375105:135375275-135375485	188	149	0.4883	0.3027	0.186	0.186	7.39688E-05	0.001412
MXE	chr11	Ogdh	+	6316765-6316855:6316978-6317080:6324878-6324922:6334533-6334648	251	193	0.3457	0.1593	0.186	0.186	1.0806E-07	6.64E-06
MXE	chr18	C330018D20Rik	-	56957088-56957921:56962401-56962518:56972805-56972943:56973384-569	287	266	0.0817	0.2677	-0.186	0.186	3.58218E-05	0.000958
MXE	chr5	Adgrl3	+	81771584-81771680:81787436-81787564:81792361-81792378:81794264-818	277	166	0.265	0.4513	-0.186	0.186	0.00445499	0.047731
RI	chr1	Fam134a	+	75146430-75146719:75146430-75147909:75146831-75147909	259	149	0.8853	0.6993	0.186	0.186	0	0
SE	chr17	Gpatch11	+	78835516-78835678:78836794-78836920:78837831-78837902	275	149	0.918	0.732	0.186	0.186	9.32936E-08	3.61E-06
SE	chr7	Mical2	+	112331896-112332039:112345246-112345353:112346722-112346908	256	149	0.9137	0.7273	0.186	0.186	1.05338E-05	0.00024
SE	chr12	Ccdc85c	-	108207834-108207929:108211515-108211619:108221709-108221782	253	149	0.947	0.761	0.186	0.186	1.14709E-07	4.39E-06
SE	chr16	Clec16a	+	10731661-10731819:10738392-10738515:10741614-10744877	272	149	0.4293	0.2437	0.186	0.186	0.000641757	0.008225
SE	chr6	Adcyap1r1	+	55487659-55487742:55491227-55491310:55494077-55494206	232	149	0.93	0.7443	0.186	0.186	3.27622E-05	0.000642
SE	chr17	Cyp4f17	+	32528047-32528111:32528276-32528363:32528717-32528809	236	149	0.2883	0.1027	0.186	0.186	0.00094594	0.01138
SE	chr1	Kansl1l	-	66801049-66802168:66807525-66807604:66817374-66817596	228	149	0.085	0.271	-0.186	0.186	3.6833E-05	0.000709
SE	chr3	Adgrl2	-	148815586-148817928:148821280-148821408:148822955-148823051	277	149	0.775	0.589	0.186	0.186	6.98116E-08	2.79E-06
SE	chr15	Hsf1	+	76495970-76496078:76496453-76496589:76497768-76497829	285	149	0.974	0.7883	0.186	0.186	6.68206E-09	3.41E-07
SE	chr15	Kifc2	+	76661236-76661384:76661480-76661618:76661727-76661872	287	149	0.9143	0.728	0.186	0.186	5.04485E-13	5.44E-11
SE	chr13	Gfm2	+	97150325-97150413:97153228-97153316:97155190-97155250	237	149	0.94	0.754	0.186	0.186	0.002767814	0.02815
SE	chr14	Exoc5	-	49011922-49014349:49015534-49015632:49016198-49016306	247	149	0.978	0.7923	0.186	0.186	5.32907E-15	7.89E-13
A5SS	chr17	Lclat1	+	73107985-73108145:73107985-73108156:73161823-73161991	159	149	0.644	0.829	-0.185	0.185	0.002834773	0.025991
MXE	chr15	Kctd17	+	78435585-78435710:78436901-78437099:78437158-78437232:78437584-784	298	223	0.8057	0.6203	0.185	0.185	5.49554E-05	0.001365
MXE	chrX	Slc25a14	+	48623578-48623828:48623962-48624197:48628136-48628183:48629252-486	298	196	0.8607	0.6753	0.185	0.185	0.000326185	0.006109
MXE	chr9	Zfp426	-	20473028-20473108:20474907-20475033:20476396-20476478:20478133-204	231	275	0.508	0.693	-0.185	0.185	0.000929065	0.014186
RI	chr1	Zcchc2	+	106029752-106031233:106029752-106034077:106032096-106034077	298	149	0.4003	0.2153	0.185	0.185	0.00587688	0.027912
RI	chr15	43711	+	82291253-82291318:82291253-82294574:82293246-82294574	298	149	0.922	0.7373	0.185	0.185	0	0
RI	chr5	Gigyf1	+	137518482-137518612:137518482-137518986:137518878-137518986	298	149	0.4707	0.2853	0.185	0.185	0.000847394	0.005458
SE	chr15	Senp1	-	98076550-98076709:98076912-98077042:98082243-98082327	279	149	0.0683	0.2537	-0.185	0.185	4.74713E-07	1.57E-05
SE	chr6	Eif2ak3	+	70878835-70878968:70881528-70881718:70882265-70882427	298	149	1	0.8153	0.185	0.185	2.24479E-08	1.01E-06
SE	chr15	Rai14	-	10595835-10595892:10599720-10599784:10610005-10610093	213	149	0.99	0.8053	0.185	0.185	5.11402E-09	2.69E-07
SE	chr2	Sdccag3	-	26388492-26388560:26388916-26389065:26389149-26389236	298	149	0.2533	0.4383	-0.185	0.185	0.004219496	0.040106
SE	chr3	Fnbp1l	-	122549156-122549265:122556412-122556570:122557903-122558106	298	149	0.3723	0.1877	0.185	0.185	0	0
SE	chr7	Zfp28	+	6390400-6390560:6392182-6392296:6393367-6395755	263	149	0.6233	0.4387	0.185	0.185	0.002577745	0.026569
SE	chr10	Ddx50	-	62646914-62647201:62647534-62647643:62651025-62651248	258	149	0.427	0.612	-0.185	0.185	8.66475E-06	0.000202
SE	chr1	Ivns1abp	+	151354634-151354759:151355167-151355311:151356129-151356236	293	149	0.227	0.4117	-0.185	0.185	0	0
SE	chr12	Lrfn5	+	61669480-61669654:61768008-61768077:61839408-61840812	218	149	0.1803	0.365	-0.185	0.185	1.46232E-07	5.45E-06
SE	chr2	Odf2	+	29892186-29892276:29893001-29893154:29893444-29893512	298	149	0.6733	0.4887	0.185	0.185	4.4752E-06	0.000113
SE	chr3	Adgrl2	-	148820973-148821025:148821280-148821408:148822955-148823051	277	149	0.979	0.7937	0.185	0.185	4.68919E-11	3.57E-09
A3SS	chr3	Dclk2	-	86792069-86792111:86786151-86789490:86786151-86787412	298	149	0.757	0.573	0.184	0.184	0.00107224	0.016119
A3SS	chr4	Usp48	+	137634898-137635008:137637760-137637890:137637765-137637890	153	149	0.7297	0.5453	0.184	0.184	2.95117E-05	0.000902
A3SS	chr5	Rsrc2	-	123742779-123742856:123740672-123740862:123740672-123740864	170	149	0.7207	0.5363	0.184	0.184	5.25985E-07	2.92E-05
A5SS	chr2	Polr1b	+	129113501-129113727:129113501-129113782:129115639-129115772	203	149	0.8207	0.6367	0.184	0.184	0.001186396	0.013293
A5SS	chr3	Dhx36	-	62484131-62484274:62483535-62484274:62480802-62480919	298	149	0.181	0.3647	-0.184	0.184	5.69544E-14	1.15E-11
MXE	chrX	Dlg3	+	100808053-100808152:100809862-100809907:100811502-100811543:100812	194	190	0.2937	0.11	0.184	0.184	0.000295712	0.005636
RI	chr2	Dlgap4	+	156748023-156748102:156748023-156748420:156748332-156748420	298	149	0.9287	0.7443	0.184	0.184	2.43677E-09	6.29E-08
SE	chr3	Tars2	-	95750701-95750765:95750867-95750984:95751506-95751630	266	149	0.9307	0.7463	0.184	0.184	1.34263E-05	0.000296
SE	chr1	Eif2d	+	131163520-131163565:131164403-131164506:131164620-131164769	252	149	0.9737	0.7897	0.184	0.184	1.59224E-10	1.09E-08
SE	chr11	Pemt	-	59974068-59974179:59976839-59976984:59983443-59983558	294	149	0.9637	0.7793	0.184	0.184	2.77292E-05	0.000558
SE	chr17	Nfya	-	48395650-48395796:48398985-48399065:48400438-48400580	229	149	0.79	0.6063	0.184	0.184	0.000244186	0.003588
SE	chr3	Slc22a15	-	101883460-101883589:101897110-101897274:101897937-101898069	298	149	1	0.8157	0.184	0.184	6.19602E-05	0.001107
SE	chr5	Dnajb6	+	29766325-29766395:29781130-29781450:29785031-29786478	298	149	0.9487	0.7643	0.184	0.184	7.751E-11	5.6E-09
SE	chr7	Ptpn5	-	47081863-47081973:47082761-47082898:47083086-47083165	286	149	0.979	0.795	0.184	0.184	0	0
SE	chr15	Hnrnpa1	+	103242644-103242718:103242922-103243080:103243959-103244028	298	149	0.9873	0.8037	0.184	0.184	0	0
SE	chr17	Wiz	-	32358943-32359494:32361461-32361964:32364182-32365057	298	149	0.9337	0.75	0.184	0.184	7.15147E-05	0.001252
SE	chr6	Ica1	-	8653497-8653535:8653630-8653692:8656362-8656408	211	149	0.9517	0.768	0.184	0.184	3.12663E-10	2.05E-08
SE	chr15	Eef1d	-	75896820-75896919:75897226-75897282:75901132-75901252	205	149	0.0923	0.2763	-0.184	0.184	0.000204487	0.003081
SE	chr9	Clpx	+	65310152-65310311:65312098-65312139:65316644-65316820	190	149	0.92	0.7357	0.184	0.184	1.05474E-07	4.05E-06
SE	chr9	Nktr	+	121727316-121727390:121728578-121728685:121729636-121729680	256	149	0.961	0.7773	0.184	0.184	1.33401E-07	5.01E-06
SE	chrX	Atp11c	-	60292319-60292383:60293607-60293657:60295189-60295292	199	149	1	0.8163	0.184	0.184	1.80589E-12	1.79E-10
A3SS	chr19	Cuedc2	-	46338569-46338660:46338313-46338420:46338313-46338411	157	149	0.427	0.2437	0.183	0.183	0.000563567	0.009569
A3SS	chr8	Zfp90	+	106419071-106419197:106419421-106419610:106419515-106419610	242	149	0.701	0.518	0.183	0.183	4.53083E-06	0.000189
A5SS	chr4	Cdk11b	+	155625531-155625654:155625531-155625773:155626774-155626889	267	149	0.406	0.5887	-0.183	0.183	1.46249E-06	5.31E-05
MXE	chr15	Kctd17	+	78435585-78435710:78436901-78436994:78437158-78437671:78438487-784	242	298	0.536	0.3533	0.183	0.183	6.33386E-08	4.16E-06
MXE	chr11	Trim37	+	87129716-87129817:87140485-87140601:87143127-87143214:87145464-871	265	236	0.2423	0.4257	-0.183	0.183	8.13145E-07	3.76E-05
MXE	chr2	Kyat1	-	30188215-30188364:30191925-30192072:30194054-30194112:30197957-301	207	296	0.2973	0.1143	0.183	0.183	0.001042705	0.015449
MXE	chr14	Appl1	-	26958453-26958511:26959425-26959466:26960951-26961038:26963647-269	236	190	0.2253	0.042	0.183	0.183	1.6903E-05	0.000501
SE	chr5	Tctn2	+	124598749-124598956:124599135-124599242:124599707-124599783	256	149	1	0.817	0.183	0.183	2.03999E-11	1.66E-09
SE	chr5	Ociad2	-	73322198-73323847:73326281-73326398:73327663-73327710	266	149	0.9073	0.7247	0.183	0.183	2.67565E-06	7.2E-05
SE	chr3	Dnajb4	-	152186415-152186983:152193377-152193618:152209835-152210083	298	149	0.8777	0.6947	0.183	0.183	0.000142019	0.002249
SE	chr5	Ttc28	+	110879784-110879988:110892832-110893110:111085022-111085169	298	149	0.7057	0.8887	-0.183	0.183	0.000678017	0.008603
SE	chr11	Zfp672	-	58319324-58319590:58322569-58322729:58323270-58323365	298	149	0.8227	0.6397	0.183	0.183	0.003145325	0.031348
SE	chr9	Map2k5	-	63216993-63217025:63217339-63217365:63235289-63235318	175	149	1	0.8173	0.183	0.183	3.44169E-15	5.22E-13
SE	chr9	Sik3	+	46202000-46202070:46204206-46204349:46207922-46208072	292	149	0.5523	0.735	-0.183	0.183	0.001935365	0.020764
SE	chr6	Adcyap1r1	+	55487659-55487742:55491230-55491310:55494077-55494206	229	149	0.9307	0.7473	0.183	0.183	2.67568E-05	0.000541
SE	chr5	G3bp2	-	92057968-92058070:92063337-92063435:92064325-92064505	247	149	0.4813	0.2987	0.183	0.183	9.11493E-14	1.11E-11
SE	chr4	Hnrnpr	+	136310942-136311089:136314090-136314255:136316361-136316479	298	149	0.7387	0.556	0.183	0.183	2.77067E-12	2.63E-10
SE	chr10	Cnot2	-	116501192-116501317:116504035-116504114:116517317-116517383	228	149	0.6493	0.832	-0.183	0.183	9.75287E-06	0.000224
SE	chr9	3110039I08Rik	+	41393194-41393299:41393378-41393417:41402124-41402263	188	149	0.1923	0.375	-0.183	0.183	0.003859782	0.03726
SE	chr12	Dtnb	+	3718412-3718489:3732618-3732707:3734939-3735026	238	149	0.3227	0.1397	0.183	0.183	3.83577E-06	9.84E-05
SE	chrX	Atp11c	-	60223283-60225793:60229148-60230013:60236557-60236710	298	149	0.9793	0.796	0.183	0.183	1.46966E-07	5.47E-06
MXE	chr6	Vopp1	-	57762435-57762571:57774711-57774788:57789977-57790035:57808536-578	207	226	0.082	0.2643	-0.182	0.182	0	0
MXE	chr9	Tcf12	-	71922652-71922787:72000416-72000518:72006701-72006762:72015638-720	210	251	0.1043	0.286	-0.182	0.182	5.05988E-06	0.000179
MXE	chr6	Ccdc136	+	29417932-29418153:29419102-29419383:29422037-29422097:29426625-294	298	209	0.0503	0.2327	-0.182	0.182	4.09901E-07	2.12E-05
MXE	chr5	Zfp644	-	106635610-106638635:106666785-106666845:106690730-106690877:106695	296	209	0.1087	0.291	-0.182	0.182	2.96887E-05	0.000817
RI	chr12	Dnajc27	+	4106802-4107348:4106802-4110611:4107550-4110611	298	149	0.9297	0.7477	0.182	0.182	2.61739E-06	3.7E-05
SE	chr16	Anks3	-	4941415-4941797:4941899-4941987:4942211-4942294	237	149	0.7307	0.549	0.182	0.182	0.002379178	0.02475
SE	chr7	Zfp180	+	24097821-24097895:24101453-24101568:24104405-24107709	264	149	0.864	0.6817	0.182	0.182	0.001026865	0.012213
SE	chr1	Usp40	-	87952340-87952458:87954012-87954051:87954151-87954245	188	149	0.2637	0.082	0.182	0.182	9.40837E-05	0.001584
SE	chr10	Tbc1d30	-	121279098-121279210:121288880-121288985:121310644-121311065	254	149	1	0.818	0.182	0.182	8.86847E-07	2.75E-05
SE	chrX	Pdzd4	-	73796376-73796477:73796830-73796892:73798194-73798292	211	149	0.9607	0.7787	0.182	0.182	0	0
SE	chr16	Dnm1l	-	16316602-16316778:16318764-16318841:16319452-16319508	226	149	0.96	0.7777	0.182	0.182	8.43769E-14	1.04E-11
SE	chr16	B4galt4	+	38757679-38757866:38763069-38763129:38765915-38766019	209	149	0.833	0.651	0.182	0.182	0.00217004	0.022888
SE	chr19	Csf2ra	-	61225324-61225652:61225792-61225821:61225928-61226055	178	149	0.8577	0.676	0.182	0.182	0.003385623	0.033385

SE	chr8	Dctd	+	48137258-48137374:48138782-48138878:48140343-48141667	245	149	0.994	0.8123	0.182	0.182	6.01442E-09	3.1E-07
SE	chr10	Tpd52l1	-	31338185-31338245:31341140-31341178:31343012-31343026	187	149	1	0.8183	0.182	0.182	8.29149E-09	4.15E-07
SE	chr1	Nfasc	-	132576286-132576417:132584364-132584480:132593351-132593365	265	149	0.0233	0.2053	-0.182	0.182	1.44178E-06	4.2E-05
SE	chr1	Bag2	-	33745484-33747016:33748217-33748326:33757579-33757750	258	149	0.744	0.562	0.182	0.182	0.001307496	0.014992
SE	chr3	Slc44a5	+	154265459-154265543:154267677-154268620:154269849-154270072	298	149	0.963	0.7813	0.182	0.182	1.64374E-05	0.000352
SE	chr2	Sec61a2	-	5876841-5877001:5877688-5878008:5882581-5882690	298	149	0.9627	0.7807	0.182	0.182	2.72957E-10	1.81E-08
SE	chr11	Synrg	+	84039211-84039321:84039656-84039691:84040797-84041455	184	149	0.1597	0.3413	-0.182	0.182	1.06527E-05	0.000242
SE	chr12	Papola	+	105807165-105807218:105808555-105808666:105809343-105809432	260	149	0.9723	0.7907	0.182	0.182	0	0
SE	chr19	Ankrd13d	-	4281918-4282042:4282169-4282304:4282881-4283137	284	149	0.9853	0.803	0.182	0.182	0	0
A3SS	chr11	Sec14l1	+	117156801-117156902:117157259-117159268:117158754-117159268	298	149	0.6323	0.4513	0.181	0.181	2.13118E-05	0.000709
A3SS	chr11	Zfp207	+	80402646-80402711:80403365-80408557:80403601-80408557	298	149	0.894	0.7133	0.181	0.181	9.47021E-07	4.88E-05
A3SS	chr16	Dcbld2	+	58451661-58451785:58455126-58455276:58455203-58455276	225	149	0.883	0.7017	0.181	0.181	0.003788134	0.041112
A3SS	chr2	Strbp	-	37580673-37580806:37577770-37579749:37577770-37579468	298	149	1	0.819	0.181	0.181	3.0972E-06	0.000134
A5SS	chr11	Zfp62	+	49213590-49213642:49213590-49214654:49215064-49218785	298	149	0.3493	0.1683	0.181	0.181	0.000122304	0.002102
A5SS	chr11	Snx11	-	96776968-96777061:96776904-96777061:96775374-96775433	212	149	0.3543	0.1733	0.181	0.181	0.001267006	0.013826
MXE	chr16	Anks3	-	4941415-4941797:4942693-4942778:4943079-4943251:4943559-4943700	298	234	0.975	0.7937	0.181	0.181	1.18372E-10	1.47E-08
MXE	chr9	Mto1	+	78457917-78458047:78460833-78461037:78461520-78461594:78464884-784	298	223	0.8977	0.7167	0.181	0.181	0.003217218	0.037385
MXE	chr16	Senp5	-	31965745-31965828:31968800-31968937:31977680-31977757:31983264-319	226	286	0.335	0.5163	-0.181	0.181	0.003894465	0.043213
RI	chr2	Gm30189	-	174320908-174322197:174320908-174323965:174323664-174323965	298	149	0.3983	0.217	0.181	0.181	0.011808502	0.049843
SE	chr17	Rhbdl1	-	25835969-25836193:25836411-25836572:25836988-25837127	298	149	0.435	0.2543	0.181	0.181	5.88314E-07	1.91E-05
SE	chr11	Rpain	+	70974069-70974132:70974900-70975040:70977658-70977687	289	149	0.957	0.7763	0.181	0.181	7.16241E-06	0.000171
SE	chr2	Mbd5	+	49018254-49018325:49086590-49086652:49099717-49099809	211	149	0.75	0.9307	-0.181	0.181	0.000652756	0.008339
SE	chr12	Ncoa1	-	4247362-4249750:4249836-4249890:4253657-4253746	203	149	0.547	0.7283	-0.181	0.181	2.18517E-05	0.000452
SE	chr17	Zfp945	-	22861506-22861561:22861732-22861852:22865247-22865336	269	149	0.3747	0.194	0.181	0.181	0.000135178	0.002155
SE	chr18	Polr2d	+	31791840-31792020:31795288-31795383:31796050-31796642	244	149	0.8973	0.716	0.181	0.181	0	0
SE	chr19	Bbs1	-	4892888-4893021:4894261-4894419:4894962-4895031	298	149	0.9717	0.7903	0.181	0.181	2.65475E-07	9.28E-06
SE	chr9	Clasp2	+	113876244-113876350:113878750-113878773:113879942-113880186	172	149	0.934	0.753	0.181	0.181	0	0
SE	chr11	Map2k4	-	65756269-65756371:65775733-65775765:65788136-65788313	181	149	0.583	0.7643	-0.181	0.181	9.16227E-06	0.000212
SE	chr2	L3mbtl1	+	162966986-162967085:162967252-162967449:162967557-162967606	298	149	1	0.8193	0.181	0.181	0.000110691	0.001821
SE	chr10	Fbxo5	-	5799158-5799822:5800256-5800435:5801152-5801242	298	149	0.981	0.8003	0.181	0.181	1.93922E-07	7.03E-06
SE	chr10	Prdm4	-	85902927-85903012:85904131-85904270:85905349-85905498	288	149	0.9817	0.8007	0.181	0.181	1.32549E-07	4.98E-06
SE	chr5	Srpk2	-	23525496-23525745:23526790-23526815:23527897-23528062	174	149	1	0.8193	0.181	0.181	0	0
SE	chr4	1110017D15Rik	-	41505568-41505654:41507099-41507328:41507521-41507593	298	149	0.234	0.4147	-0.181	0.181	0.001940993	0.020814
SE	chr14	Slc4a7	+	14760235-14760341:14762348-14762522:14765577-14765710	298	149	0.845	0.664	0.181	0.181	0.001329972	0.015213
A3SS	chrX	Syn1	-	20862499-20863090:20860511-20861609:20860511-20861571	186	149	0.5217	0.3417	0.18	0.18	2.40548E-07	1.54E-05
A3SS	chr9	Tmed1	-	21509994-21510234:21508931-21509351:21508931-21509114	298	149	0.9653	0.785	0.18	0.18	1.46041E-06	6.89E-05
A5SS	chr2	St6galnac6	+	32608042-32608096:32608042-32608225:32609226-32609316	277	149	0.4067	0.227	0.18	0.18	0.005225221	0.041284
MXE	chr8	Eps15l1	-	72340990-72341524:72345966-72346171:72358313-72358445:72367904-723	281	298	0.4603	0.6403	-0.18	0.18	2.67743E-07	1.49E-05
MXE	chr11	B9d1	+	61506344-61506412:61507628-61507739:61509470-61509532:61512372-615	260	211	0.514	0.3343	0.18	0.18	0.000176388	0.003644
SE	chr8	Zdhhc1	-	105472991-105473069:105473206-105473299:105473484-105473566	242	149	0.893	0.7127	0.18	0.18	0.000168159	0.002604
SE	chr7	Ntrk3	-	78300690-78302747:78304292-78304426:78356027-78356215	283	149	0.973	0.7933	0.18	0.18	7.45122E-10	4.56E-08
SE	chr11	Trim37	+	87129716-87129817:87137590-87137630:87140485-87140601	189	149	0.87	0.6897	0.18	0.18	7.20669E-11	5.25E-09
SE	chr6	Ppfibp1	+	146888080-146888683:146917748-146917908:146938906-146938989	298	149	0.1477	0.328	-0.18	0.18	0.001174931	0.013683
SE	chr19	Shtn1	-	58990189-58990381:58995018-58995138:58999893-58999946	269	149	0.9887	0.8087	0.18	0.18	4.64747E-09	2.48E-07
SE	chr5	Tmem165	+	76204637-76204816:76207802-76207907:76208510-76209244	254	149	0.995	0.815	0.18	0.18	0	0
SE	chr11	Acsf2	-	94569941-94570032:94570303-94570460:94570614-94570709	298	149	0.985	0.8047	0.18	0.18	6.9591E-05	0.001222
SE	chr14	Mycbp2	-	103262702-103262821:103275789-103275890:103276373-103276518	250	149	0.984	0.804	0.18	0.18	4.97047E-13	5.37E-11
SE	chr1	Syt14	-	192933114-192933385:192940986-192941110:192980346-192980428	273	149	0.2613	0.4417	-0.18	0.18	0.004879504	0.045231
SE	chr7	Lsm14a	-	34344637-34345958:34347926-34347982:34351286-34351517	205	149	0.5017	0.3213	0.18	0.18	4.34592E-09	2.34E-07
SE	chr12	Trim9	-	70252443-70252484:70255038-70255226:70267234-70267263	298	149	0.8917	0.712	0.18	0.18	2.75171E-05	0.000554
SE	chr11	Fbf1	-	116164663-116165912:116167133-116167228:116167552-116168178	244	149	0.7057	0.886	-0.18	0.18	4.2764E-05	0.000806
A5SS	chr8	Pnpla6	+	3537767-3537883:3537767-3538085:3541291-3541592	298	149	0.9697	0.7903	0.179	0.179	2.50135E-07	1.23E-05
A5SS	chr9	Cep70	+	99277937-99278018:99277937-99278157:99281028-99281102	287	149	1	0.8213	0.179	0.179	7.41905E-07	2.99E-05
RI	chr18	Poli	-	70525344-70525528:70525344-70526721:70526557-70526721	298	149	0.3003	0.1213	0.179	0.179	0.000498294	0.003455
RI	chr18	Slc35a4	+	36681528-36681765:36681528-36683862:36681874-36683862	256	149	0.5773	0.3987	0.179	0.179	0.000830014	0.005382
SE	chr7	2310010J17Rik	-	90124836-90125032:90126928-90127044:90129872-90129936	265	149	0.0447	0.2237	-0.179	0.179	7.96139E-05	0.001372
SE	chr3	Clk2-scamp3	+	89168695-89168923:89169615-89169702:89170049-89170115	236	149	0.8133	0.634	0.179	0.179	0.000152167	0.002387
SE	chr6	Ptcd3	-	71889594-71889686:71892469-71892519:71893382-71893526	199	149	0.9897	0.8103	0.179	0.179	5.55112E-16	9.03E-14
SE	chr17	Slc25a27	-	43661614-43661698:43664074-43664265:43666900-43667015	298	149	0.9793	0.8007	0.179	0.179	7.28306E-14	9.03E-12
SE	chr11	Srcin1	-	97537044-97537239:97542534-97542557:97551677-97551978	172	149	0.4513	0.63	-0.179	0.179	8.09536E-05	0.001393
SE	chr16	Ttc3	+	94403314-94403368:94412060-94412123:94415949-94416009	212	149	0.5917	0.7707	-0.179	0.179	7.54952E-15	1.11E-12
SE	chr10	Mical1	+	41479326-41479499:41479619-41479774:41480285-41480385	298	149	0.9903	0.8113	0.179	0.179	3.18381E-11	2.5E-09
SE	chr11	Socs7	+	97376437-97376541:97376997-97377098:97377936-97378066	250	149	0.9757	0.7963	0.179	0.179	1.74749E-13	2.05E-11
SE	chr7	Shank2	+	144405330-144405460:144407121-144407205:144408056-144408076	233	149	0.9203	0.7417	0.179	0.179	3.12568E-05	0.000618
SE	chr12	Vipas39	-	87253166-87253258:87254230-87254286:87256485-87256549	205	149	0.9977	0.819	0.179	0.179	0	0
SE	chrX	Upf3b	-	37096858-37097018:37099066-37099104:37099507-37099689	187	149	0.4033	0.224	0.179	0.179	7.92797E-06	0.000187
SE	chr2	Edem2	-	155713338-155713479:155716010-155716221:155718264-155718389	298	149	0.9427	0.764	0.179	0.179	1.84286E-07	6.71E-06
SE	chr17	Ptprs	-	56427939-56428132:56428852-56429157:56434453-56434597	298	149	0.988	0.809	0.179	0.179	0	0
SE	chr7	Lyrm1	+	119896137-119896252:119909671-119909962:119914173-119914265	298	149	0.8073	0.9867	-0.179	0.179	6.66154E-07	2.14E-05
A3SS	chr15	Hdac10	-	89124532-89124588:89124175-89124428:89124175-89124256	298	149	0.8973	0.7197	0.178	0.178	0.001057169	0.015926
A3SS	chr9	Elmod1	-	53946848-53946949:53935730-53935913:53935730-53935875	186	149	0.1213	0.299	-0.178	0.178	3.34033E-12	6.34E-10
A5SS	chr5	Gm1673	+	33983433-33983564:33983433-33983639:33984052-33984140	223	149	0.6347	0.8127	-0.178	0.178	0.000269593	0.004
A5SS	chr4	Tardbp	-	148618665-148618791:148618647-148618791:148612382-148617705	166	149	0.8393	0.6613	0.178	0.178	2.09776E-05	0.000482
A5SS	chr4	Tex10	-	48468754-48468993:48468281-48468993:48467664-48467904	298	149	0.9237	0.7457	0.178	0.178	4.68452E-07	2.01E-05
MXE	chr3	Fnbp1l	-	122549156-122549265:122552136-122552150:122556412-122556570:122557	298	163	0.8847	0.7063	0.178	0.178	6.66134E-16	2.31E-13
MXE	chr11	Slc38a10	-	120140593-120140686:120141600-120141645:120147721-120147838:12015	266	194	0.236	0.4143	-0.178	0.178	3.81379E-07	2.01E-05
MXE	chr12	Atxn7l1	+	33147598-33147915:33148433-33148501:33217712-33217816:33325911-333	217	253	0.6507	0.829	-0.178	0.178	0.000590781	0.009876
MXE	chr2	Rapgef4	+	72137242-72137314:72141153-72141172:72174423-72174476:72174802-721	168	202	0.6933	0.8717	-0.178	0.178	0.004045132	0.044337
RI	chr16	Ece2	+	20644068-20644258:20644068-20644436:20644341-20644436	230	149	0.527	0.3487	0.178	0.178	0.007962054	0.035461
SE	chr15	Kctd17	+	78435585-78435710:78437158-78437671:78438487-78439303	298	149	0.364	0.5423	-0.178	0.178	7.44929E-05	0.001296
SE	chr2	Mtg2	+	180082086-180082233:180084451-180084589:180085380-180085902	287	149	0.9593	0.781	0.178	0.178	0.000130554	0.00209
SE	chr13	Pfkp	-	6588489-6588653:6597859-6598011:6598739-6598826	298	149	0.852	0.6743	0.178	0.178	4.98683E-05	0.000922
SE	chr19	Syt7	+	10417963-10418042:10422785-10423012:10435550-10435685	298	149	0.1803	0.3583	-0.178	0.178	0.004394094	0.041443
SE	chr5	Fbrsl1	-	110379269-110379593:110381601-110381646:110396045-110396074	194	149	0.9523	0.7747	0.178	0.178	1.19292E-09	7.02E-08
SE	chr3	Dnajb4	-	152186415-152186983:152193377-152193664:152209835-152210328	298	149	0.8847	0.707	0.178	0.178	9.80888E-05	0.00164
SE	chr2	Ptprt	-	161697974-161698109:161718355-161718411:161732879-161732915	205	149	0.064	0.2423	-0.178	0.178	5.40357E-05	0.000988
SE	chr11	4933427D14Rik	-	72159400-72159520:72165517-72165598:72166638-72166823	230	149	0.8327	0.6547	0.178	0.178	0.00024611	0.003613
A3SS	chr6	Tra2a	-	49263838-49264248:49252382-49253108:49252382-49252515	298	149	0.422	0.245	0.177	0.177	6.06263E-06	0.000243
A5SS	chr7	Zfp61	-	24295979-24296024:24295710-24296024:24294955-24295081	298	149	0.284	0.1073	0.177	0.177	4.00534E-06	0.000123
MXE	chrX	Cnksr2	-	157906670-157906881:157910117-157910250:157919746-157919892:15793	295	282	0.183	0.3597	-0.177	0.177	7.96924E-07	3.69E-05
RI	chr1	Kdm5b	+	134630433-134630753:134630433-134631950:134631237-134631950	298	149	0.4307	0.254	0.177	0.177	2.04679E-09	5.45E-08
SE	chr8	Nek1	+	61044060-61044107:61044259-61044333:61049784-61049947	223	149	0.9747	0.798	0.177	0.177	1.36937E-06	4.02E-05
SE	chr11	Flot2	+	78053363-78053453:78054785-78054875:78055757-78055880	239	149	0.7613	0.5847	0.177	0.177	6.4872E-05	0.001149
SE	chr11	Fbxw11	+	32642003-32642935:32652748-32652904:32680201-32680302	298	149	0.2113	0.388	-0.177	0.177	4.51069E-05	0.000842
SE	chrX	Armcx2	-	134807262-134807327:134807454-134807513:134807621-134807725	208	149	0.8957	0.7183	0.177	0.177	4.26548E-13	4.67E-11
SE	chr9	C2cd2l	-	44313878-44314014:44314155-44314256:44314880-44315054	250	149	0.9857	0.8083	0.177	0.177	9.01063E-10	5.42E-08
SE	chr2	Ryr3	-	112678377-112678547:112681735-112681749:112686575-112686667	163	149	0.987	0.8103	0.177	0.177	1.81072E-06	5.14E-05
SE	chr1	Rgs7	-	175059076-175059794:175066617-175066694:175078189-175078278	226	149	0.487	0.6637	-0.177	0.177	0.0010655	0.012591
MXE	chr2	Mtg2	+	180082086-180082233:180083345-180083460:180084451-180084589:18008	264	287	0.3697	0.5453	-0.176	0.176	0.00123982	0.017866
MXE	chr12	Mta1	+	113133185-113133337:113133496-113133576:113134801-113134812:11313	229	160	0.3493	0.173	0.176	0.176	0.001122749	0.016475
MXE	chr2	Bbs5	+	69654063-69654112:69655499-69655634:69656897-69656992:69661036-696	284	244	0.075	0.251	-0.176	0.176	0.000218739	0.004377
RI	chr3	Fxr1	+	34058024-34058081:34058024-34058231:34058169-34058231	235	149	0.3253	0.5013	-0.176	0.176	1.01179E-08	2.39E-07
RI	chr10	Nap1l1	+	111496040-111496105:111496040-111498150:111496754-111498150	298	149	0.7423	0.566	0.176	0.176	1.62093E-14	9.66E-13
RI	chr8	Trmt1	+	84690517-84690572:84690517-84690947:84690811-84690947	298	149	0.503	0.3273	0.176	0.176	3.3173E-05	0.000333
SE	chr16	Fopnl	-	14304454-14304587:14311047-14311131:14317294-14317400	233	149	0.249	0.425	-0.176	0.176	3.48717E-07	1.19E-05
SE	chr5	Adam22	-	8092731-8092831:8107002-8107019:8111895-8111985	166	149	0.9433	0.7673	0.176	0.176	3.33371E-05	0.000652
SE	chr19	Ssh3	-	4264314-4264460:4265103-4265149:4265292-4265420	195	149	1	0.824	0.176	0.176	3.25775E-09	1.79E-07
SE	chr5	Epha5	-	84156412-84156536:84233583-84233918:84331228-84331891	298	149	0.451	0.6267	-0.176	0.176	0.002978027	0.029933
SE	chr16	Dnm1l	-	16316602-16316778:16318445-16318477:16318764-16318841	181	149	0.945	0.7687	0.176	0.176	3.33067E-16	5.56E-14
SE	chr9	Dnajc13	-	104151597-104152423:104153268-104153376:104156814-104156913	257	149	0.6703	0.4947	0.176	0.176	0.001520932	0.01698
SE	chr15	Prpf40b	+	99295087-99295273:99303283-99303363:99304041-99304184	229	149	0.976	0.8003	0.176	0.176	6.67122E-12	5.91E-10
SE	chr6	Tnpo3	-	29589029-29589229:29599540-29599698:29609366-29609550	298	149	0.127	0.303	-0.176	0.176	2.33147E-15	3.57E-13
SE	chr1	Pfkfb2	-	130689043-130691148:130697017-130698085:130698679-130698743	298	149	0.8683	0.6923	0.176	0.176	6.01224E-06	0.000147
SE	chr10	Agpat3	-	78284189-78284350:78284995-78285164:78287847-78288073	298	149	0.98	0.804	0.176	0.176	0	0
SE	chr17	Slc9a3r2	-	24639281-24640389:24640595-24640657:24641709-24641752	211	149	0.717	0.8927	-0.176	0.176	5.94331E-07	1.93E-05
SE	chr11	Slc22a5	-	53873668-53873794:53874935-53875106:53891147-53891595	298	149	0.967	0.7913	0.176	0.176	1.44644E-05	0.000315
SE	chr7	Osbpl5	-	143700714-143700810:143701157-143701290:143702690-143702771	282	149	0.9267	0.7503	0.176	0.176	0.000154087	0.002412
A3SS	chr2	Dlgap4	+	156745835-156746196:156748023-156748420:156748332-156748420	298	149	0.4503	0.2753	0.175	0.175	3.24551E-08	2.86E-06
A3SS	chr2	Nr4a2	-	57109063-57109265:57108561-57108739:57108561-57108618	269	149	0.85	0.6747	0.175	0.175	3.72718E-05	0.001094
A3SS	chr12	Kidins220	+	25053798-25054034:25056582-25061278:25061218-25061278	298	149	0.9777	0.8027	0.175	0.175	0	0
A3SS	chr10	Pip5k1c	+	81314992-81315118:81315879-81317403:81317326-81317403	298	149	0.2883	0.463	-0.175	0.175	2.39198E-12	4.79E-10
A3SS	chr8	C230057M02Rik	-	122752114-122752649:122729771-122738893:122729771-122729894	298	149	0.1497	0.325	-0.175	0.175	3.47678E-05	0.001028
A3SS	chr6	Tia1	+	86420324-86420411:86423606-86424420:86424345-86424420	298	149	0.3743	0.199	0.175	0.175	4.80918E-11	7.38E-09
A3SS	chr12	Trim9	-	70268062-70268200:70267234-70267532:70267234-70267263	298	149	0.7753	0.6007	0.175	0.175	0.001588472	0.021586

A3SS	chr1	Edem3	+	151811519-151811704:151812754-151812768:151812757-151812768	151	149	0.8437	0.6687	0.175	0.175	0.004231363	0.044705
MXE	chr11	Ogdh	+	6313788-6313979:6316765-6316855:6316978-6317080:6324878-6324922	239	251	0.6627	0.838	-0.175	0.175	6.90148E-07	3.31E-05
MXE	chr3	Dclk2	-	86791995-86792111:86793817-86793913:86799011-86799088:86799461-867	226	245	0.9133	0.738	0.175	0.175	2.22045E-16	7.87E-14
MXE	chr15	Cacnb3	+	98640339-98640454:98640689-98640750:98640959-98640978:98641177-986	210	168	0.1723	0.3473	-0.175	0.175	0.000331533	0.006202
MXE	chr1	Hjurp	-	88269321-88269413:88270202-88270299:88272729-88272765:88275042-882	185	246	0.096	0.271	-0.175	0.175	0.001287391	0.018326
RI	chr6	Luc7l2	+	38557852-38558075:38557852-38559304:38559204-38559304	298	149	0.521	0.3463	0.175	0.175	4.83969E-06	6.21E-05
SE	chrX	Nlgn3	+	101301785-101302431:101307075-101307134:101308757-101308906	208	149	0.623	0.448	0.175	0.175	0.001868513	0.020145
SE	chr12	Nrcam	+	44576604-44576780:44584835-44584987:44590108-44590239	298	149	0.295	0.12	0.175	0.175	0.000120049	0.001946
SE	chr8	Sugp2	+	70260469-70260589:70261231-70261317:70262871-70263105	235	149	0.242	0.417	-0.175	0.175	8.37066E-06	0.000196
SE	chrX	Rpgr	-	10158216-10158880:10162602-10162690:10164722-10164779	237	149	0.8793	0.704	0.175	0.175	9.12404E-05	0.001544
SE	chr11	Unk	+	116056191-116056379:116058327-116058424:116058989-116059246	246	149	0.92	0.7447	0.175	0.175	0.000105347	0.001745
SE	chr16	Dzip3	-	48943457-48943552:48944763-48944928:48945598-48945623	298	149	0.9603	0.7853	0.175	0.175	2.07112E-12	2.03E-10
SE	chr10	Tab2	-	7924685-7924872:7933068-7933208:7955801-7956653	289	149	0.0613	0.2367	-0.175	0.175	9.21485E-15	1.31E-12
SE	chr15	Azin1	-	38501404-38501600:38507187-38507346:38514473-38514609	298	149	0.1563	0.331	-0.175	0.175	0	0
SE	chr6	Itpr1	+	108363610-108363705:108365299-108365343:108369011-108369165	193	149	0.9447	0.77	0.175	0.175	2.73523E-05	0.000551
SE	chr4	Ctnnal1	-	56817247-56817297:56822518-56822555:56826274-56826424	186	149	1	0.8247	0.175	0.175	2.22545E-06	6.13E-05
SE	chr13	Mtr	-	12190204-12190404:12193614-12193810:12195238-12195393	298	149	0.9857	0.811	0.175	0.175	3.27272E-05	0.000642
SE	chrX	Las1l	-	95946867-95947073:95947398-95947448:95947770-95947855	199	149	0.4477	0.2727	0.175	0.175	5.83713E-05	0.001056
SE	chr5	Add1	+	34628415-34628513:34629136-34629172:34630470-34631885	185	149	0.8767	0.7017	0.175	0.175	0	0
SE	chr8	Vps9d1	-	123245114-123245210:123245507-123245712:123245944-123246069	298	149	0.976	0.801	0.175	0.175	1.09912E-14	1.55E-12
SE	chr17	Srf	-	46549436-46549512:46549845-46550036:46550901-46551020	298	149	0.7227	0.5477	0.175	0.175	0.000725212	0.009118
A3SS	chr19	Nhlrc2	+	56591576-56591807:56592393-56592515:56592427-56592515	182	149	0.8567	0.6823	0.174	0.174	0.001166302	0.017106
A3SS	chr10	Kcnc2	+	112458490-112458654:112463117-112466304:112463991-112466304	298	149	1	0.826	0.174	0.174	1.12412E-06	5.56E-05
A5SS	chr9	Tmem205	-	21927292-21927535:21927287-21927535:21926249-21926357	153	149	0.1073	0.281	-0.174	0.174	1.43674E-06	5.26E-05
A5SS	chr11	Ghdc	-	100769398-100769529:100769028-100769529:100768385-100768577	298	149	0.9443	0.77	0.174	0.174	0.001135929	0.012887
MXE	chr15	Kctd17	+	78435585-78435710:78436901-78437099:78437158-78437671:78438487-784	298	298	0.496	0.3223	0.174	0.174	2.7587E-07	1.52E-05
MXE	chr4	Ube2j2	+	155955234-155955336:155955422-155955560:155956368-155956448:155957	287	229	0.5807	0.4067	0.174	0.174	8.22946E-07	3.78E-05
MXE	chr5	Ccdc92	-	124834432-124836291:124838943-124838984:124839661-124839807:124862	295	190	0.8503	0.6763	0.174	0.174	0.003631948	0.041047
MXE	chr9	Map4	+	109978850-109979091:109999772-109999840:110026101-110026223:110027	217	271	0.624	0.798	-0.174	0.174	1.9551E-13	4.45E-11
RI	chr8	Sugp2	+	70260469-70260589:70260469-70261317:70261231-70261317	298	149	0.8087	0.6347	0.174	0.174	0.000144975	0.001178
RI	chr11	Tbrg4	-	6615598-6615766:6615598-6616714:6616558-6616714	298	149	0.8447	0.6707	0.174	0.174	4.94847E-06	6.32E-05
SE	chr3	Rrnad1	-	87927055-87927117:87927563-87927755:87927924-87928048	298	149	0.964	0.7903	0.174	0.174	6.08086E-08	2.47E-06
SE	chr4	Ptprd	-	76050327-76050439:76054589-76054600:76059669-76059826	160	149	0.4417	0.268	0.174	0.174	3.88127E-05	0.00074
SE	chr11	Zfp672	-	58319324-58319590:58322600-58322729:58323270-58323365	278	149	0.82	0.6463	0.174	0.174	0.00465564	0.043525
SE	chr7	Peg3	-	6712673-6712775:6712950-6713012:6716054-6716157	211	149	0.2817	0.1073	0.174	0.174	2.46315E-06	6.71E-05
SE	chr9	Tcf12	-	72000416-72000518:72006701-72006762:72015638-72015711	210	149	0.1963	0.3707	-0.174	0.174	4.3466E-08	1.83E-06
SE	chr16	Mina	+	59487428-59487599:59489345-59489433:59491205-59491294	237	149	0.898	0.7237	0.174	0.174	0.002658415	0.027243
SE	chr9	Map4	+	110068693-110068985:110071026-110071139:110072613-110072705	262	149	0.179	0.3527	-0.174	0.174	1.69414E-08	7.77E-07
SE	chr4	Fbxo44	-	148156146-148156281:148156395-148156490:148156609-148156706	244	149	0.225	0.3987	-0.174	0.174	6.45639E-07	2.08E-05
SE	chr11	Clint1	+	45895060-45895129:45902211-45902285:45906181-45906467	223	149	0.9343	0.76	0.174	0.174	7.26533E-10	4.47E-08
SE	chr8	Wdr17	-	54643114-54643225:54645338-54645358:54648220-54648359	169	149	0.2647	0.0903	0.174	0.174	2.43533E-06	6.63E-05
SE	chr7	Lyrm1	+	119896138-119896252:119909804-119909962:119914173-119914265	298	149	0.8127	0.987	-0.174	0.174	9.20541E-07	2.84E-05
A3SS	chr11	Hdac5	-	102224734-102224955:102218416-102218953:102218416-102218487	298	149	0.1723	0.345	-0.173	0.173	1.3158E-07	9.31E-06
A3SS	chr8	Atp13a1	+	69799321-69799478:69799762-69799937:69799832-69799937	218	149	0.6007	0.4273	0.173	0.173	1.7234E-06	7.87E-05
A5SS	chr18	Slc12a2	+	57934951-57934998:57934951-57935491:57936308-57936430	298	149	0.2407	0.0673	0.173	0.173	7.37943E-09	5.39E-07
A5SS	chrX	Atrx	-	105929100-105929406:105928959-105929406:105899881-105899993	289	149	0.711	0.5383	0.173	0.173	0.000271059	0.004002
MXE	chr2	Fbxw2	-	34808514-34808600:34812815-34813009:34822558-34822975:34825962-348	298	298	0.9357	0.7627	0.173	0.173	1.34102E-07	7.99E-06
MXE	chr7	Fam168a	+	100812920-100813000:100824132-100824257:100831328-100831470:10083	274	291	0.2573	0.4303	-0.173	0.173	2.26887E-08	1.66E-06
MXE	chr9	Man2c1	+	57139246-57139401:57139611-57139708:57139870-57139964:57140382-571	246	243	0.896	0.723	0.173	0.173	3.96942E-05	0.001045
RI	chrX	Gpm6b	+	166385391-166385481:166385391-166389033:166386952-166389033	298	149	0.864	0.6907	0.173	0.173	6.66134E-16	4.39E-14
RI	chr14	Ndst2	-	20725574-20725697:20725574-20727090:20726994-20727090	298	149	0.3403	0.1673	0.173	0.173	0.000221447	0.00169
SE	chr17	Pot1b	-	55692722-55692877:55695027-55695317:55698457-55698587	298	149	0.9533	0.7803	0.173	0.173	0.000861022	0.010534
SE	chr12	Jag2	-	112915471-112915517:112915605-112915718:112915880-112915993	262	149	0.8797	0.7063	0.173	0.173	5.31045E-05	0.000973
SE	chr8	Adgrl1	+	83928299-83928382:83928668-83928891:83929464-83930264	298	149	0.0997	0.2727	-0.173	0.173	6.40215E-08	2.58E-06
SE	chr7	Zfand6	-	84634239-84634409:84652882-84653042:84679310-84679351	298	149	0.1683	0.3413	-0.173	0.173	2.0228E-10	1.36E-08
SE	chr2	Cacna1b	-	24678876-24679643:24682976-24683038:24685768-24685874	211	149	0.0637	0.2363	-0.173	0.173	4.79489E-07	1.58E-05
SE	chr2	Sh3glb2	-	30347081-30347104:30348555-30348617:30350288-30350380	211	149	0.752	0.925	-0.173	0.173	4.72164E-10	3.01E-08
SE	chr6	Tsen2	+	115547822-115548030:115549913-115549994:115553991-115554027	230	149	0.783	0.6097	0.173	0.173	5.51117E-05	0.001005
SE	chr11	Ccdc157	-	4147700-4147777:4148439-4149084:4160159-4160293	298	149	0.8273	1	-0.173	0.173	0.001154487	0.013486
SE	chr2	Odf2	+	29892186-29892276:29893038-29893154:29893444-29893512	265	149	0.5163	0.343	0.173	0.173	0.000117492	0.001909
SE	chr7	Dlg2	+	92417224-92417323:92418088-92418133:92420632-92420673	194	149	0.4537	0.6267	-0.173	0.173	0.000285931	0.004107
SE	chr18	Ss18	-	14636496-14636618:14637392-14637484:14640257-14640361	241	149	0.347	0.174	0.173	0.173	0.001644357	0.018107
SE	chr8	Coprs	-	13884788-13885201:13885666-13885881:13887760-13887826	298	149	0.9643	0.791	0.173	0.173	0	0
A3SS	chr2	Dph7	+	24963456-24963543:24965557-24965654:24965560-24965654	151	149	0.408	0.5803	-0.172	0.172	0.004463211	0.046609
A5SS	chr5	Trafd1	-	121373339-121373474:121373138-121373474:121372603-121372675	298	149	0.9223	0.7503	0.172	0.172	0	0
A5SS	chr2	Nrsn2	-	152374223-152374290:152374096-152374290:152368755-152369923	275	149	0.9087	0.7367	0.172	0.172	3.69116E-06	0.000115
MXE	chr15	Kctd17	+	78435585-78435710:78436901-78436994:78437158-78437232:78437584-784	242	223	0.8287	0.6563	0.172	0.172	3.14435E-05	0.00086
MXE	chr13	Fam169a	+	97091863-97091952:97094062-97094147:97097540-97097711:97106954-971	234	298	0.053	0.225	-0.172	0.172	6.64402E-07	3.25E-05
MXE	chr16	Dlg1	+	31842770-31842839:31846834-31846933:31847636-31847669:31853843-318	248	182	0.2913	0.1197	0.172	0.172	1.73132E-05	0.000511
MXE	chr19	Nrxn2	+	6509778-6509949:6513716-6513805:6516923-6517248:6519249-6519327	238	298	0.203	0.3753	-0.172	0.172	3.10862E-15	9.67E-13
MXE	chr17	Gltscr1l	-	46807396-46807490:46808349-46808494:46809568-46809626:46811924-468	207	294	0.263	0.4347	-0.172	0.172	0.001867404	0.024501
RI	chr17	Haghl	-	25784075-25784178:25784075-25784368:25784260-25784368	229	149	0.267	0.0953	0.172	0.172	0	0
SE	chr2	Zmynd8	-	165852000-165852148:165852868-165852879:165875705-165875775	160	149	0.681	0.5093	0.172	0.172	0.000367112	0.005071
SE	chr15	Pphln1	+	93410692-93410783:93420284-93420490:93424014-93424075	298	149	0.075	0.2467	-0.172	0.172	1.70075E-12	1.7E-10
SE	chr1	Fam20b	-	156705668-156706167:156716888-156716980:156718866-156719027	241	149	0.3957	0.5673	-0.172	0.172	0.000275008	0.003972
SE	chr16	Grik1	-	87950028-87950141:87955289-87955333:87957490-87957597	193	149	0.3367	0.165	0.172	0.172	0.000195546	0.002964
SE	chr10	Polrmt	-	79738031-79738210:79738328-79738450:79738682-79738804	271	149	0.9463	0.7747	0.172	0.172	0.000186639	0.002851
SE	chr5	Auts2	-	131466032-131466127:131470414-131470458:131472259-131472479	193	149	0.9447	0.7727	0.172	0.172	2.82199E-11	2.24E-09
SE	chr9	Zfp560	-	20345136-20349159:20349581-20349663:20350532-20350612	231	149	0.9437	0.772	0.172	0.172	0.000779849	0.009679
SE	chr11	Exoc7	-	116295544-116295689:116297571-116297663:116300266-116300433	241	149	0.8443	0.6727	0.172	0.172	5.79783E-09	3.01E-07
SE	chr1	Rgs8	+	153665836-153665916:153667825-153667953:153690898-153691064	277	149	0.785	0.6133	0.172	0.172	0.00487904	0.045231
SE	chr6	Pcyox1	-	86392137-86392348:86394409-86394583:86396973-86397161	298	149	0.951	0.779	0.172	0.172	1.07625E-12	1.11E-10
SE	chr6	Pcyox1	-	86392137-86392348:86395914-86396120:86396973-86397150	298	149	0.9527	0.781	0.172	0.172	7.06213E-13	7.43E-11
SE	chr11	Med7	+	46436937-46437191:46439894-46439975:46440563-46441608	230	149	0.4023	0.574	-0.172	0.172	0.002199149	0.023136
A3SS	chr17	Srrm2	+	23815122-23821690:23821921-23822700:23822413-23822700	298	149	0.3247	0.154	0.171	0.171	8.43769E-15	2.44E-12
A3SS	chr13	Eci2	-	35027009-35027090:34993068-34993328:34993068-34993230	246	149	0.8147	0.986	-0.171	0.171	1.52545E-05	0.000527
MXE	chr6	Ctnna2	-	76881637-76882837:76915734-76915868:76919880-76919985:76932346-769	254	283	0.8483	0.677	0.171	0.171	1.22458E-13	2.92E-11
MXE	chr16	Anks3	-	4942693-4942778:4943559-4943700:4946055-4946179:4947265-4947432	273	290	0.9777	0.807	0.171	0.171	8.25517E-12	1.4E-09
MXE	chr4	Tmem222	-	133268409-133268505:133269778-133269809:133270963-133271047:133277	233	180	0.585	0.4137	0.171	0.171	4.47623E-07	2.3E-05
MXE	chr15	Poldip3	-	83133147-83133326:83135294-83135389:83137468-83137554:83138132-831	235	244	0.4587	0.2877	0.171	0.171	1.23892E-07	7.5E-06
MXE	chr11	Ccdc85a	-	28392854-28392952:28396046-28396165:28434081-28434157:28576391-285	225	268	0.7177	0.8887	-0.171	0.171	0.000902126	0.013865
MXE	chr16	Itsn1	+	91837061-91837188:91839616-91839842:91841489-91841610:91848722-918	298	270	0.6353	0.4647	0.171	0.171	3.73712E-07	1.97E-05
RI	chr6	Aebp2	+	140650756-140650782:140650756-140655481:140651316-140655481	298	149	0.8087	0.6373	0.171	0.171	3.96088E-05	0.000386
SE	chr2	Pax6	+	105683828-105683958:105684751-105684792:105684887-105685102	190	149	0.3583	0.5297	-0.171	0.171	0.003346003	0.033044
SE	chr13	Fam193b	-	55554217-55554448:55555983-55556225:55570775-55571085	298	149	0.9443	0.7737	0.171	0.171	1.0321E-09	6.14E-08
SE	chr17	Telo2	-	25101299-25101704:25101784-25102110:25102637-25102728	298	149	0.974	0.803	0.171	0.171	4.52933E-07	1.5E-05
SE	chr5	Ablim2	+	35841281-35841401:35843168-35843266:35848804-35848905	247	149	0.925	0.7543	0.171	0.171	0.000114352	0.001868
SE	chr7	Fam71e1	+	44496600-44496730:44496915-44497064:44497503-44497633	298	149	1	0.829	0.171	0.171	2.46609E-08	1.1E-06
SE	chr8	Ankrd10	-	11619062-11619297:11623697-11623858:11628439-11628530	298	149	0.2183	0.047	0.171	0.171	7.77156E-16	1.25E-13
SE	chr1	Prrc2c	-	162671785-162673656:162674099-162674151:162675605-162675841	201	149	0.2457	0.417	-0.171	0.171	0.000298725	0.004259
A3SS	chr11	Zfp207	+	80402646-80402724:80403365-80403662:80403601-80403662	298	149	0.9047	0.735	0.17	0.17	1.07464E-07	7.96E-06
A5SS	chr16	Dlg1	+	31796866-31796965:31796866-31797010:31805614-31805734	193	149	0.7843	0.6147	0.17	0.17	0.001422543	0.015018
MXE	chr7	Actn4	-	28906972-28907064:28909903-28909988:28911490-28911575:28912240-289	234	234	0.24	0.4097	-0.17	0.17	0.000541777	0.009239
MXE	chr1	Ptprn	-	75258379-75258637:75260571-75260667:75262027-75262140:75262323-752	262	245	0.578	0.4077	0.17	0.17	0.000144138	0.003073
RI	chr11	Mmgt2	+	62664670-62665236:62664670-62666356:62665744-62666356	298	149	0.655	0.485	0.17	0.17	0.002810694	0.014874
SE	chr4	Epb41	-	131963929-131963970:131964131-131964187:131967788-131967972	205	149	0.9847	0.8143	0.17	0.17	1.24111E-08	5.94E-07
SE	chr1	Armc9	+	86244566-86244670:86252277-86252392:86257226-86257362	264	149	0.8987	0.7287	0.17	0.17	0.000247356	0.003628
SE	chr2	D430041D05Rik	-	104254956-104255570:104258164-104258508:104409929-104410334	298	149	0.5057	0.6757	-0.17	0.17	0.000151138	0.002374
SE	chr5	Cdc7	+	106975485-106975660:106979316-106979444:106982964-106984439	277	149	0.9757	0.8053	0.17	0.17	1.39423E-05	0.000306
SE	chr17	Mapk8ip3	-	24907329-24907386:24908411-24908428:24909150-24909161	166	149	0.846	0.6763	0.17	0.17	1.9158E-08	8.7E-07
SE	chr1	Clk1	-	58414590-58414684:58417013-58417179:58417267-58417383	298	149	0.9777	0.808	0.17	0.17	0	0
SE	chr5	Zfp644	-	106619540-106619642:106635610-106638635:106666785-106666845	298	149	0.9367	0.767	0.17	0.17	6.9446E-06	0.000167
SE	chr5	Hip1	-	135460407-135460470:135465130-135465260:135489081-135489328	279	149	0	0.1703	-0.17	0.17	1.49514E-12	1.52E-10
SE	chr13	Homer1	+	93355940-93356079:93386345-93386380:93387524-93387680	184	149	0.9193	0.749	0.17	0.17	1.50248E-07	5.59E-06
SE	chr9	Commd4	-	57155412-57155588:57155902-57156064:57156172-57156291	298	149	1	0.8303	0.17	0.17	0	0
SE	chr10	Ltv1	-	13179123-13179323:13179655-13179707:13179995-13180134	201	149	0.9757	0.8057	0.17	0.17	1.51178E-07	5.61E-06
SE	chr13	4833439L19Rik	-	54561693-54561871:54564185-54564272:54565182-54565375	236	149	0.313	0.483	-0.17	0.17	5.58695E-05	0.001017
SE	chr7	Tenm4	+	96797320-96797505:96805105-96805131:96811833-96811979	175	149	0.6453	0.4753	0.17	0.17	0.00359792	0.035122
A3SS	chr7	Folr1	-	101869157-101869304:101868398-101868554:101868398-101868467	235	149	0.2727	0.1033	0.169	0.169	0.003561404	0.039462
A3SS	chr9	Cdv3	-	103356280-103356439:103353102-103356026:103353102-103355313	298	149	0.6797	0.5107	0.169	0.169	1.44763E-05	0.000513
A5SS	chr7	Zfp939	+	39472858-39474439:39472858-39474772:39477311-39477427	298	149	0.914	0.745	0.169	0.169	0.001720828	0.017595
A5SS	chr19	Rpp30	+	36100172-36100239:36100172-36100288:36100809-36100838	197	149	0.9227	0.754	0.169	0.169	3.15886E-07	1.46E-05
MXE	chr2	Dph7	+	24962174-24962810:24963023-24963156:24965557-24965654:24966502-249	282	246	0.232	0.0633	0.169	0.169	1.57565E-08	1.17E-06
MXE	chr2	Macrod2	+	140395356-140395721:140400862-140400978:140452579-140452686:14082	265	256	0.7267	0.8957	-0.169	0.169	2.47148E-10	2.92E-08

MXE	chr9	Tcf12	-	71922652-71922787:71943954-71944018:72006701-72006762:72015638-720	210	213	0.128	0.2967	-0.169	0.169	0.000131257	0.002839
RI	chr9	Dalrd3	+	108571782-108571864:108571782-108572297:108572187-108572297	298	149	0.988	0.819	0.169	0.169	0	0
SE	chr1	Map4k4	+	40000531-40000749:40003763-40003987:40004807-40004913	298	149	0.8307	0.6613	0.169	0.169	1.47781E-11	1.23E-09
SE	chr4	Srsf10	+	135863040-135863202:135863526-135863576:135867126-135869908	199	149	0.9923	0.823	0.169	0.169	0	0
SE	chr13	Pfkp	-	6588489-6596062:6597859-6598011:6598739-6598826	298	149	0.775	0.9437	-0.169	0.169	6.03994E-06	0.000148
SE	chr7	Tgfb1i1	+	128248420-128248472:128248583-128248720:128248887-128248974	286	149	0.9383	0.7693	0.169	0.169	0.000193584	0.002941
SE	chr2	Ppig	+	69723088-69723363:69731746-69731813:69732197-69732274	216	149	0.963	0.7943	0.169	0.169	8.07667E-07	2.53E-05
SE	chr6	Ldhb	-	142490249-142490604:142492493-142492616:142494100-142494217	272	149	0.9343	0.7653	0.169	0.169	0	0
SE	chr9	Dnajc13	-	104237577-104237726:104238465-104238540:104246760-104246840	224	149	0.9697	0.801	0.169	0.169	1.99217E-07	7.2E-06
SE	chr4	Rnf38	-	44152360-44152553:44158903-44159052:44167627-44167972	298	149	0.429	0.5977	-0.169	0.169	1.41165E-05	0.00031
SE	chr9	Snrk	+	122117266-122117407:122131893-122131942:122136906-122137601	198	149	0.6963	0.865	-0.169	0.169	0.000931727	0.011258
SE	chr9	Zfp810	-	22276737-22279280:22280453-22280559:22283168-22283260	255	149	0.928	0.759	0.169	0.169	2.06892E-07	7.44E-06
SE	chr5	Zfp932	+	109996502-109996633:110003353-110003452:110006944-110007070	248	149	0.5543	0.723	-0.169	0.169	0.002704153	0.02763
SE	chrX	Atp11c	-	60223283-60225793:60229148-60229252:60236557-60236710	253	149	0.982	0.813	0.169	0.169	2.58524E-07	9.07E-06
A5SS	chr16	Kalrn	-	34256191-34256360:34256152-34256360:34252225-34252399	187	149	0.729	0.5613	0.168	0.168	0.001068994	0.0123
MXE	chr18	Fhod3	+	25001791-25002032:25028065-25028169:25056260-25056394:25060326-250	253	283	0.468	0.3	0.168	0.168	0.000526627	0.009089
MXE	chrX	Pdzd4	-	73796376-73796477:73796830-73796892:73798194-73798292:73798758-737	247	211	0.3287	0.497	-0.168	0.168	6.69255E-07	3.25E-05
MXE	chr2	Cacna1b	-	24622599-24622707:24631899-24632026:24635096-24635240:24637426-246	293	276	0.8577	0.69	0.168	0.168	1.00497E-06	4.44E-05
MXE	chr17	Bag6	+	35146026-35146169:35146267-35146353:35146757-35146903:35147141-351	235	295	0.2527	0.421	-0.168	0.168	1.49988E-09	1.4E-07
MXE	chr15	Shank3	+	89543100-89543230:89543794-89543878:89547626-89549882:89557735-895	233	298	0.669	0.5013	0.168	0.168	0.000105734	0.002354
MXE	chr17	Cacna1h	-	25384979-25385079:25385561-25385983:25386970-25387178:25387479-253	298	298	0.405	0.5733	-0.168	0.168	0.000815158	0.012747
MXE	chr14	Erc2	+	28302876-28303010:28317249-28317314:28383824-28384043:28475603-284	214	298	0.8403	0.6723	0.168	0.168	8.20014E-08	5.17E-06
RI	chr9	Adamts7	+	90192899-90193865:90192899-90194269:90194043-90194269	298	149	0.834	0.6663	0.168	0.168	0.003320285	0.017135
SE	chr5	Kmt2e	+	23433129-23434783:23446526-23446598:23450152-23450336	221	149	0.1063	0.2747	-0.168	0.168	3.5083E-14	4.54E-12
SE	chr11	Unc119	+	78347218-78347331:78347797-78347899:78348087-78348259	251	149	0.939	0.771	0.168	0.168	2.22045E-16	3.82E-14
SE	chr4	Elavl2	-	91250766-91253570:91254141-91254179:91263992-91264145	187	149	0.991	0.823	0.168	0.168	5.57814E-08	2.28E-06
SE	chr11	Srr	-	74912962-74913133:74919437-74919662:74925619-74925755	298	149	0.5577	0.3897	0.168	0.168	7.9793E-06	0.000188
SE	chr10	Grip1	+	119818641-119819634:119897715-119897795:119929901-119930036	229	149	0.7917	0.96	-0.168	0.168	8.29456E-06	0.000194
SE	chr14	Rnf31	+	55601881-55601938:55602603-55602699:55603256-55603424	245	149	0.9527	0.785	0.168	0.168	2.20462E-06	6.08E-05
SE	chr5	Cdc7	+	106975485-106975660:106979295-106979444:106982964-106984439	298	149	0.975	0.8067	0.168	0.168	1.5311E-05	0.000331
A3SS	chr1	Diexf	-	193114605-193114717:193113757-193114002:193113757-193113887	263	149	0.814	0.647	0.167	0.167	0.000210705	0.004382
A3SS	chr12	Acp1	-	30905069-30905142:30904718-30904983:30904718-30904831	298	149	0.306	0.1387	0.167	0.167	1.93989E-12	4E-10
A3SS	chr8	Polb	-	22639934-22640004:22637068-22637154:22637068-22637099	203	149	0.795	0.6277	0.167	0.167	6.88647E-07	3.68E-05
A5SS	chr13	4833420G17Rik	+	119473833-119473956:119473833-119474194:119474833-119474924	298	149	0.5517	0.7187	-0.167	0.167	7.82543E-08	4.59E-06
MXE	chr2	Dph7	+	24962172-24962810:24963023-24963156:24965560-24965654:24966502-249	282	243	0.2367	0.07	0.167	0.167	6.0658E-08	4.05E-06
MXE	chr5	Ttc28	+	110879784-110879988:110892832-110893110:111085022-111085169:11110	298	296	0.1793	0.3467	-0.167	0.167	1.20244E-07	7.3E-06
MXE	chr2	Pkp4	+	59347924-59348041:59350494-59350622:59352262-59352335:59354203-593	277	222	0.5943	0.427	0.167	0.167	1.21382E-05	0.000376
RI	chr1	Abl2	+	156640996-156641229:156640996-156649568:156641542-156649568	298	149	0.9143	0.7473	0.167	0.167	9.20281E-05	0.000803
SE	chr2	Dlgap4	+	156748023-156748102:156748332-156748420:156748944-156749356	237	149	0.4923	0.6597	-0.167	0.167	2.3607E-05	0.000483
SE	chr1	Pogk	-	166393610-166400165:166401866-166401964:166408399-166408532	247	149	0.8507	0.684	0.167	0.167	3.21424E-05	0.000633
SE	chr17	Zfp944	-	22343640-22343693:22355081-22355134:22358823-22358915	202	149	0.1533	0.3203	-0.167	0.167	0.003472136	0.03406
SE	chr7	Cars	-	143559649-143559708:143561859-143561926:143562961-143563041	216	149	0.9443	0.777	0.167	0.167	8.21618E-11	5.91E-09
SE	chr13	Erbb2ip	-	103823314-103823406:103824720-103824926:103833498-103835028	298	149	0.7277	0.5603	0.167	0.167	0.001407726	0.015931
SE	chr19	Vldlr	+	27234790-27234912:27236240-27236362:27238053-27238424	271	149	0.683	0.8497	-0.167	0.167	5.558E-05	0.001012
SE	chr9	Usp3	-	66540062-66540175:66542513-66542613:66543946-66544028	249	149	0.9927	0.8253	0.167	0.167	3.16293E-11	2.48E-09
SE	chr17	Ppard	+	28286261-28286467:28295341-28295495:28297086-28297224	298	149	0.9607	0.7933	0.167	0.167	3.0975E-05	0.000613
SE	chr9	Rnf111	-	70475776-70476668:70502940-70503002:70503459-70503548	211	149	0.433	0.5997	-0.167	0.167	0.001269735	0.014638
SE	chr8	Tcf25	+	123400625-123400697:123401058-123401189:123403147-123403815	280	149	0.0187	0.186	-0.167	0.167	0	0
SE	chr18	Ythdc2	+	44847744-44847879:44850308-44850434:44850636-44850701	275	149	0.9487	0.782	0.167	0.167	0.000689549	0.008727
SE	chr11	Trim41	-	48806403-48807846:48808125-48808252:48809050-48809280	276	149	0.9773	0.81	0.167	0.167	4.86731E-11	3.7E-09
SE	chr5	Srpk2	-	23525496-23525745:23526790-23526815:23527924-23528062	174	149	0.9957	0.8287	0.167	0.167	4.55191E-15	6.77E-13
SE	chr16	Mcm4	-	15634439-15634577:15635049-15635144:15635230-15635325	244	149	0.974	0.807	0.167	0.167	1.21896E-07	4.62E-06
SE	chr12	Trim9	-	70255038-70255226:70267234-70267266:70268062-70268200	181	149	0.7983	0.6317	0.167	0.167	0.00278975	0.028343
SE	chrX	Fgf13	-	59131840-59131950:59250799-59250966:59567140-59567348	298	149	0.6913	0.8583	-0.167	0.167	2.9901E-11	2.36E-09
SE	chr5	Mapre3	+	30862498-30862643:30863204-30863405:30864574-30864728	298	149	0.936	0.7693	0.167	0.167	9.85692E-07	3.01E-05
SE	chr2	Scn2a1	+	65735609-65735890:65743065-65743118:65748294-65748431	202	149	0.9983	0.8317	0.167	0.167	0	0
A3SS	chr10	Izumo4	+	80704180-80704194:80704290-80704435:80704418-80704435	276	149	0.3987	0.2327	0.166	0.166	0.00012547	0.002893
A5SS	chr1	Rnpep	-	135267170-135267282:135267129-135267282:135266870-135266978	189	149	0.2143	0.38	-0.166	0.166	0.000188202	0.002936
A5SS	chr4	Rims3	+	120854779-120854933:120854779-120855122:120872477-120872650	298	149	0.9167	0.7503	0.166	0.166	8.52767E-08	4.87E-06
MXE	chr12	Nrcam	+	44576604-44576789:44578145-44578180:44584835-44584987:44590108-445	184	298	0.8997	0.7337	0.166	0.166	6.83089E-05	0.001645
MXE	chr16	Lrch3	+	32981828-32981904:32985976-32986027:32986739-32986888:32988431-329	200	298	0.5637	0.3973	0.166	0.166	0.002504326	0.030866
MXE	chr11	Spag9	+	94013623-94013743:94036600-94036670:94043572-94043666:94048408-940	219	243	0.4063	0.2403	0.166	0.166	0.003240724	0.037601
MXE	chr18	Fam13b	-	34462961-34463009:34465156-34465220:34473550-34473685:34486979-344	284	213	0.5113	0.6777	-0.166	0.166	4.77099E-06	0.00017
SE	chr13	Pdlim7	-	55507864-55507880:55508208-55508326:55508780-55508810	267	149	0.8987	0.733	0.166	0.166	7.75952E-11	5.6E-09
SE	chr11	Ogdh	+	6316765-6316855:6324878-6324922:6334533-6334648	193	149	0.5353	0.7013	-0.166	0.166	0.000369263	0.005091
SE	chr3	Gm3764	+	88227366-88227624:88228559-88228694:88229012-88229365	284	149	0.4943	0.328	0.166	0.166	2.23763E-08	1E-06
SE	chr12	Rock2	+	16928945-16929034:16934241-16934346:16940403-16940540	254	149	0.068	0.2337	-0.166	0.166	2.00005E-06	5.61E-05
SE	chr14	Pbrm1	+	31054158-31054255:31056236-31056280:31061469-31061745	193	149	0.5497	0.716	-0.166	0.166	0.001623307	0.017902
SE	chr4	Mib2	-	155657855-155657959:155658894-155659043:155659123-155659215	298	149	0.2587	0.425	-0.166	0.166	2.47182E-06	6.73E-05
SE	chr3	Ccnl1	-	65951434-65951554:65955204-65955276:65956853-65956962	221	149	0.4667	0.6323	-0.166	0.166	0.005111884	0.047044
SE	chr4	Mysm1	-	94950926-94951058:94952174-94952377:94952863-94952937	298	149	1	0.8343	0.166	0.166	9.31886E-07	2.87E-05
SE	chr2	Phf21a	+	92320229-92320459:92326913-92327164:92328312-92328401	298	149	0.9843	0.8187	0.166	0.166	7.87148E-07	2.47E-05
SE	chr2	Rbm39	-	156172828-156177385:156178880-156178952:156179177-156179240	221	149	0.3283	0.494	-0.166	0.166	1.55201E-08	7.23E-07
SE	chr17	Sik1	-	31851533-31851596:31854208-31854324:31855672-31855792	265	149	0.9713	0.8057	0.166	0.166	6.99734E-06	0.000168
SE	chr7	Tnrc6a	+	123179717-123179859:123180249-123180395:123180540-123180677	295	149	0.5337	0.368	0.166	0.166	0.000176522	0.002716
SE	chr1	Nfasc	-	132576286-132576417:132593351-132593365:132595484-132595657	163	149	0.8893	0.723	0.166	0.166	0.001898713	0.020404
SE	chr5	Ept1	+	30247463-30247531:30248412-30248520:30252710-30252784	257	149	0.8933	0.7277	0.166	0.166	1.67996E-06	4.79E-05
SE	chr2	Smox	+	131520412-131521171:131522148-131522219:131524743-131525183	220	149	0.846	0.6797	0.166	0.166	0.000217013	0.003235
SE	chrX	Gripap1	+	7803323-7803400:7803594-7803686:7804360-7804494	241	149	0.8727	0.7063	0.166	0.166	6.47693E-09	3.32E-07
SE	chr7	Gramd1a	-	31138114-31138257:31138752-31138950:31139397-31139547	298	149	0.973	0.8073	0.166	0.166	0	0
SE	chr13	4833439L19Rik	-	54556527-54556651:54564185-54564272:54565182-54565375	236	149	0.2607	0.427	-0.166	0.166	0.001178063	0.013713
A3SS	chr2	Frmd5	-	121547972-121548023:121545529-121547897:121545529-121547648	298	149	0.9463	0.7817	0.165	0.165	1.5083E-05	0.000525
A5SS	chr2	Dph7	+	24962174-24962625:24962174-24962810:24963023-24963156	298	149	0.3907	0.2253	0.165	0.165	7.7162E-07	3.08E-05
A5SS	chr1	Slc19a2	+	164256747-164257235:164256747-164257349:164260792-164261017	262	149	0.9617	0.7963	0.165	0.165	6.9315E-05	0.001333
A5SS	chr17	Fer	+	63981480-63981621:63981480-63981686:63991594-63991716	213	149	0.9533	0.788	0.165	0.165	3.21522E-07	1.47E-05
A5SS	chr14	Uggt2	-	119001771-119001885:119000729-119001885:118998097-118998213	298	149	0.5163	0.351	0.165	0.165	0.003992495	0.033934
A5SS	chr5	Dgkq	-	108650817-108650968:108650490-108650968:108649825-108650379	298	149	0.9643	0.7997	0.165	0.165	0.000169253	0.002724
MXE	chr13	Erbb2ip	-	103824720-103824926:103827907-103828023:103830188-103830331:10383	292	265	0.1987	0.3637	-0.165	0.165	0.000673263	0.010931
MXE	chr4	Tle1	-	72141827-72141880:72145310-72145420:72157463-72157479:72158213-721	165	259	0.107	0.2717	-0.165	0.165	0.000540383	0.009226
MXE	chr12	Brf1	-	112979685-112979757:112983027-112983058:112983733-112983849:11298	265	180	0.0197	0.1847	-0.165	0.165	2.40578E-09	2.13E-07
MXE	chr4	Eif4g3	+	138045343-138045418:138082893-138082988:138095909-138095929:13809	244	169	0.7293	0.8947	-0.165	0.165	4.7887E-05	0.001219
SE	chr1	Shisa4	-	135372197-135372364:135373152-135373285:135374378-135374549	282	149	0.973	0.8077	0.165	0.165	3.33067E-16	5.56E-14
SE	chr5	Ncor2	-	125036821-125036918:125037062-125037166:125037274-125037376	253	149	0.9247	0.76	0.165	0.165	9.34542E-07	2.88E-05
SE	chr19	Adrbk1	-	4287823-4287985:4288412-4288507:4288592-4288658	244	149	0.883	0.7177	0.165	0.165	2.13243E-07	7.65E-06
SE	chr3	Gm3764	+	88209635-88209716:88220079-88220325:88221103-88221284	298	149	0.3257	0.1603	0.165	0.165	1.25424E-06	3.73E-05
SE	chr17	Nfya	-	48395650-48395796:48398982-48399068:48400438-48400580	235	149	0.89	0.725	0.165	0.165	1.62398E-05	0.000348
SE	chr10	Tmpo	-	91153085-91153180:91153278-91153397:91158748-91158845	268	149	0.675	0.51	0.165	0.165	1.51081E-06	4.38E-05
SE	chr1	Scyl3	+	163938740-163938796:163939881-163939983:163943824-163943901	251	149	0.936	0.771	0.165	0.165	0.001305418	0.014971
SE	chr6	Repin1	+	48593883-48594052:48594862-48594976:48596293-48599082	263	149	0.8943	0.7297	0.165	0.165	0.003066528	0.030708
SE	chr12	Syt16	+	74129372-74129551:74162056-74162876:74222582-74222794	298	149	0.5303	0.6957	-0.165	0.165	3.18866E-08	1.38E-06
SE	chr14	Rcbtb2	+	73162327-73162477:73164684-73164850:73166480-73166638	298	149	0.9457	0.7807	0.165	0.165	3.42171E-10	2.22E-08
SE	chr3	Fnbp1l	-	122552136-122552150:122556412-122556570:122557903-122558106	298	149	0.949	0.7837	0.165	0.165	0	0
SE	chr5	Mpv17	-	31144700-31144752:31145246-31145278:31145495-31145590	181	149	0.745	0.58	0.165	0.165	5.75137E-05	0.001041
SE	chr4	Pum1	+	130701042-130701110:130730278-130730548:130741463-130741556	298	149	0.6413	0.806	-0.165	0.165	0.000705118	0.008889
SE	chr6	Ccdc136	+	29417072-29417578:29422037-29422097:29426625-29426955	209	149	0.8467	0.6817	0.165	0.165	0.004840654	0.04493
SE	chr1	Tceb1	-	16647955-16648007:16656296-16656459:16656862-16656897	298	149	0.8503	0.6857	0.165	0.165	0.001011684	0.012061
SE	chr5	Mphosph9	-	124259968-124260215:124260935-124261035:124261922-124262042	249	149	1	0.8347	0.165	0.165	3.58919E-07	1.22E-05
SE	chr5	Dgkq	-	108649825-108650379:108650490-108650638:108650817-108650968	297	149	0.954	0.789	0.165	0.165	0.000370196	0.005101
SE	chr4	Klhl17	-	156231085-156231173:156231547-156231714:156231802-156231946	298	149	0.8853	0.72	0.165	0.165	3.51635E-06	9.11E-05
SE	chr10	Myl6	-	128491290-128491347:128491720-128491764:128492059-128492136	193	149	0.8607	0.6957	0.165	0.165	1.93592E-06	5.46E-05
A3SS	chr19	Tmem179b	-	8774335-8774467:8773505-8773692:8773505-8773622	218	149	0.9133	0.7493	0.164	0.164	0.001492019	0.020705
A5SS	chr15	Xrcc6	+	82027225-82027411:82027225-82028977:82029122-82029290	298	149	0.2353	0.0717	0.164	0.164	1.0116E-08	7.14E-07
A5SS	chr11	Pcyt2	-	120613030-120613083:120612846-120613083:120612463-120612601	298	149	0.4353	0.2713	0.164	0.164	2.10197E-06	7.2E-05
MXE	chr11	Clk4	+	51267171-51267331:51268165-51268231:51268769-51268846:51271800-512	215	226	0.9863	0.822	0.164	0.164	4.40143E-09	3.69E-07
RI	chr11	Sec14l1	+	117156801-117156902:117156801-117159265:117158754-117159265	298	149	0.795	0.6313	0.164	0.164	9.56497E-09	2.28E-07
RI	chr4	Gm2164	-	124656840-124656965:124656840-124657504:124657266-124657504	298	149	0.9697	0.8057	0.164	0.164	5.61667E-05	0.000527
RI	chr16	Lrch3	+	33009189-33012202:33009189-33015608:33012341-33015608	286	149	0.71	0.8737	-0.164	0.164	0.002177524	0.012058
RI	chr5	Zfp644	-	106635610-106636323:106635610-106638635:106636417-106638635	241	149	0.572	0.4077	0.164	0.164	4.86354E-05	0.000463
SE	chr2	Stx16	+	174091482-174091622:174092402-174092567:174093434-174093525	298	149	0.8587	0.6947	0.164	0.164	5.20965E-09	2.73E-07
SE	chr10	Lin7a	+	107380080-107380151:107380227-107380303:107382504-107382713	225	149	0.0843	0.2483	-0.164	0.164	2.97737E-05	0.000594
SE	chr11	Tada2a	-	84109954-84110045:84118248-84118307:84126988-84127095	208	149	0.764	0.9283	-0.164	0.164	0.00461888	0.04323
SE	chr13	Tmem14c	+	41016256-41016398:41017115-41017268:41017706-41017782	298	149	0.2777	0.1137	0.164	0.164	6.39439E-10	3.97E-08
SE	chr4	Erp44	-	48219343-48219527:48236873-48236988:48241296-48241335	264	149	0.9307	0.7663	0.164	0.164	5.63526E-09	2.93E-07
SE	chr5	Wbscr22	-	135060734-135060830:135061073-135061155:135061249-135061344	231	149	0.9447	0.781	0.164	0.164	3.15624E-08	1.37E-06

SE	chr5	Fip1l1	+	74557040-74557149:74564519-74564626:74571099-74571192	256	149	0.6433	0.4797	0.164	0.164	5.34265E-05	0.000978
SE	chr11	Thoc5	+	4910627-4910759:4911435-4911470:4912781-4912858	184	149	0.4533	0.2897	0.164	0.164	4.97507E-05	0.00092
SE	chr12	Zc3h14	+	98774245-98774319:98779108-98779267:98783582-98783702	298	149	0.3433	0.5073	-0.164	0.164	0.000453276	0.0061
SE	chr15	Ppp1r16a	+	76693014-76693138:76693227-76693352:76693595-76693724	274	149	0.8857	0.7217	0.164	0.164	0.000216739	0.003233
SE	chr12	Dus4l	-	31642764-31642886:31646621-31646738:31648766-31648887	266	149	0.9547	0.791	0.164	0.164	0.000733556	0.009209
SE	chr5	Ep400	-	110739296-110739681:110740182-110740289:110741970-110742060	256	149	0.8787	0.7147	0.164	0.164	0.000272271	0.00394
SE	chr10	Mdm1	+	118146595-118146971:118150789-118150956:118152002-118152096	298	149	1	0.8357	0.164	0.164	6.72307E-05	0.001185
SE	chr16	Son	+	91671349-91671465:91674695-91674774:91675401-91675499	228	149	0.316	0.48	-0.164	0.164	0.000258987	0.003771
SE	chr3	Zc2hc1a	+	7526269-7526417:7528030-7528129:7539076-7539183	248	149	1	0.836	0.164	0.164	3.9746E-14	5.1E-12
SE	chr1	Ipo9	-	135420292-135420353:135426924-135427041:135430268-135430441	266	149	0.1437	0.3077	-0.164	0.164	4.63527E-10	2.96E-08
SE	chrX	Gripap1	+	7792228-7792337:7794992-7795099:7799290-7799428	256	149	0.5357	0.6993	-0.164	0.164	7.79941E-06	0.000184
SE	chr10	Polr3b	+	84692797-84693006:84695555-84695713:84696949-84697066	298	149	1	0.836	0.164	0.164	2.06786E-08	9.33E-07
SE	chr9	Pkm	+	59675029-59675195:59675568-59675734:59678044-59678225	298	149	0.1257	0.2897	-0.164	0.164	0	0
SE	chr3	Hltf	+	20092678-20092816:20098080-20098215:20105641-20105765	284	149	0.9573	0.793	0.164	0.164	0.000299829	0.004273
SE	chr11	4933427D14Rik	-	72189548-72189758:72191278-72191344:72191419-72191634	215	149	0.2953	0.4593	-0.164	0.164	0.001459034	0.016405
SE	chr14	Rab2b	-	52268642-52268734:52268918-52269000:52275443-52275510	231	149	0.9873	0.8237	0.164	0.164	0	0
A3SS	chr10	Kcnc2	+	112458490-112458654:112461965-112466304:112463991-112466304	298	149	1	0.8367	0.163	0.163	1.17281E-06	5.72E-05
A5SS	chr13	Fbxw17	+	50419840-50419877:50419840-50420028:50423202-50423362	298	149	0.156	0.319	-0.163	0.163	1.8874E-05	0.000446
MXE	chr3	Rrnad1	-	87927055-87927117:87927563-87927755:87927924-87928048:87930444-879	273	298	0.14	0.303	-0.163	0.163	0.001311975	0.018572
MXE	chr1	Nckap5	-	125981383-125981697:126014571-126014649:126023033-126023210:126024	298	227	0.6967	0.534	0.163	0.163	0.002879005	0.034292
MXE	chr14	Camk2g	-	20744604-20744648:20745792-20745860:20747819-20747851:20755659-207	181	217	0.886	0.7233	0.163	0.163	1.09382E-09	1.07E-07
RI	chr1	Ppox	-	171280140-171280255:171280140-171280522:171280388-171280522	280	149	0.6857	0.5223	0.163	0.163	0.000833516	0.005391
RI	chr13	Prpf4b	+	34901393-34904089:34901393-34906064:34905883-34906064	298	149	0.4973	0.3343	0.163	0.163	3.33672E-06	4.47E-05
RI	chr15	Kifc2	+	76662595-76662672:76662595-76662896:76662833-76662896	298	149	0.7217	0.8847	-0.163	0.163	1.53744E-07	2.87E-06
SE	chr16	Hspbap1	+	35813918-35814054:35814330-35814409:35817152-35817323	228	149	0.036	0.199	-0.163	0.163	3.69202E-05	0.000711
SE	chr3	Ank2	-	127104761-127104862:127248134-127248232:127339132-127339192	247	149	0.5493	0.7127	-0.163	0.163	0.000302524	0.004304
SE	chr10	Psap	+	60295946-60296002:60297540-60297545:60299098-60299229	154	149	0.475	0.3123	0.163	0.163	6.45426E-11	4.76E-09
SE	chr6	Tmcc1	-	116042871-116043805:116133767-116134029:116138195-116138250	298	149	0.9537	0.7907	0.163	0.163	0.000470434	0.006287
SE	chr19	9930021J03Rik	-	29786249-29786449:29791349-29791444:29805282-29808053	244	149	0.078	0.2407	-0.163	0.163	0.00034821	0.004848
SE	chr15	Smug1	-	103157617-103157918:103158157-103158227:103163272-103163437	219	149	0.5173	0.68	-0.163	0.163	0.004507706	0.042333
SE	chr2	Metap1d	+	71515637-71515800:71522533-71522630:71523802-71523849	246	149	0.971	0.8083	0.163	0.163	2.30383E-09	1.3E-07
SE	chr18	Diaph1	-	37905004-37905159:37906388-37906414:37935202-37935423	175	149	0.9797	0.817	0.163	0.163	1.59504E-09	9.16E-08
SE	chr7	Snrnp70	-	45380717-45380818:45381825-45383184:45383804-45383885	298	149	0.3007	0.464	-0.163	0.163	4.33975E-12	3.98E-10
SE	chr2	Kyat1	-	30194054-30194112:30197957-30198159:30205633-30205699	298	149	0.3613	0.1983	0.163	0.163	0.000991367	0.011846
SE	chr2	Ankrd16	+	11784291-11784452:11785123-11785260:11786224-11786302	286	149	0.4583	0.295	0.163	0.163	0.001188664	0.013814
SE	chr6	Amn1	-	149170833-149171050:149183394-149183538:149188606-149188712	293	149	0.7053	0.8683	-0.163	0.163	5.96931E-07	1.93E-05
SE	chr8	Pbx4	+	69866468-69866603:69870029-69870182:69870578-69870684	298	149	0.832	0.6693	0.163	0.163	0.000857276	0.010494
A3SS	chr2	Gfra4	-	131040399-131040475:131039632-131040325:131039632-131040258	215	149	0.9493	0.787	0.162	0.162	1.45569E-11	2.56E-09
A5SS	chr4	Mib2	-	155661323-155661374:155661106-155661374:155660850-155661021	298	149	0.908	0.7457	0.162	0.162	3.28542E-09	2.71E-07
A5SS	chr14	Kat6b	+	21624833-21624888:21624833-21625761:21626719-21626840	298	149	0.2847	0.4467	-0.162	0.162	0.002067811	0.020373
MXE	chr15	Cacnb3	+	98632301-98632520:98634148-98634251:98634436-98635213:98639488-986	252	298	0.087	0.2487	-0.162	0.162	9.9021E-06	0.000315
MXE	chr2	Ubr1	-	120870989-120871045:120873124-120873232:120875366-120875499:120881	282	257	0.046	0.208	-0.162	0.162	7.84864E-05	0.001838
MXE	chr2	Cers6	+	69068629-69068757:69071332-69071438:69105003-69105159:69108442-691	255	298	0.97	0.808	0.162	0.162	1.37035E-09	1.31E-07
MXE	chr3	Hltf	+	20092678-20092816:20099776-20099955:20105641-20105765:20106350-201	298	273	0	0.162	-0.162	0.162	5.32725E-09	4.37E-07
MXE	chr12	Papola	+	105809524-105809662:105812274-105812394:105813198-105813282:105818	269	233	0.047	0.2087	-0.162	0.162	4.26365E-09	3.59E-07
RI	chr7	2310022A10Rik	+	27580337-27580691:27580337-27582099:27581351-27582099	298	149	0.5727	0.4103	0.162	0.162	0.000219492	0.00168
SE	chr7	Shisa7	-	4830759-4830908:4831673-4831723:4834230-4834363	199	149	0.357	0.5193	-0.162	0.162	6.24907E-05	0.001115
SE	chr16	Fopnl	-	14311043-14311131:14313816-14314013:14317294-14317332	298	149	0.9643	0.8027	0.162	0.162	1.14653E-12	1.18E-10
SE	chr11	Taf15	+	83487223-83487413:83497262-83497296:83497894-83497926	183	149	0.938	0.7763	0.162	0.162	8.31924E-07	2.6E-05
SE	chr1	Tram1	-	13579450-13579566:13579690-13579811:13581023-13581086	270	149	0.9513	0.789	0.162	0.162	1.28023E-10	8.96E-09
SE	chr8	Adgrl1	+	83923019-83923108:83928298-83928382:83929464-83930264	233	149	0.1447	0.3067	-0.162	0.162	6.97754E-05	0.001225
SE	chr3	Lrrcc1	+	14533788-14534022:14535999-14536204:14536474-14536539	298	149	0.911	0.749	0.162	0.162	0.000258553	0.003767
SE	chr5	Rundc3b	-	8494784-8494905:8512261-8512407:8520859-8521016	295	149	0.187	0.3487	-0.162	0.162	3.75643E-08	1.6E-06
SE	chr12	Brms1l	+	55865951-55866077:55868162-55868405:55868497-55869736	298	149	0.9813	0.8197	0.162	0.162	3.23104E-11	2.53E-09
SE	chr12	Map4k5	-	69818384-69818498:69818809-69818811:69822803-69822863	151	149	0.9093	0.747	0.162	0.162	3.09048E-05	0.000612
SE	chr15	Zc3h3	-	75837467-75837663:75839256-75840594:75841850-75841936	298	149	0.9257	0.764	0.162	0.162	0.00461306	0.043182
SE	chr4	Szrd1	-	141118527-141118781:141120324-141120373:141139692-141139796	198	149	0.605	0.443	0.162	0.162	0.000120617	0.001955
SE	chr3	Ect2	-	27145956-27146045:27146837-27147019:27148993-27149072	298	149	0.9683	0.8067	0.162	0.162	1.05146E-05	0.00024
SE	chr2	Dnajc5	+	181520485-181520697:181521235-181521287:181546371-181546488	201	149	0.086	0.2477	-0.162	0.162	0	0
SE	chr3	Phtf1	+	103985753-103985909:103987427-103987561:103991038-103991211	283	149	0.9477	0.7857	0.162	0.162	0.000745091	0.009321
SE	chr6	Aak1	+	86956259-86956572:86959032-86959274:86963996-86964225	298	149	0.7217	0.8833	-0.162	0.162	0.000320988	0.004525
SE	chr3	Adgrl2	-	148826365-148826533:148827541-148827585:148828477-148828568	193	149	0.3423	0.1803	0.162	0.162	8.85883E-09	4.41E-07
SE	chr13	Gfm2	+	97165011-97165197:97168266-97168342:97171486-97171624	225	149	0.9907	0.829	0.162	0.162	7.52612E-07	2.38E-05
SE	chr7	Zfp446	+	12978998-12979378:12979451-12979646:12979885-12979979	298	149	0.9853	0.8233	0.162	0.162	2.67254E-05	0.000541
SE	chr5	Agap3	+	24496733-24496903:24498070-24498207:24499658-24499812	286	149	0.9287	0.7667	0.162	0.162	0.000145242	0.002296
A3SS	chrX	Fmr1	+	68710584-68710779:68711496-68712413:68712231-68712413	298	149	0.2533	0.092	0.161	0.161	4.57862E-07	2.64E-05
A3SS	chr3	4932438A13Rik	+	37022384-37022474:37026090-37026343:37026231-37026343	289	149	0.8987	0.738	0.161	0.161	5.19446E-06	0.000213
A3SS	chr9	Nfrkb	+	31396323-31396497:31396794-31397137:31397036-31397137	298	149	0.373	0.212	0.161	0.161	0.000473461	0.008457
A5SS	chr7	Gm9801	-	62207363-62207440:62207322-62207440:62206740-62206803	189	149	0.278	0.117	0.161	0.161	0.001212552	0.013474
A5SS	chr6	Cttnbp2	-	18433983-18435446:18433820-18435446:18427439-18427642	298	149	0.4093	0.2487	0.161	0.161	0.000377475	0.005191
MXE	chr4	Eif4g3	+	138045343-138045418:138057949-138058077:138082893-138082988:138095	277	244	0.5467	0.7073	-0.161	0.161	9.04201E-06	0.000292
MXE	chr10	Ahi1	+	21000506-21000705:21017913-21018033:21041186-21041241:21054981-210	269	204	0.1787	0.3397	-0.161	0.161	2.89781E-07	1.58E-05
RI	chr11	Lrrc45	+	120718442-120718553:120718442-120719132:120718650-120719132	244	149	0.6207	0.4597	0.161	0.161	0.000108174	0.000927
RI	chr8	Ssbp4	-	70597485-70597768:70597485-70598120:70598013-70598120	298	149	0.4263	0.265	0.161	0.161	1.61819E-11	6.05E-10
SE	chr9	Ncam1	-	49502134-49505563:49506853-49507653:49508589-49508705	298	149	0.4383	0.5993	-0.161	0.161	6.48911E-10	4.03E-08
SE	chr4	Plch2	-	155000065-155000185:155000448-155000711:155000804-155000897	298	149	1	0.839	0.161	0.161	1.35764E-07	5.09E-06
SE	chr11	Ogdh	+	6313788-6313979:6316765-6316855:6316978-6317080	239	149	0.3067	0.468	-0.161	0.161	0.000774283	0.009627
SE	chr1	Map4k4	+	40001430-40001591:40003763-40003987:40004807-40004913	298	149	0.9693	0.8087	0.161	0.161	0	0
SE	chr17	Gfer	-	24693191-24694442:24695254-24695450:24695831-24696156	298	149	0.9763	0.8153	0.161	0.161	8.8507E-13	9.22E-11
SE	chr5	Col26a1	-	136747021-136747068:136748324-136748359:136749265-136749317	184	149	1	0.8393	0.161	0.161	4.19936E-11	3.22E-09
SE	chr18	Hars2	+	36788577-36788670:36789157-36789284:36790134-36790250	276	149	0.9233	0.7623	0.161	0.161	6.94408E-05	0.00122
SE	chr2	Sh3glb2	-	30347081-30347104:30347483-30348617:30349226-30349237	298	149	0.9767	0.8153	0.161	0.161	9.0602E-09	4.5E-07
SE	chr16	Hira	+	18894774-18894836:18895903-18895927:18896454-18896564	173	149	0.2133	0.052	0.161	0.161	1.5149E-12	1.54E-10
SE	chr5	Jakmip1	+	37142793-37142858:37147951-37148029:37149101-37150614	227	149	0.949	0.7877	0.161	0.161	0.000448837	0.006051
SE	chr10	Herc4	+	63290504-63290676:63291180-63291283:63299120-63299208	252	149	0.153	0.3137	-0.161	0.161	0.001311456	0.015027
SE	chr9	Mthfsl	-	88688846-88688985:88703926-88704136:88715488-88715749	298	149	0.0803	0.2413	-0.161	0.161	4.48536E-05	0.000839
SE	chr1	Rnasel	+	153749426-153749607:153751459-153751667:153751796-153752059	298	149	0.8043	0.9653	-0.161	0.161	6.84507E-05	0.001205
SE	chr19	Sorbs1	-	40364994-40365166:40369690-40369719:40373476-40373617	178	149	0.2837	0.4443	-0.161	0.161	0.001103723	0.012971
A3SS	chr17	Tjap1	-	46264883-46264978:46263689-46263813:46263689-46263810	151	149	0.253	0.093	0.16	0.16	0.00110399	0.016392
A3SS	chr11	Lrrc45	+	120717480-120717538:120717641-120718172:120718037-120718172	298	149	0.9263	0.7667	0.16	0.16	6.53981E-05	0.001722
A3SS	chr2	Celf2	-	6560671-6560790:6553785-6553982:6553785-6553964	166	149	0.334	0.1743	0.16	0.16	2.69784E-13	6.28E-11
MXE	chr11	Slc38a10	-	120138986-120139129:120140593-120140686:120141600-120141645:120150	194	242	0.3317	0.1717	0.16	0.16	0.000400827	0.007156
MXE	chr7	Dlg2	+	92386915-92386990:92418088-92418133:92420632-92420673:92427691-924	194	190	0.4533	0.613	-0.16	0.16	9.86262E-05	0.002231
RI	chr16	Zbtb21	-	97945795-97950869:97945795-97953093:97952928-97953093	298	149	0.766	0.6057	0.16	0.16	0.011156189	0.04749
SE	chr18	Socs6	-	88865169-88870918:88886992-88887065:88887209-88887333	222	149	0.0857	0.2453	-0.16	0.16	7.92095E-05	0.001367
SE	chr8	Adgrl1	+	83923019-83923108:83924206-83924355:83928298-83928382	298	149	0.813	0.973	-0.16	0.16	0.00042203	0.005723
SE	chr14	Pbrm1	+	31022720-31022859:31023067-31023170:31025498-31025650	252	149	0.563	0.4027	0.16	0.16	0.005247136	0.048066
SE	chr1	R3hdm1	+	128175092-128175232:128176824-128176956:128178883-128178991	281	149	0.15	0.31	-0.16	0.16	1.25658E-06	3.73E-05
SE	chr2	Rbck1	-	152319124-152319303:152323172-152323332:152324267-152324440	298	149	0.8343	0.6743	0.16	0.16	0.003175057	0.031592
SE	chr1	Cdc42bpa	+	179999362-179999453:180024645-180024802:180031505-180031588	298	149	0.3367	0.4963	-0.16	0.16	0.00179822	0.019531
SE	chr9	Ano10	-	122175882-122176478:122182821-122182872:122201771-122201887	200	149	0.0157	0.176	-0.16	0.16	4.77086E-11	3.63E-09
SE	chr11	Camta2	-	70670549-70670736:70670891-70670911:70671008-70671079	169	149	0.923	0.7633	0.16	0.16	3.24177E-08	1.4E-06
SE	chr2	Spopl	-	23545444-23545579:23562968-23563055:23571843-23571917	236	149	0.1297	0.29	-0.16	0.16	0.001394173	0.015824
SE	chr6	Chl1	+	103711067-103711246:103714445-103714606:103715268-103715399	298	149	0.9563	0.7963	0.16	0.16	0	0
SE	chr14	Gpr180	+	118139737-118139895:118148085-118148285:118153569-118153749	298	149	0.9017	0.742	0.16	0.16	2.19742E-06	6.07E-05
SE	chr5	Git2	-	114739033-114739281:114743731-114743880:114745429-114745571	298	149	0.517	0.357	0.16	0.16	0.000831017	0.010213
SE	chr1	Stradb	+	58991013-58991136:58992204-58992375:58992651-58992755	298	149	0.951	0.791	0.16	0.16	3.72169E-12	3.43E-10
SE	chr16	Shisa9	+	11984118-11985143:11993994-11994050:11997053-11997180	205	149	0.087	0.2467	-0.16	0.16	0.000129913	0.002082
SE	chr1	Mark1	-	184896424-184898878:184900809-184900853:184905403-184905654	193	149	0.9773	0.8177	0.16	0.16	0	0
SE	chr12	Jkamp	+	72085839-72086183:72089333-72089406:72089956-72090110	222	149	0.9973	0.8377	0.16	0.16	4.44089E-16	7.35E-14
SE	chr3	Snx16	-	10417817-10419223:10420197-10420316:10420740-10420876	268	149	0.8653	0.7057	0.16	0.16	8.10745E-07	2.54E-05
SE	chr10	Sppl2b	+	80863455-80863551:80864038-80864154:80865121-80865202	265	149	0.8987	0.7387	0.16	0.16	4.15957E-07	1.39E-05
SE	chr6	Tmem209	-	30508454-30508512:30508666-30508802:30509685-30509770	285	149	0.8277	0.6677	0.16	0.16	0.003285351	0.032518
SE	chr4	1110017D15Rik	-	41505568-41505654:41507099-41507269:41507521-41507764	298	149	0.147	0.307	-0.16	0.16	0.000324971	0.004567
A3SS	chr11	Hdac5	-	102224734-102224955:102218416-102218781:102218416-102218487	298	149	0.1617	0.3207	-0.159	0.159	5.10725E-07	2.88E-05
MXE	chr3	Ank2	-	127056993-127057091:127104761-127104862:127248134-127248232:127339	247	250	0.578	0.7367	-0.159	0.159	0.0017516	0.023242
MXE	chr11	Flot2	+	78049478-78049559:78053363-78053453:78054785-78054875:78055757-780	239	239	0.1573	0.3167	-0.159	0.159	1.6841E-08	1.25E-06
MXE	chr4	Ptprd	-	76765700-76765758:76981506-76981539:77194296-77194343:77335411-773	196	182	0.7087	0.8673	-0.159	0.159	0.001155216	0.016903
MXE	chr9	Map4	+	109931489-109931940:109978850-109979091:109999772-109999840:110026	298	217	0.7073	0.8667	-0.159	0.159	6.47637E-12	1.13E-09
MXE	chr9	Ctsh	+	90062717-90062787:90064153-90064257:90064507-90064593:90065392-900	253	235	0.6723	0.5133	0.159	0.159	0.003892546	0.043213
MXE	chr9	U2surp	-	95495835-95495933:95500688-95500819:95502540-95502584:95511817-955	193	280	0.0813	0.24	-0.159	0.159	7.06309E-07	3.38E-05
MXE	chr10	Txnrd1	+	82859206-82859300:82860524-82860630:82874613-82874735:82877266-828	255	271	0.8277	0.6683	0.159	0.159	7.6669E-07	3.61E-05
MXE	chr7	Dlg2	+	92375554-92375730:92386915-92386990:92417224-92417323:92420632-924	224	248	0.6797	0.839	-0.159	0.159	1.55552E-06	6.36E-05
RI	chr2	Madd	-	91138709-91138755:91138709-91140626:91140557-91140626	298	149	0.5613	0.402	0.159	0.159	0.003597855	0.018378

RI	chr4	Dhrs3	+	144892917-144893375:144892917-144893709:144893585-144893709	298	149	0.9867	0.8273	0.159	0.159	2.58898E-05	0.000267
RI	chr11	Srsf1	+	88049043-88049215:88049043-88053757:88049412-88053757	298	149	0.272	0.4307	-0.159	0.159	1.12576E-10	3.81E-09
SE	chr15	Skp2	-	9123870-9124004:9125060-9125203:9139432-9139703	292	149	1	0.8407	0.159	0.159	0.00019619	0.002973
SE	chr5	Slc25a40	+	8447298-8447407:8449613-8449694:8452746-8452826	230	149	0.988	0.8287	0.159	0.159	7.43695E-05	0.001294
SE	chr6	Tmcc1	-	116042871-116043805:116133767-116134453:116138195-116138250	298	149	0.9567	0.7977	0.159	0.159	0.000405969	0.00553
SE	chr12	Sel1l	-	91831569-91831674:91833205-91833222:91841638-91841854	166	149	0.967	0.808	0.159	0.159	4.47308E-05	0.000837
SE	chr4	1110037F02Rik	+	11534189-11534321:11539910-11540062:11540481-11540641	298	149	0.9447	0.7857	0.159	0.159	7.63782E-07	2.41E-05
SE	chr17	Crim1	+	78237742-78237915:78302987-78303108:78313124-78313306	270	149	0.7737	0.933	-0.159	0.159	0.001015276	0.012097
SE	chr2	Cntrl	+	35113324-35113454:35116672-35116802:35118055-35118196	279	149	0.841	1	-0.159	0.159	1.40615E-05	0.000309
SE	chr16	Trmt2a	+	18251549-18251660:18252126-18252248:18252402-18252477	271	149	0.9487	0.7893	0.159	0.159	6.09957E-13	6.51E-11
SE	chr3	Zfp687	-	95010345-95012476:95013833-95013928:95015320-95015413	244	149	0.163	0.3223	-0.159	0.159	0.000111231	0.001828
SE	chr1	Mrpl30	+	37890553-37890584:37892943-37893023:37893939-37894015	229	149	0.7903	0.6317	0.159	0.159	3.80695E-13	4.21E-11
SE	chr7	Map6	+	99320499-99321285:99323692-99323999:99335985-99336052	298	149	0.8163	0.9753	-0.159	0.159	0.000146818	0.002317
SE	chr11	Fbxw11	+	32642003-32642935:32652748-32652904:32708188-32708250	298	149	0.2223	0.381	-0.159	0.159	6.40979E-05	0.001138
SE	chr16	Trap1	-	4056330-4056439:4060757-4060917:4062330-4062401	298	149	0.984	0.825	0.159	0.159	4.66693E-12	4.24E-10
SE	chr7	Shank2	+	144407121-144407232:144408056-144408076:144409462-144410968	169	149	0.9303	0.7717	0.159	0.159	0.000145069	0.002293
SE	chr1	Sdccag8	+	176824752-176824904:176826199-176826284:176828870-176828983	234	149	1	0.841	0.159	0.159	1.65821E-06	4.75E-05
SE	chr3	Slc44a5	+	153973423-153973798:154046929-154047012:154171569-154171617	232	149	0.0563	0.2157	-0.159	0.159	9.96674E-07	3.04E-05
SE	chr6	Wbp1	-	83120185-83120287:83120772-83120873:83121203-83121461	250	149	0.8783	0.7197	0.159	0.159	1.09646E-09	6.51E-08
SE	chr9	Apeh	-	108092981-108093074:108093622-108093748:108094227-108094359	275	149	0.913	0.7543	0.159	0.159	0.000173556	0.002677
SE	chr13	E430024I08Rik	-	74449956-74450016:74450236-74450362:74462582-74462666	275	149	0.9047	0.746	0.159	0.159	0.000493654	0.006553
SE	chr15	Cdh9	+	16728969-16729035:16777570-16777624:16778061-16778325	203	149	0	0.1587	-0.159	0.159	1.18983E-10	8.39E-09
SE	chr14	Ddhd1	-	45593171-45595664:45599102-45599185:45601565-45601756	232	149	0.1733	0.332	-0.159	0.159	5.72916E-05	0.001039
MXE	chr12	Slc8a3	-	81204892-81205016:81214066-81214169:81314260-81316104:81332641-813	298	252	0.2833	0.4417	-0.158	0.158	0.004516616	0.048154
MXE	chr4	Lrp8	+	107861133-107861357:107864009-107864077:107868978-107869150:107872	217	298	0.642	0.484	0.158	0.158	1.11821E-06	4.82E-05
RI	chr3	Ccnl1	-	65948691-65948790:65948691-65950544:65950480-65950544	298	149	0.348	0.506	-0.158	0.158	0.000253095	0.001885
SE	chr17	Nfya	-	48395650-48395796:48398982-48399065:48400438-48400580	232	149	0.8823	0.7243	0.158	0.158	8.17893E-05	0.001405
SE	chr2	Dync1i2	+	71228600-71228690:71233649-71233708:71235915-71236030	208	149	0.9157	0.758	0.158	0.158	0	0
SE	chr2	Rprd1b	+	158058652-158058827:158070561-158070656:158074930-158077320	244	149	0.219	0.3773	-0.158	0.158	0.002827151	0.028674
SE	chr10	Tcf3	-	80410398-80410624:80412827-80413062:80413205-80413337	298	149	0.6907	0.8487	-0.158	0.158	9.66698E-07	2.97E-05
SE	chr8	Pkn1	-	83671299-83671441:83671717-83671793:83671907-83671969	225	149	0.9003	0.742	0.158	0.158	5.84095E-11	4.35E-09
SE	chr7	Lrrc56	+	141207186-141207250:141207487-141207623:141207831-141207970	285	149	0.9247	0.7667	0.158	0.158	0.001800488	0.019552
SE	chr2	D430041D05Rik	-	104254956-104255570:104255968-104258508:104409929-104411771	298	149	0.4157	0.5737	-0.158	0.158	0.001963633	0.02102
SE	chrX	C230004F18Rik	+	61122012-61122159:61133366-61133503:61134721-61134796	286	149	0.9817	0.8237	0.158	0.158	1.26023E-05	0.00028
SE	chr11	Bcas3	+	85801785-85801952:85814000-85814065:85825202-85826058	214	149	0.1757	0.334	-0.158	0.158	0.000369004	0.005089
SE	chr14	Erc2	+	28302876-28303010:28317249-28317314:28475603-28478537	214	149	0.517	0.6753	-0.158	0.158	0.000260947	0.003797
SE	chr10	Slc39a3	-	81033612-81033933:81034918-81035009:81037204-81037337	240	149	0.9303	0.7723	0.158	0.158	1.56707E-08	7.28E-07
SE	chr2	Slc25a25	-	32420305-32420416:32420656-32420691:32421303-32421390	184	149	0.6673	0.5093	0.158	0.158	0.004700429	0.043863
SE	chr9	Dzip1l	+	99639814-99639898:99641734-99641852:99642533-99642694	267	149	0.9813	0.823	0.158	0.158	1.27351E-05	0.000283
A3SS	chr8	Tcf25	+	123400625-123400697:123401058-123401189:123401076-123401189	166	149	0.286	0.1287	0.157	0.157	1.15634E-05	0.000419
A5SS	chr14	Kat6b	+	21624833-21624888:21624833-21625215:21626719-21626840	298	149	0.262	0.4187	-0.157	0.157	0.002154572	0.021057
MXE	chr13	Faf2	+	54641419-54641553:54645245-54645321:54648292-54648430:54649978-546	225	287	0.3903	0.5477	-0.157	0.157	0.002421201	0.030134
RI	chr2	Kcnj3	+	55446826-55446844:55446826-55447042:55446957-55447042	260	149	0.989	0.8323	0.157	0.157	7.51657E-07	1.19E-05
RI	chr13	Srek1	-	103748759-103748854:103748759-103752702:103752394-103752702	298	149	0.3713	0.5283	-0.157	0.157	6.87757E-06	8.52E-05
RI	chr8	Slc10a7	+	78731609-78732149:78731609-78734005:78732487-78734005	298	149	0.9363	0.779	0.157	0.157	0.00438938	0.021791
RI	chr10	Zfr2	+	81249165-81249260:81249165-81249796:81249685-81249796	298	149	0.233	0.0763	0.157	0.157	2.29851E-08	5E-07
RI	chr19	Csf2ra	-	61225324-61225420:61225324-61225652:61225523-61225652	250	149	0.538	0.3813	0.157	0.157	3.79261E-06	5.02E-05
RI	chr13	Bicd2	+	49383043-49383253:49383043-49387026:49384971-49387026	298	149	0.8683	0.7113	0.157	0.157	1.68391E-05	0.000184
RI	chr13	Prpf4b	+	34901393-34902131:34901393-34902675:34902630-34902675	298	149	0.6613	0.504	0.157	0.157	1.1438E-05	0.000131
SE	chr1	Cep170	-	176758397-176758517:176761351-176761500:176769713-176769870	298	149	0.7977	0.641	0.157	0.157	1.36634E-06	4.01E-05
SE	chr9	Azi2	+	118059057-118059175:118059620-118059666:118061714-118063580	195	149	0.072	0.2293	-0.157	0.157	3.86583E-10	2.49E-08
SE	chr6	Zfp638	+	83954711-83954761:83957509-83957592:83958184-83958252	232	149	0.555	0.712	-0.157	0.157	0.000156815	0.002449
SE	chr4	Galt	+	41756513-41756561:41756682-41756811:41756939-41756995	278	149	0.6903	0.5333	0.157	0.157	0.002743507	0.02795
SE	chr4	Mier1	+	103115560-103115660:103118239-103118315:103127273-103127297	225	149	0.7953	0.638	0.157	0.157	0.001794829	0.019497
SE	chr17	Rhbdl1	-	25835969-25836193:25836399-25836572:25836988-25838367	298	149	0.3247	0.168	0.157	0.157	4.08513E-10	2.63E-08
SE	chr17	Nthl1	+	24633927-24634165:24634725-24634895:24635588-24635747	298	149	1	0.8427	0.157	0.157	2.75632E-05	0.000555
SE	chr2	Mtg2	+	180083345-180083460:180084064-180084312:180085380-180085902	298	149	0.9823	0.8257	0.157	0.157	1.29427E-06	3.83E-05
SE	chr2	Dph7	+	24962174-24962625:24963023-24963156:24965557-24965654	282	149	0.7947	0.6373	0.157	0.157	0.001170922	0.013641
SE	chr11	Lrrc75a	-	62604884-62606246:62609203-62609315:62620434-62620562	261	149	0.8987	0.742	0.157	0.157	0.000684562	0.008673
SE	chr14	Pcdh17	+	84477515-84477687:84517945-84518028:84532875-84533796	232	149	0.2077	0.3643	-0.157	0.157	0.000206635	0.00311
SE	chr5	Ythdc1	+	86820086-86820175:86820633-86820686:86821252-86821346	202	149	0.6037	0.761	-0.157	0.157	7.66007E-06	0.000182
SE	chr15	Rbfox2	-	77086067-77086155:77089862-77089893:77094451-77094490	180	149	0.2497	0.0923	0.157	0.157	0	0
SE	chr6	C2cd5	-	143017883-143018073:143020324-143020374:143029160-143029246	199	149	0.207	0.364	-0.157	0.157	0.000304763	0.004335
SE	chr12	Map3k9	-	81725722-81725834:81727331-81727399:81729683-81729836	217	149	0.781	0.6243	0.157	0.157	0.004477657	0.042141
A3SS	chr7	Fgfr2	-	130167703-130167808:130162451-130164215:130162451-130164123	240	149	0.3977	0.2413	0.156	0.156	0.002022961	0.025882
A3SS	chr1	Mgat4a	-	37490260-37490393:37466537-37466583:37466537-37466579	152	149	0.768	0.9237	-0.156	0.156	0.002988452	0.034782
A5SS	chr3	Dclk2	-	86792069-86792111:86791995-86792111:86786151-86787412	222	149	0.9227	0.7667	0.156	0.156	2.59426E-12	4.07E-10
A5SS	chr1	Prrc2c	-	162721445-162721539:162721430-162721539:162720481-162720606	163	149	0.7417	0.8977	-0.156	0.156	3.39095E-05	0.000718
MXE	chr12	Rian	+	109627048-109627153:109640267-109640328:109642565-109642652:109643	210	236	0.0463	0.2023	-0.156	0.156	1.2409E-10	1.53E-08
MXE	chr6	Ccdc136	+	29415431-29415655:29417072-29417578:29417932-29418153:29419102-294	298	298	0.958	0.8017	0.156	0.156	1.07942E-05	0.000339
MXE	chr2	Tmem87b	+	128841425-128841483:128842596-128842699:128845461-128845534:128846	252	222	0.484	0.6397	-0.156	0.156	0.003221309	0.037404
RI	chr16	Ece2	+	20644341-20644436:20644341-20646480:20645959-20646480	298	149	0.981	0.8247	0.156	0.156	9.08184E-06	0.000106
SE	chr9	Dopey1	+	86506405-86506519:86506871-86506900:86507504-86507737	178	149	0.9223	0.7663	0.156	0.156	0.000487067	0.006481
SE	chr1	Pogk	-	166394865-166400165:166403498-166403624:166408399-166408532	275	149	0.8427	0.6863	0.156	0.156	0.00013909	0.00221
SE	chr1	Sphkap	-	83362229-83362328:83384894-83384972:83407980-83408189	227	149	0.531	0.6867	-0.156	0.156	0.002165407	0.02285
SE	chr3	Dclk2	-	86824636-86824730:86830824-86830871:86831733-86831834	196	149	0.5553	0.399	0.156	0.156	2.54242E-08	1.13E-06
SE	chr4	Ece1	+	137913768-137913854:137921021-137921162:137926219-137926431	290	149	1	0.8437	0.156	0.156	1.25603E-08	6E-07
SE	chr2	Nkain4	-	180943030-180943227:180944077-180944214:180954571-180954699	286	149	0.707	0.5513	0.156	0.156	0.000173691	0.002678
SE	chr4	Tnc	-	63982651-63982923:64000698-64000970:64006246-64006509	298	149	0.7573	0.913	-0.156	0.156	0.000186098	0.002844
SE	chr11	Mettl2	+	105126776-105126867:105128741-105129075:105130447-105130496	298	149	0.9823	0.826	0.156	0.156	3.48656E-08	1.5E-06
SE	chr8	C230057M02Rik	-	122729771-122729894:122734088-122734273:122752114-122752648	298	149	0.027	0.1833	-0.156	0.156	8.53854E-09	4.26E-07
SE	chr10	Syt1	-	108993838-108993917:109004783-109004934:109010576-109010978	298	149	0.041	0.197	-0.156	0.156	3.24005E-06	8.48E-05
SE	chr6	Ccdc136	+	29419987-29420265:29421991-29422097:29426625-29426953	255	149	0.9567	0.801	0.156	0.156	0.000119284	0.001935
SE	chr1	Agfg1	+	82886111-82886187:82886375-82886467:82893424-82893582	241	149	0.8363	0.68	0.156	0.156	1.38246E-08	6.55E-07
SE	chr14	Sdr39u1	-	55897283-55898012:55898243-55898477:55899184-55899305	298	149	0.9213	0.7657	0.156	0.156	1.7242E-05	0.000367
SE	chr2	Odf2	+	29889242-29889278:29890766-29891002:29892186-29892276	298	149	0.0653	0.2217	-0.156	0.156	0.000202661	0.003059
SE	chr3	Gyg	-	20122084-20122864:20123122-20123172:20125258-20125477	199	149	0.5063	0.35	0.156	0.156	4.30837E-05	0.00081
SE	chrX	Irak1	-	74022185-74022249:74022473-74022661:74022849-74022952	298	149	0.9623	0.8063	0.156	0.156	0.000236279	0.003486
SE	chr14	Ppp3cc	-	70217864-70218277:70220017-70220046:70224310-70224407	178	149	0.9193	0.7637	0.156	0.156	3.66094E-05	0.000706
SE	chr9	Oxsr1	-	119284546-119284687:119290737-119290865:119294415-119294523	277	149	0.0317	0.1873	-0.156	0.156	3.74145E-14	4.83E-12
A3SS	chrX	Mospd2	-	164939643-164939745:164936169-164938692:164936169-164937303	298	149	0.9677	0.8127	0.155	0.155	1.28209E-06	6.21E-05
A5SS	chr17	Fam120b	+	15401745-15403129:15401745-15404397:15405755-15405935	298	149	0.198	0.3527	-0.155	0.155	1.77427E-06	6.21E-05
MXE	chr10	Pip5k1c	+	81314992-81315118:81315879-81315956:81317326-81317403:81317766-813	226	226	0.1333	0.2887	-0.155	0.155	0	0
RI	chrX	Porcn	-	8197975-8198904:8197975-8199152:8199067-8199152	298	149	0.972	0.8167	0.155	0.155	2.81837E-06	3.94E-05
RI	chr18	Osbpl1a	-	12755305-12756095:12755305-12756307:12756188-12756307	240	149	0.922	0.767	0.155	0.155	1.71702E-11	6.32E-10
RI	chr2	Strbp	-	37577770-37579749:37577770-37580806:37580673-37580806	298	149	0.981	0.8257	0.155	0.155	6.96206E-07	1.11E-05
SE	chr12	Rian	+	109640267-109640328:109642565-109642652:109643414-109643461	236	149	0.982	0.827	0.155	0.155	0	0
SE	chr6	Ptcd3	-	71905098-71905143:71907653-71907689:71907794-71907846	185	149	0.9817	0.8267	0.155	0.155	1.70286E-12	1.7E-10
SE	chr11	Usp34	+	23472950-23473054:23473170-23473294:23484102-23484181	273	149	0.6957	0.5403	0.155	0.155	0.002874608	0.029115
SE	chr3	Unc5c	+	141771018-141771182:141786857-141786913:141788871-141789062	205	149	0.0207	0.1757	-0.155	0.155	0.000106722	0.001763
SE	chr7	Lins1	+	66708639-66708728:66709294-66709435:66710230-66710746	290	149	1	0.845	0.155	0.155	2.29688E-05	0.000472
SE	chr4	Mib2	-	155657855-155657959:155658894-155659001:155659123-155659265	256	149	0.3693	0.5243	-0.155	0.155	4.6156E-05	0.00086
SE	chr5	Adgrl3	+	81771584-81771680:81787436-81787564:81792361-81792378	277	149	0.1927	0.3473	-0.155	0.155	0.000120689	0.001955
SE	chr2	D430041D05Rik	-	104254956-104255570:104255998-104258508:104409929-104411770	298	149	0.4653	0.6207	-0.155	0.155	0.001418334	0.016015
SE	chr9	Snrk	+	122131893-122131942:122132628-122132750:122136906-122137601	271	149	0.6993	0.5447	0.155	0.155	3.29968E-05	0.000647
SE	chr6	Amn1	-	149170833-149171050:149184992-149185124:149188606-149188712	281	149	0.5347	0.6897	-0.155	0.155	0.004366522	0.041218
SE	chr8	Nfix	-	84713766-84713857:84716141-84716288:84721643-84721818	296	149	0.1917	0.3467	-0.155	0.155	1.4988E-14	2.05E-12
SE	chr16	Dlg1	+	31842770-31842839:31846837-31846933:31847636-31847669	245	149	0.2323	0.0777	0.155	0.155	0.000509201	0.006726
SE	chr3	Hltf	+	20099776-20099955:20105641-20105765:20106350-20106528	273	149	1	0.8447	0.155	0.155	2.54707E-08	1.13E-06
SE	chr8	Heatr3	+	88158129-88158265:88161878-88161966:88164888-88165031	237	149	0.9887	0.8333	0.155	0.155	7.32858E-10	4.5E-08
A3SS	chr15	Hdac10	-	89124713-89124804:89124175-89124588:89124175-89124253	298	149	0.9953	0.8417	0.154	0.154	3.14593E-12	6.14E-10
A3SS	chr7	Zdhhc13	+	48799829-48799951:48801307-48801384:48801324-48801384	165	149	0.9383	0.7847	0.154	0.154	1.63913E-07	1.12E-05
A5SS	chr5	Mrps17	+	129715684-129715858:129715684-129715941:129716727-129716866	231	149	0.8703	0.716	0.154	0.154	0.00033638	0.004787
MXE	chr3	Pde7a	-	19229688-19229768:19230256-19230352:19230957-19231101:19233100-192	293	245	0.607	0.761	-0.154	0.154	7.57565E-05	0.001791
MXE	chr13	Gpbp1	-	111440632-111440816:111448933-111448999:111453024-111453247:111453	298	215	0.782	0.6283	0.154	0.154	8.86723E-06	0.000289
MXE	chr9	Lrrc49	-	60598086-60598195:60602579-60602816:60615094-60615177:60621569-606	232	298	0.9927	0.8387	0.154	0.154	0	0
MXE	chr14	Erc2	+	28302876-28303010:28317249-28317314:28383906-28384043:28475603-284	214	286	0.8417	0.688	0.154	0.154	6.6729E-07	3.25E-05
RI	chr13	Dapk1	+	60760635-60761863:60760635-60763191:60762357-60763191	298	149	0.83	0.6763	0.154	0.154	8.17006E-08	1.71E-06
RI	chr16	Abcc5	-	20400761-20401034:20400761-20402334:20402260-20402334	298	149	0.9517	0.7973	0.154	0.154	7.20757E-13	3.54E-11
SE	chr18	Ablim3	-	61808380-61808438:61808932-61808961:61811311-61811458	178	149	0.986	0.8323	0.154	0.154	0	0
SE	chr4	Mast2	-	116330391-116330469:116333413-116333433:116337422-116337497	169	149	0.9007	0.7467	0.154	0.154	2.61994E-07	9.17E-06
SE	chr10	Fam19a2	+	123264002-123265541:123430712-123430923:123593494-123593600	298	149	0.391	0.5453	-0.154	0.154	0.000452152	0.006087
SE	chr8	Rfx1	+	84066836-84067048:84073731-84074069:84079863-84079972	298	149	0.9563	0.802	0.154	0.154	9.1239E-06	0.000211
SE	chr18	Fhod3	+	25001791-25002032:25020662-25020757:25028065-25028169	244	149	0.0813	0.2357	-0.154	0.154	3.00036E-05	0.000598

SE	chr15	Adcy6	-	98589978-98592232:98592312-98592475:98592673-98592797	298	149	0.9783	0.8243	0.154	0.154	6.44261E-07	2.08E-05
SE	chrX	Hs6st2	-	51386637-51390361:51428658-51428744:51679890-51680408	235	149	0.6703	0.8247	-0.154	0.154	0.000402845	0.005494
SE	chr4	Nol9	+	152045695-152045830:152045948-152046035:152046547-152046644	236	149	0.876	0.722	0.154	0.154	0.001921543	0.020629
SE	chr7	Gltscr1	-	15987479-15989440:15993080-15993145:15996401-15996443	214	149	0.8893	0.735	0.154	0.154	0.002377013	0.024742
SE	chr16	Itsn1	+	91854573-91854655:91858209-91858375:91863257-91863378	298	149	1	0.8463	0.154	0.154	9.88098E-15	1.4E-12
SE	chr3	Adgrl2	-	148815855-148817928:148818648-148818686:148821280-148821408	187	149	0.3083	0.154	0.154	0.154	7.50998E-07	2.38E-05
SE	chr11	Sirt7	-	120618372-120619049:120619683-120619768:120620163-120620269	234	149	0.125	0.2793	-0.154	0.154	7.08519E-08	2.83E-06
SE	chr18	Mib1	+	10778149-10778300:10792895-10793027:10794478-10794564	281	149	0.9733	0.819	0.154	0.154	7.52192E-08	2.98E-06
SE	chr7	Aamdc	-	97565151-97565300:97569687-97569774:97579366-97579509	236	149	0.086	0.24	-0.154	0.154	0.000923566	0.011169
SE	chr9	Man2c1	+	57139246-57139401:57139598-57139708:57139870-57139964	259	149	1	0.8457	0.154	0.154	4.73123E-06	0.000118
SE	chr9	Neo1	-	58880592-58880855:58884470-58884628:58888401-58888493	298	149	0.3897	0.2353	0.154	0.154	9.15218E-09	4.54E-07
A5SS	chr5	Wdfy3	-	101900059-101900249:101900014-101900249:101898427-101898561	193	149	0.52	0.367	0.153	0.153	0.003395643	0.029994
A5SS	chr8	Ssbp4	-	70598013-70598120:70597897-70598120:70597485-70597768	264	149	0.3277	0.175	0.153	0.153	5.56666E-12	7.74E-10
MXE	chr3	Tars2	-	95746535-95746697:95747439-95747656:95748042-95748140:95750339-957	247	298	0.0887	0.2413	-0.153	0.153	0.000194015	0.003944
MXE	chr14	Ccser2	-	36874936-36879762:36885751-36885790:36890342-36890426:36896255-368	233	188	0.612	0.4593	0.153	0.153	0.00205905	0.0266
MXE	chr12	Meg3	+	109544027-109544742:109546430-109546542:109549479-109549552:109549	261	222	0.4063	0.559	-0.153	0.153	2.40719E-12	4.59E-10
MXE	chr1	Lrrfip1	+	91107298-91107390:91108580-91108672:91110355-91110447:91112156-911	241	241	0.2967	0.1433	0.153	0.153	1.95289E-06	7.75E-05
MXE	chr15	Ptk2	-	73343133-73343359:73365041-73365195:73391759-73391880:73394648-733	270	298	0.2573	0.1043	0.153	0.153	0.000646526	0.010587
MXE	chr19	Nrxn2	+	6513716-6513805:6516923-6517248:6519249-6519327:6531268-6533217	298	227	0.638	0.791	-0.153	0.153	1.33201E-09	1.28E-07
SE	chrX	Ocrl	+	47948125-47948360:47956210-47956233:47960453-47960569	172	149	0.872	0.7187	0.153	0.153	0.000160968	0.002503
SE	chr3	Ift80	-	68916092-68916263:68917957-68918104:68918490-68918625	296	149	0.9753	0.8223	0.153	0.153	2.9644E-05	0.000592
SE	chr9	Cyb561d2	-	107541003-107541040:107541497-107541650:107542035-107542188	298	149	0.951	0.7983	0.153	0.153	9.60189E-07	2.95E-05
SE	chr14	Pbrm1	+	31107094-31107310:31110415-31110579:31117013-31117083	298	149	0.2543	0.407	-0.153	0.153	0.005385275	0.049163
SE	chr18	Csnk1g3	+	53948048-53948154:53948645-53948668:53953245-53955578	172	149	0.8613	0.708	0.153	0.153	9.36181E-07	2.88E-05
SE	chr10	Tcf3	-	80410398-80410624:80412834-80413062:80413205-80413337	298	149	0.6897	0.843	-0.153	0.153	2.38074E-06	6.51E-05
SE	chr5	Srrd	-	112339897-112340123:112341096-112341136:112342875-112343040	189	149	0.9697	0.8163	0.153	0.153	6.13176E-13	6.53E-11
SE	chr10	Grik2	-	49099463-49101272:49104979-49105065:49113370-49113620	235	149	0.077	0.2303	-0.153	0.153	4.18554E-14	5.36E-12
SE	chr11	Otx1	-	21998370-21998521:21999380-21999585:22001122-22001189	298	149	1	0.8467	0.153	0.153	6.96908E-07	2.22E-05
SE	chr1	Tmem237	-	59117331-59117341:59117975-59118006:59119703-59119806	180	149	0.983	0.8297	0.153	0.153	5.3164E-11	4E-09
SE	chr2	Cdc123	-	5803947-5803975:5804911-5805033:5807431-5807506	271	149	0.9767	0.824	0.153	0.153	0	0
SE	chr14	Fam160b2	-	70590129-70590251:70590340-70590444:70591575-70591747	253	149	0.9503	0.7977	0.153	0.153	0.000153447	0.002403
A3SS	chr7	Ntrk3	-	78191650-78192744:78179986-78189405:78179986-78182940	298	149	0.9917	0.8397	0.152	0.152	0.00011164	0.002659
A3SS	chr5	Srrd	-	112339719-112339817:112338318-112338567:112338318-112338472	243	149	0.6127	0.4607	0.152	0.152	0.004193088	0.044562
A3SS	chrX	Cstf2	+	134072415-134072474:134073136-134073277:134073214-134073277	226	149	0.7217	0.8737	-0.152	0.152	0.00041393	0.0076
A5SS	chr6	Hnrnpa2b1	-	51461983-51462055:51461920-51462055:51460930-51461433	211	149	0.8043	0.6523	0.152	0.152	1.80525E-09	1.6E-07
A5SS	chr4	Rnf38	-	44167627-44167972:44167466-44167972:44152360-44152553	298	149	0.15	0.3017	-0.152	0.152	2.54129E-06	8.4E-05
MXE	chr12	Spata7	+	98631963-98632037:98633990-98634151:98634224-98634319:98637566-986	298	244	0.269	0.4207	-0.152	0.152	0.002804363	0.033586
MXE	chr2	Oprl1	+	181715690-181715946:181716502-181716535:181717955-181718093:181718	182	287	0.3473	0.4997	-0.152	0.152	0.001600276	0.021698
MXE	chr7	Fam168a	+	100706640-100706893:100786804-100786891:100812920-100813000:100824	236	229	0.8077	0.656	0.152	0.152	1.62844E-07	9.54E-06
MXE	chr7	Dhcr7	+	143830139-143830265:143836638-143836729:143840433-143840523:143841	240	239	0.093	0.2447	-0.152	0.152	4.68414E-06	0.000168
MXE	chr2	H13	+	152695482-152695584:152700183-152700268:152703995-152704194:152704	234	298	0.635	0.7873	-0.152	0.152	3.56037E-12	6.46E-10
MXE	chr4	Rars2	+	34630473-34630570:34633658-34633713:34639245-34639321:34645697-346	204	225	0.307	0.1547	0.152	0.152	0.001830713	0.024103
RI	chr8	Tmem161a	+	70180706-70180849:70180706-70181049:70180936-70181049	234	149	0.3803	0.2287	0.152	0.152	0.000502449	0.003474
RI	chr3	Srsf11	-	158022841-158022950:158022841-158024231:158023243-158024231	298	149	0.4377	0.286	0.152	0.152	6.64127E-07	1.07E-05
SE	chr12	Rian	+	109640267-109640328:109643414-109643461:109645262-109645331	196	149	0.986	0.8337	0.152	0.152	1.21464E-10	8.55E-09
SE	chrX	Arhgef9	-	95048930-95052154:95054229-95054290:95054943-95055011	210	149	0.39	0.2377	0.152	0.152	0.000493131	0.006549
SE	chr11	Tada2a	-	84084714-84084757:84085563-84085771:84087111-84087182	298	149	0.9923	0.8403	0.152	0.152	1.58765E-08	7.36E-07
SE	chr3	D930015E06Rik	-	83906347-83906425:83909152-83909334:83910369-83910508	298	149	0.4357	0.2833	0.152	0.152	1.19757E-06	3.58E-05
SE	chr5	Asphd2	-	112391158-112392255:112393460-112393583:112393785-112393999	272	149	0.6353	0.4837	0.152	0.152	0.001939254	0.020803
SE	chr11	Dhx33	-	70992108-70992311:70993610-70993698:70993857-70994016	237	149	0.9887	0.8367	0.152	0.152	0.000109004	0.001797
SE	chr5	Wbscr22	-	135053549-135053638:135056339-135056384:135056842-135056927	194	149	0.9883	0.8367	0.152	0.152	3.46455E-06	8.99E-05
SE	chr4	Srsf4	+	131873612-131873870:131884276-131884482:131891207-131891349	298	149	0.1537	0.3053	-0.152	0.152	7.6002E-07	2.4E-05
SE	chr4	Ptpru	-	131793518-131793675:131794961-131794990:131797853-131797991	178	149	0.9183	0.7667	0.152	0.152	0.000108622	0.001792
SE	chr13	Hmgcs1	+	119690351-119690513:119694989-119695047:119699664-119700121	207	149	0.3617	0.5137	-0.152	0.152	5.50662E-11	4.12E-09
SE	chr5	Fbxl5	-	43759743-43759825:43760707-43760855:43762817-43762942	297	149	0.9667	0.815	0.152	0.152	2.24265E-14	2.99E-12
SE	chr2	Prkra	-	76643223-76643304:76647125-76647294:76647792-76647994	298	149	0.908	0.756	0.152	0.152	9.10383E-15	1.3E-12
SE	chr7	Gdpd5	+	99387104-99387188:99405092-99405199:99410409-99410491	256	149	0.047	0.1993	-0.152	0.152	1.44833E-08	6.81E-07
SE	chr16	Hira	+	18894774-18894836:18895903-18896046:18896454-18896564	292	149	0.25	0.098	0.152	0.152	2.49257E-11	1.99E-09
SE	chr6	Tia1	+	86419101-86420411:86423606-86423671:86424345-86424420	214	149	0.251	0.099	0.152	0.152	7.55618E-13	7.92E-11
SE	chr10	Coq10a	-	128364405-128364506:128365023-128365215:128368007-128368153	298	149	0.9843	0.8323	0.152	0.152	3.91021E-13	4.31E-11
SE	chr2	Chd6	-	160961863-160962014:160964758-160964794:160965169-160966740	185	149	0.073	0.225	-0.152	0.152	1.63558E-06	4.7E-05
SE	chrX	Araf	+	20858394-20858512:20858742-20858873:20859979-20860519	280	149	0.9863	0.8343	0.152	0.152	1.519E-06	4.41E-05
SE	chr1	Ptprn	-	75260571-75260667:75262027-75262140:75262323-75262373	262	149	0.9733	0.821	0.152	0.152	6.53988E-12	5.81E-10
SE	chr14	Dmtn	-	70617285-70617440:70617955-70618029:70618239-70618408	223	149	0.3877	0.5397	-0.152	0.152	0.000587648	0.007626
SE	chr3	Rap1gds1	-	138965868-138965996:138983722-138983868:139015546-139015671	295	149	0.432	0.5837	-0.152	0.152	0.005302052	0.048497
SE	chr8	Ap1g1	+	109829599-109829675:109830613-109830621:109832717-109832812	157	149	0.8557	0.7037	0.152	0.152	1.43708E-06	4.19E-05
SE	chr1	Fastkd2	+	63735463-63735571:63735845-63735968:63737779-63737918	272	149	1	0.848	0.152	0.152	0.000198159	0.002997
A3SS	chr8	Atp11a	+	12859314-12859479:12861640-12868728:12863759-12868728	298	149	0.9463	0.795	0.151	0.151	0.000286892	0.005687
A3SS	chr19	Rbm4b	+	4761977-4762647:4764350-4765940:4765263-4765940	298	149	0.976	0.8247	0.151	0.151	1.01208E-06	5.11E-05
A5SS	chr7	Uros	-	133691086-133691166:133690863-133691166:133690508-133690593	298	149	0.1927	0.042	0.151	0.151	5.77316E-15	1.4E-12
MXE	chr2	Kcnt1	+	25910993-25911356:25912655-25912717:25915779-25915866:25915997-259	211	236	0.47	0.319	0.151	0.151	0.003676708	0.0414
MXE	chr9	Ireb2	+	54882319-54882456:54885875-54885944:54886490-54886673:54887268-548	218	298	0.0133	0.164	-0.151	0.151	4.32987E-15	1.27E-12
MXE	chr13	Atxn1	-	45735115-45735178:45795509-45795637:45852328-45852456:45956455-459	277	277	0.5873	0.738	-0.151	0.151	0.00473135	0.049885
RI	chr9	Ccpg1	+	73011930-73013326:73011930-73015429:73015332-73015429	298	149	0.5253	0.3743	0.151	0.151	6.67199E-05	0.000607
RI	chr11	Wnk4	+	101276828-101276883:101276828-101277409:101277033-101277409	297	149	1	0.8487	0.151	0.151	2.99666E-06	4.1E-05
RI	chr5	Camkk2	-	122731170-122734245:122731170-122737030:122736988-122737030	298	149	0.7713	0.922	-0.151	0.151	0.001358416	0.008253
SE	chr3	Rwdd3	-	121158768-121159255:121171112-121171182:121171592-121171695	219	149	0.0377	0.1887	-0.151	0.151	3.72799E-06	9.6E-05
SE	chr2	Anapc1	-	128680109-128680209:128680394-128680445:128684502-128684663	200	149	0.987	0.8357	0.151	0.151	2.09388E-13	2.42E-11
SE	chr18	Osbpl1a	-	12892167-12892289:12893637-12893720:12898203-12898301	232	149	0.091	0.2417	-0.151	0.151	0.000130011	0.002083
SE	chr14	Sec24c	+	20683885-20684021:20685212-20685280:20686509-20686620	217	149	0.6857	0.5343	0.151	0.151	0.000791441	0.009799
SE	chr10	Med16	-	79907039-79907146:79907491-79907653:79908818-79908938	298	149	0.9827	0.832	0.151	0.151	6.15918E-12	5.51E-10
SE	chr1	Kif1a	-	93025621-93025735:93026832-93026855:93036846-93036951	172	149	0.4777	0.3267	0.151	0.151	2.20383E-06	6.08E-05
SE	chr5	Arap2	-	62638962-62639036:62641328-62641396:62642795-62642942	217	149	0.9417	0.7903	0.151	0.151	0.000965499	0.011577
SE	chr15	Zcrb1	-	93391051-93391158:93391454-93391565:93395587-93395615	260	149	0.897	0.746	0.151	0.151	8.92619E-14	1.09E-11
SE	chr2	Cdk5rap1	-	154345866-154346015:154348651-154348781:154350818-154350873	279	149	0.9787	0.8273	0.151	0.151	0.000238887	0.003518
SE	chr13	Vcan	-	89688285-89693501:89702918-89705797:89712205-89712498	298	149	0.706	0.8573	-0.151	0.151	0.001661072	0.018272
SE	chr6	Ccdc136	+	29417072-29417578:29421991-29422097:29426625-29426953	255	149	0.8663	0.7153	0.151	0.151	0.005077224	0.046771
SE	chr5	Chfr	+	110151527-110151686:110152336-110152490:110153118-110153280	298	149	0.9893	0.8383	0.151	0.151	0	0
SE	chr1	Rftn2	-	55195516-55195637:55201345-55201422:55204183-55204392	226	149	0.0667	0.2177	-0.151	0.151	0.002116208	0.02241
SE	chr5	Actr3b	+	25832386-25832478:25849073-25849156:25849851-25850688	232	149	1	0.8493	0.151	0.151	4.13715E-08	1.75E-06
SE	chr19	Pi4k2a	+	42115885-42116018:42116507-42116566:42119793-42119984	208	149	0.948	0.7967	0.151	0.151	3.79674E-07	1.28E-05
SE	chr10	Hbs1l	+	21352542-21352610:21354639-21354743:21358313-21358403	253	149	0.998	0.8473	0.151	0.151	0	0
SE	chr14	Msra	-	64313687-64313755:64440710-64441026:64450964-64451038	298	149	0.8487	1	-0.151	0.151	1.05477E-06	3.2E-05
