# Supplementary material for: Deletion of ARGLU1 causes global defects in alternative splicing in vivo and mouse cortical malformations primarily via apoptosis
Source: Cell Death Dis. 2023 Aug 23;14(8):543. doi: 10.1038/s41419-023-06071-w (PMC10447433; doi:10.1038/s41419-023-06071-w)

Uncropped original western blots in Figure 4E

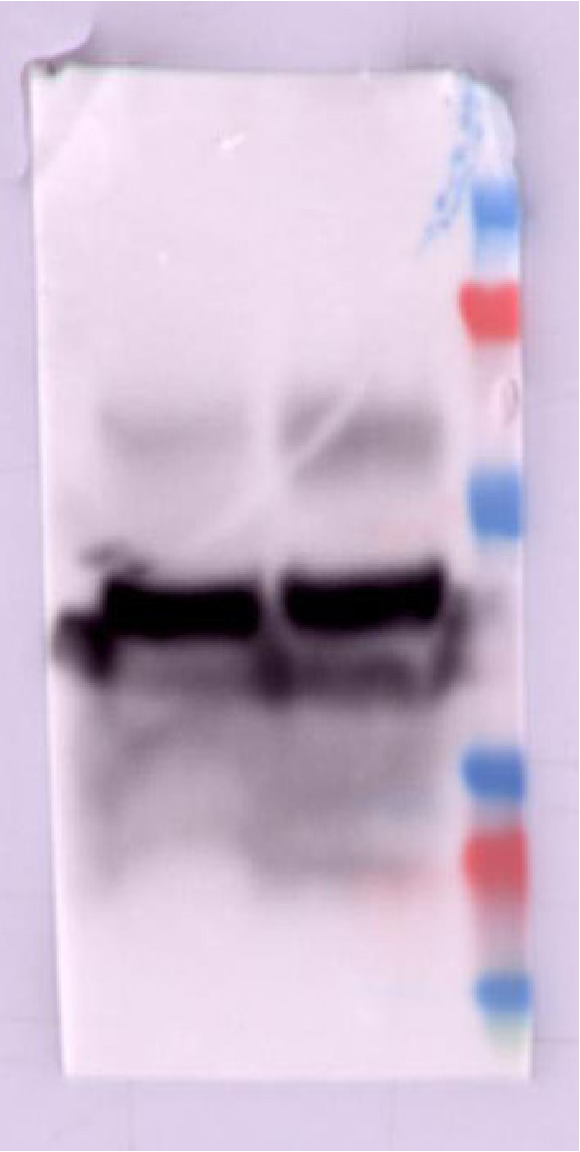

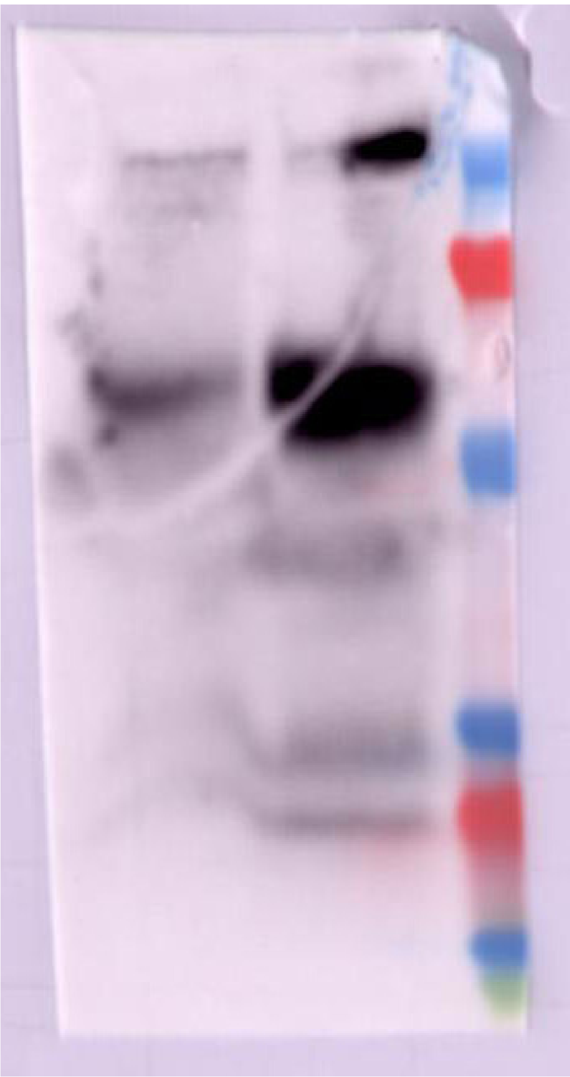

Uncropped original western blots in Figure 4G

PUMA

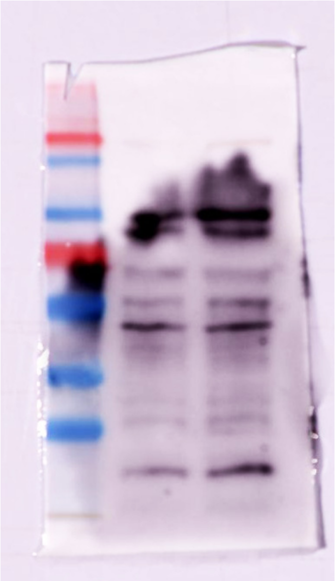

Actin corresponding to PUMA

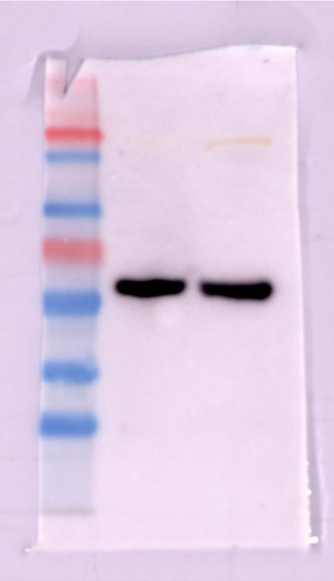

NOX1

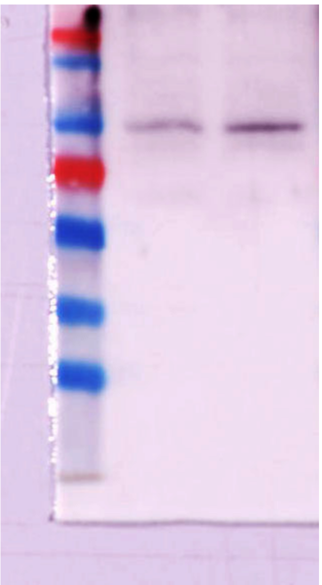

Actin corresponding to NOX1

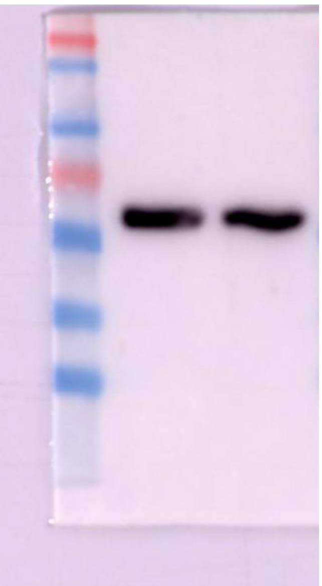

P21

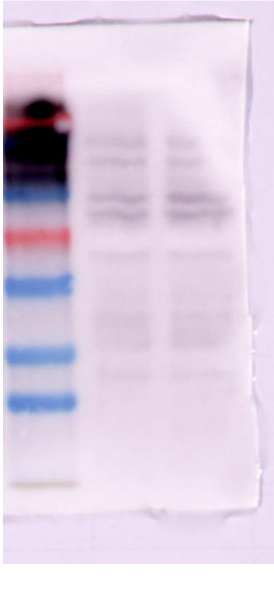

Actin corresponding to P21

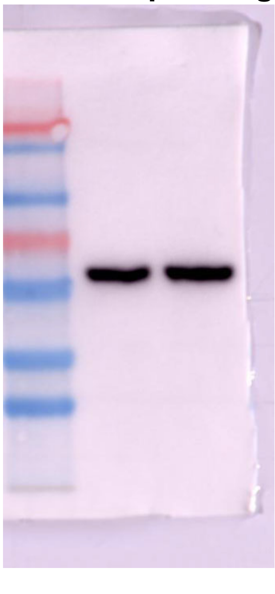

Uncropped original western blots in Figure 5G

Actin corresponding to Mdm2

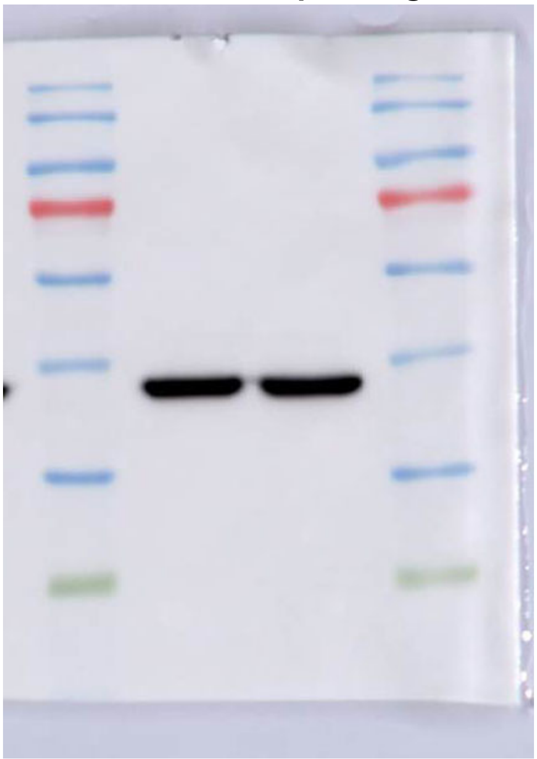

Mdm2

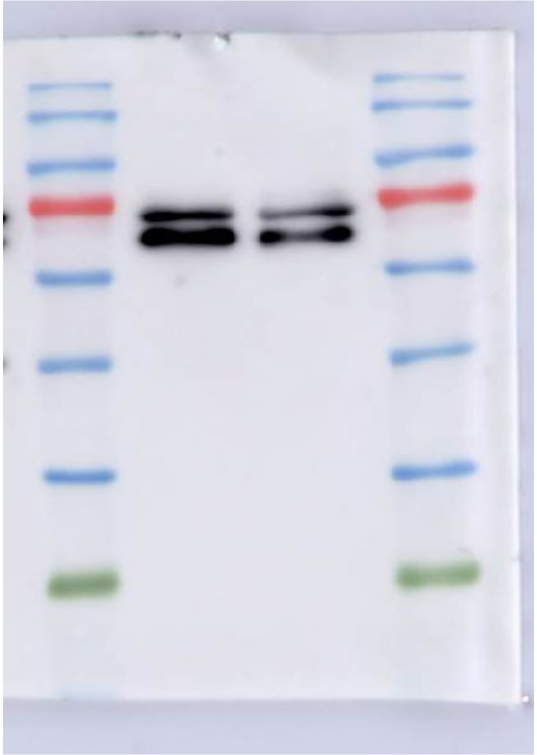

Actin corresponding to Mdm4

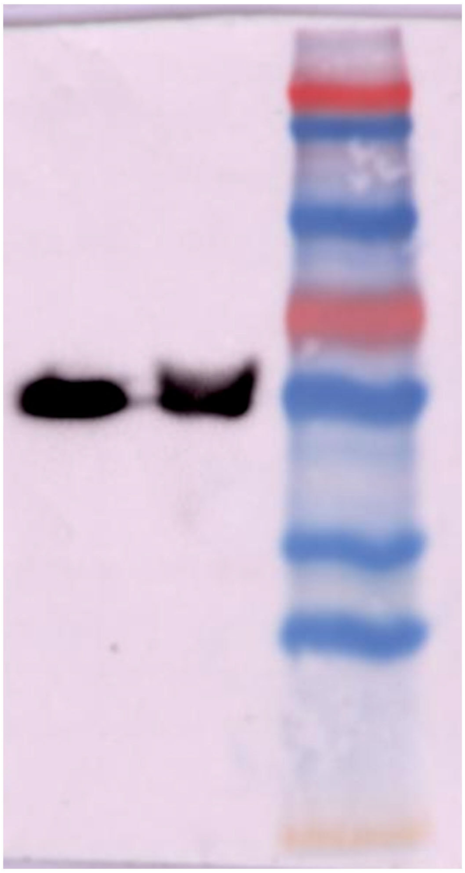

Mdm4

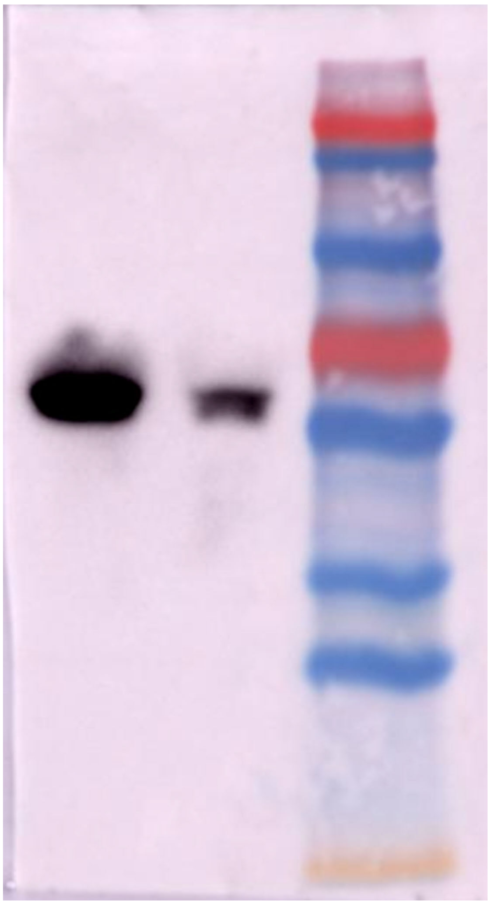

Uncropped original western blots in Supplementary Figure 6B

p53

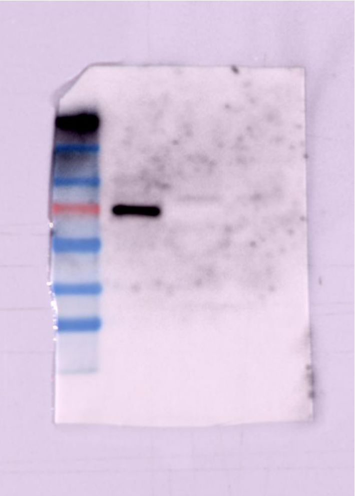

Actin corresponding to p53

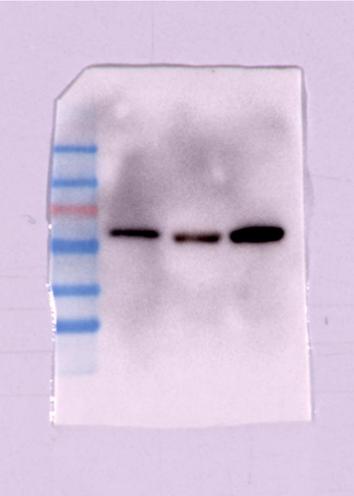

Uncropped original western blots in Supplementary Figure 9E

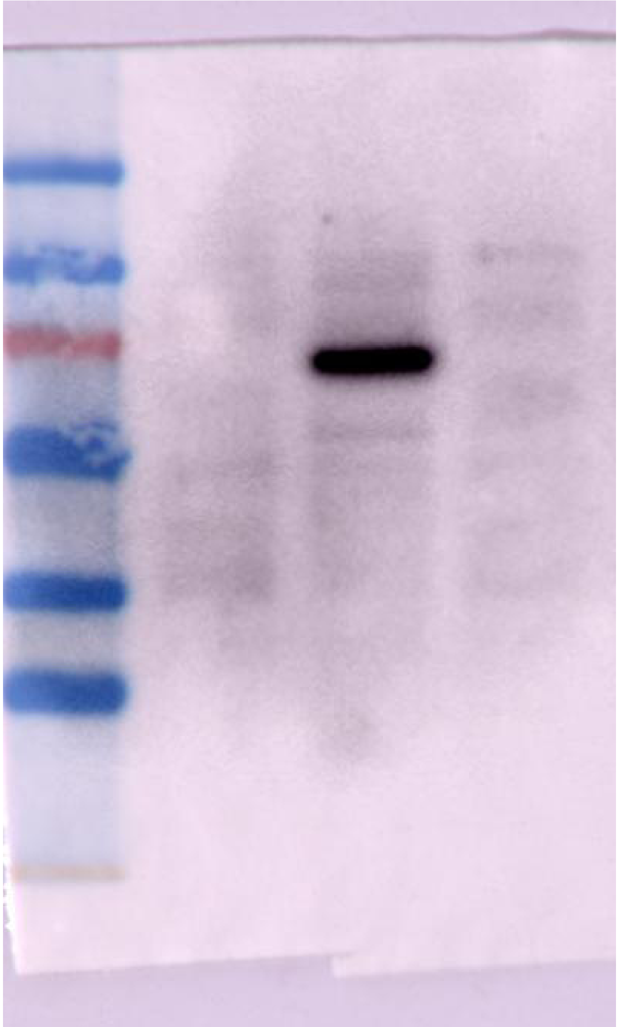

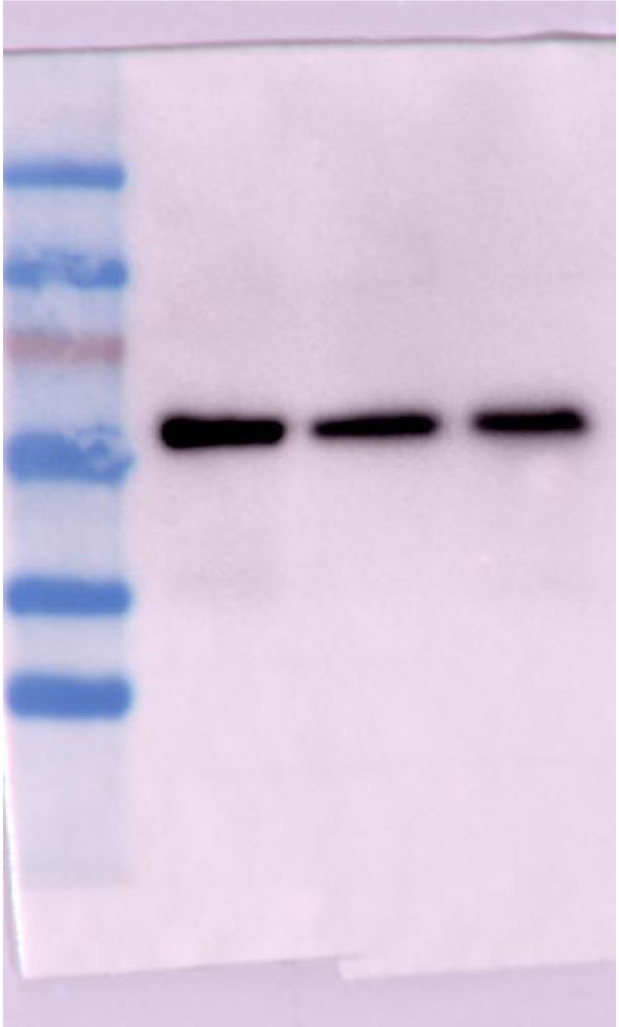

Supplement: Supplementary file 2 — Original WB Data File [file 41419_2023_6071_MOESM2_ESM.pdf]
